# Causal relationship in gut microbiota and upper urinary urolithiasis using Mendelian randomization

**DOI:** 10.3389/fmicb.2023.1170793

**Published:** 2023-05-18

**Authors:** Ruiqiao Zhang, Weijie Zhao, Ruijie Zhao, Yunhai Zhao, Yanlong Zhang, Xuezhi Liang

**Affiliations:** ^1^Department of Urology Surgery, First Hospital of Shanxi Medical University, Taiyuan, Shanxi, China; ^2^The First Clinical Medical College, Shanxi Medical University, Taiyuan, Shanxi, China; ^3^Department of Urology Surgery, Capital Medical University, Beijing, China

**Keywords:** upper urinary urolithiasis, renal stones, gut microbiota, Mendelian randomization, causal relationship, inverse variance weighted

## Abstract

**Background:**

Several reports in recent years have found an association between gut microbiota and upper urinary urolithiasis. However, the causal relationship between them remains to be clarified.

**Methods:**

Genetic variation is used as a tool in Mendelian randomization for inference of whether exposure factors have a causal effect on disease outcomes. We selected summary statistics from a large genome-wide association study of the gut microbiome published by the MiBioGen consortium with a sample size of 18,340 as an exposure factor and upper urinary urolithiasis data from FinnGen GWAS with 4,969 calculi cases and 213,445 controls as a disease outcome. Then, a two-sample Mendelian randomization analysis was performed by applying inverse variance-weighted, MR-Egger, maximum likelihood, and weighted median. In addition, heterogeneity and horizontal pleiotropy were excluded by sensitivity analysis.

**Results:**

IVW results confirmed that class *Deltaproteobacteria* (OR = 0.814, 95% CI: 0.666–0.995, *P* = 0.045), order *NB1n* (OR = 0.833, 95% CI: 0.737–0.940, *P* = 3.15 × 10^−3^), family *Clostridiaceae1* (OR = 0.729, 95% CI: 0.581–0.916, *P* = 6.61 × 10^−3^), genus *Barnesiella* (OR = 0.695, 95% CI: 0.551–0.877, *P* = 2.20 × 10^−3^), genus *Clostridium sensu_stricto_1* (OR = 0.777, 95% CI: 0.612–0.986, *P* = 0.0380), genus *Flavonifractor* (OR = 0.711, 95% CI: 0.536–0.944, *P* = 0.0181), genus *Hungatella* (OR = 0.829, 95% CI: 0.690–0.995, *P* = 0.0444), and genus *Oscillospira* (OR = 0.758, 95% CI: 0.577–0.996, *P* = 0.0464) had a protective effect on upper urinary urolithiasis, while *Eubacterium xylanophilum* (OR =1.26, 95% CI: 1.010–1.566, *P* = 0.0423) had the opposite effect. Sensitivity analysis did not find outlier SNPs.

**Conclusion:**

In summary, a causal relationship was found between several genera and upper urinary urolithiasis. However, we still need further randomized controlled trials to validate.

## 1. Introduction

Urinary stones are one of the most frequent benign diseases with a high incidence of up to 20% worldwide (Hoffman et al., 2021). The most troubled and most studied of urolithiasis is the upper urinary stones, including nephrolithiasis and ureterolithiasis derived from the renal. The prevalence of renal stones is projected to rise further as the growing population with associated diseases including diabetes and hypertension and the changing environmental trends of global warming (Carbone et al., 2018; Johnson et al., 2019). It will likely cause complications including urinary obstruction, infection, pain, and even permanent damage to renal function (Rule et al., 2020). Renal stones are also generally considered to be a lifelong disease with high recurrence (Corbo and Wang, 2019), which has a tremendous influence on individuals and society, and has emerged as a substantial public health issue (Johnson et al., 2019). The mechanism of formation and growth of renal stones is complicated. A variety of processes including supersaturation of urinary stone components, reduction of inhibitors of stone formation (Cicerello et al., 2019), and renal tubular epithelial cell injury (Aggarwal et al., 2013) are involved. Metabolism and inflammation are considered important factors involved in the formation of renal stones (Tian et al., 2022; Capolongo et al., 2023).

The gut microbiota is the largest micro-ecosystem of the human body, participating in and influencing the metabolism of the substance and energy (Anand and Mande, 2022). Crosstalk between the gut microbiome and kidney has been widely documented, for example, intestinal ecological disorders are often found in patients with chronic kidney disease (Voroneanu et al., 2023). The role of gut microbiome in the pathogenesis of kidney stones has also attracted more and more attention. There are significant changes in gut microbiota in patients with and without renal calculi (Siener et al., 2013b; Stern et al., 2016; Tavasoli et al., 2020; Kim et al., 2022). Furthermore, the normal group had a greater abundance of Bifidobacterium (Kim et al., 2022). Stern reported that Bacillus was 3.4 times more abundant in the stone group and Prevotella was 2.8 times more abundant in the non-stone group (Stern et al., 2016). Some microbial producers of short-chain fatty acids deserved our attention, and an observational study found that the proportion of some key taxa responsible for the production of short-chain fatty acids decreased in groups with nephrolithiasis (Liu et al., 2020). Moreover, many studies have focused on Oxalobacter formigenes, Bifidobacterium, and Lactobacillus due to the oxalic acid-degrading ability (Siener et al., 2013a; Tavasoli et al., 2020). Oxalobacter formigenes can stimulate the secretion of oxalate in the colon, thereby reducing oxalic acid levels in the urine (Allison et al., 1985). Some studies found that individuals with oxalate stones had considerably greater urine oxalate excretion and very low levels of Oxalobacter formigenes compared to controls, so it can be assumed that the formation of oxalate stones is related to the lack of colonization by Oxalobacter formigenes (Siener et al., 2013b; Tavasoli et al., 2020). However, clinical supplementation with Oxalobacter reagents did not improve blood and urine oxalate levels in patients with hyperoxaluria in another study (Siener et al., 2013b). Therefore, exploring the causal relationship between gut microbiota and calculi may provide new targets and ideas for the prevention and treatment of upper urinary urolithiasis.

In conclusion, since the gut microbiota is a complex ecosystem, there may be regulatory networks among various types of bacteria as well as the presence of some confounding factors that limit the causal inference between intestinal flora and renal calculi disease. Mendelian Randomization (MR) can be employed to infer the causal link between exposure factors and disease through genetic variation. MR provides a more convenient method for exploration of potential protective and risk factors for disease and has been applied to several research studies on the relationship between gut microbiota and diseases (Freidin et al., 2021; Liu et al., 2022; Luo et al., 2022). The genome-wide association study (GWAS) summary datasets about gut microbiota and renal and ureter stones were applied to this analysis.

## 2. Materials and methods

### 2.1. Data source

**Figure 1:**
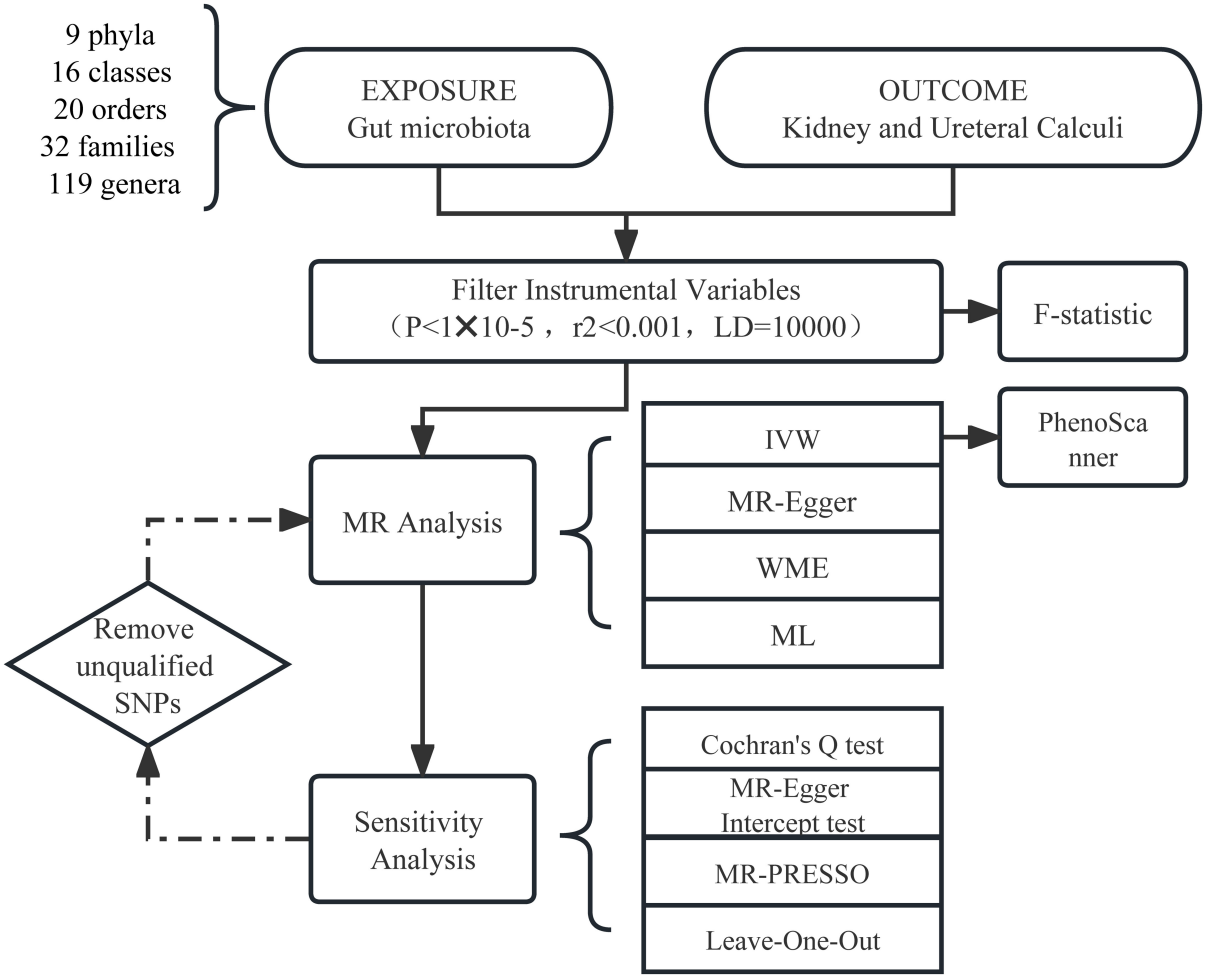
Workflow of the MR analysis.

Our workflow diagram is presented in Figure 1. The MiBioGen group released the biggest genome-wide meta-analysis of gut microbiota composition, which included genetic variation data for the gut microbiota (Kurilshikov et al., 2021). The research contains 16SrRNA gene sequencing profiles and genotyping data from 18,340 individuals from the United States, the United Kingdom, Finland, Sweden, Denmark, the Netherlands, and other countries. Nine phyla, 16 classes, 20 orders, 35 families, and 131 genera (Supplementary Table 1) of bacteria were classified in the summary data of this study. Then, we excluded three unknown families and 12 unknown genera. Since genus is the minimal level of bacterial classification, we enrolled nine phyla, 16 classes, 20 orders, 32 families, and 119 genera in the subsequent MR analysis.

Several summary statistics for urolithiasis were available (Supplementary Table 2), and to ensure the credibility of the data, we chose GWAS data with the highest number of SNPs published in 2021 from FinnGen (Kurki et al., 2023). The phenotype “Calculus of renal and ureter” was used, and this GWAS had 218,414 Finnish adult subjects including 4,969 cases and 213,445 controls.

### 2.2. Filter instrumental variables

Gut microbiota and urolithiasis were exposure factors and outcomes, respectively. Valid IVs must satisfy three key assumptions (Slob and Burgess, 2020): (1) The correlation hypothesis: instrumental variables are strongly correlated with exposure. (2) The exclusivity hypothesis: instrumental variables are independent of the outcome. (3) The independence assumption: instrumental variables are independent of confounding factors. Thus, we made the following criteria. A *P*-value of < 1^*^10^−5^ was chosen as the significance threshold to avoid too few single-nucleotide polymorphisms (SNPs) (Sanna et al., 2019). Linkage disequilibrium (LD) is a phenomenon in which two genes at different seats in a population are inherited at a significantly higher frequency than would be expected at random (Roze, 2023). To avoid LD, we set the chain imbalance threshold r^2^ <0.001 and the distance to 10,000 kb. Palindromic variation means if the alleles are A and T (or C and G), then the same alleles will appear on the plus and minus chains (Girault and Ménigot, 2022). Thus, Palindromic SNPs were removed to prevent inconsistent SNP orientation in the exposure and outcome. Second, IVs should fulfill a *P*-value of >1^*^10^−5^ in the outcome for the *p*-value according to assumption (2). The PhenoScanner (Kamat et al., 2019) online tool was used to inquire whether the SNPs were related to confounders of urolithiasis according to the European Association of Urology Guidelines section on urolithiasis (Zeng et al., 2022). Then we excluded the relevant SNPs.

### 2.3. Statistical analysis

The inverse variance-weighted (IVW) approach was used as the principal analysis method, while three other methods, namely MR-Egger regression, weighted median analysis (WME), and maximum likelihood (ML), were used as secondary references. For exposure factors with individual SNP, the IVW technique provided a consistent estimate when all SNPs were believed to be genuine and the presence of an intercept term was not taken into account. The WME method was premised on the assumption that over half of the SNPs had valid IVs (Bowden et al., 2016). The MR-Egger method assumes that all SNPs were invalid instrumental variables and defaulted to the presence of an intercept term (Bowden et al., 2015). Further sensitivity analysis was carried out only when the IVW results were meaningful.

The F-statistic was utilized to determine the intensity of IVs (F $=\frac{R^{2}\times \left(n-k-1\right)}{\left(1-R^{2}\right)\times K}$ denotes the fraction of variance explained by genetic variation in exposure, *n* means sample size, and *k* represents the number of SNPs) (Pierce et al., 2011). When the F-statistic for SNP was more than 10, it was assumed that there was no substantial weak instrumental bias; otherwise, the instrumental variable should be omitted. After removing the corresponding IVs that did not qualify as described above, the MR analysis was rerun to acquire the final MR estimations. When there did not exist heterogeneity and pleiotropy, the IVW results were trustworthy (Bowden et al., 2015). Effect estimates were expressed as odds ratio for binary outcomes.

### 2.4. Sensitivity analysis

The sensitivity analysis involved a heterogeneity test and a multiplicity of validity test. Cochran's Q-test was performed to confirm IV heterogeneity, with a *p*-value <0.05 indicating the lack of heterogeneity. MR-PRESSO summed the residuals for each SNP to assess the magnitude of horizontal pleiotropy. The MR-PRESSO outlier test allowed the assessment of outlier SNPs that contributed to the presence of pleiotropy at the overall level. The impact of one outlier on the overall results was assessed by calculating the remaining SNP effects after removing individual SNPs one by the leave-one-out analysis. Both MR-PRESSO and leave-one-out analysis methods could identify and remove SNPs that exhibited pleiotropy or heterogeneity. MR Steiger test was also carried out (Xue and Pan, 2020) to investigate the correctness of the causal direction. Additionally, we performed the reverse Mendelian randomization analysis.

MR analyses were carried out using the R (version 4.1.2) computational environment and the TwoSampleMR (version 0.5.6) and MR-PRESSO packages (version 1.0). The R package “ggplot2” was applied for drawing some figures. For evidence of causal effects, a *p*-value of <0.05 was judged statistically significant.

## 3. Results

**Figure 2:**
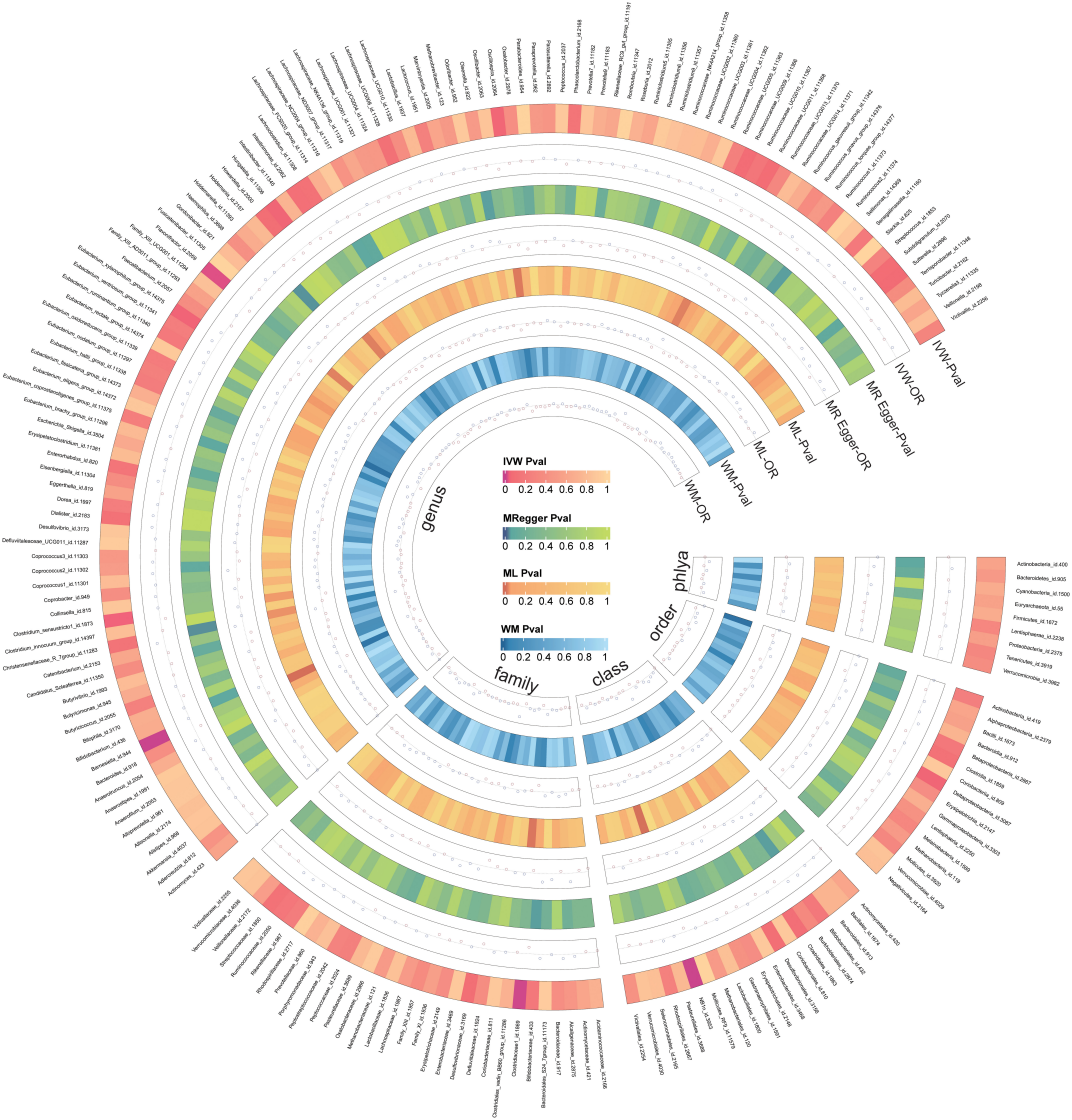
The circus plot showing four method results of all gut microbiota.

**Figure 3:**
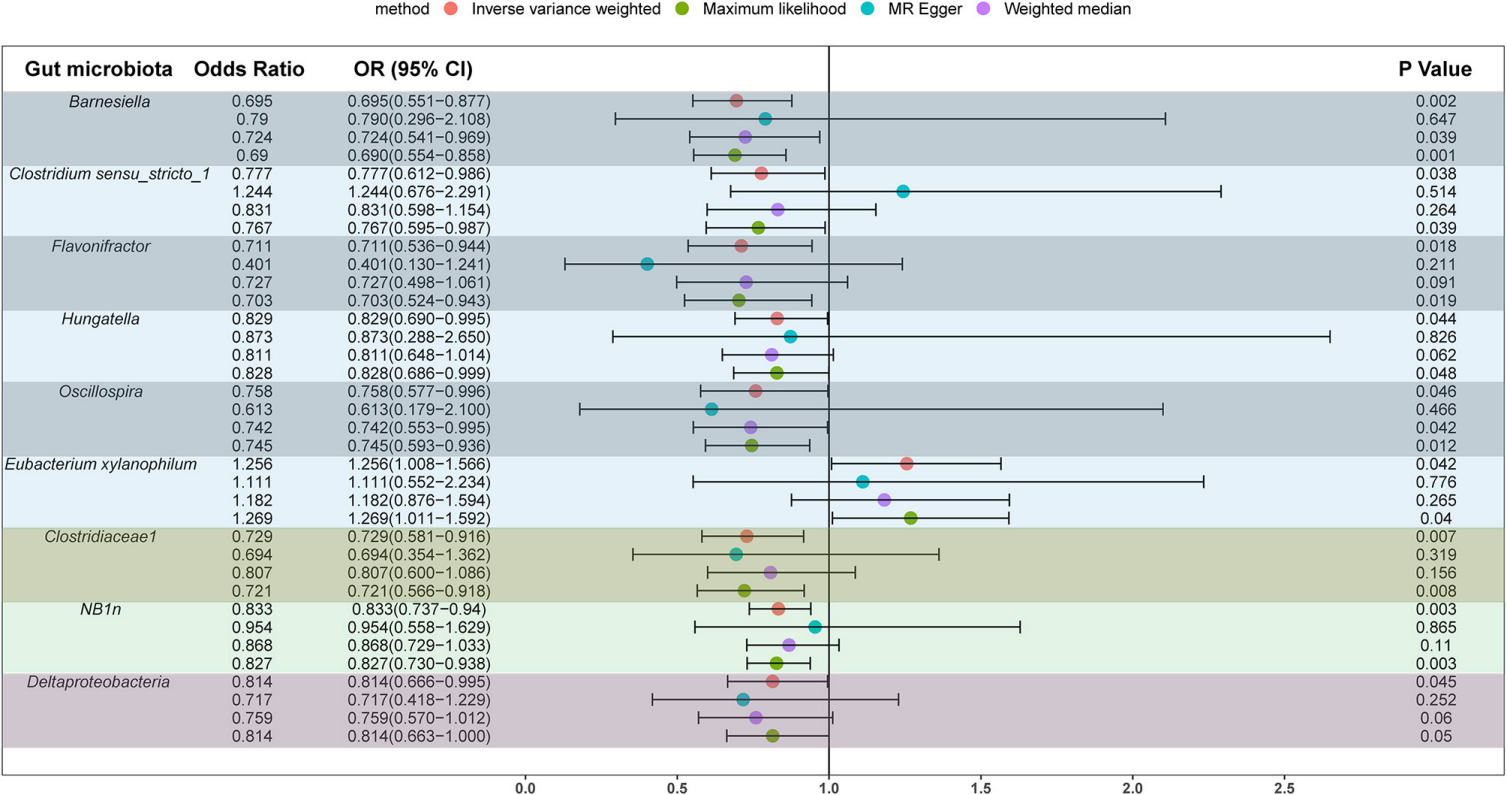
Significant results for the IVW analysis.

We screened 2,104 SNPs as instrumental variables from 196 gut microbiota. F-statistics for all instrumental variables were calculated. Two SNPs (rs17074066 and rs2835874) not satisfying F-statistic > 10 were excluded (Supplementary Table 3). The results of the MR analysis for IVs are shown in a circus plot (Figure 2) and detailed in Supplementary Table 4. We queried the aforementioned SNPs for positive findings in PhenoScanner and found no SNPs associated with the aforementioned confounders (Supplementary Table 5). Finally, one class, one order, one family, and six genera showing significant results for IVW analysis (Figure 3) were class *Deltaproteobacteria* (OR = 0.814, 95% CI: 0.666–0.995, *P* = 0.045), order *NB1n* (OR = 0.833, 95% CI: 0.737–0.940, *P* = 3.15 × 10^−3^), family *Clostridiaceae1* (OR = 0.729, 95% CI: 0.581–0.916, *P* = 6.61 × 10^−3^), genus *Barnesiella* (OR = 0.695, 95% CI: 0.551–0.877, *P* = 2.20 × 10^−3^), genus *Clostridium sensu_stricto_1* (OR = 0.777, 95% CI: 0.612–0.986, *P* = 0.0380), genus *Flavonifractor* (OR = 0.711, 95% CI: 0.536–0.944, *P* = 0.0181), genus *Hungatella* (OR = 0.829, 95% CI: 0.690–0.995, *P* = 0.0444), genus *Oscillospira* (OR = 0.758, 95% CI: 0.577–0.996, *P* = 0.0464), and genus *Eubacterium xylanophilum* (OR =1.26, 95% CI: 1.010–1.566, *P* = 0.0423).

**Figure 4:**
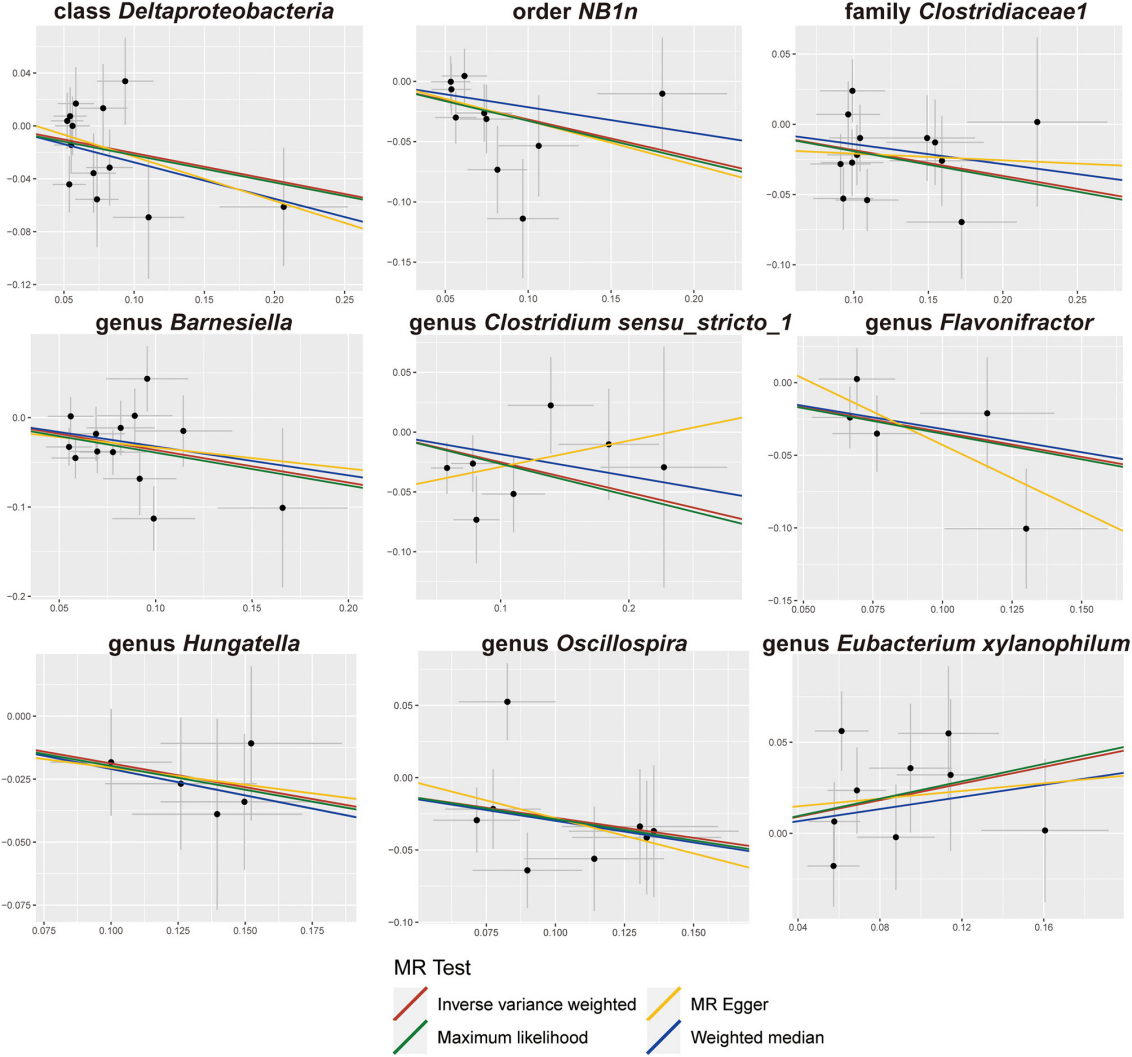
Scatter plots of the MR analysis.

**Figure 5:**
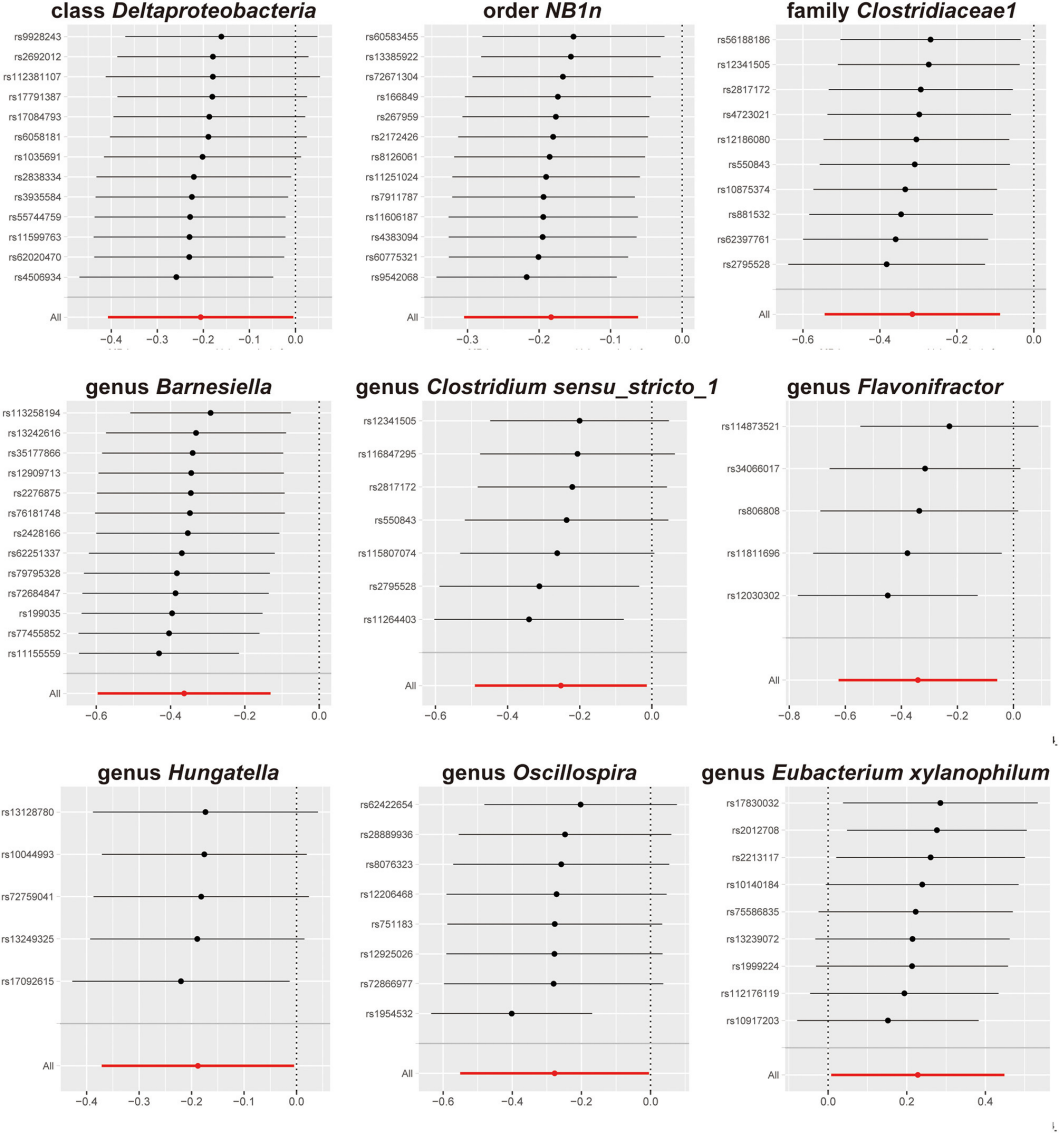
Leave-one-out results.

The results of sensitivity analyses are presented in Table 1. Cochran's Q-test results revealed no significant values for any of these gut microbiota, indicating that none of the IVs were heterogeneous. MR-PRESSO results did not show the presence of outliers. MR-Egger's intercept analysis had no meaningful results and demonstrated that there was no horizontal pleiotropy (Figure 4) and the directions calculated by each method were consistent except for the genus *Clostridium sensu_stricto_1*. The effect value calculated using the MR-Egger method for Clostridia was not consistent with the other three methods. Considering that the MR-Egger method assumed that all IVs were invalid, which weakened the statistical power making the results less precise. Therefore, we primarily used it to assess horizontal pleiotropy. As shown in Figure 5, the results of the leave-one-out technique were really robust to the outcomes of this MR analysis, where no matter which SNP was removed, it did not have a fundamental effect on the results.

**Table 1:** Sensitivity analysis of significant gut microbiota.

**Gut microbiota**		**Q_pval (IVW)**	**MR PRESSO**	**MR-egger_test**		**Steiger test**
				**Intercept**	**Pleiotropy test**	
Genus	Barnesiella	0.231	0.269	−0.010	0.797	8.67E-63
	Clostridium sensus_tricto_1	0.426	0.471	−0.051	0.161	3.54E-36
	Flavonifractor	0.445	0.492	0.048	0.380	5.58E-33
	Hungatella	0.972	0.973	−0.007	0.932	7.97E-21
	Oscillospira	0.141	0.166	0.021	0.741	8.74E-36
	Eubacterium xylanophilum	0.424	0.453	0.011	0.725	5.38E-49
Class	Deltaproteobacteria	0.478	0.511	0.010	0.627	4.24E-56
Family	Clostridiaceae1	0.514	0.569	0.004	0.882	5.02E-42
Order	NB1n	0.430	0.471	−0.016	0.619	1.09E-64

**Figure 6:**
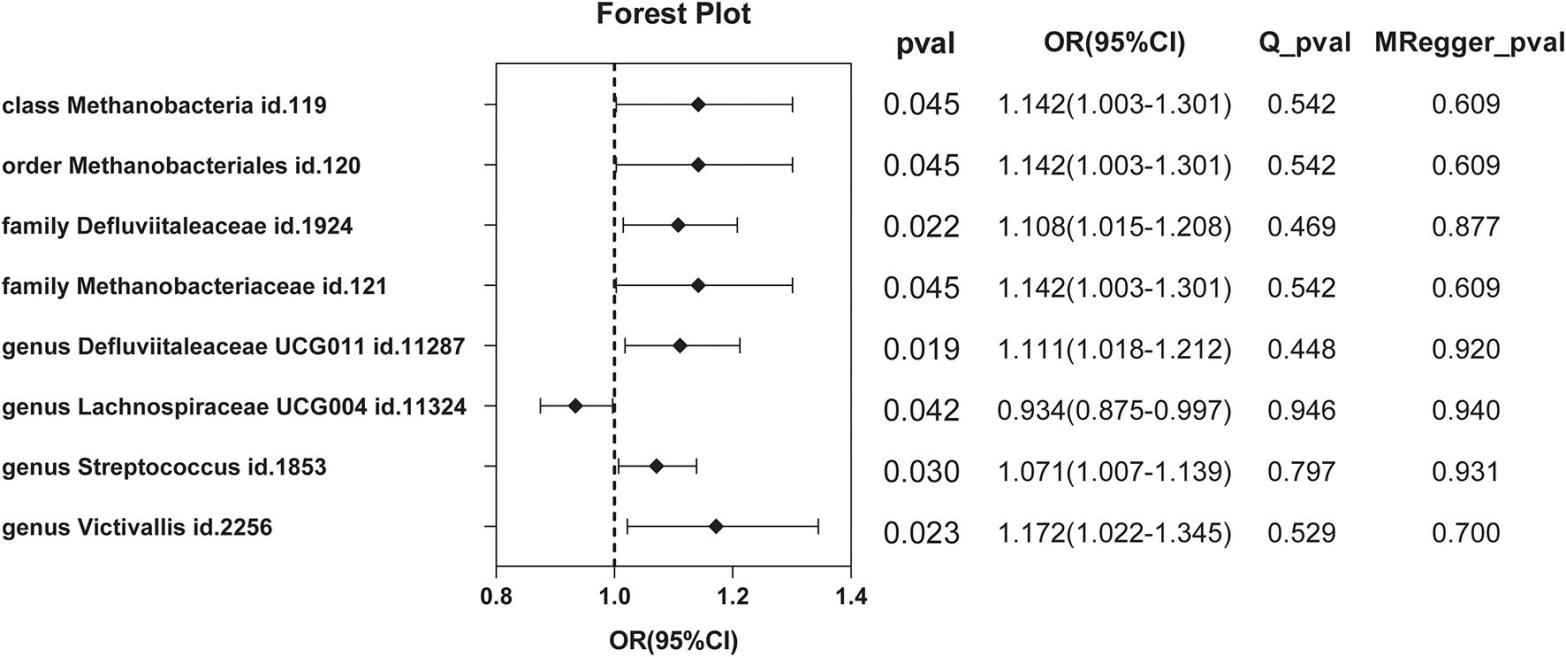
Significant results of reverse MR by the IVW method.

A total of nine SNPs were used as instrumental variables for each reverse Mendelian randomization (Supplementary Table 6). Significant results of the IVW method (Figure 6) showed class *Methanobacteria* (OR = 1.142, 95% CI: 1.003–1.301, *P* = 0.045), order *Methanobacteriales* (OR = 1.142, 95% CI: 1.003–1.301, *P* = 0.045), family *Methanobacteriaceae* (OR = 1.142, 95% CI: 1.003–1.301, *P* = 0.045), family *Defluviitaleaceae* (OR =1.108, 95% CI: 1.015–1.208, *P* = 0.022), genus *Defluviitaleaceae UCG001* (OR =1.111, 95% CI: 1.018–1.212, *P* = 0.019), genus *Lachnospiraceae UCG004* (OR = 0.934, 95% CI: 0.875–0.997, *P* = 0.042), genus *Streptococcus* (OR =1.071, 95% CI: 1.007–1.139, *P* = 0.030), and genus *Victivallis* (OR = 1.172, 95% CI: 1.022–1.345, *P* = 0.023).

## 4. Discussion

The gut microbiome is associated with several diseases in humans. Since the theory of the kidney gut axis became available, several clinical and animal model studies have confirmed the association of the gut microbiome with kidney diseases, especially chronic kidney disease (Cigarran Guldris et al., 2017; Yang et al., 2018). The relationship between intestinal flora and renal and ureter stones has also been gradually recognized in recent years. However, the correct direction of causality could not be inferred from observational studies. Our study was the first to confirm a causal relationship between gut microbiota and upper urinary tract stones using an MR analysis.

In this study, we used summary information on the gut microbiota from the largest GWAS meta-analysis conducted by the MiBioGen Consortium and summary statistics on upper urinary urolithiasis released by FinnGen R8 to investigate the causal link. Mendelian randomization and sensitivity analysis were performed on the filtered qualifying instrumental variables, and we found a causal relationship between several gut microbiota for upper urinary urolithiasis, with high levels of the genera *Barnesiella, Clostridium sensu_stricto_1, Flavonifractor, Hungatella, Oscillospira*, family *Clostridiaceae1*, class *Deltaproteobacteria*, and order *NB1n*, reducing the risk of upper urinary tract stones (OR <1), whereas the genus *Eubacterium xylanophilum* (OR > 1) had the opposite effect.

The association between these florae in our findings and upper urinary tract stones has rarely been reported. However, risk-associated flora is often reported to be associated with some inflammation-related diseases. For instance, *Barnesiella* is anti-inflammatory and protective in animal models and is associated with several immunomodulatory cells. Higher levels of *Barnesiella* in the colon are correlated with the intestinal environment less prone to inflammation (Berry and Reinisch, 2013). The main products of *Barnesiella* derivation are butyric acid and isobutyric acid (Sakamoto et al., 2009), and butyrate stimulates GFR receptors, which limit the production of inflammatory proteins such as IL-6, IL-1, and NF-B, reducing inflammation (Chen et al., 2018; Clemente et al., 2018). *Clostridium sensu_stricto_1* belonging to the family *Clostridiaceae* is one of the most important anaerobic bacteria in the intestine (Guo et al., 2020) and has positive effects on short-chain fatty acid production and immune regulation through the production of butyrate through fermentation (Vital et al., 2014; Li et al., 2019). Bioinformatics analysis of the phylum Firmicutes (Rawat et al., 2022) showed that their genomes, particularly the genus *Hungatella*, are a rich source of glycosaminoglycan-specific catabolic enzymes and that the interaction of glycosaminoglycans with many ligands is relevant to the biological function of inflammation. Li found that feeding *Hungatella* to a mouse model ameliorated inflammation and extracellular matrix remodeling (He et al., 2016). *Flavonifractors* have been suggested in several articles to affect inflammation and obesity through multiple mechanisms (Kasai et al., 2015; Mikami et al., 2020; Ogita et al., 2020). Animal experiments by Tasuku (Mikami et al., 2020) have demonstrated that this genus suppresses allergen-specific IgE synthesis and may contribute to the relief of antigen-specific immunological responses in a Th2-dominated environment. Another study (Ogita et al., 2020) found that oral treatment of *Flavonifractor* preparations reduced the inflammatory response in the adipose tissue of obese mice, raising the possibility that it is involved in the suppression of TNF expression in an inflammatory environment. Mice treated with *Hungatella* exhibited reduced cytokine release and NF-κB activation in dendritic cells (Rossi et al., 2016). Oscillospira, a genus capable of synthesizing short-chain fatty acids like butyrate, has been linked to inflammation-related disorders, such as inflammatory bowel disease, non-alcoholic fatty liver disease, and aging processes distinguished through increased levels of circulating inflammatory mediators (Chierico et al., 2017; Lima and Longman, 2021; Xu et al., 2021), and is strongly negatively associated with pro-inflammatory monocyte chemoattractant protein-1 (Buford, 2017). *Deltaproteobacteria* were found to be negatively related to antineutrophil cytoplasmic antibody-associated vasculitis (AAV) with kidney injury (Yu et al., 2022). The herbal tea ingredient Rabdosia serra acts as an anti-inflammatory agent by boosting the number of helpful bacteria, such as Lactobacillus, as well as reducing the number of harmful bacteria including *Eubacterium xylanophilum*, thus alleviating artificially induced colitis in mice (Li et al., 2022).

It is believed that in the prevalent explanation of kidney stone production, the inflammatory immunological response contributes to the creation of Randall's plaques and calcium stones. Crystal deposition in mouse kidneys has been linked to reactive oxygen species generation, inflammatory vesicle activation, and the increased expression of molecules involved in the inflammatory cascade response (Khan et al., 2021). The renal—intestinal axis theory suggests that inflammatory cells, cytokines, soluble urokinase produced in our intestines promote renal inflammation *via* the circulation and metabolites of the microbiota entering the circulation may also have an impact on the kidney (Ticinesi et al., 2018). This is laterally supported by the higher prevalence of urinary stones in the inflammatory bowel population than in the general population (Dimke et al., 2021). Genera with protective effects usually exhibit anti-inflammatory actions in this study. Because this anti-inflammatory effect may depend on butyrate, a kind of SCFs, we hypothesized that the gut microbiota may influence the development of kidney stones by altering the level of inflammation in the body.

Reverse MR analysis revealed that several gut microbiota had the propensity to colonize in the gut of patients with kidney and ureteral stones. This may be related to the dietary habits and antibiotic usage of the patients. For example, increased abundances of *Methanobacteria* and *Victivallis* were found in mice fed with a high-fat diet (Mathur et al., 2013; Rodriguez et al., 2020) and a high sugar diet-induced changes in *Lachnospiraceae UCG004* (Han et al., 2022). An epidemiological survey revealed a greater predilection for a western-style diet (a diet high in fat, calories, and animal protein and low in fiber and plant-based proteins) among the population with kidney stones (Kohjimoto et al., 2013).

The major strength of our study was that MR analysis results were unlikely to be biased by confounders and reverse causation compared with conventional observational studies; thus, our results provided more convincing evidence to support the causality of gut microbiota and upper urinary tract stones. In addition, the data of both gut microbiota and upper urinary tract stones were obtained from a large sample population, which could greatly improve the MR analysis power based on the pooled data.

This study had some limitations. First, our study was conducted in a European population only and may not be applicable to other populations. Second, because the minimal taxonomic level was genus, we could not further explore the causal relationship between gut microbiota and upper urinary urolithiasis at the species level. Furthermore, summary statistics lacked grouping information for stone composition, such as calcium oxalate stones or uric acid stones; therefore, we were unable to perform subgroup analyses.

## 5. Conclusion

Finally, a causal relationship was established between upper urinary urolithiasis and the gut microbiota through two-sample MR. *Deltaproteobacteria, NB1n, Clostridiaceae1, Barnesiella, Clostridium sensu_stricto_1, Flavonifractor, Hungatella, Oscillospira*, and *Eubacterium xylanophilum* were identified. These strains may develop into new biomarkers and provide potential direction for the treatment and prevention of urinary stones. In addition, the mechanism and role of the inflammatory response in the formation of upper urinary tract stones deserve our attention.

## Data availability statement

The original contributions presented in the study are included in the article/Supplementary material, further inquiries can be directed to the corresponding author.

## Author contributions

RZhang and XL were responsible for the concept and design. XL, WZ, RZhao, and YZhao assisted with carrying out the analyses. YZhang assisted in revising the manuscript. RZhang and XL drafted the early version of the manuscript. All authors contributed to the article and approved the submitted version.
