# Supplementary material for: Causal relationship in gut microbiota and upper urinary urolithiasis using Mendelian randomization
Source: Front Microbiol. 2023 May 18;14:1170793. doi: 10.3389/fmicb.2023.1170793 (PMC10233049; doi:10.3389/fmicb.2023.1170793)
Supplement: Supplementary file 1 [file Table_1.DOCX]

Supplementary Material

**Causal Relationship in Gut Microbiota and Upper Urinary Urolithiasis Using Mendelian randomization**

**Ruiqiao Zhang^1，2^,Weijie Zhao^2^,Ruijie Zhao^2^,Yunhai Zhao^2^,Yanlong Zhang^3^，Xuezhi Liang^1*^**

^1^Department of Urology Surgery, First Hospital of Shanxi Medical University, Taiyuan of Shanxi Province,P.R.China.

^2^Department of Urology Surgery, Shanxi Medical University, Taiyuan of Shanxi Province,P.R.China.

^3^Department of Urology Surgery,Capital Medical University , Beijing,P.R.China.

***Correspondence:**

Xuezhi Liang

Address: No. 85, Jiefang South Road, Taiyuan City, Shanxi Province,China. Phone: +86 13007089928 ;Email:liangxz2008@sina.com

Supplementary Table 1:Summary of gut microbiota information.

| id | | trait | year | pmid | population | sample_size | nsnp |
| --- | --- | --- | --- | --- | --- | --- | --- |
| FAMILY | ebi-a-GCST90016924 | family Acidaminococcaceae id.2167 | 2021 | 33462485 | European | 18340 | 5547067 |
|  | ebi-a-GCST90016925 | family Actinomycetaceae id.422 | 2021 | 33462485 | European | 18340 | 5424030 |
|  | ebi-a-GCST90016926 | family Alcaligenaceae id.2876 | 2021 | 33462485 | European | 18340 | 5696994 |
|  | ebi-a-GCST90016927 | family Bacteroidaceae id.918 | 2021 | 33462485 | European | 18340 | 5729148 |
|  | ebi-a-GCST90016928 | family Bacteroidales S24 7group id.11174 | 2021 | 33462485 | European | 18340 | 5432872 |
|  | ebi-a-GCST90016929 | family Bifidobacteriaceae id.434 | 2021 | 33462485 | European | 18340 | 5668532 |
|  | ebi-a-GCST90016930 | family Christensenellaceae id.1867 | 2021 | 33462485 | European | 18340 | 1515865 |
|  | ebi-a-GCST90016931 | family Clostridiaceae1 id.1870 | 2021 | 33462485 | European | 18340 | 5598586 |
|  | ebi-a-GCST90016932 | family Clostridiales vadin BB60 group id.11287 | 2021 | 33462485 | European | 18340 | 5497800 |
|  | ebi-a-GCST90016933 | family Coriobacteriaceae id.812 | 2021 | 33462485 | European | 18340 | 5698092 |
|  | ebi-a-GCST90016934 | family Defluviitaleaceae id.1925 | 2021 | 33462485 | European | 18340 | 5440708 |
|  | ebi-a-GCST90016935 | family Desulfovibrionaceae id.3170 | 2021 | 33462485 | European | 18340 | 5616478 |
|  | ebi-a-GCST90016936 | family Enterobacteriaceae id.3470 | 2021 | 33462485 | European | 18340 | 5598485 |
|  | ebi-a-GCST90016937 | family Erysipelotrichaceae id.2150 | 2021 | 33462485 | European | 18340 | 5720820 |
|  | ebi-a-GCST90016938 | family Family XI id.1937 | 2021 | 33462485 | European | 18340 | 4330602 |
|  | ebi-a-GCST90016939 | family Family XIII id.1958 | 2021 | 33462485 | European | 18340 | 5620554 |
|  | ebi-a-GCST90016940 | family Lachnospiraceae id.1988 | 2021 | 33462485 | European | 18340 | 5729268 |
|  | ebi-a-GCST90016941 | family Lactobacillaceae id.1837 | 2021 | 33462485 | European | 18340 | 5401891 |
|  | ebi-a-GCST90016942 | family Methanobacteriaceae id.122 | 2021 | 33462485 | European | 18340 | 5118613 |
|  | ebi-a-GCST90016943 | family Oxalobacteraceae id.2967 | 2021 | 33462485 | European | 18340 | 5323881 |
|  | ebi-a-GCST90016944 | family Pasteurellaceae id.3690 | 2021 | 33462485 | European | 18340 | 5491834 |
|  | ebi-a-GCST90016945 | family Peptococcaceae id.2025 | 2021 | 33462485 | European | 18340 | 5490085 |
|  | ebi-a-GCST90016946 | family Peptostreptococcaceae id.2043 | 2021 | 33462485 | European | 18340 | 5643812 |
|  | ebi-a-GCST90016947 | family Porphyromonadaceae id.944 | 2021 | 33462485 | European | 18340 | 5716664 |
|  | ebi-a-GCST90016948 | family Prevotellaceae id.961 | 2021 | 33462485 | European | 18340 | 5643858 |
|  | ebi-a-GCST90016949 | family Rhodospirillaceae id.2718 | 2021 | 33462485 | European | 18340 | 5444973 |
|  | ebi-a-GCST90016950 | family Rikenellaceae id.968 | 2021 | 33462485 | European | 18340 | 5665279 |
|  | ebi-a-GCST90016951 | family Ruminococcaceae id.2051 | 2021 | 33462485 | European | 18340 | 5729189 |
|  | ebi-a-GCST90016952 | family Streptococcaceae id.1851 | 2021 | 33462485 | European | 18340 | 5648128 |
|  | ebi-a-GCST90016956 | family Veillonellaceae id.2173 | 2021 | 33462485 | European | 18340 | 5688519 |
|  | ebi-a-GCST90016957 | family Verrucomicrobiaceae id.4037 | 2021 | 33462485 | European | 18340 | 5518011 |
|  | ebi-a-GCST90016958 | family Victivallaceae id.2256 | 2021 | 33462485 | European | 18340 | 5184073 |
|  | ebi-a-GCST90016953 | unknown family id.1000001215 | 2021 | 33462485 | European | 18340 | 5355169 |
|  | ebi-a-GCST90016954 | unknown family id.1000005472 | 2021 | 33462485 | European | 18340 | 5488779 |
|  | ebi-a-GCST90016955 | unknown family id.1000006162 | 2021 | 33462485 | European | 18340 | 5316331 |
| CLASS | ebi-a-GCST90016908 | class Actinobacteria id.420 | 2021 | 33462485 | European | 18340 | 5684804 |
|  | ebi-a-GCST90016909 | class Alphaproteobacteria id.2380 | 2021 | 33462485 | European | 18340 | 5470627 |
|  | ebi-a-GCST90016910 | class Bacilli id.1674 | 2021 | 33462485 | European | 18340 | 5663902 |
|  | ebi-a-GCST90016911 | class Bacteroidia id.913 | 2021 | 33462485 | European | 18340 | 5729267 |
|  | ebi-a-GCST90016912 | class Betaproteobacteria id.2868 | 2021 | 33462485 | European | 18340 | 5707741 |
|  | ebi-a-GCST90016913 | class Clostridia id.1860 | 2021 | 33462485 | European | 18340 | 5729268 |
|  | ebi-a-GCST90016914 | class Coriobacteriia id.810 | 2021 | 33462485 | European | 18340 | 5698092 |
|  | ebi-a-GCST90016915 | class Deltaproteobacteria id.3088 | 2021 | 33462485 | European | 18340 | 5616854 |
|  | ebi-a-GCST90016916 | class Erysipelotrichia id.2148 | 2021 | 33462485 | European | 18340 | 5720820 |
|  | ebi-a-GCST90016917 | class Gammaproteobacteria id.3304 | 2021 | 33462485 | European | 18340 | 5689849 |
|  | ebi-a-GCST90016918 | class Lentisphaeria id.2251 | 2021 | 33462485 | European | 18340 | 5282234 |
|  | ebi-a-GCST90016919 | class Melainabacteria id.1590 | 2021 | 33462485 | European | 18340 | 5355624 |
|  | ebi-a-GCST90016920 | class Methanobacteria id.120 | 2021 | 33462485 | European | 18340 | 5118613 |
|  | ebi-a-GCST90016921 | class Mollicutes id.3921 | 2021 | 33462485 | European | 18340 | 5510407 |
|  | ebi-a-GCST90016922 | class Negativicutes id.2165 | 2021 | 33462485 | European | 18340 | 5721008 |
|  | ebi-a-GCST90016923 | class Verrucomicrobiae id.4030 | 2021 | 33462485 | European | 18340 | 5518011 |
| GENUS | ebi-a-GCST90016959 | genus Actinomyces id.424 | 2021 | 33462485 | European | 18340 | 5417207 |
|  | ebi-a-GCST90016960 | genus Adlercreutzia id.813 | 2021 | 33462485 | European | 18340 | 5437332 |
|  | ebi-a-GCST90016961 | genus Akkermansia id.4038 | 2021 | 33462485 | European | 18340 | 5518001 |
|  | ebi-a-GCST90016962 | genus Alistipes id.969 | 2021 | 33462485 | European | 18340 | 5662599 |
|  | ebi-a-GCST90016963 | genus Allisonella id.2175 | 2021 | 33462485 | European | 18340 | 4116508 |
|  | ebi-a-GCST90016964 | genus Alloprevotella id.962 | 2021 | 33462485 | European | 18340 | 3047413 |
|  | ebi-a-GCST90016965 | genus Anaerofilum id.2054 | 2021 | 33462485 | European | 18340 | 5321133 |
|  | ebi-a-GCST90016966 | genus Anaerostipes id.1992 | 2021 | 33462485 | European | 18340 | 5691265 |
|  | ebi-a-GCST90016967 | genus Anaerotruncus id.2055 | 2021 | 33462485 | European | 18340 | 5646976 |
|  | ebi-a-GCST90016968 | genus Bacteroides id.919 | 2021 | 33462485 | European | 18340 | 5729148 |
|  | ebi-a-GCST90016969 | genus Barnesiella id.945 | 2021 | 33462485 | European | 18340 | 5561375 |
|  | ebi-a-GCST90016970 | genus Bifidobacterium id.437 | 2021 | 33462485 | European | 18340 | 5666549 |
|  | ebi-a-GCST90016971 | genus Bilophila id.3171 | 2021 | 33462485 | European | 18340 | 5552365 |
|  | ebi-a-GCST90016972 | genus Blautia id.1993 | 2021 | 33462485 | European | 18340 | 335714 |
|  | ebi-a-GCST90016973 | genus Butyricicoccus id.2056 | 2021 | 33462485 | European | 18340 | 5648432 |
|  | ebi-a-GCST90016974 | genus Butyricimonas id.946 | 2021 | 33462485 | European | 18340 | 5487648 |
|  | ebi-a-GCST90016975 | genus Butyrivibrio id.1994 | 2021 | 33462485 | European | 18340 | 5096306 |
|  | ebi-a-GCST90016976 | genus Candidatus Soleaferrea id.11351 | 2021 | 33462485 | European | 18340 | 5372145 |
|  | ebi-a-GCST90016977 | genus Catenibacterium id.2154 | 2021 | 33462485 | European | 18340 | 4089327 |
|  | ebi-a-GCST90016978 | genus Christensenellaceae R 7group id.11284 | 2021 | 33462485 | European | 18340 | 5645310 |
|  | ebi-a-GCST90016979 | genus Clostridium innocuum group id.14398 | 2021 | 33462485 | European | 18340 | 5231958 |
|  | ebi-a-GCST90016980 | genus Clostridium sensustricto1 id.1874 | 2021 | 33462485 | European | 18340 | 5594934 |
|  | ebi-a-GCST90016981 | genus Collinsella id.816 | 2021 | 33462485 | European | 18340 | 5563963 |
|  | ebi-a-GCST90016982 | genus Coprobacter id.950 | 2021 | 33462485 | European | 18340 | 5394658 |
|  | ebi-a-GCST90016983 | genus Coprococcus1 id.11302 | 2021 | 33462485 | European | 18340 | 5649452 |
|  | ebi-a-GCST90016984 | genus Coprococcus2 id.11303 | 2021 | 33462485 | European | 18340 | 5525173 |
|  | ebi-a-GCST90016985 | genus Coprococcus3 id.11304 | 2021 | 33462485 | European | 18340 | 5637737 |
|  | ebi-a-GCST90016986 | genus Defluviitaleaceae UCG011 id.11288 | 2021 | 33462485 | European | 18340 | 5440169 |
|  | ebi-a-GCST90016987 | genus Desulfovibrio id.3174 | 2021 | 33462485 | European | 18340 | 5459163 |
|  | ebi-a-GCST90016988 | genus Dialister id.2184 | 2021 | 33462485 | European | 18340 | 5553148 |
|  | ebi-a-GCST90016989 | genus Dorea id.1998 | 2021 | 33462485 | European | 18340 | 5702812 |
|  | ebi-a-GCST90016990 | genus Eggerthella id.820 | 2021 | 33462485 | European | 18340 | 5317951 |
|  | ebi-a-GCST90016991 | genus Eisenbergiella id.11305 | 2021 | 33462485 | European | 18340 | 5348517 |
|  | ebi-a-GCST90016992 | genus Enterorhabdus id.821 | 2021 | 33462485 | European | 18340 | 5416385 |
|  | ebi-a-GCST90016993 | genus Erysipelatoclostridium id.11382 | 2021 | 33462485 | European | 18340 | 5478224 |
|  | ebi-a-GCST90016994 | genus Erysipelotrichaceae UCG003 id.11385 | 2021 | 33462485 | European | 18340 | 563252 |
|  | ebi-a-GCST90016995 | genus Escherichia Shigella id.3505 | 2021 | 33462485 | European | 18340 | 5537082 |
|  | ebi-a-GCST90016996 | genus Eubacterium brachy group id.11297 | 2021 | 33462485 | European | 18340 | 5221253 |
|  | ebi-a-GCST90016997 | genus Eubacterium coprostanoligenes group id.11376 | 2021 | 33462485 | European | 18340 | 5699448 |
|  | ebi-a-GCST90016998 | genus Eubacterium eligens group id.14373 | 2021 | 33462485 | European | 18340 | 5631614 |
|  | ebi-a-GCST90016999 | genus Eubacterium fissicatena group id.14374 | 2021 | 33462485 | European | 18340 | 5159588 |
|  | ebi-a-GCST90017000 | genus Eubacterium hallii group id.11339 | 2021 | 33462485 | European | 18340 | 5686593 |
|  | ebi-a-GCST90017001 | genus Eubacterium nodatum group id.11298 | 2021 | 33462485 | European | 18340 | 5050598 |
|  | ebi-a-GCST90017002 | genus Eubacterium oxidoreducens group id.11340 | 2021 | 33462485 | European | 18340 | 5324438 |
|  | ebi-a-GCST90017003 | genus Eubacterium rectale group id.14375 | 2021 | 33462485 | European | 18340 | 5708381 |
|  | ebi-a-GCST90017004 | genus Eubacterium ruminantium group id.11341 | 2021 | 33462485 | European | 18340 | 5427661 |
|  | ebi-a-GCST90017005 | genus Eubacterium ventriosum group id.11342 | 2021 | 33462485 | European | 18340 | 5603754 |
|  | ebi-a-GCST90017006 | genus Eubacterium xylanophilum group id.14376 | 2021 | 33462485 | European | 18340 | 5543813 |
|  | ebi-a-GCST90017007 | genus Faecalibacterium id.2058 | 2021 | 33462485 | European | 18340 | 5720140 |
|  | ebi-a-GCST90017008 | genus Family XIII AD3011 group id.11294 | 2021 | 33462485 | European | 18340 | 5577002 |
|  | ebi-a-GCST90017009 | genus Family XIII UCG001 id.11295 | 2021 | 33462485 | European | 18340 | 5521084 |
|  | ebi-a-GCST90017010 | genus Flavonifractor id.2060 | 2021 | 33462485 | European | 18340 | 5510045 |
|  | ebi-a-GCST90017011 | genus Fusicatenibacter id.11306 | 2021 | 33462485 | European | 18340 | 5697232 |
|  | ebi-a-GCST90017012 | genus Gordonibacter id.822 | 2021 | 33462485 | European | 18340 | 5165457 |
|  | ebi-a-GCST90017013 | genus Haemophilus id.3699 | 2021 | 33462485 | European | 18340 | 5481944 |
|  | ebi-a-GCST90017014 | genus Holdemanella id.11394 | 2021 | 33462485 | European | 18340 | 5421763 |
|  | ebi-a-GCST90017015 | genus Holdemania id.2158 | 2021 | 33462485 | European | 18340 | 5464928 |
|  | ebi-a-GCST90017016 | genus Howardella id.2001 | 2021 | 33462485 | European | 18340 | 5174541 |
|  | ebi-a-GCST90017017 | genus Hungatella id.11307 | 2021 | 33462485 | European | 18340 | 5211608 |
|  | ebi-a-GCST90017018 | genus Intestinibacter id.11346 | 2021 | 33462485 | European | 18340 | 5535009 |
|  | ebi-a-GCST90017019 | genus Intestinimonas id.2063 | 2021 | 33462485 | European | 18340 | 5540147 |
|  | ebi-a-GCST90017020 | genus Lachnoclostridium id.11309 | 2021 | 33462485 | European | 18340 | 5714724 |
|  | ebi-a-GCST90017029 | genus Lachnospira id.2005 | 2021 | 33462485 | European | 18340 | 462269 |
|  | ebi-a-GCST90017021 | genus Lachnospiraceae FCS020 group id.11315 | 2021 | 33462485 | European | 18340 | 5547835 |
|  | ebi-a-GCST90017022 | genus Lachnospiraceae NC2004 group id.11317 | 2021 | 33462485 | European | 18340 | 5369940 |
|  | ebi-a-GCST90017023 | genus Lachnospiraceae ND3007 group id.11318 | 2021 | 33462485 | European | 18340 | 5629458 |
|  | ebi-a-GCST90017024 | genus Lachnospiraceae NK4A136 group id.11320 | 2021 | 33462485 | European | 18340 | 5663428 |
|  | ebi-a-GCST90017025 | genus Lachnospiraceae UCG001 id.11322 | 2021 | 33462485 | European | 18340 | 5482153 |
|  | ebi-a-GCST90017026 | genus Lachnospiraceae UCG004 id.11325 | 2021 | 33462485 | European | 18340 | 5633244 |
|  | ebi-a-GCST90017027 | genus Lachnospiraceae UCG008 id.11329 | 2021 | 33462485 | European | 18340 | 5401028 |
|  | ebi-a-GCST90017028 | genus Lachnospiraceae UCG010 id.11331 | 2021 | 33462485 | European | 18340 | 5565113 |
|  | ebi-a-GCST90017030 | genus Lactobacillus id.1838 | 2021 | 33462485 | European | 18340 | 5398287 |
|  | ebi-a-GCST90017031 | genus Lactococcus id.1852 | 2021 | 33462485 | European | 18340 | 5200746 |
|  | ebi-a-GCST90017032 | genus Marvinbryantia id.2006 | 2021 | 33462485 | European | 18340 | 5507087 |
|  | ebi-a-GCST90017033 | genus Methanobrevibacter id.124 | 2021 | 33462485 | European | 18340 | 5092705 |
|  | ebi-a-GCST90017034 | genus Odoribacter id.953 | 2021 | 33462485 | European | 18340 | 5584164 |
|  | ebi-a-GCST90017035 | genus Olsenella id.823 | 2021 | 33462485 | European | 18340 | 5215565 |
|  | ebi-a-GCST90017036 | genus Oscillibacter id.2064 | 2021 | 33462485 | European | 18340 | 5450090 |
|  | ebi-a-GCST90017037 | genus Oscillospira id.2065 | 2021 | 33462485 | European | 18340 | 5487907 |
|  | ebi-a-GCST90017038 | genus Oxalobacter id.2979 | 2021 | 33462485 | European | 18340 | 5290208 |
|  | ebi-a-GCST90017039 | genus Parabacteroides id.955 | 2021 | 33462485 | European | 18340 | 5709019 |
|  | ebi-a-GCST90017040 | genus Paraprevotella id.963 | 2021 | 33462485 | European | 18340 | 5407469 |
|  | ebi-a-GCST90017041 | genus Parasutterella id.2893 | 2021 | 33462485 | European | 18340 | 5512811 |
|  | ebi-a-GCST90017042 | genus Peptococcus id.2038 | 2021 | 33462485 | European | 18340 | 5350051 |
|  | ebi-a-GCST90017043 | genus Phascolarctobacterium id.2169 | 2021 | 33462485 | European | 18340 | 5509695 |
|  | ebi-a-GCST90017044 | genus Prevotella7 id.11183 | 2021 | 33462485 | European | 18340 | 5089245 |
|  | ebi-a-GCST90017045 | genus Prevotella9 id.11184 | 2021 | 33462485 | European | 18340 | 5535200 |
|  | ebi-a-GCST90017046 | genus Rikenellaceae RC9 gut group id.11192 | 2021 | 33462485 | European | 18340 | 4945903 |
|  | ebi-a-GCST90017047 | genus Romboutsia id.11348 | 2021 | 33462485 | European | 18340 | 5594534 |
|  | ebi-a-GCST90017048 | genus Roseburia id.2013 | 2021 | 33462485 | European | 18340 | 5712148 |
|  | ebi-a-GCST90017049 | genus Ruminiclostridium5 id.11356 | 2021 | 33462485 | European | 18340 | 5653350 |
|  | ebi-a-GCST90017050 | genus Ruminiclostridium6 id.11357 | 2021 | 33462485 | European | 18340 | 5557669 |
|  | ebi-a-GCST90017051 | genus Ruminiclostridium9 id.11358 | 2021 | 33462485 | European | 18340 | 5649657 |
|  | ebi-a-GCST90017052 | genus Ruminococcaceae NK4A214 group id.11359 | 2021 | 33462485 | European | 18340 | 5616365 |
|  | ebi-a-GCST90017053 | genus Ruminococcaceae UCG002 id.11361 | 2021 | 33462485 | European | 18340 | 5694947 |
|  | ebi-a-GCST90017054 | genus Ruminococcaceae UCG003 id.11362 | 2021 | 33462485 | European | 18340 | 5624730 |
|  | ebi-a-GCST90017055 | genus Ruminococcaceae UCG004 id.11363 | 2021 | 33462485 | European | 18340 | 5478416 |
|  | ebi-a-GCST90017056 | genus Ruminococcaceae UCG005 id.11364 | 2021 | 33462485 | European | 18340 | 5638483 |
|  | ebi-a-GCST90017057 | genus Ruminococcaceae UCG009 id.11367 | 2021 | 33462485 | European | 18340 | 5418432 |
|  | ebi-a-GCST90017058 | genus Ruminococcaceae UCG010 id.11368 | 2021 | 33462485 | European | 18340 | 5545122 |
|  | ebi-a-GCST90017059 | genus Ruminococcaceae UCG011 id.11369 | 2021 | 33462485 | European | 18340 | 5176480 |
|  | ebi-a-GCST90017060 | genus Ruminococcaceae UCG013 id.11371 | 2021 | 33462485 | European | 18340 | 5642029 |
|  | ebi-a-GCST90017061 | genus Ruminococcaceae UCG014 id.11372 | 2021 | 33462485 | European | 18340 | 5582406 |
|  | ebi-a-GCST90017064 | genus Ruminococcus gauvreauii group id.11343 | 2021 | 33462485 | European | 18340 | 5572328 |
|  | ebi-a-GCST90017065 | genus Ruminococcus gnavus group id.14377 | 2021 | 33462485 | European | 18340 | 5381061 |
|  | ebi-a-GCST90017066 | genus Ruminococcus torques group id.14378 | 2021 | 33462485 | European | 18340 | 5709224 |
|  | ebi-a-GCST90017062 | genus Ruminococcus1 id.11374 | 2021 | 33462485 | European | 18340 | 5653204 |
|  | ebi-a-GCST90017063 | genus Ruminococcus2 id.11375 | 2021 | 33462485 | European | 18340 | 5619774 |
|  | ebi-a-GCST90017067 | genus Sellimonas id.14370 | 2021 | 33462485 | European | 18340 | 4950440 |
|  | ebi-a-GCST90017068 | genus Senegalimassilia id.11161 | 2021 | 33462485 | European | 18340 | 5398286 |
|  | ebi-a-GCST90017069 | genus Slackia id.826 | 2021 | 33462485 | European | 18340 | 5369110 |
|  | ebi-a-GCST90017070 | genus Streptococcus id.1854 | 2021 | 33462485 | European | 18340 | 5643866 |
|  | ebi-a-GCST90017071 | genus Subdoligranulum id.2071 | 2021 | 33462485 | European | 18340 | 5708796 |
|  | ebi-a-GCST90017072 | genus Sutterella id.2897 | 2021 | 33462485 | European | 18340 | 5586151 |
|  | ebi-a-GCST90017073 | genus Terrisporobacter id.11349 | 2021 | 33462485 | European | 18340 | 5391104 |
|  | ebi-a-GCST90017074 | genus Turicibacter id.2163 | 2021 | 33462485 | European | 18340 | 5459137 |
|  | ebi-a-GCST90017075 | genus Tyzzerella3 id.11336 | 2021 | 33462485 | European | 18340 | 5326934 |
|  | ebi-a-GCST90017088 | genus Veillonella id.2199 | 2021 | 33462485 | European | 18340 | 5486191 |
|  | ebi-a-GCST90017089 | genus Victivallis id.2257 | 2021 | 33462485 | Mixed | 18340 | 4291501 |
|  | ebi-a-GCST90017076 | unknown genus id.1000000074 | 2021 | 33462485 | European | 18340 | 5497800 |
|  | ebi-a-GCST90017077 | unknown genus id.1000001216 | 2021 | 33462485 | European | 18340 | 5355169 |
|  | ebi-a-GCST90017078 | unknown genus id.1000005473 | 2021 | 33462485 | European | 18340 | 5488779 |
|  | ebi-a-GCST90017079 | unknown genus id.1000005480 | 2021 | 33462485 | European | 18340 | 5432872 |
|  | ebi-a-GCST90017080 | unknown genus id.1000006163 | 2021 | 33462485 | European | 18340 | 5316331 |
|  | ebi-a-GCST90017081 | unknown genus id.1869 | 2021 | 33462485 | European | 18340 | 5493572 |
|  | ebi-a-GCST90017082 | unknown genus id.2002 | 2021 | 33462485 | European | 18340 | 5485285 |
|  | ebi-a-GCST90017083 | unknown genus id.2042 | 2021 | 33462485 | European | 18340 | 5431786 |
|  | ebi-a-GCST90017084 | unknown genus id.2072 | 2021 | 33462485 | European | 18340 | 5546384 |
|  | ebi-a-GCST90017085 | unknown genus id.2756 | 2021 | 33462485 | European | 18340 | 5443849 |
|  | ebi-a-GCST90017086 | unknown genus id.827 | 2021 | 33462485 | European | 18340 | 5529970 |
|  | ebi-a-GCST90017087 | unknown genus id.960 | 2021 | 33462485 | European | 18340 | 5279880 |
| ORDER | ebi-a-GCST90017090 | order Actinomycetales id.421 | 2021 | 33462485 | European | 18340 | 5424038 |
|  | ebi-a-GCST90017091 | order Bacillales id.1675 | 2021 | 33462485 | European | 18340 | 4738158 |
|  | ebi-a-GCST90017092 | order Bacteroidales id.914 | 2021 | 33462485 | European | 18340 | 5729267 |
|  | ebi-a-GCST90017093 | order Bifidobacteriales id.433 | 2021 | 33462485 | European | 18340 | 5668532 |
|  | ebi-a-GCST90017094 | order Burkholderiales id.2875 | 2021 | 33462485 | European | 18340 | 5707170 |
|  | ebi-a-GCST90017095 | order Clostridiales id.1864 | 2021 | 33462485 | European | 18340 | 5729268 |
|  | ebi-a-GCST90017096 | order Coriobacteriales id.811 | 2021 | 33462485 | European | 18340 | 5698092 |
|  | ebi-a-GCST90017097 | order Desulfovibrionales id.3157 | 2021 | 33462485 | European | 18340 | 5616504 |
|  | ebi-a-GCST90017098 | order Enterobacteriales id.3469 | 2021 | 33462485 | European | 18340 | 5598485 |
|  | ebi-a-GCST90017099 | order Erysipelotrichales id.2149 | 2021 | 33462485 | European | 18340 | 5720820 |
|  | ebi-a-GCST90017100 | order Gastranaerophilales id.1592 | 2021 | 33462485 | European | 18340 | 5355169 |
|  | ebi-a-GCST90017101 | order Lactobacillales id.1801 | 2021 | 33462485 | European | 18340 | 5662756 |
|  | ebi-a-GCST90017102 | order Methanobacteriales id.121 | 2021 | 33462485 | European | 18340 | 5118613 |
|  | ebi-a-GCST90017103 | order Mollicutes RF9 id.11580 | 2021 | 33462485 | European | 18340 | 5488779 |
|  | ebi-a-GCST90017104 | order NB1n id.3954 | 2021 | 33462485 | European | 18340 | 5316331 |
|  | ebi-a-GCST90017105 | order Pasteurellales id.3689 | 2021 | 33462485 | European | 18340 | 5491834 |
|  | ebi-a-GCST90017106 | order Rhodospirillales id.2668 | 2021 | 33462485 | European | 18340 | 5447392 |
|  | ebi-a-GCST90017107 | order Selenomonadales id.2166 | 2021 | 33462485 | European | 18340 | 5721008 |
|  | ebi-a-GCST90017108 | order Verrucomicrobiales id.4031 | 2021 | 33462485 | European | 18340 | 5518011 |
|  | ebi-a-GCST90017109 | order Victivallales id.2255 | 2021 | 33462485 | European | 18340 | 5282234 |
| PHYLUM | ebi-a-GCST90017110 | phylum Actinobacteria id.401 | 2021 | 33462485 | European | 18340 | 5706452 |
|  | ebi-a-GCST90017111 | phylum Bacteroidetes id.906 | 2021 | 33462485 | European | 18340 | 5729267 |
|  | ebi-a-GCST90017112 | phylum Cyanobacteria id.1501 | 2021 | 33462485 | European | 18340 | 5403633 |
|  | ebi-a-GCST90017113 | phylum Euryarchaeota id.56 | 2021 | 33462485 | European | 18340 | 5165248 |
|  | ebi-a-GCST90017114 | phylum Firmicutes id.1673 | 2021 | 33462485 | European | 18340 | 5729268 |
|  | ebi-a-GCST90017115 | phylum Lentisphaerae id.2239 | 2021 | 33462485 | European | 18340 | 5282630 |
|  | ebi-a-GCST90017116 | phylum Proteobacteria id.2376 | 2021 | 33462485 | European | 18340 | 5728442 |
|  | ebi-a-GCST90017117 | phylum Tenericutes id.3920 | 2021 | 33462485 | European | 18340 | 5510407 |
|  | ebi-a-GCST90017118 | phylum Verrucomicrobia id.3983 | 2021 | 33462485 | European | 18340 | 5530329 |

| Supplementary Table 2: Summary of GWAS data about urolithiasis. | | | | | | | | | | | | | |
| --- | --- | --- | --- | --- | --- | --- | --- | --- | --- | --- | --- | --- | --- |
| **id** | **trait** | **year** | **author** | **sex** | **pmid** | **population** | **unit** | **sample_size** | **nsnp** | **build** | **category** | **ncase** | **ncontrol** |
| bbj-a-155 | Urolithiasis | 2019 | Ishigaki K | Males and Females | NA | East Asian | NA | 212453 | 8885805 | HG19/GRCh37 | Binary | 6638 | 205815 |
| ukb-b-18372 | Diagnoses - main ICD10: N20.0 Calculus of kidney | 2018 | Ben Elsworth | Males and Females | NA | European | SD | 463010 | 9851867 | HG19/GRCh37 | Binary | 2186 | 460824 |
| ukb-b-7205 | Operative procedures - main OPCS: M14.1 Extracorporeal shock wave lithotripsy of calculus of kidney | 2018 | Ben Elsworth | Males and Females | NA | European | SD | 463010 | 9851867 | HG19/GRCh37 | Binary | 1296 | 461714 |
| ukb-b-8297 | Non-cancer illness code, self-reported: kidney stone/ureter stone/bladder stone | 2018 | Ben Elsworth | Males and Females | NA | European | SD | 462933 | 9851867 | HG19/GRCh37 | Binary | 3625 | 459308 |
| ukb-b-13537 | Operation code: percutaneous/open kidney stone surgery/lithotripsy | 2018 | Ben Elsworth | Males and Females | NA | European | SD | 462933 | 9851867 | HG19/GRCh37 | Binary | 2645 | 460288 |
| ukb-a-574 | Diagnoses - main ICD10: N20 Calculus of kidney and ureter | 2017 | Neale | Males and Females | NA | European | SD | 337199 | 10894596 | HG19/GRCh37 | NA | 2427 | 334772 |
| ukb-a-72 | Non-cancer illness code self-reported: kidney stone/ureter stone/bladder stone | 2017 | Neale | Males and Females | NA | European | SD | 337159 | 10894596 | HG19/GRCh37 | NA | 2694 | 334465 |
| finn-b-N14_CALCUKIDUR | Calculus of kidney and ureter | 2021 | NA | Males and Females | NA | European | NA | 218414 | 16380464 | HG19/GRCh37 | Binary | 4969 | 213445 |
| finn-b-N14_UROLITHIASIS | Urolithiasis | 2021 | NA | Males and Females | NA | European | NA | 218414 | 16380466 | HG19/GRCh37 | Binary | 5347 | 213445 |

| Supplementary Table 3: All IVs used for MR analysis | | | | | | | | | | | | | | |  |
| --- | --- | --- | --- | --- | --- | --- | --- | --- | --- | --- | --- | --- | --- | --- | --- |
| id | genus | SNP | effect_allele | other_allele | eaf | exposure | | | outcome | | | R² | | F |  |
|  |  |  |  |  |  | beta | se | pval | beta | se | pval |  |  |  |  |
| ebi-a-GCST90016959 | Gut microbiota abundance (genus Actinomyces id.423) | rs2715439 | T | C | 0.528 | -0.075 | 0.016 | 6.27E-06 | 0.017 | 0.021 | 0.425 | 0.003 | | 51.098 |  |
|  |  | rs34583783 | G | T | 0.061 | 0.127 | 0.027 | 4.49E-06 | -0.032 | 0.044 | 0.471 | 0.002 | | 33.564 |  |
|  |  | rs35011108 | A | G | 0.068 | 0.233 | 0.051 | 6.34E-06 | -0.038 | 0.043 | 0.371 | 0.007 | | 126.226 |  |
|  |  | rs4073240 | G | A | 0.381 | 0.075 | 0.017 | 7.94E-06 | 0.011 | 0.022 | 0.617 | 0.003 | | 48.728 |  |
|  |  | rs4146653 | G | A | 0.142 | 0.099 | 0.021 | 4.50E-06 | 0.034 | 0.030 | 0.267 | 0.002 | | 43.400 |  |
|  |  | rs71315246 | A | G | 0.136 | -0.097 | 0.022 | 9.83E-06 | -0.009 | 0.031 | 0.767 | 0.002 | | 40.496 |  |
|  |  | rs7915461 | C | T | 0.931 | -0.188 | 0.040 | 5.92E-06 | -0.096 | 0.042 | 0.024 | 0.005 | | 83.212 |  |
| ebi-a-GCST90016960 | Gut microbiota abundance (genus Adlercreutzia id.812) | rs11604400 | C | T | 0.105 | -0.103 | 0.023 | 9.74E-06 | -0.047 | 0.034 | 0.174 | 0.002 | | 36.269 |  |
|  |  | rs13231526 | C | A | 0.080 | 0.143 | 0.031 | 4.81E-06 | -0.002 | 0.039 | 0.956 | 0.003 | | 55.588 |  |
|  |  | rs2717140 | C | T | 0.099 | -0.119 | 0.025 | 2.05E-06 | -0.019 | 0.036 | 0.597 | 0.003 | | 46.415 |  |
|  |  | rs55719207 | G | A | 0.402 | -0.070 | 0.016 | 9.61E-06 | 0.012 | 0.022 | 0.597 | 0.002 | | 43.214 |  |
|  |  | rs6664405 | T | C | 0.146 | -0.095 | 0.021 | 5.23E-06 | 0.037 | 0.030 | 0.224 | 0.002 | | 41.515 |  |
|  |  | rs7680684 | T | C | 0.658 | 0.083 | 0.017 | 9.77E-07 | -0.024 | 0.022 | 0.292 | 0.003 | | 57.585 |  |
|  |  | rs9490822 | C | T | 0.451 | -0.073 | 0.016 | 2.54E-06 | 0.013 | 0.021 | 0.531 | 0.003 | | 49.114 |  |
|  |  | rs9915817 | C | T | 0.707 | -0.075 | 0.017 | 8.22E-06 | 0.011 | 0.023 | 0.628 | 0.002 | | 42.746 |  |
| ebi-a-GCST90016961 | Gut microbiota abundance (genus Akkermansia id.4037) | rs111862613 | T | C | 0.178 | 0.091 | 0.020 | 3.39E-06 | 0.013 | 0.029 | 0.662 | 0.002 | | 44.624 |  |
|  |  | rs117107102 | A | G | 0.048 | 0.204 | 0.043 | 3.01E-06 | 0.026 | 0.049 | 0.603 | 0.004 | | 69.748 |  |
|  |  | rs11729256 | T | C | 0.170 | 0.075 | 0.015 | 6.58E-07 | 0.004 | 0.028 | 0.875 | 0.002 | | 29.151 |  |
|  |  | rs12908520 | G | A | 0.426 | 0.062 | 0.013 | 2.26E-06 | 0.017 | 0.021 | 0.429 | 0.002 | | 34.291 |  |
|  |  | rs2602429 | T | C | 0.739 | -0.075 | 0.016 | 2.72E-06 | -0.015 | 0.024 | 0.532 | 0.002 | | 39.423 |  |
|  |  | rs4242783 | A | G | 0.717 | -0.069 | 0.015 | 3.00E-06 | 0.017 | 0.024 | 0.471 | 0.002 | | 35.063 |  |
|  |  | rs4936098 | G | A | 0.651 | -0.065 | 0.014 | 1.10E-06 | 0.005 | 0.022 | 0.832 | 0.002 | | 35.185 |  |
|  |  | rs61779207 | G | A | 0.224 | -0.076 | 0.017 | 6.32E-06 | -0.010 | 0.026 | 0.707 | 0.002 | | 36.891 |  |
|  |  | rs74542928 | T | C | 0.048 | 0.113 | 0.024 | 1.48E-06 | -0.023 | 0.049 | 0.640 | 0.001 | | 21.257 |  |
|  |  | rs9349825 | A | G | 0.190 | -0.070 | 0.015 | 2.60E-06 | 0.044 | 0.027 | 0.103 | 0.002 | | 27.925 |  |
|  |  | rs941682 | G | A | 0.274 | -0.063 | 0.014 | 9.17E-06 | 0.028 | 0.024 | 0.237 | 0.002 | | 29.256 |  |
| ebi-a-GCST90016962 | Gut microbiota abundance (genus Alistipes id.968) | rs1107244 | G | A | 0.078 | 0.076 | 0.017 | 3.59E-06 | -0.006 | 0.039 | 0.878 | 0.001 | | 15.239 |  |
|  |  | rs11769002 | G | A | 0.415 | -0.053 | 0.011 | 1.45E-06 | 0.008 | 0.022 | 0.724 | 0.001 | | 24.938 |  |
|  |  | rs11958296 | A | G | 0.045 | -0.098 | 0.022 | 9.30E-06 | 0.058 | 0.051 | 0.257 | 0.001 | | 15.072 |  |
|  |  | rs12990744 | C | T | 0.103 | -0.078 | 0.017 | 8.21E-06 | 0.033 | 0.035 | 0.345 | 0.001 | | 20.425 |  |
|  |  | rs1689282 | A | C | 0.323 | -0.052 | 0.011 | 5.28E-06 | -0.011 | 0.023 | 0.641 | 0.001 | | 21.706 |  |
|  |  | rs2290844 | C | T | 0.113 | 0.081 | 0.019 | 9.10E-06 | 0.063 | 0.034 | 0.064 | 0.001 | | 24.388 |  |
|  |  | rs2450745 | A | C | 0.069 | -0.081 | 0.018 | 7.12E-06 | -0.004 | 0.042 | 0.928 | 0.001 | | 15.324 |  |
|  |  | rs2875322 | C | T | 0.830 | 0.058 | 0.013 | 8.78E-06 | -0.015 | 0.028 | 0.601 | 0.001 | | 17.523 |  |
|  |  | rs34417064 | A | G | 0.450 | -0.048 | 0.011 | 7.01E-06 | -0.003 | 0.021 | 0.897 | 0.001 | | 21.113 |  |
|  |  | rs4810359 | A | G | 0.123 | -0.065 | 0.015 | 7.50E-06 | 0.006 | 0.032 | 0.853 | 0.001 | | 16.797 |  |
|  |  | rs62576416 | T | C | 0.383 | 0.049 | 0.011 | 7.50E-06 | 0.004 | 0.022 | 0.854 | 0.001 | | 21.061 |  |
|  |  | rs7129639 | A | C | 0.668 | 0.052 | 0.011 | 1.78E-06 | -0.007 | 0.023 | 0.769 | 0.001 | | 22.449 |  |
|  |  | rs8130320 | A | G | 0.560 | -0.049 | 0.011 | 4.84E-06 | -0.024 | 0.021 | 0.255 | 0.001 | | 21.728 |  |
| ebi-a-GCST90016963 | Gut microbiota abundance (genus Allisonella id.2174) | rs1901739 | G | T | 0.463 | -0.116 | 0.025 | 3.59E-06 | -0.022 | 0.021 | 0.303 | 0.007 | | 123.031 |  |
|  |  | rs35110698 | T | C | 0.147 | -0.146 | 0.032 | 5.72E-06 | -0.038 | 0.030 | 0.206 | 0.005 | | 98.713 |  |
|  |  | rs35778461 | C | T | 0.228 | 0.147 | 0.030 | 1.21E-06 | 0.027 | 0.025 | 0.291 | 0.008 | | 139.862 |  |
|  |  | rs594561 | T | C | 0.489 | -0.112 | 0.025 | 9.41E-06 | 0.016 | 0.021 | 0.457 | 0.006 | | 116.168 |  |
|  |  | rs602075 | G | A | 0.737 | -0.169 | 0.030 | 3.57E-08 | -0.008 | 0.024 | 0.730 | 0.011 | | 205.451 |  |
|  |  | rs6742198 | A | G | 0.758 | -0.149 | 0.032 | 3.35E-06 | 0.063 | 0.025 | 0.011 | 0.008 | | 150.812 |  |
|  |  | rs76904847 | G | A | 0.176 | 0.149 | 0.033 | 6.09E-06 | 0.002 | 0.028 | 0.951 | 0.006 | | 118.245 |  |
|  |  | rs7898615 | T | G | 0.137 | 0.168 | 0.037 | 8.87E-06 | 0.016 | 0.031 | 0.615 | 0.007 | | 123.082 |  |
| ebi-a-GCST90016964 | Gut microbiota abundance (genus Alloprevotella id.961) | rs12675596 | G | T | 0.215 | 0.146 | 0.029 | 9.64E-07 | 0.013 | 0.026 | 0.605 | 0.007 | | 132.341 |  |
|  |  | rs2154444 | G | T | 0.735 | -0.138 | 0.031 | 8.37E-06 | -0.004 | 0.024 | 0.879 | 0.007 | | 137.427 |  |
|  |  | rs34619204 | G | A | 0.174 | -0.156 | 0.034 | 8.84E-06 | 0.006 | 0.028 | 0.833 | 0.007 | | 128.950 |  |
|  |  | rs4364940 | A | G | 0.306 | 0.126 | 0.028 | 8.58E-06 | -0.008 | 0.023 | 0.720 | 0.007 | | 125.210 |  |
|  |  | rs4680035 | G | A | 0.386 | 0.120 | 0.026 | 4.99E-06 | 0.026 | 0.022 | 0.234 | 0.007 | | 125.171 |  |
|  |  | rs58212166 | A | G | 0.184 | -0.162 | 0.036 | 7.94E-06 | 0.024 | 0.027 | 0.377 | 0.008 | | 144.894 |  |
| ebi-a-GCST90016965 | Gut microbiota abundance (genus Anaerofilum id.2053) | rs10794359 | C | T | 0.535 | 0.095 | 0.020 | 2.23E-06 | -0.032 | 0.022 | 0.132 | 0.005 | | 83.338 |  |
|  |  | rs1563175 | A | C | 0.458 | 0.092 | 0.020 | 5.54E-06 | 0.001 | 0.021 | 0.955 | 0.004 | | 78.028 |  |
|  |  | rs17012738 | T | G | 0.490 | 0.090 | 0.020 | 7.24E-06 | 0.007 | 0.021 | 0.731 | 0.004 | | 75.113 |  |
|  |  | rs17096874 | C | T | 0.212 | -0.126 | 0.027 | 2.86E-06 | 0.007 | 0.026 | 0.800 | 0.005 | | 98.146 |  |
|  |  | rs356049 | G | A | 0.069 | 0.133 | 0.029 | 6.56E-06 | -0.066 | 0.043 | 0.119 | 0.002 | | 41.555 |  |
|  |  | rs4244069 | A | G | 0.873 | 0.147 | 0.033 | 9.81E-06 | -0.019 | 0.032 | 0.556 | 0.005 | | 88.020 |  |
|  |  | rs4506496 | A | G | 0.707 | -0.103 | 0.021 | 1.49E-06 | -0.012 | 0.023 | 0.622 | 0.004 | | 81.034 |  |
|  |  | rs712981 | A | C | 0.386 | 0.101 | 0.020 | 6.83E-07 | 0.030 | 0.022 | 0.164 | 0.005 | | 88.651 |  |
|  |  | rs79598899 | C | T | 0.047 | 0.183 | 0.036 | 3.75E-07 | -0.011 | 0.050 | 0.834 | 0.003 | | 55.364 |  |
|  |  | rs816292 | T | C | 0.296 | -0.113 | 0.022 | 2.64E-07 | -0.032 | 0.023 | 0.168 | 0.005 | | 98.078 |  |
|  |  | rs9299345 | T | C | 0.096 | -0.136 | 0.030 | 8.04E-06 | -0.014 | 0.036 | 0.706 | 0.003 | | 59.470 |  |
| ebi-a-GCST90016966 | Gut microbiota abundance (genus Anaerostipes id.1991) | rs10502061 | A | G | 0.114 | 0.084 | 0.019 | 7.94E-06 | 0.005 | 0.033 | 0.872 | 0.001 | | 25.991 |  |
|  |  | rs2014785 | T | C | 0.443 | 0.052 | 0.011 | 4.68E-06 | -0.005 | 0.022 | 0.831 | 0.001 | | 24.094 |  |
|  |  | rs2396460 | C | T | 0.515 | 0.051 | 0.011 | 2.91E-06 | 0.017 | 0.021 | 0.418 | 0.001 | | 24.108 |  |
|  |  | rs2804244 | G | A | 0.618 | 0.053 | 0.011 | 2.04E-06 | -0.008 | 0.022 | 0.709 | 0.001 | | 24.428 |  |
|  |  | rs3900776 | G | A | 0.028 | -0.110 | 0.024 | 2.75E-06 | 0.000 | 0.063 | 0.996 | 0.001 | | 12.215 |  |
|  |  | rs60983350 | G | A | 0.361 | -0.054 | 0.012 | 4.42E-06 | 0.026 | 0.023 | 0.255 | 0.001 | | 24.692 |  |
|  |  | rs62157625 | T | C | 0.125 | 0.089 | 0.019 | 1.45E-06 | -0.013 | 0.032 | 0.699 | 0.002 | | 31.503 |  |
|  |  | rs62215703 | G | A | 0.216 | 0.064 | 0.014 | 1.98E-06 | -0.031 | 0.026 | 0.233 | 0.001 | | 25.907 |  |
|  |  | rs6474958 | G | A | 0.691 | 0.050 | 0.011 | 6.74E-06 | 0.005 | 0.023 | 0.819 | 0.001 | | 19.590 |  |
|  |  | rs6726833 | C | A | 0.079 | -0.088 | 0.019 | 3.32E-06 | -0.041 | 0.039 | 0.293 | 0.001 | | 20.461 |  |
|  |  | rs6854026 | C | T | 0.438 | 0.051 | 0.011 | 3.20E-06 | -0.018 | 0.021 | 0.397 | 0.001 | | 23.377 |  |
|  |  | rs7193624 | T | C | 0.917 | -0.075 | 0.015 | 5.35E-07 | -0.012 | 0.038 | 0.757 | 0.001 | | 15.794 |  |
|  |  | rs78735375 | A | C | 0.040 | -0.137 | 0.031 | 5.33E-06 | -0.070 | 0.054 | 0.196 | 0.001 | | 26.638 |  |
| ebi-a-GCST90016967 | Gut microbiota abundance (genus Anaerotruncus id.2054) | rs10150232 | A | G | 0.200 | 0.057 | 0.012 | 6.68E-06 | -0.011 | 0.026 | 0.673 | 0.001 | | 18.862 |  |
|  |  | rs11018566 | A | G | 0.053 | -0.156 | 0.037 | 6.14E-06 | -0.005 | 0.047 | 0.908 | 0.002 | | 45.531 |  |
|  |  | rs115414803 | A | C | 0.062 | -0.144 | 0.032 | 6.83E-06 | -0.003 | 0.044 | 0.940 | 0.002 | | 44.771 |  |
|  |  | rs1272208 | T | G | 0.762 | 0.061 | 0.013 | 4.28E-06 | 0.074 | 0.025 | 0.003 | 0.001 | | 24.961 |  |
|  |  | rs1431492 | C | T | 0.157 | -0.065 | 0.015 | 7.36E-06 | 0.033 | 0.029 | 0.253 | 0.001 | | 20.881 |  |
|  |  | rs17734739 | T | C | 0.141 | 0.066 | 0.015 | 7.43E-06 | -0.057 | 0.030 | 0.059 | 0.001 | | 19.362 |  |
|  |  | rs34449434 | A | C | 0.392 | -0.050 | 0.011 | 9.85E-06 | 0.005 | 0.022 | 0.815 | 0.001 | | 21.623 |  |
|  |  | rs4669806 | G | T | 0.214 | 0.058 | 0.012 | 2.42E-06 | -0.009 | 0.026 | 0.742 | 0.001 | | 20.518 |  |
|  |  | rs6494922 | A | G | 0.054 | 0.090 | 0.020 | 6.62E-06 | 0.054 | 0.047 | 0.248 | 0.001 | | 15.296 |  |
|  |  | rs6563550 | T | C | 0.078 | 0.088 | 0.018 | 2.35E-07 | -0.006 | 0.039 | 0.878 | 0.001 | | 20.372 |  |
|  |  | rs7155595 | C | A | 0.302 | 0.054 | 0.012 | 7.55E-06 | -0.009 | 0.023 | 0.704 | 0.001 | | 22.508 |  |
|  |  | rs8005030 | C | T | 0.331 | 0.055 | 0.012 | 2.28E-06 | 0.036 | 0.023 | 0.114 | 0.001 | | 25.016 |  |
|  |  | rs9347879 | T | C | 0.489 | 0.051 | 0.011 | 4.22E-06 | 0.006 | 0.021 | 0.793 | 0.001 | | 23.511 |  |
| ebi-a-GCST90016968 | Gut microbiota abundance (genus Bacteroides id.918) | rs11585893 | A | G | 0.247 | -0.074 | 0.015 | 1.80E-06 | -0.045 | 0.025 | 0.071 | 0.002 | | 37.506 |  |
|  |  | rs13207588 | A | G | 0.195 | -0.059 | 0.013 | 7.48E-06 | -0.023 | 0.027 | 0.402 | 0.001 | | 20.234 |  |
|  |  | rs1340391 | T | C | 0.135 | -0.059 | 0.013 | 6.73E-06 | -0.001 | 0.031 | 0.963 | 0.001 | | 15.022 |  |
|  |  | rs17619981 | T | G | 0.135 | 0.088 | 0.019 | 2.69E-06 | 0.018 | 0.031 | 0.561 | 0.002 | | 33.280 |  |
|  |  | rs2023437 | T | C | 0.124 | -0.078 | 0.017 | 5.02E-06 | 0.007 | 0.032 | 0.834 | 0.001 | | 24.432 |  |
|  |  | rs66474973 | G | T | 0.101 | 0.081 | 0.016 | 6.81E-07 | -0.004 | 0.035 | 0.907 | 0.001 | | 22.069 |  |
|  |  | rs66710942 | T | C | 0.475 | -0.049 | 0.011 | 5.86E-06 | -0.057 | 0.021 | 0.007 | 0.001 | | 21.810 |  |
|  |  | rs6795673 | C | T | 0.479 | 0.054 | 0.011 | 3.38E-07 | -0.004 | 0.021 | 0.846 | 0.001 | | 26.585 |  |
|  |  | rs9507307 | C | T | 0.245 | 0.060 | 0.013 | 2.13E-06 | -0.052 | 0.025 | 0.034 | 0.001 | | 24.814 |  |
| ebi-a-GCST90016969 | Gut microbiota abundance (genus Barnesiella id.944) | rs11155559 | T | C | 0.092 | 0.096 | 0.021 | 8.92E-06 | 0.044 | 0.037 | 0.233 | 0.002 | | 27.987 |  |
|  |  | rs113258194 | A | G | 0.096 | 0.099 | 0.021 | 7.31E-06 | -0.113 | 0.036 | 0.002 | 0.002 | | 31.267 |  |
|  |  | rs12909713 | C | T | 0.489 | -0.055 | 0.012 | 4.95E-06 | 0.033 | 0.021 | 0.120 | 0.002 | | 27.837 |  |
|  |  | rs13242616 | C | T | 0.675 | 0.058 | 0.012 | 2.29E-06 | -0.045 | 0.023 | 0.048 | 0.001 | | 27.454 |  |
|  |  | rs199035 | G | A | 0.555 | 0.056 | 0.012 | 3.00E-06 | 0.002 | 0.021 | 0.944 | 0.002 | | 28.396 |  |
|  |  | rs2276875 | A | G | 0.260 | -0.070 | 0.014 | 4.65E-07 | 0.038 | 0.024 | 0.121 | 0.002 | | 34.397 |  |
|  |  | rs2428166 | G | A | 0.014 | -0.166 | 0.034 | 8.51E-07 | 0.101 | 0.089 | 0.256 | 0.001 | | 14.193 |  |
|  |  | rs35177866 | A | G | 0.077 | 0.092 | 0.019 | 2.95E-06 | -0.068 | 0.041 | 0.093 | 0.001 | | 21.862 |  |
|  |  | rs62251337 | G | A | 0.858 | 0.069 | 0.015 | 4.24E-06 | -0.018 | 0.030 | 0.545 | 0.001 | | 21.296 |  |
|  |  | rs72684847 | T | C | 0.075 | -0.114 | 0.025 | 6.76E-06 | 0.015 | 0.040 | 0.711 | 0.002 | | 33.309 |  |
|  |  | rs76181748 | C | T | 0.230 | -0.078 | 0.017 | 6.78E-06 | 0.039 | 0.025 | 0.126 | 0.002 | | 39.480 |  |
|  |  | rs77455852 | T | G | 0.151 | -0.089 | 0.020 | 3.16E-06 | -0.002 | 0.031 | 0.945 | 0.002 | | 37.424 |  |
|  |  | rs79795328 | A | G | 0.139 | -0.082 | 0.018 | 4.23E-06 | 0.012 | 0.031 | 0.707 | 0.002 | | 29.482 |  |
| ebi-a-GCST90016970 | Gut microbiota abundance (genus Bifidobacterium id.436) | rs12022129 | A | G | 0.725 | -0.062 | 0.014 | 8.00E-06 | 0.015 | 0.024 | 0.519 | 0.002 | | 28.099 |  |
|  |  | rs182549 | T | C | 0.598 | -0.120 | 0.013 | 1.28E-20 | -0.048 | 0.022 | 0.026 | 0.007 | | 127.262 |  |
|  |  | rs2491158 | A | G | 0.867 | -0.071 | 0.016 | 8.05E-06 | 0.029 | 0.031 | 0.353 | 0.001 | | 21.461 |  |
|  |  | rs2686790 | C | T | 0.853 | -0.071 | 0.016 | 7.50E-06 | 0.013 | 0.030 | 0.653 | 0.001 | | 23.082 |  |
|  |  | rs4957061 | T | C | 0.423 | 0.053 | 0.012 | 5.78E-06 | -0.006 | 0.022 | 0.787 | 0.001 | | 25.578 |  |
|  |  | rs540489 | T | G | 0.176 | -0.064 | 0.014 | 5.19E-06 | -0.015 | 0.028 | 0.592 | 0.001 | | 21.651 |  |
|  |  | rs55888705 | A | G | 0.282 | 0.055 | 0.012 | 6.67E-06 | -0.020 | 0.023 | 0.390 | 0.001 | | 22.176 |  |
|  |  | rs5746486 | T | C | 0.387 | -0.054 | 0.012 | 9.00E-06 | 0.014 | 0.022 | 0.532 | 0.001 | | 25.039 |  |
|  |  | rs62181700 | G | A | 0.245 | -0.062 | 0.013 | 2.17E-06 | 0.006 | 0.025 | 0.819 | 0.001 | | 26.516 |  |
|  |  | rs7322849 | T | C | 0.092 | 0.112 | 0.020 | 1.08E-08 | 0.055 | 0.037 | 0.140 | 0.002 | | 38.869 |  |
|  |  | rs73797465 | T | G | 0.115 | -0.095 | 0.021 | 4.38E-06 | -0.022 | 0.034 | 0.516 | 0.002 | | 34.004 |  |
|  |  | rs75344046 | C | T | 0.047 | 0.232 | 0.051 | 4.86E-06 | -0.012 | 0.050 | 0.814 | 0.005 | | 89.555 |  |
|  |  | rs857444 | C | T | 0.369 | 0.056 | 0.012 | 3.57E-06 | -0.035 | 0.022 | 0.108 | 0.001 | | 26.638 |  |
| ebi-a-GCST90016971 | Gut microbiota abundance (genus Bilophila id.3170) | rs11069458 | C | T | 0.814 | 0.068 | 0.016 | 7.72E-06 | 0.012 | 0.027 | 0.665 | 0.001 | | 25.806 |  |
|  |  | rs1241171 | G | A | 0.144 | -0.069 | 0.015 | 4.24E-06 | 0.043 | 0.030 | 0.150 | 0.001 | | 21.765 |  |
|  |  | rs1571225 | T | C | 0.835 | -0.083 | 0.017 | 1.12E-06 | -0.022 | 0.029 | 0.444 | 0.002 | | 34.644 |  |
|  |  | rs1969927 | A | G | 0.665 | -0.056 | 0.013 | 9.07E-06 | -0.018 | 0.023 | 0.417 | 0.001 | | 26.074 |  |
|  |  | rs2728491 | T | G | 0.761 | 0.063 | 0.014 | 6.33E-06 | 0.019 | 0.025 | 0.450 | 0.001 | | 26.297 |  |
|  |  | rs3827020 | C | T | 0.196 | 0.077 | 0.016 | 1.79E-06 | 0.017 | 0.027 | 0.530 | 0.002 | | 34.057 |  |
|  |  | rs4798126 | G | A | 0.197 | 0.073 | 0.017 | 7.15E-06 | 0.037 | 0.027 | 0.163 | 0.002 | | 31.251 |  |
|  |  | rs542415 | T | C | 0.391 | -0.061 | 0.013 | 4.71E-06 | 0.013 | 0.022 | 0.546 | 0.002 | | 32.928 |  |
|  |  | rs60178956 | G | A | 0.226 | -0.062 | 0.014 | 8.06E-06 | 0.031 | 0.025 | 0.215 | 0.001 | | 25.112 |  |
|  |  | rs6793291 | A | C | 0.947 | -0.113 | 0.024 | 3.11E-06 | 0.007 | 0.048 | 0.883 | 0.001 | | 23.381 |  |
|  |  | rs72676854 | T | C | 0.053 | 0.123 | 0.027 | 5.62E-06 | -0.022 | 0.048 | 0.640 | 0.002 | | 28.037 |  |
|  |  | rs7802841 | A | C | 0.697 | -0.067 | 0.014 | 1.77E-06 | -0.016 | 0.023 | 0.491 | 0.002 | | 34.846 |  |
|  |  | rs9899990 | A | G | 0.084 | -0.103 | 0.023 | 9.07E-06 | 0.016 | 0.038 | 0.669 | 0.002 | | 29.956 |  |
| ebi-a-GCST90016972 | Gut microbiota abundance (genus Blautia id.1992) | rs115043014 | G | A | 0.017 | -0.207 | 0.044 | 5.19E-06 | 0.100 | 0.081 | 0.218 | 0.001 | | 26.730 |  |
|  |  | rs2788271 | G | T | 0.827 | 0.058 | 0.013 | 7.16E-06 | -0.037 | 0.028 | 0.189 | 0.001 | | 17.393 |  |
| ebi-a-GCST90016973 | Gut microbiota abundance (genus Butyricicoccus id.2055) | rs10084203 | G | A | 0.870 | -0.055 | 0.012 | 8.59E-06 | 0.022 | 0.031 | 0.480 | 0.001 | | 12.567 |  |
|  |  | rs12034718 | G | A | 0.776 | -0.070 | 0.016 | 9.58E-06 | -0.015 | 0.025 | 0.561 | 0.002 | | 31.359 |  |
|  |  | rs12585793 | T | C | 0.029 | -0.262 | 0.056 | 5.79E-06 | 0.020 | 0.063 | 0.756 | 0.004 | | 71.232 |  |
|  |  | rs2017189 | T | G | 0.528 | 0.051 | 0.011 | 3.87E-06 | 0.019 | 0.021 | 0.373 | 0.001 | | 23.519 |  |
|  |  | rs4962426 | T | G | 0.805 | -0.061 | 0.014 | 7.38E-06 | 0.028 | 0.027 | 0.290 | 0.001 | | 21.779 |  |
|  |  | rs56221232 | T | C | 0.100 | 0.083 | 0.017 | 7.62E-07 | -0.032 | 0.035 | 0.365 | 0.001 | | 22.720 |  |
|  |  | rs62478070 | T | G | 0.026 | 0.224 | 0.049 | 5.94E-06 | -0.080 | 0.068 | 0.238 | 0.003 | | 46.088 |  |
|  |  | rs7322368 | C | T | 0.909 | -0.082 | 0.018 | 5.52E-06 | 0.027 | 0.037 | 0.466 | 0.001 | | 20.230 |  |
| ebi-a-GCST90016974 | Gut microbiota abundance (genus Butyricimonas id.945) | rs11228830 | A | G | 0.085 | 0.135 | 0.030 | 6.55E-06 | -0.023 | 0.039 | 0.557 | 0.003 | | 52.592 |  |
|  |  | rs113054641 | G | A | 0.044 | -0.145 | 0.027 | 1.74E-07 | -0.044 | 0.051 | 0.390 | 0.002 | | 32.771 |  |
|  |  | rs12304031 | G | A | 0.123 | -0.086 | 0.020 | 6.70E-06 | 0.007 | 0.032 | 0.834 | 0.002 | | 29.498 |  |
|  |  | rs12458763 | A | C | 0.045 | 0.122 | 0.027 | 6.37E-06 | 0.000 | 0.050 | 0.998 | 0.001 | | 23.514 |  |
|  |  | rs1862649 | G | A | 0.069 | 0.113 | 0.025 | 4.76E-06 | -0.014 | 0.042 | 0.744 | 0.002 | | 30.139 |  |
|  |  | rs2114713 | G | T | 0.449 | 0.063 | 0.014 | 6.88E-06 | -0.003 | 0.021 | 0.895 | 0.002 | | 35.766 |  |
|  |  | rs62130338 | A | G | 0.655 | 0.073 | 0.016 | 3.90E-06 | 0.001 | 0.022 | 0.956 | 0.002 | | 44.572 |  |
|  |  | rs62390301 | T | C | 0.200 | -0.087 | 0.017 | 7.42E-07 | 0.006 | 0.027 | 0.819 | 0.002 | | 44.797 |  |
|  |  | rs7083431 | A | C | 0.274 | 0.070 | 0.014 | 8.85E-07 | -0.023 | 0.024 | 0.329 | 0.002 | | 36.216 |  |
|  |  | rs71428626 | G | T | 0.032 | -0.133 | 0.029 | 4.80E-06 | 0.025 | 0.059 | 0.673 | 0.001 | | 20.315 |  |
|  |  | rs72814525 | A | G | 0.244 | 0.066 | 0.015 | 8.25E-06 | -0.001 | 0.025 | 0.959 | 0.002 | | 29.874 |  |
|  |  | rs78453362 | A | G | 0.025 | -0.149 | 0.033 | 4.06E-06 | -0.037 | 0.067 | 0.587 | 0.001 | | 20.348 |  |
|  |  | rs9657374 | C | T | 0.294 | 0.068 | 0.015 | 4.50E-06 | 0.078 | 0.023 | 0.001 | 0.002 | | 35.308 |  |
| ebi-a-GCST90016975 | Gut microbiota abundance (genus Butyrivibrio id.1993) | rs1007475 | G | T | 0.277 | 0.118 | 0.026 | 7.92E-06 | -0.006 | 0.024 | 0.808 | 0.006 | | 102.938 |  |
|  |  | rs11761679 | T | C | 0.140 | 0.155 | 0.032 | 2.20E-06 | 0.074 | 0.030 | 0.015 | 0.006 | | 106.139 |  |
|  |  | rs142855850 | A | G | 0.098 | 0.205 | 0.046 | 6.86E-06 | -0.031 | 0.036 | 0.386 | 0.007 | | 137.484 |  |
|  |  | rs16934069 | T | C | 0.178 | -0.134 | 0.030 | 8.86E-06 | 0.012 | 0.028 | 0.672 | 0.005 | | 96.595 |  |
|  |  | rs16941336 | C | T | 0.231 | 0.127 | 0.027 | 1.53E-06 | -0.004 | 0.025 | 0.886 | 0.006 | | 106.538 |  |
|  |  | rs17163238 | G | A | 0.192 | 0.141 | 0.031 | 5.51E-06 | 0.050 | 0.027 | 0.063 | 0.006 | | 113.572 |  |
|  |  | rs4537857 | T | C | 0.333 | -0.125 | 0.026 | 1.80E-06 | 0.018 | 0.022 | 0.420 | 0.007 | | 127.328 |  |
|  |  | rs486484 | A | G | 0.426 | -0.108 | 0.024 | 6.61E-06 | 0.007 | 0.021 | 0.763 | 0.006 | | 105.864 |  |
|  |  | rs4928024 | G | A | 0.815 | 0.175 | 0.039 | 8.19E-06 | 0.005 | 0.028 | 0.855 | 0.009 | | 170.645 |  |
|  |  | rs72723662 | C | T | 0.142 | 0.224 | 0.045 | 7.86E-07 | 0.005 | 0.031 | 0.887 | 0.012 | | 227.415 |  |
|  |  | rs74622183 | A | G | 0.089 | -0.201 | 0.043 | 2.46E-06 | -0.019 | 0.037 | 0.617 | 0.007 | | 121.420 |  |
|  |  | rs77356209 | T | C | 0.046 | 0.217 | 0.048 | 6.66E-06 | -0.032 | 0.051 | 0.530 | 0.004 | | 75.968 |  |
|  |  | rs7752361 | G | A | 0.518 | 0.119 | 0.024 | 7.69E-07 | -0.055 | 0.021 | 0.010 | 0.007 | | 131.116 |  |
|  |  | rs7763512 | A | G | 0.576 | -0.120 | 0.025 | 3.11E-06 | 0.028 | 0.022 | 0.190 | 0.007 | | 129.577 |  |
|  |  | rs9349693 | G | A | 0.709 | -0.118 | 0.026 | 5.55E-06 | -0.028 | 0.023 | 0.221 | 0.006 | | 106.009 |  |
| ebi-a-GCST90016976 | Gut microbiota abundance (genus Candidatus Soleaferrea id.11350) | rs10090365 | G | A | 0.522 | 0.083 | 0.018 | 4.17E-06 | -0.020 | 0.021 | 0.340 | 0.003 | | 63.941 |  |
|  |  | rs10108780 | A | G | 0.243 | -0.093 | 0.020 | 3.64E-06 | -0.023 | 0.025 | 0.344 | 0.003 | | 58.279 |  |
|  |  | rs10809135 | C | T | 0.557 | -0.083 | 0.018 | 5.47E-06 | 0.013 | 0.022 | 0.554 | 0.003 | | 63.309 |  |
|  |  | rs36155147 | T | C | 0.660 | -0.105 | 0.024 | 5.41E-06 | 0.041 | 0.023 | 0.075 | 0.005 | | 91.116 |  |
|  |  | rs4294381 | C | T | 0.843 | -0.112 | 0.023 | 1.37E-06 | 0.043 | 0.029 | 0.141 | 0.003 | | 61.468 |  |
|  |  | rs4678258 | T | C | 0.242 | 0.099 | 0.022 | 5.53E-06 | 0.029 | 0.025 | 0.249 | 0.004 | | 65.696 |  |
|  |  | rs6489992 | A | G | 0.372 | -0.084 | 0.019 | 7.89E-06 | -0.034 | 0.022 | 0.125 | 0.003 | | 60.700 |  |
|  |  | rs6494306 | A | G | 0.324 | -0.097 | 0.021 | 5.80E-06 | -0.013 | 0.023 | 0.557 | 0.004 | | 75.736 |  |
|  |  | rs7400877 | C | T | 0.786 | 0.095 | 0.021 | 9.29E-06 | -0.017 | 0.026 | 0.522 | 0.003 | | 55.897 |  |
|  |  | rs830151 | A | G | 0.940 | -0.195 | 0.035 | 4.26E-08 | 0.067 | 0.045 | 0.133 | 0.004 | | 78.391 |  |
|  |  | rs9973954 | G | A | 0.644 | -0.089 | 0.020 | 5.95E-06 | 0.007 | 0.022 | 0.756 | 0.004 | | 67.202 |  |
| ebi-a-GCST90016977 | Gut microbiota abundance (genus Catenibacterium id.2153) | rs12404911 | C | T | 0.185 | 0.141 | 0.030 | 2.80E-06 | -0.020 | 0.027 | 0.464 | 0.006 | | 110.109 |  |
|  |  | rs212393 | A | G | 0.793 | 0.135 | 0.029 | 3.62E-06 | -0.045 | 0.026 | 0.088 | 0.006 | | 110.880 |  |
|  |  | rs73128290 | A | G | 0.300 | 0.130 | 0.028 | 4.29E-06 | 0.024 | 0.023 | 0.304 | 0.007 | | 130.613 |  |
|  |  | rs7742829 | C | T | 0.437 | 0.114 | 0.025 | 5.61E-06 | 0.002 | 0.021 | 0.935 | 0.006 | | 118.234 |  |
| ebi-a-GCST90016978 | Gut microbiota abundance (genus Christensenellaceae R 7group id.11283) | rs10461257 | A | G | 0.352 | -0.055 | 0.012 | 6.51E-06 | -0.032 | 0.023 | 0.159 | 0.001 | | 25.516 |  |
|  |  | rs17081797 | A | G | 0.065 | -0.090 | 0.020 | 3.34E-06 | -0.124 | 0.043 | 0.004 | 0.001 | | 18.166 |  |
|  |  | rs60954665 | T | G | 0.496 | 0.050 | 0.011 | 7.13E-06 | -0.024 | 0.021 | 0.259 | 0.001 | | 22.780 |  |
|  |  | rs62132810 | A | G | 0.144 | -0.083 | 0.018 | 5.67E-06 | 0.000 | 0.031 | 0.994 | 0.002 | | 31.147 |  |
|  |  | rs62190261 | A | C | 0.088 | 0.096 | 0.021 | 8.74E-06 | 0.036 | 0.038 | 0.337 | 0.001 | | 26.943 |  |
|  |  | rs62467127 | C | T | 0.025 | 0.114 | 0.025 | 3.25E-06 | 0.070 | 0.067 | 0.298 | 0.001 | | 11.702 |  |
|  |  | rs73952017 | C | T | 0.103 | -0.086 | 0.019 | 8.46E-06 | -0.051 | 0.035 | 0.141 | 0.001 | | 25.114 |  |
|  |  | rs78521377 | C | T | 0.030 | 0.125 | 0.027 | 5.61E-06 | 0.046 | 0.061 | 0.450 | 0.001 | | 16.829 |  |
|  |  | rs79150079 | C | A | 0.068 | 0.122 | 0.027 | 9.42E-06 | 0.045 | 0.042 | 0.283 | 0.002 | | 34.338 |  |
|  |  | rs892686 | A | G | 0.473 | 0.051 | 0.011 | 3.97E-06 | -0.034 | 0.021 | 0.111 | 0.001 | | 24.193 |  |
| ebi-a-GCST90016979 | Gut microbiota abundance (genus Clostridium innocuum group id.14397) | rs10074000 | T | C | 0.377 | -0.103 | 0.023 | 7.00E-06 | -0.040 | 0.022 | 0.070 | 0.005 | | 91.244 |  |
|  |  | rs10506058 | A | G | 0.422 | 0.100 | 0.022 | 8.92E-06 | -0.058 | 0.021 | 0.006 | 0.005 | | 89.388 |  |
|  |  | rs1942371 | G | A | 0.124 | -0.158 | 0.034 | 4.06E-06 | 0.001 | 0.033 | 0.984 | 0.005 | | 99.639 |  |
|  |  | rs40656 | C | T | 0.206 | 0.143 | 0.031 | 8.62E-06 | 0.015 | 0.027 | 0.582 | 0.007 | | 122.914 |  |
|  |  | rs4869133 | G | A | 0.177 | -0.181 | 0.041 | 7.24E-06 | 0.008 | 0.028 | 0.779 | 0.010 | | 176.147 |  |
|  |  | rs61267978 | T | C | 0.121 | 0.147 | 0.032 | 5.59E-06 | -0.012 | 0.032 | 0.714 | 0.005 | | 85.017 |  |
|  |  | rs6577484 | G | A | 0.104 | 0.160 | 0.036 | 8.41E-06 | -0.020 | 0.035 | 0.563 | 0.005 | | 88.607 |  |
|  |  | rs6890185 | C | T | 0.675 | -0.113 | 0.023 | 1.12E-06 | 0.000 | 0.023 | 0.991 | 0.006 | | 104.080 |  |
|  |  | rs77845139 | A | G | 0.245 | -0.115 | 0.026 | 8.41E-06 | -0.017 | 0.025 | 0.502 | 0.005 | | 90.175 |  |
| ebi-a-GCST90016980 | Gut microbiota abundance (genus Clostridium sensustricto1 id.1873) | rs11264403 | G | A | 0.076 | -0.139 | 0.033 | 7.76E-06 | -0.022 | 0.040 | 0.579 | 0.003 | | 49.699 |  |
|  |  | rs115807074 | A | G | 0.011 | -0.227 | 0.049 | 4.32E-06 | 0.029 | 0.101 | 0.770 | 0.001 | | 20.704 |  |
|  |  | rs116847295 | C | T | 0.127 | 0.110 | 0.025 | 4.58E-06 | -0.052 | 0.032 | 0.106 | 0.003 | | 49.420 |  |
|  |  | rs12341505 | G | A | 0.094 | 0.081 | 0.018 | 4.82E-06 | -0.073 | 0.036 | 0.043 | 0.001 | | 20.456 |  |
|  |  | rs2795528 | G | A | 0.056 | -0.184 | 0.039 | 2.72E-06 | 0.010 | 0.047 | 0.825 | 0.004 | | 65.937 |  |
|  |  | rs2817172 | C | T | 0.396 | 0.058 | 0.012 | 2.77E-06 | -0.030 | 0.022 | 0.166 | 0.002 | | 29.702 |  |
|  |  | rs550843 | T | C | 0.282 | -0.078 | 0.017 | 2.05E-06 | 0.026 | 0.024 | 0.269 | 0.002 | | 45.700 |  |
| ebi-a-GCST90016981 | Gut microbiota abundance (genus Collinsella id.815) | rs10890671 | C | T | 0.437 | 0.054 | 0.012 | 6.52E-06 | -0.020 | 0.021 | 0.345 | 0.001 | | 26.095 |  |
|  |  | rs11597285 | G | T | 0.399 | -0.054 | 0.012 | 9.38E-06 | -0.016 | 0.022 | 0.465 | 0.001 | | 25.487 |  |
|  |  | rs1496626 | T | C | 0.135 | -0.072 | 0.016 | 6.78E-06 | 0.009 | 0.031 | 0.782 | 0.001 | | 22.302 |  |
|  |  | rs149807560 | C | A | 0.069 | -0.104 | 0.024 | 7.10E-06 | -0.050 | 0.042 | 0.234 | 0.001 | | 25.631 |  |
|  |  | rs2103510 | G | A | 0.116 | 0.079 | 0.017 | 2.42E-06 | 0.035 | 0.033 | 0.293 | 0.001 | | 23.332 |  |
|  |  | rs62448871 | C | A | 0.536 | -0.054 | 0.012 | 6.78E-06 | -0.030 | 0.021 | 0.155 | 0.001 | | 26.668 |  |
|  |  | rs73052258 | G | A | 0.077 | 0.093 | 0.020 | 1.72E-06 | -0.050 | 0.040 | 0.213 | 0.001 | | 22.562 |  |
|  |  | rs75672793 | A | G | 0.046 | -0.109 | 0.024 | 6.14E-06 | 0.073 | 0.050 | 0.146 | 0.001 | | 19.082 |  |
|  |  | rs9541268 | C | A | 0.090 | 0.096 | 0.020 | 8.79E-07 | -0.006 | 0.037 | 0.865 | 0.002 | | 27.782 |  |
| ebi-a-GCST90016982 | Gut microbiota abundance (genus Coprobacter id.949) | rs11532348 | C | T | 0.150 | -0.104 | 0.023 | 5.71E-06 | 0.018 | 0.030 | 0.545 | 0.003 | | 50.594 |  |
|  |  | rs12684609 | T | C | 0.197 | 0.101 | 0.022 | 6.10E-06 | 0.012 | 0.027 | 0.650 | 0.003 | | 59.240 |  |
|  |  | rs12996055 | A | C | 0.268 | 0.092 | 0.021 | 8.08E-06 | -0.006 | 0.024 | 0.809 | 0.003 | | 61.358 |  |
|  |  | rs143662916 | C | T | 0.030 | 0.253 | 0.054 | 3.07E-06 | 0.027 | 0.062 | 0.668 | 0.004 | | 67.733 |  |
|  |  | rs189356 | G | A | 0.571 | 0.078 | 0.017 | 6.26E-06 | 0.014 | 0.021 | 0.518 | 0.003 | | 54.991 |  |
|  |  | rs213863 | T | C | 0.640 | 0.089 | 0.019 | 2.35E-06 | 0.003 | 0.022 | 0.888 | 0.004 | | 66.804 |  |
|  |  | rs305411 | A | G | 0.104 | 0.129 | 0.026 | 1.01E-06 | 0.007 | 0.035 | 0.840 | 0.003 | | 56.999 |  |
|  |  | rs3828477 | G | T | 0.344 | -0.091 | 0.020 | 2.89E-06 | 0.010 | 0.022 | 0.644 | 0.004 | | 69.116 |  |
|  |  | rs72821405 | T | C | 0.089 | -0.147 | 0.032 | 4.76E-06 | -0.060 | 0.038 | 0.108 | 0.004 | | 64.931 |  |
|  |  | rs74919520 | G | A | 0.107 | 0.126 | 0.028 | 5.76E-06 | 0.017 | 0.034 | 0.610 | 0.003 | | 55.725 |  |
| ebi-a-GCST90016983 | Gut microbiota abundance (genus Coprococcus1 id.11301) | rs1010560 | C | A | 0.268 | 0.058 | 0.012 | 1.96E-06 | -0.007 | 0.024 | 0.771 | 0.001 | | 24.272 |  |
|  |  | rs12794898 | G | T | 0.129 | 0.090 | 0.020 | 4.92E-06 | 0.019 | 0.032 | 0.562 | 0.002 | | 33.686 |  |
|  |  | rs1519491 | T | C | 0.397 | 0.050 | 0.011 | 8.95E-06 | 0.008 | 0.022 | 0.703 | 0.001 | | 21.907 |  |
|  |  | rs1576241 | A | G | 0.381 | -0.051 | 0.011 | 3.33E-06 | 0.004 | 0.022 | 0.844 | 0.001 | | 22.549 |  |
|  |  | rs2907920 | G | A | 0.716 | -0.056 | 0.013 | 7.65E-06 | -0.019 | 0.024 | 0.420 | 0.001 | | 23.541 |  |
|  |  | rs4277593 | G | A | 0.410 | -0.059 | 0.011 | 1.14E-07 | -0.034 | 0.021 | 0.113 | 0.002 | | 30.484 |  |
|  |  | rs56405618 | A | G | 0.116 | -0.090 | 0.019 | 1.57E-06 | -0.014 | 0.033 | 0.677 | 0.002 | | 30.172 |  |
|  |  | rs73031725 | T | C | 0.031 | 0.168 | 0.036 | 1.98E-06 | -0.047 | 0.061 | 0.436 | 0.002 | | 31.325 |  |
|  |  | rs73167075 | T | C | 0.210 | 0.057 | 0.013 | 8.57E-06 | -0.036 | 0.026 | 0.168 | 0.001 | | 20.009 |  |
|  |  | rs74101919 | T | C | 0.108 | -0.072 | 0.014 | 1.03E-06 | -0.020 | 0.034 | 0.560 | 0.001 | | 18.343 |  |
|  |  | rs946513 | T | C | 0.954 | -0.206 | 0.046 | 8.62E-06 | 0.034 | 0.051 | 0.513 | 0.004 | | 68.488 |  |
| ebi-a-GCST90016984 | Gut microbiota abundance (genus Coprococcus2 id.11302) | rs10070053 | A | G | 0.415 | 0.059 | 0.014 | 7.65E-06 | -0.008 | 0.021 | 0.722 | 0.002 | | 31.497 |  |
|  |  | rs12634070 | T | C | 0.257 | 0.074 | 0.016 | 9.95E-06 | 0.021 | 0.024 | 0.395 | 0.002 | | 38.046 |  |
|  |  | rs2482516 | C | T | 0.226 | 0.075 | 0.016 | 4.72E-06 | -0.021 | 0.025 | 0.415 | 0.002 | | 36.621 |  |
|  |  | rs35890118 | A | G | 0.254 | -0.067 | 0.015 | 8.26E-06 | -0.026 | 0.024 | 0.293 | 0.002 | | 30.777 |  |
|  |  | rs61823518 | A | C | 0.106 | -0.096 | 0.022 | 6.68E-06 | -0.118 | 0.035 | 0.001 | 0.002 | | 31.778 |  |
|  |  | rs6677933 | C | T | 0.147 | -0.080 | 0.016 | 1.19E-06 | 0.015 | 0.030 | 0.622 | 0.002 | | 29.789 |  |
|  |  | rs72680320 | T | C | 0.336 | -0.065 | 0.014 | 2.27E-06 | 0.023 | 0.022 | 0.295 | 0.002 | | 34.571 |  |
|  |  | rs9426473 | A | G | 0.258 | 0.073 | 0.016 | 6.31E-06 | 0.004 | 0.024 | 0.886 | 0.002 | | 37.211 |  |
| ebi-a-GCST90016985 | Gut microbiota abundance (genus Coprococcus3 id.11303) | rs10810043 | G | A | 0.664 | -0.052 | 0.012 | 9.27E-06 | 0.003 | 0.023 | 0.900 | 0.001 | | 21.772 |  |
|  |  | rs11077359 | C | T | 0.830 | 0.065 | 0.015 | 9.64E-06 | 0.024 | 0.028 | 0.409 | 0.001 | | 21.604 |  |
|  |  | rs11080344 | C | T | 0.445 | 0.052 | 0.011 | 4.79E-06 | 0.021 | 0.021 | 0.331 | 0.001 | | 24.233 |  |
|  |  | rs13247359 | G | A | 0.446 | 0.051 | 0.011 | 7.33E-06 | -0.003 | 0.021 | 0.907 | 0.001 | | 23.795 |  |
|  |  | rs13394391 | C | T | 0.154 | -0.071 | 0.015 | 2.20E-06 | -0.007 | 0.029 | 0.824 | 0.001 | | 24.046 |  |
|  |  | rs178271 | C | T | 0.983 | -0.145 | 0.029 | 7.81E-07 | 0.056 | 0.082 | 0.494 | 0.001 | | 12.866 |  |
|  |  | rs4575475 | A | G | 0.770 | -0.062 | 0.014 | 7.04E-06 | 0.015 | 0.025 | 0.544 | 0.001 | | 24.963 |  |
|  |  | rs7521171 | A | G | 0.690 | 0.060 | 0.013 | 4.32E-06 | 0.009 | 0.023 | 0.709 | 0.002 | | 27.970 |  |
|  |  | rs8100692 | T | C | 0.558 | 0.058 | 0.011 | 4.16E-07 | 0.022 | 0.021 | 0.307 | 0.002 | | 30.208 |  |
| ebi-a-GCST90016986 | Gut microbiota abundance (genus Defluviitaleaceae UCG011 id.11287) | rs112893842 | T | C | 0.090 | 0.114 | 0.023 | 1.45E-06 | -0.043 | 0.037 | 0.243 | 0.002 | | 38.892 |  |
|  |  | rs1582238 | C | T | 0.641 | -0.081 | 0.017 | 1.57E-06 | -0.020 | 0.022 | 0.366 | 0.003 | | 54.874 |  |
|  |  | rs2892880 | G | A | 0.249 | 0.082 | 0.018 | 6.83E-06 | 0.003 | 0.025 | 0.915 | 0.003 | | 45.991 |  |
|  |  | rs4344384 | T | G | 0.504 | -0.072 | 0.016 | 4.83E-06 | 0.018 | 0.021 | 0.386 | 0.003 | | 47.120 |  |
|  |  | rs4677103 | A | G | 0.181 | 0.098 | 0.020 | 9.60E-07 | 0.027 | 0.028 | 0.329 | 0.003 | | 52.208 |  |
|  |  | rs55658617 | T | C | 0.035 | 0.174 | 0.036 | 2.15E-06 | -0.015 | 0.058 | 0.798 | 0.002 | | 37.824 |  |
|  |  | rs72731813 | C | T | 0.049 | -0.147 | 0.029 | 4.33E-07 | 0.023 | 0.049 | 0.640 | 0.002 | | 36.961 |  |
|  |  | rs9608282 | T | G | 0.034 | 0.143 | 0.030 | 2.52E-06 | 0.038 | 0.059 | 0.523 | 0.001 | | 24.442 |  |
|  |  | rs9725395 | A | G | 0.115 | -0.138 | 0.030 | 3.52E-06 | 0.007 | 0.033 | 0.839 | 0.004 | | 71.607 |  |
| ebi-a-GCST90016987 | Gut microbiota abundance (genus Desulfovibrio id.3173) | rs12031543 | T | C | 0.139 | -0.127 | 0.028 | 6.55E-06 | -0.033 | 0.031 | 0.294 | 0.004 | | 71.062 |  |
|  |  | rs13066142 | G | A | 0.091 | 0.119 | 0.025 | 3.79E-06 | 0.000 | 0.037 | 0.993 | 0.002 | | 43.105 |  |
|  |  | rs16863365 | A | G | 0.047 | 0.109 | 0.023 | 1.79E-06 | -0.058 | 0.050 | 0.239 | 0.001 | | 19.699 |  |
|  |  | rs2032031 | G | A | 0.493 | 0.065 | 0.015 | 9.14E-06 | -0.018 | 0.021 | 0.391 | 0.002 | | 39.406 |  |
|  |  | rs2456226 | A | C | 0.689 | 0.085 | 0.017 | 8.55E-07 | -0.003 | 0.023 | 0.913 | 0.003 | | 57.580 |  |
|  |  | rs2590913 | A | G | 0.947 | -0.154 | 0.034 | 6.65E-06 | -0.043 | 0.048 | 0.370 | 0.002 | | 43.729 |  |
|  |  | rs2853179 | T | C | 0.771 | -0.081 | 0.017 | 2.42E-06 | 0.017 | 0.025 | 0.498 | 0.002 | | 42.819 |  |
|  |  | rs4797774 | A | G | 0.956 | -0.213 | 0.047 | 5.64E-06 | 0.015 | 0.053 | 0.782 | 0.004 | | 69.679 |  |
|  |  | rs6580353 | T | C | 0.205 | 0.077 | 0.017 | 4.94E-06 | 0.031 | 0.027 | 0.248 | 0.002 | | 35.547 |  |
|  |  | rs72647089 | T | G | 0.080 | -0.107 | 0.024 | 8.30E-06 | 0.006 | 0.039 | 0.881 | 0.002 | | 30.775 |  |
| ebi-a-GCST90016988 | Gut microbiota abundance (genus Dialister id.2183) | rs10138457 | T | C | 0.093 | -0.113 | 0.026 | 7.88E-06 | 0.008 | 0.036 | 0.826 | 0.002 | | 39.634 |  |
|  |  | rs10938938 | A | G | 0.843 | 0.077 | 0.017 | 7.37E-06 | 0.016 | 0.029 | 0.578 | 0.002 | | 29.104 |  |
|  |  | rs11071887 | T | C | 0.324 | 0.066 | 0.015 | 5.91E-06 | 0.001 | 0.023 | 0.956 | 0.002 | | 35.326 |  |
|  |  | rs11166701 | G | A | 0.510 | -0.066 | 0.013 | 5.51E-07 | -0.015 | 0.021 | 0.475 | 0.002 | | 39.441 |  |
|  |  | rs2314294 | T | C | 0.136 | 0.087 | 0.019 | 8.08E-06 | -0.030 | 0.031 | 0.331 | 0.002 | | 32.312 |  |
|  |  | rs2435610 | A | C | 0.252 | 0.065 | 0.014 | 5.93E-06 | -0.003 | 0.024 | 0.913 | 0.002 | | 28.997 |  |
|  |  | rs4747450 | C | A | 0.225 | 0.067 | 0.015 | 5.84E-06 | 0.027 | 0.025 | 0.281 | 0.002 | | 28.608 |  |
|  |  | rs4753063 | A | G | 0.554 | 0.060 | 0.013 | 4.86E-06 | -0.003 | 0.021 | 0.906 | 0.002 | | 32.286 |  |
|  |  | rs75416973 | A | G | 0.221 | 0.073 | 0.016 | 9.46E-06 | 0.017 | 0.026 | 0.496 | 0.002 | | 33.486 |  |
|  |  | rs764177 | C | A | 0.367 | -0.060 | 0.014 | 9.61E-06 | -0.053 | 0.022 | 0.016 | 0.002 | | 30.888 |  |
|  |  | rs76680460 | G | A | 0.043 | -0.161 | 0.036 | 8.19E-06 | -0.067 | 0.053 | 0.204 | 0.002 | | 39.472 |  |
| ebi-a-GCST90016989 | Gut microbiota abundance (genus Dorea id.1997) | rs11150408 | G | T | 0.521 | -0.049 | 0.011 | 7.06E-06 | -0.008 | 0.021 | 0.718 | 0.001 | | 21.846 |  |
|  |  | rs12537781 | T | C | 0.238 | -0.056 | 0.013 | 9.15E-06 | 0.020 | 0.025 | 0.417 | 0.001 | | 20.538 |  |
|  |  | rs13279148 | G | A | 0.111 | 0.072 | 0.015 | 2.25E-06 | 0.047 | 0.034 | 0.165 | 0.001 | | 18.522 |  |
|  |  | rs1899291 | T | C | 0.842 | -0.070 | 0.015 | 4.57E-06 | -0.015 | 0.029 | 0.615 | 0.001 | | 23.749 |  |
|  |  | rs3005511 | G | A | 0.701 | -0.052 | 0.011 | 5.29E-06 | -0.016 | 0.023 | 0.488 | 0.001 | | 20.501 |  |
|  |  | rs345219 | G | T | 0.530 | 0.050 | 0.011 | 8.80E-06 | 0.035 | 0.021 | 0.106 | 0.001 | | 22.629 |  |
|  |  | rs3752849 | G | A | 0.050 | 0.164 | 0.037 | 7.68E-06 | 0.005 | 0.049 | 0.919 | 0.003 | | 46.941 |  |
|  |  | rs4793307 | C | T | 0.240 | 0.057 | 0.012 | 4.01E-06 | -0.005 | 0.025 | 0.847 | 0.001 | | 22.049 |  |
|  |  | rs62503162 | A | G | 0.043 | -0.097 | 0.019 | 7.47E-07 | 0.014 | 0.052 | 0.785 | 0.001 | | 14.284 |  |
|  |  | rs73729431 | C | T | 0.021 | -0.137 | 0.030 | 3.17E-06 | -0.050 | 0.075 | 0.506 | 0.001 | | 14.163 |  |
| ebi-a-GCST90016990 | Gut microbiota abundance (genus Eggerthella id.819) | rs112205261 | T | C | 0.082 | -0.189 | 0.040 | 3.35E-06 | 0.004 | 0.039 | 0.927 | 0.005 | | 98.232 |  |
|  |  | rs13070736 | A | C | 0.164 | -0.121 | 0.027 | 7.62E-06 | 0.014 | 0.029 | 0.614 | 0.004 | | 74.471 |  |
|  |  | rs1784446 | A | G | 0.512 | -0.091 | 0.020 | 5.23E-06 | -0.030 | 0.021 | 0.158 | 0.004 | | 75.820 |  |
|  |  | rs2223081 | A | G | 0.716 | -0.103 | 0.022 | 3.89E-06 | -0.011 | 0.024 | 0.648 | 0.004 | | 78.835 |  |
|  |  | rs2240838 | G | A | 0.460 | -0.098 | 0.020 | 7.36E-07 | -0.021 | 0.021 | 0.334 | 0.005 | | 88.028 |  |
|  |  | rs3851328 | G | T | 0.777 | 0.108 | 0.024 | 4.18E-06 | -0.031 | 0.025 | 0.225 | 0.004 | | 74.112 |  |
|  |  | rs4985746 | A | G | 0.897 | -0.111 | 0.025 | 5.71E-06 | 0.009 | 0.035 | 0.794 | 0.002 | | 41.449 |  |
|  |  | rs6430926 | T | C | 0.532 | -0.088 | 0.020 | 8.37E-06 | 0.005 | 0.021 | 0.805 | 0.004 | | 70.949 |  |
|  |  | rs67490567 | T | C | 0.258 | 0.108 | 0.025 | 8.94E-06 | 0.010 | 0.025 | 0.699 | 0.005 | | 82.979 |  |
|  |  | rs76663501 | C | T | 0.052 | 0.175 | 0.038 | 4.83E-06 | 0.039 | 0.048 | 0.410 | 0.003 | | 56.115 |  |
| ebi-a-GCST90016991 | Gut microbiota abundance (genus Eisenbergiella id.11304) | rs11027642 | C | T | 0.143 | 0.129 | 0.028 | 4.92E-06 | -0.050 | 0.030 | 0.098 | 0.004 | | 75.109 |  |
|  |  | rs11079158 | T | C | 0.217 | 0.101 | 0.023 | 7.35E-06 | 0.037 | 0.026 | 0.152 | 0.003 | | 63.212 |  |
|  |  | rs11938607 | C | T | 0.747 | -0.098 | 0.022 | 8.22E-06 | 0.031 | 0.024 | 0.198 | 0.004 | | 66.486 |  |
|  |  | rs12257723 | A | C | 0.327 | -0.095 | 0.021 | 8.85E-06 | 0.021 | 0.023 | 0.346 | 0.004 | | 73.512 |  |
|  |  | rs12710729 | C | A | 0.322 | 0.089 | 0.020 | 9.84E-06 | 0.001 | 0.023 | 0.975 | 0.003 | | 64.098 |  |
|  |  | rs13258851 | A | G | 0.142 | 0.137 | 0.030 | 7.75E-06 | 0.000 | 0.030 | 0.998 | 0.005 | | 84.006 |  |
|  |  | rs1508033 | A | C | 0.316 | 0.092 | 0.020 | 3.23E-06 | -0.013 | 0.023 | 0.565 | 0.004 | | 66.655 |  |
|  |  | rs1553971 | T | G | 0.226 | 0.121 | 0.026 | 5.27E-06 | -0.011 | 0.026 | 0.658 | 0.005 | | 94.299 |  |
|  |  | rs2683098 | T | C | 0.783 | -0.107 | 0.023 | 2.24E-06 | -0.036 | 0.026 | 0.161 | 0.004 | | 71.958 |  |
|  |  | rs3812426 | A | G | 0.845 | -0.106 | 0.022 | 2.72E-06 | -0.029 | 0.029 | 0.321 | 0.003 | | 54.707 |  |
|  |  | rs4462860 | G | A | 0.397 | 0.094 | 0.020 | 4.16E-06 | -0.049 | 0.022 | 0.024 | 0.004 | | 77.748 |  |
| ebi-a-GCST90016992 | Gut microbiota abundance (genus Enterorhabdus id.820) | rs10098492 | T | C | 0.057 | 0.132 | 0.029 | 6.41E-06 | 0.000 | 0.045 | 0.994 | 0.002 | | 34.691 |  |
|  |  | rs114731706 | T | G | 0.032 | 0.182 | 0.038 | 2.17E-06 | 0.076 | 0.060 | 0.208 | 0.002 | | 37.860 |  |
|  |  | rs2051957 | C | T | 0.200 | 0.084 | 0.019 | 8.90E-06 | 0.015 | 0.027 | 0.565 | 0.002 | | 41.842 |  |
|  |  | rs3017103 | G | A | 0.808 | -0.098 | 0.021 | 2.94E-06 | -0.008 | 0.027 | 0.761 | 0.003 | | 54.887 |  |
|  |  | rs73331712 | T | C | 0.041 | 0.262 | 0.055 | 4.85E-06 | 0.054 | 0.053 | 0.305 | 0.005 | | 100.616 |  |
|  |  | rs77655283 | G | A | 0.070 | 0.133 | 0.030 | 5.88E-06 | 0.015 | 0.042 | 0.720 | 0.002 | | 42.139 |  |
| ebi-a-GCST90016993 | Gut microbiota abundance (genus Erysipelatoclostridium id.11381) | rs1434153 | G | A | 0.444 | -0.068 | 0.015 | 6.85E-06 | 0.005 | 0.021 | 0.802 | 0.002 | | 42.515 |  |
|  |  | rs16936671 | C | T | 0.139 | -0.097 | 0.022 | 6.04E-06 | 0.013 | 0.031 | 0.663 | 0.002 | | 41.149 |  |
|  |  | rs17804233 | C | T | 0.527 | 0.066 | 0.014 | 4.59E-06 | 0.034 | 0.021 | 0.113 | 0.002 | | 40.269 |  |
|  |  | rs2901723 | A | C | 0.468 | -0.064 | 0.014 | 8.79E-06 | -0.030 | 0.021 | 0.163 | 0.002 | | 37.637 |  |
|  |  | rs340991 | A | G | 0.266 | -0.074 | 0.016 | 3.75E-06 | -0.008 | 0.024 | 0.740 | 0.002 | | 39.338 |  |
|  |  | rs3804326 | A | G | 0.050 | 0.141 | 0.034 | 9.85E-06 | -0.012 | 0.049 | 0.803 | 0.002 | | 34.727 |  |
|  |  | rs45480394 | T | G | 0.360 | -0.069 | 0.015 | 7.66E-06 | -0.016 | 0.022 | 0.468 | 0.002 | | 39.994 |  |
|  |  | rs4697572 | A | G | 0.199 | -0.081 | 0.016 | 7.59E-07 | -0.003 | 0.026 | 0.909 | 0.002 | | 38.541 |  |
|  |  | rs58236560 | G | T | 0.121 | -0.111 | 0.023 | 2.16E-06 | -0.003 | 0.033 | 0.938 | 0.003 | | 48.187 |  |
|  |  | rs61806970 | C | T | 0.068 | 0.143 | 0.032 | 9.09E-06 | -0.047 | 0.042 | 0.262 | 0.003 | | 47.269 |  |
|  |  | rs622418 | A | G | 0.488 | -0.067 | 0.014 | 3.68E-06 | -0.019 | 0.021 | 0.374 | 0.002 | | 40.988 |  |
|  |  | rs6474512 | C | A | 0.612 | -0.067 | 0.014 | 3.02E-06 | 0.025 | 0.022 | 0.257 | 0.002 | | 39.196 |  |
|  |  | rs710230 | C | T | 0.928 | -0.143 | 0.028 | 6.33E-07 | 0.039 | 0.041 | 0.347 | 0.003 | | 50.777 |  |
|  |  | rs7221249 | G | A | 0.469 | -0.084 | 0.014 | 4.31E-09 | -0.008 | 0.021 | 0.709 | 0.004 | | 64.660 |  |
|  |  | rs9590927 | A | G | 0.546 | 0.065 | 0.014 | 6.39E-06 | -0.004 | 0.021 | 0.867 | 0.002 | | 38.019 |  |
| ebi-a-GCST90016994 | Gut microbiota abundance (genus Erysipelotrichaceae UCG003 id.11384) | rs75949021 | TRUE | C | 0.040 | -0.170 | 0.037 | 3.58E-06 | 0.021 | 0.054 | 0.697 | 0.002 | | 40.339 |  |
| ebi-a-GCST90016995 | Gut microbiota abundance (genus Escherichia Shigella id.3504) | rs112767262 | T | C | 0.223 | 0.073 | 0.016 | 8.21E-06 | -0.030 | 0.026 | 0.247 | 0.002 | | 34.215 |  |
|  |  | rs113127095 | A | G | 0.042 | 0.151 | 0.032 | 3.33E-06 | 0.040 | 0.053 | 0.450 | 0.002 | | 34.067 |  |
|  |  | rs113513883 | A | G | 0.033 | 0.172 | 0.038 | 5.28E-06 | 0.026 | 0.059 | 0.655 | 0.002 | | 35.212 |  |
|  |  | rs1154904 | G | A | 0.507 | 0.061 | 0.013 | 3.04E-06 | -0.022 | 0.021 | 0.305 | 0.002 | | 34.555 |  |
|  |  | rs118526 | A | C | 0.700 | 0.059 | 0.014 | 8.00E-06 | 0.015 | 0.023 | 0.513 | 0.001 | | 27.278 |  |
|  |  | rs2798105 | A | G | 0.099 | -0.101 | 0.022 | 8.24E-06 | -0.039 | 0.035 | 0.268 | 0.002 | | 33.445 |  |
|  |  | rs4731451 | G | A | 0.316 | -0.061 | 0.014 | 7.47E-06 | 0.011 | 0.023 | 0.623 | 0.002 | | 29.544 |  |
|  |  | rs57024273 | T | C | 0.257 | 0.063 | 0.014 | 9.70E-06 | -0.025 | 0.024 | 0.310 | 0.001 | | 27.529 |  |
|  |  | rs592299 | T | C | 0.545 | -0.059 | 0.013 | 4.77E-06 | -0.046 | 0.021 | 0.031 | 0.002 | | 31.932 |  |
|  |  | rs73208162 | A | G | 0.032 | -0.119 | 0.025 | 2.19E-06 | -0.011 | 0.059 | 0.853 | 0.001 | | 16.373 |  |
| ebi-a-GCST90016996 | Gut microbiota abundance (genus Eubacterium brachy group id.11296) | rs112617308 | T | C | 0.093 | -0.171 | 0.036 | 2.38E-06 | 0.004 | 0.036 | 0.908 | 0.005 | | 90.334 |  |
|  |  | rs12151423 | G | A | 0.481 | -0.101 | 0.023 | 9.27E-06 | 0.038 | 0.021 | 0.073 | 0.005 | | 94.497 |  |
|  |  | rs13139592 | T | C | 0.133 | -0.146 | 0.033 | 7.97E-06 | 0.047 | 0.031 | 0.138 | 0.005 | | 90.358 |  |
|  |  | rs1384962 | G | A | 0.678 | -0.121 | 0.027 | 6.99E-06 | -0.022 | 0.023 | 0.343 | 0.006 | 117.803 | | |
|  |  | rs2913110 | T | C | 0.650 | -0.105 | 0.023 | 4.56E-06 | -0.006 | 0.022 | 0.772 | 0.005 | | 92.683 |  |
|  |  | rs4862235 | A | G | 0.545 | -0.105 | 0.023 | 3.73E-06 | -0.020 | 0.021 | 0.347 | 0.005 | 100.435 | | |
|  |  | rs62348779 | T | C | 0.078 | -0.201 | 0.043 | 3.78E-06 | 0.005 | 0.040 | 0.904 | 0.006 | | 107.268 |  |
|  |  | rs6591893 | A | G | 0.652 | -0.108 | 0.024 | 7.34E-06 | -0.014 | 0.022 | 0.521 | 0.005 | 97.950 | | |
|  |  | rs720439 | G | A | 0.753 | 0.112 | 0.025 | 7.03E-06 | 0.000 | 0.025 | 0.986 | 0.005 | | 85.856 |  |
|  |  | rs73199919 | T | C | 0.049 | -0.237 | 0.053 | 8.16E-06 | 0.019 | 0.049 | 0.700 | 0.005 | 96.926 | | |
| ebi-a-GCST90016997 | Gut microbiota abundance (genus Eubacterium coprostanoligenes group id.11375) | rs1020520 | T | G | 0.152 | -0.059 | 0.013 | 8.89E-06 | 0.002 | 0.030 | 0.955 | 0.001 | | 16.466 |  |
|  |  | rs10444197 | A | G | 0.348 | -0.051 | 0.011 | 5.98E-06 | 0.007 | 0.022 | 0.763 | 0.001 | 21.311 | | |
|  |  | rs11052069 | C | T | 0.559 | -0.048 | 0.011 | 9.38E-06 | 0.017 | 0.021 | 0.417 | 0.001 | | 20.667 |  |
|  |  | rs11720857 | C | T | 0.175 | 0.063 | 0.014 | 9.26E-06 | -0.011 | 0.028 | 0.695 | 0.001 | 21.092 | | |
|  |  | rs12906958 | C | T | 0.292 | -0.053 | 0.012 | 4.35E-06 | -0.014 | 0.023 | 0.542 | 0.001 | | 21.583 |  |
|  |  | rs17159861 | C | T | 0.108 | 0.096 | 0.017 | 1.04E-08 | -0.008 | 0.035 | 0.827 | 0.002 | 32.638 | | |
|  |  | rs2644213 | A | G | 0.694 | -0.054 | 0.012 | 9.86E-06 | -0.017 | 0.023 | 0.461 | 0.001 | | 22.638 |  |
|  |  | rs4076415 | G | T | 0.631 | -0.052 | 0.011 | 1.99E-06 | -0.006 | 0.022 | 0.788 | 0.001 | 22.701 | | |
|  |  | rs62024432 | C | T | 0.096 | -0.077 | 0.017 | 7.50E-06 | -0.055 | 0.036 | 0.125 | 0.001 | | 18.930 |  |
|  |  | rs6762473 | A | C | 0.660 | -0.052 | 0.011 | 4.26E-06 | 0.004 | 0.022 | 0.873 | 0.001 | 22.421 | | |
|  |  | rs76898927 | G | A | 0.054 | 0.123 | 0.027 | 4.79E-06 | 0.089 | 0.047 | 0.057 | 0.002 | | 28.295 |  |
|  |  | rs79895140 | T | C | 0.115 | -0.064 | 0.014 | 8.62E-06 | 0.027 | 0.034 | 0.422 | 0.001 | 15.362 | | |
|  |  | rs9648214 | T | C | 0.086 | -0.083 | 0.016 | 2.52E-07 | -0.067 | 0.038 | 0.075 | 0.001 | | 19.916 |  |
| ebi-a-GCST90016998 | Gut microbiota abundance (genus Eubacterium eligens group id.14372) | rs182318 | G | A | 0.080 | -0.082 | 0.020 | 8.40E-06 | -0.083 | 0.039 | 0.033 | 0.001 | | 18.462 |  |
|  |  | rs2200429 | A | G | 0.098 | -0.089 | 0.020 | 5.30E-06 | 0.024 | 0.036 | 0.494 | 0.001 | 25.718 | | |
|  |  | rs265534 | T | G | 0.468 | -0.056 | 0.012 | 2.27E-06 | -0.008 | 0.021 | 0.702 | 0.002 | | 29.068 |  |
|  |  | rs4583233 | A | C | 0.288 | 0.067 | 0.013 | 2.84E-07 | 0.001 | 0.024 | 0.985 | 0.002 | 33.836 | | |
|  |  | rs56080211 | T | C | 0.922 | -0.123 | 0.028 | 9.14E-06 | 0.017 | 0.040 | 0.681 | 0.002 | | 40.005 |  |
|  |  | rs6923695 | T | G | 0.067 | 0.103 | 0.023 | 4.87E-06 | -0.029 | 0.042 | 0.492 | 0.001 | 24.329 | | |
| ebi-a-GCST90016999 | Gut microbiota abundance (genus Eubacterium fissicatena group id.14373) | rs10147907 | T | G | 0.079 | 0.172 | 0.040 | 8.27E-06 | 0.062 | 0.040 | 0.119 | 0.004 | | 79.429 |  |
|  |  | rs11818408 | G | A | 0.396 | 0.106 | 0.024 | 8.20E-06 | -0.019 | 0.022 | 0.378 | 0.005 | 98.808 | | |
|  |  | rs11876297 | T | C | 0.284 | 0.131 | 0.028 | 2.67E-06 | -0.023 | 0.024 | 0.347 | 0.007 | | 129.759 |  |
|  |  | rs151257695 | A | G | 0.071 | 0.210 | 0.045 | 3.10E-06 | 0.009 | 0.041 | 0.837 | 0.006 | 106.846 | | |
|  |  | rs1768152 | C | T | 0.895 | -0.139 | 0.032 | 8.70E-06 | 0.011 | 0.035 | 0.759 | 0.004 | | 67.478 |  |
|  |  | rs2733072 | G | A | 0.503 | 0.110 | 0.023 | 1.49E-06 | 0.003 | 0.021 | 0.873 | 0.006 | 110.891 | | |
|  |  | rs3771393 | T | C | 0.806 | -0.131 | 0.027 | 7.38E-07 | 0.003 | 0.027 | 0.899 | 0.005 | | 98.745 |  |
|  |  | rs6934739 | A | G | 0.333 | 0.111 | 0.025 | 9.75E-06 | -0.004 | 0.022 | 0.868 | 0.006 | 101.708 | | |
|  |  | rs7104872 | G | A | 0.110 | 0.139 | 0.029 | 2.73E-06 | -0.019 | 0.034 | 0.578 | 0.004 | | 69.469 |  |
| ebi-a-GCST90017000 | Gut microbiota abundance (genus Eubacterium hallii group id.11338) | rs10501370 | C | T | 0.063 | -0.116 | 0.025 | 5.42E-06 | -0.028 | 0.044 | 0.521 | 0.002 | 28.856 | | |
|  |  | rs10798999 | C | T | 0.268 | 0.060 | 0.013 | 2.61E-06 | 0.037 | 0.024 | 0.124 | 0.001 | | 26.101 |  |
|  |  | rs10808115 | C | A | 0.512 | 0.050 | 0.011 | 4.42E-06 | -0.046 | 0.021 | 0.029 | 0.001 | 23.377 | | |
|  |  | rs117748144 | T | C | 0.051 | -0.127 | 0.029 | 7.86E-06 | -0.025 | 0.048 | 0.598 | 0.002 | | 28.365 |  |
|  |  | rs13116360 | T | C | 0.067 | 0.154 | 0.030 | 2.94E-07 | -0.028 | 0.043 | 0.520 | 0.003 | 54.478 | | |
|  |  | rs138531890 | A | G | 0.079 | 0.153 | 0.035 | 5.43E-06 | 0.053 | 0.040 | 0.182 | 0.003 | | 62.852 |  |
|  |  | rs17074066 | T | C | 0.020 | -0.081 | 0.019 | 9.35E-06 | 0.079 | 0.074 | 0.282 | 0.000 | | 4.876 |  |
|  |  | rs17474256 | G | A | 0.098 | 0.081 | 0.018 | 9.45E-06 | -0.020 | 0.036 | 0.577 | 0.001 | 21.315 | | |
|  |  | rs281379 | A | G | 0.409 | -0.050 | 0.011 | 9.33E-06 | 0.009 | 0.022 | 0.671 | 0.001 | | 22.141 |  |
|  |  | rs28584818 | A | G | 0.080 | 0.126 | 0.027 | 4.43E-06 | 0.069 | 0.039 | 0.081 | 0.002 | 43.034 | | |
|  |  | rs60254196 | G | A | 0.535 | 0.052 | 0.011 | 2.70E-06 | 0.013 | 0.021 | 0.541 | 0.001 | | 24.974 |  |
|  |  | rs630939 | C | T | 0.451 | -0.051 | 0.011 | 9.16E-06 | -0.027 | 0.021 | 0.200 | 0.001 | 23.553 | | |
|  |  | rs6550770 | C | T | 0.958 | 0.198 | 0.044 | 4.82E-06 | 0.002 | 0.053 | 0.970 | 0.003 | | 58.353 |  |
|  |  | rs74018587 | C | T | 0.040 | 0.209 | 0.044 | 3.70E-06 | 0.079 | 0.054 | 0.141 | 0.003 | 62.418 | | |
|  |  | rs78056098 | G | T | 0.355 | -0.051 | 0.011 | 8.29E-06 | 0.015 | 0.022 | 0.509 | 0.001 | | 21.653 |  |
|  |  | rs949971 | T | G | 0.325 | -0.054 | 0.012 | 3.29E-06 | 0.004 | 0.023 | 0.866 | 0.001 | 23.502 | | |
| ebi-a-GCST90017001 | Gut microbiota abundance (genus Eubacterium nodatum group id.11297) | rs10263623 | C | T | 0.039 | 0.193 | 0.044 | 8.91E-06 | -0.065 | 0.054 | 0.227 | 0.003 | | 51.737 |  |
|  |  | rs10458299 | T | C | 0.074 | -0.188 | 0.042 | 8.37E-06 | 0.045 | 0.040 | 0.261 | 0.005 | 89.456 | | |
|  |  | rs11006576 | A | G | 0.541 | -0.110 | 0.025 | 7.99E-06 | 0.003 | 0.021 | 0.909 | 0.006 | | 111.222 |  |
|  |  | rs113893692 | C | T | 0.124 | -0.185 | 0.040 | 5.76E-06 | -0.136 | 0.033 | 0.000 | 0.007 | 137.146 | | |
|  |  | rs34297067 | A | G | 0.147 | -0.187 | 0.034 | 6.60E-08 | -0.018 | 0.030 | 0.549 | 0.009 | | 162.353 |  |
|  |  | rs61841040 | G | T | 0.197 | 0.161 | 0.034 | 3.56E-06 | 0.018 | 0.027 | 0.498 | 0.008 | 150.738 | | |
|  |  | rs6818880 | G | A | 0.533 | 0.110 | 0.025 | 7.83E-06 | 0.045 | 0.021 | 0.036 | 0.006 | | 111.284 |  |
|  |  | rs77910827 | C | T | 0.108 | 0.202 | 0.041 | 9.05E-07 | 0.033 | 0.034 | 0.340 | 0.008 | 145.242 | | |
|  |  | rs7827125 | C | T | 0.280 | 0.122 | 0.027 | 7.17E-06 | -0.009 | 0.024 | 0.703 | 0.006 | | 111.337 |  |
|  |  | rs7880204 | T | C | 0.246 | -0.125 | 0.028 | 6.84E-06 | 0.018 | 0.025 | 0.454 | 0.006 | 107.637 | | |
|  |  | rs9425984 | T | C | 0.218 | -0.130 | 0.029 | 7.21E-06 | 0.003 | 0.026 | 0.901 | 0.006 | | 106.734 |  |
| ebi-a-GCST90017002 | Gut microbiota abundance (genus Eubacterium oxidoreducens group id.11339) | rs12129908 | A | C | 0.602 | -0.089 | 0.020 | 5.80E-06 | -0.034 | 0.022 | 0.119 | 0.004 | | 70.387 |  |
|  |  | rs12423772 | G | T | 0.151 | 0.141 | 0.030 | 2.63E-06 | 0.057 | 0.031 | 0.061 | 0.005 | 93.929 | | |
|  |  | rs2973294 | G | T | 0.414 | 0.092 | 0.020 | 2.39E-06 | -0.021 | 0.021 | 0.322 | 0.004 | | 76.226 |  |
|  |  | rs34561138 | G | A | 0.039 | 0.216 | 0.046 | 2.51E-06 | -0.038 | 0.055 | 0.492 | 0.003 | 64.190 | | |
|  |  | rs440215 | T | C | 0.560 | -0.093 | 0.020 | 1.65E-06 | -0.048 | 0.021 | 0.024 | 0.004 | | 78.957 |  |
| ebi-a-GCST90017003 | Gut microbiota abundance (genus Eubacterium rectale group id.14374) | rs10248854 | C | A | 0.397 | -0.053 | 0.011 | 4.21E-06 | 0.014 | 0.022 | 0.520 | 0.001 | 24.485 | | |
|  |  | rs10797540 | A | G | 0.444 | 0.050 | 0.011 | 3.53E-06 | -0.018 | 0.021 | 0.406 | 0.001 | | 22.954 |  |
|  |  | rs143694765 | T | C | 0.103 | 0.087 | 0.020 | 9.75E-06 | 0.113 | 0.035 | 0.001 | 0.001 | 25.619 | | |
|  |  | rs2884897 | A | G | 0.035 | -0.129 | 0.029 | 6.44E-06 | -0.006 | 0.058 | 0.914 | 0.001 | | 20.811 |  |
|  |  | rs314726 | T | C | 0.496 | 0.053 | 0.011 | 1.38E-06 | -0.026 | 0.021 | 0.225 | 0.001 | 25.676 | | |
|  |  | rs35398954 | A | G | 0.161 | -0.090 | 0.017 | 5.40E-07 | 0.021 | 0.029 | 0.460 | 0.002 | | 40.322 |  |
|  |  | rs59427698 | A | G | 0.190 | -0.058 | 0.013 | 5.37E-06 | -0.026 | 0.027 | 0.330 | 0.001 | 18.718 | | |
|  |  | rs62547233 | A | G | 0.285 | 0.054 | 0.012 | 9.90E-06 | -0.003 | 0.024 | 0.895 | 0.001 | | 21.500 |  |
| ebi-a-GCST90017004 | Gut microbiota abundance (genus Eubacterium ruminantium group id.11340) | rs10131724 | C | A | 0.900 | 0.200 | 0.041 | 2.39E-06 | 0.041 | 0.036 | 0.257 | 0.007 | 132.409 | | |
|  |  | rs10923018 | G | A | 0.578 | 0.073 | 0.016 | 6.80E-06 | 0.007 | 0.021 | 0.752 | 0.003 | | 47.324 |  |
|  |  | rs11637981 | T | G | 0.443 | 0.073 | 0.016 | 5.44E-06 | -0.030 | 0.021 | 0.155 | 0.003 | 48.697 | | |
|  |  | rs13025464 | C | T | 0.597 | 0.074 | 0.016 | 6.97E-06 | -0.011 | 0.022 | 0.616 | 0.003 | | 48.072 |  |
|  |  | rs139749 | C | T | 0.342 | -0.085 | 0.017 | 8.59E-07 | -0.017 | 0.022 | 0.441 | 0.003 | 59.183 | | |
|  |  | rs16891896 | G | A | 0.085 | -0.175 | 0.039 | 2.38E-06 | 0.001 | 0.038 | 0.970 | 0.005 | | 87.345 |  |
|  |  | rs17519472 | C | T | 0.140 | 0.108 | 0.023 | 4.70E-06 | 0.044 | 0.031 | 0.155 | 0.003 | 51.401 | | |
|  |  | rs209813 | G | A | 0.145 | -0.103 | 0.024 | 9.23E-06 | 0.004 | 0.030 | 0.908 | 0.003 | | 48.854 |  |
|  |  | rs2116427 | A | G | 0.255 | 0.091 | 0.018 | 4.67E-07 | 0.019 | 0.024 | 0.427 | 0.003 | | 58.112 |  |
|  |  | rs2229917 | A | G | 0.042 | 0.154 | 0.032 | 2.16E-06 | -0.072 | 0.052 | 0.168 | 0.002 | 35.013 | | |
|  |  | rs2418654 | C | T | 0.432 | -0.075 | 0.017 | 6.17E-06 | -0.010 | 0.022 | 0.639 | 0.003 | | 50.601 |  |
|  |  | rs2817174 | C | T | 0.395 | -0.073 | 0.016 | 7.87E-06 | -0.035 | 0.022 | 0.106 | 0.003 | 47.398 | | |
|  |  | rs57340348 | T | C | 0.202 | -0.098 | 0.021 | 4.93E-06 | -0.040 | 0.027 | 0.137 | 0.003 | | 56.804 |  |
|  |  | rs606117 | G | A | 0.730 | -0.083 | 0.018 | 4.82E-06 | -0.008 | 0.024 | 0.731 | 0.003 | 50.351 | | |
|  |  | rs6676699 | T | G | 0.698 | 0.089 | 0.020 | 6.38E-06 | -0.013 | 0.023 | 0.582 | 0.003 | | 61.196 |  |
|  |  | rs7000472 | G | A | 0.584 | 0.076 | 0.017 | 4.07E-06 | 0.033 | 0.022 | 0.132 | 0.003 | 51.932 | | |
|  |  | rs72836424 | C | T | 0.111 | -0.140 | 0.030 | 2.62E-06 | -0.011 | 0.034 | 0.743 | 0.004 | | 70.976 |  |
|  |  | rs73139629 | A | C | 0.092 | -0.115 | 0.025 | 5.36E-06 | 0.046 | 0.036 | 0.207 | 0.002 | 40.769 | | |
| ebi-a-GCST90017005 | Gut microbiota abundance (genus Eubacterium ventriosum group id.11341) | rs11617697 | A | G | 0.056 | -0.143 | 0.029 | 7.22E-07 | -0.012 | 0.047 | 0.795 | 0.002 | | 39.760 |  |
|  |  | rs12964517 | G | A | 0.283 | 0.059 | 0.012 | 2.08E-06 | 0.007 | 0.024 | 0.768 | 0.001 | 25.668 | | |
|  |  | rs13082419 | C | T | 0.110 | -0.072 | 0.016 | 9.56E-06 | -0.025 | 0.034 | 0.458 | 0.001 | | 18.401 |  |
|  |  | rs16884680 | G | T | 0.104 | -0.091 | 0.019 | 1.74E-06 | -0.013 | 0.035 | 0.708 | 0.002 | 28.218 | | |
|  |  | rs35179274 | C | T | 0.179 | -0.063 | 0.014 | 5.76E-06 | 0.008 | 0.027 | 0.785 | 0.001 | | 21.222 |  |
|  |  | rs3809430 | T | C | 0.306 | -0.055 | 0.012 | 3.55E-06 | 0.008 | 0.023 | 0.740 | 0.001 | 23.451 | | |
|  |  | rs57199565 | T | C | 0.200 | 0.078 | 0.016 | 7.97E-07 | -0.021 | 0.028 | 0.450 | 0.002 | | 36.030 |  |
|  |  | rs66746423 | C | T | 0.152 | 0.075 | 0.016 | 6.11E-06 | 0.034 | 0.029 | 0.248 | 0.001 | 26.708 | | |
|  |  | rs6704822 | G | A | 0.870 | -0.074 | 0.017 | 6.62E-06 | 0.023 | 0.031 | 0.462 | 0.001 | | 22.752 |  |
|  |  | rs72783037 | C | A | 0.206 | 0.066 | 0.014 | 6.55E-06 | 0.009 | 0.026 | 0.731 | 0.001 | 26.121 | | |
|  |  | rs73615400 | T | C | 0.097 | -0.096 | 0.019 | 9.54E-07 | -0.047 | 0.036 | 0.190 | 0.002 | | 29.328 |  |
|  |  | rs73849225 | T | C | 0.082 | 0.098 | 0.022 | 5.21E-06 | 0.040 | 0.038 | 0.297 | 0.001 | | 26.383 |  |
|  |  | rs78250280 | G | A | 0.142 | 0.075 | 0.016 | 3.36E-06 | 0.004 | 0.030 | 0.908 | 0.001 | 25.139 | | |
|  |  | rs876734 | T | C | 0.717 | 0.062 | 0.013 | 2.89E-06 | 0.019 | 0.024 | 0.420 | 0.002 | | 28.525 |  |
|  |  | rs9316536 | T | G | 0.145 | -0.082 | 0.018 | 7.84E-06 | -0.012 | 0.030 | 0.703 | 0.002 | 30.321 | | |
| ebi-a-GCST90017006 | Gut microbiota abundance (genus Eubacterium xylanophilum group id.14375) | rs10140184 | A | C | 0.474 | 0.058 | 0.013 | 4.96E-06 | 0.007 | 0.022 | 0.764 | 0.002 | | 30.470 |  |
|  |  | rs10917203 | A | C | 0.393 | 0.061 | 0.013 | 3.15E-06 | 0.056 | 0.022 | 0.009 | 0.002 | 32.905 | | |
|  |  | rs112176119 | C | T | 0.092 | -0.113 | 0.025 | 3.33E-06 | -0.055 | 0.037 | 0.137 | 0.002 | | 39.708 |  |
|  |  | rs13239072 | G | A | 0.276 | 0.069 | 0.014 | 1.82E-06 | 0.024 | 0.024 | 0.320 | 0.002 | 34.675 | | |
|  |  | rs17830032 | G | A | 0.081 | -0.161 | 0.031 | 2.39E-07 | -0.002 | 0.039 | 0.969 | 0.004 | | 71.044 |  |
|  |  | rs1999224 | G | T | 0.098 | -0.095 | 0.020 | 3.75E-06 | -0.036 | 0.035 | 0.311 | 0.002 | 29.114 | | |
|  |  | rs2012708 | G | A | 0.656 | -0.057 | 0.013 | 6.53E-06 | 0.018 | 0.022 | 0.422 | 0.001 | | 27.226 |  |
|  |  | rs2213117 | T | G | 0.162 | 0.088 | 0.019 | 4.21E-06 | -0.002 | 0.029 | 0.942 | 0.002 | 38.502 | | |
|  |  | rs75586835 | A | G | 0.071 | -0.114 | 0.026 | 9.39E-06 | -0.032 | 0.042 | 0.441 | 0.002 | | 31.636 |  |
| ebi-a-GCST90017007 | Gut microbiota abundance (genus Faecalibacterium id.2057) | rs10927394 | G | T | 0.019 | -0.232 | 0.051 | 7.02E-06 | 0.147 | 0.076 | 0.054 | 0.002 | 37.544 | | |
|  |  | rs114946999 | C | T | 0.126 | -0.086 | 0.019 | 5.70E-06 | -0.020 | 0.032 | 0.538 | 0.002 | | 30.050 |  |
|  |  | rs11776390 | T | C | 0.067 | -0.078 | 0.017 | 6.40E-06 | 0.028 | 0.043 | 0.512 | 0.001 | 13.992 | | |
|  |  | rs1271565 | C | T | 0.261 | -0.058 | 0.012 | 1.30E-06 | -0.047 | 0.024 | 0.050 | 0.001 | | 23.538 |  |
|  |  | rs12753492 | A | C | 0.113 | 0.064 | 0.015 | 8.80E-06 | 0.036 | 0.034 | 0.285 | 0.001 | 15.084 | | |
|  |  | rs2835874 | T | C | 0.035 | -0.087 | 0.020 | 7.54E-06 | 0.082 | 0.056 | 0.145 | 0.001 | | 9.323 |  |
|  |  | rs6910935 | G | A | 0.940 | -0.135 | 0.028 | 1.38E-06 | -0.068 | 0.044 | 0.126 | 0.002 | 37.818 | | |
|  |  | rs75499067 | C | T | 0.075 | 0.228 | 0.047 | 1.76E-06 | 0.047 | 0.040 | 0.247 | 0.007 | | 132.937 |  |
|  |  | rs79656633 | T | C | 0.101 | 0.146 | 0.032 | 8.14E-06 | 0.014 | 0.035 | 0.701 | 0.004 | | 70.775 |  |
|  |  | rs9536330 | T | C | 0.472 | -0.048 | 0.011 | 5.33E-06 | 0.006 | 0.021 | 0.773 | 0.001 | 21.364 | | |
| ebi-a-GCST90017008 | Gut microbiota abundance (genus Family XIII AD3011 group id.11293) | rs11126423 | T | C | 0.906 | -0.090 | 0.020 | 5.91E-06 | 0.040 | 0.037 | 0.280 | 0.001 | | 25.657 |  |
|  |  | rs11736617 | G | A | 0.055 | -0.076 | 0.017 | 9.02E-06 | 0.084 | 0.046 | 0.067 | 0.001 | 11.089 | | |
|  |  | rs12812672 | T | C | 0.072 | -0.096 | 0.021 | 2.56E-06 | -0.033 | 0.041 | 0.414 | 0.001 | | 22.573 |  |
|  |  | rs149302 | T | C | 0.232 | -0.065 | 0.014 | 7.48E-06 | 0.012 | 0.025 | 0.623 | 0.001 | 27.313 | | |
|  |  | rs16840310 | G | A | 0.569 | 0.061 | 0.012 | 6.75E-07 | 0.004 | 0.022 | 0.841 | 0.002 | | 33.319 |  |
|  |  | rs16940167 | C | T | 0.191 | 0.073 | 0.016 | 3.91E-06 | 0.009 | 0.027 | 0.737 | 0.002 | 30.450 | | |
|  |  | rs17156849 | G | A | 0.059 | -0.113 | 0.025 | 4.19E-06 | 0.082 | 0.044 | 0.064 | 0.001 | | 26.136 |  |
|  |  | rs62029761 | A | G | 0.058 | 0.129 | 0.028 | 3.89E-06 | -0.034 | 0.047 | 0.478 | 0.002 | 33.295 | | |
|  |  | rs62200412 | C | T | 0.262 | -0.080 | 0.016 | 5.80E-07 | 0.030 | 0.025 | 0.214 | 0.002 | | 45.618 |  |
|  |  | rs72730932 | C | A | 0.094 | -0.090 | 0.018 | 6.89E-07 | 0.005 | 0.036 | 0.883 | 0.001 | 25.259 | | |
|  |  | rs739451 | C | T | 0.208 | 0.065 | 0.015 | 7.88E-06 | 0.020 | 0.026 | 0.439 | 0.001 | | 25.530 |  |
|  |  | rs9276029 | A | G | 0.195 | -0.081 | 0.019 | 8.93E-06 | -0.061 | 0.029 | 0.036 | 0.002 | 38.040 | | |
|  |  | rs9837139 | A | G | 0.085 | 0.108 | 0.024 | 8.71E-06 | 0.006 | 0.038 | 0.879 | 0.002 | | 32.954 |  |
| ebi-a-GCST90017009 | Gut microbiota abundance (genus Family XIII UCG001 id.11294) | rs112362903 | A | G | 0.034 | -0.149 | 0.033 | 7.88E-06 | -0.022 | 0.057 | 0.699 | 0.001 | 27.005 | | |
|  |  | rs12049454 | T | C | 0.395 | -0.065 | 0.013 | 1.17E-06 | 0.004 | 0.022 | 0.865 | 0.002 | | 36.781 |  |
|  |  | rs1426266 | C | T | 0.728 | 0.067 | 0.014 | 1.25E-06 | -0.012 | 0.024 | 0.612 | 0.002 | 32.253 | | |
|  |  | rs3842897 | G | A | 0.089 | -0.113 | 0.024 | 5.20E-06 | 0.025 | 0.038 | 0.515 | 0.002 | | 37.891 |  |
|  |  | rs62414802 | C | T | 0.249 | -0.061 | 0.013 | 4.29E-06 | 0.025 | 0.024 | 0.313 | 0.001 | 25.736 | | |
|  |  | rs7119679 | G | A | 0.243 | -0.081 | 0.017 | 3.52E-06 | -0.009 | 0.025 | 0.726 | 0.002 | | 44.259 |  |
|  |  | rs76463770 | A | G | 0.030 | 0.193 | 0.042 | 3.77E-06 | 0.030 | 0.062 | 0.623 | 0.002 | | 39.792 |  |
|  |  | rs8076666 | G | A | 0.880 | -0.089 | 0.020 | 8.02E-06 | -0.053 | 0.033 | 0.105 | 0.002 | 30.425 | | |
| ebi-a-GCST90017010 | Gut microbiota abundance (genus Flavonifractor id.2059) | rs114873521 | C | T | 0.073 | -0.130 | 0.029 | 7.13E-06 | 0.101 | 0.041 | 0.015 | 0.002 | | 42.190 |  |
|  |  | rs11811696 | T | C | 0.082 | -0.116 | 0.024 | 2.07E-06 | 0.021 | 0.038 | 0.582 | 0.002 | 37.398 | | |
|  |  | rs12030302 | G | A | 0.528 | 0.069 | 0.014 | 5.61E-07 | 0.003 | 0.021 | 0.905 | 0.002 | | 43.908 |  |
|  |  | rs34066017 | A | G | 0.206 | 0.076 | 0.016 | 1.52E-06 | -0.035 | 0.026 | 0.180 | 0.002 | 35.059 | | |
|  |  | rs806808 | C | T | 0.559 | -0.067 | 0.014 | 1.18E-06 | 0.024 | 0.021 | 0.261 | 0.002 | | 40.343 |  |
| ebi-a-GCST90017011 | Gut microbiota abundance (genus Fusicatenibacter id.11305) | rs10439674 | A | G | 0.203 | -0.057 | 0.013 | 7.68E-06 | 0.005 | 0.026 | 0.840 | 0.001 | 19.430 | | |
|  |  | rs167879 | T | C | 0.853 | 0.066 | 0.015 | 5.87E-06 | 0.020 | 0.030 | 0.503 | 0.001 | | 20.038 |  |
|  |  | rs1864685 | A | C | 0.407 | -0.049 | 0.011 | 4.96E-06 | 0.017 | 0.022 | 0.442 | 0.001 | 21.689 | | |
|  |  | rs2025938 | G | A | 0.066 | -0.097 | 0.021 | 2.99E-06 | -0.074 | 0.043 | 0.083 | 0.001 | | 21.170 |  |
|  |  | rs206581 | A | G | 0.217 | -0.057 | 0.013 | 8.96E-06 | -0.008 | 0.026 | 0.744 | 0.001 | | 20.151 |  |
|  |  | rs2132128 | G | A | 0.103 | -0.077 | 0.016 | 1.08E-06 | 0.024 | 0.035 | 0.503 | 0.001 | | 20.178 |  |
|  |  | rs3303 | T | C | 0.065 | -0.095 | 0.020 | 3.94E-06 | 0.019 | 0.043 | 0.664 | 0.001 | | 20.149 |  |
|  |  | rs4378146 | A | C | 0.256 | -0.062 | 0.013 | 7.20E-07 | 0.001 | 0.024 | 0.985 | 0.001 | | 26.577 |  |
|  |  | rs60254196 | G | A | 0.535 | 0.049 | 0.011 | 5.47E-06 | 0.013 | 0.021 | 0.541 | 0.001 | | 22.151 |  |
|  |  | rs62187631 | T | C | 0.192 | -0.071 | 0.016 | 4.55E-06 | -0.060 | 0.027 | 0.025 | 0.002 | | 28.750 |  |
|  |  | rs62353480 | A | G | 0.176 | -0.070 | 0.015 | 1.57E-06 | 0.039 | 0.028 | 0.171 | 0.001 | | 26.169 |  |
|  |  | rs6515626 | G | A | 0.067 | 0.142 | 0.031 | 7.29E-06 | 0.037 | 0.043 | 0.391 | 0.003 | | 45.966 |  |
|  |  | rs704418 | C | T | 0.868 | -0.074 | 0.015 | 7.77E-07 | 0.080 | 0.032 | 0.012 | 0.001 | | 22.926 |  |
|  |  | rs73103914 | A | G | 0.150 | -0.060 | 0.013 | 8.30E-06 | 0.044 | 0.030 | 0.136 | 0.001 | | 16.718 |  |
|  |  | rs792108 | C | T | 0.590 | 0.051 | 0.011 | 8.50E-06 | 0.006 | 0.022 | 0.765 | 0.001 | | 22.953 |  |
|  |  | rs8028026 | G | A | 0.908 | 0.079 | 0.018 | 8.06E-06 | -0.050 | 0.036 | 0.167 | 0.001 | | 19.339 |  |
|  |  | rs8063430 | T | C | 0.050 | -0.104 | 0.022 | 4.93E-06 | -0.097 | 0.048 | 0.044 | 0.001 | | 19.018 |  |
|  |  | rs9905659 | G | A | 0.184 | -0.062 | 0.014 | 7.31E-06 | 0.017 | 0.027 | 0.540 | 0.001 | | 20.887 |  |
| ebi-a-GCST90017012 | Gut microbiota abundance (genus Gordonibacter id.821) | rs13412653 | A | C | 0.371 | 0.108 | 0.024 | 8.61E-06 | 0.001 | 0.022 | 0.972 | 0.005 | | 99.634 |  |
|  |  | rs16955299 | G | A | 0.099 | -0.196 | 0.043 | 6.37E-06 | 0.050 | 0.036 | 0.166 | 0.007 | | 126.864 |  |
|  |  | rs322296 | G | A | 0.075 | 0.179 | 0.038 | 4.02E-06 | 0.028 | 0.040 | 0.483 | 0.004 | | 81.152 |  |
|  |  | rs35042269 | C | A | 0.114 | -0.180 | 0.040 | 8.11E-06 | -0.033 | 0.033 | 0.316 | 0.007 | | 121.024 |  |
|  |  | rs3765837 | T | G | 0.072 | -0.191 | 0.043 | 7.17E-06 | 0.033 | 0.041 | 0.422 | 0.005 | | 89.234 |  |
|  |  | rs4596722 | G | A | 0.500 | -0.103 | 0.023 | 9.06E-06 | 0.017 | 0.021 | 0.419 | 0.005 | | 97.617 |  |
|  |  | rs61934597 | C | T | 0.063 | -0.172 | 0.039 | 8.37E-06 | -0.042 | 0.043 | 0.335 | 0.004 | | 64.416 |  |
|  |  | rs71545975 | A | G | 0.171 | -0.154 | 0.034 | 7.04E-06 | -0.030 | 0.028 | 0.290 | 0.007 | | 123.860 |  |
|  |  | rs72714787 | C | A | 0.132 | 0.181 | 0.038 | 1.43E-06 | -0.018 | 0.031 | 0.570 | 0.008 | | 138.884 |  |
|  |  | rs72939513 | A | G | 0.051 | -0.214 | 0.049 | 7.98E-06 | -0.015 | 0.048 | 0.751 | 0.004 | | 82.057 |  |
|  |  | rs7294633 | T | C | 0.727 | -0.129 | 0.025 | 3.44E-07 | 0.024 | 0.024 | 0.315 | 0.007 | | 121.394 |  |
|  |  | rs768830 | A | G | 0.844 | -0.150 | 0.033 | 7.76E-06 | 0.001 | 0.029 | 0.987 | 0.006 | | 109.139 |  |
| ebi-a-GCST90017013 | Gut microbiota abundance (genus Haemophilus id.3698) | rs10781340 | A | G | 0.874 | -0.095 | 0.020 | 4.32E-06 | 0.022 | 0.032 | 0.499 | 0.002 | | 36.366 |  |
|  |  | rs111582866 | G | A | 0.087 | -0.124 | 0.026 | 1.27E-06 | -0.005 | 0.038 | 0.892 | 0.002 | | 45.054 |  |
|  |  | rs35509 | G | A | 0.043 | 0.128 | 0.027 | 2.01E-06 | -0.030 | 0.052 | 0.567 | 0.001 | | 24.664 |  |
|  |  | rs4822728 | T | C | 0.483 | 0.071 | 0.015 | 3.48E-06 | 0.054 | 0.021 | 0.012 | 0.002 | | 45.746 |  |
|  |  | rs76022354 | C | T | 0.050 | 0.245 | 0.051 | 1.83E-06 | 0.000 | 0.049 | 0.997 | 0.006 | | 104.478 |  |
|  |  | rs78909003 | T | C | 0.055 | -0.246 | 0.050 | 1.67E-06 | -0.019 | 0.047 | 0.688 | 0.006 | | 115.990 |  |
|  |  | rs9328464 | T | C | 0.447 | 0.072 | 0.015 | 1.42E-06 | 0.010 | 0.021 | 0.638 | 0.003 | | 47.520 |  |
|  |  | rs9382510 | C | T | 0.265 | -0.094 | 0.017 | 7.12E-08 | 0.020 | 0.024 | 0.415 | 0.003 | | 62.677 |  |
|  |  | rs9895850 | T | C | 0.044 | -0.193 | 0.042 | 2.14E-06 | -0.054 | 0.052 | 0.300 | 0.003 | | 57.520 |  |
| ebi-a-GCST90017014 | Gut microbiota abundance (genus Holdemanella id.11393) | rs12513188 | G | A | 0.270 | 0.090 | 0.020 | 4.65E-06 | -0.021 | 0.024 | 0.377 | 0.003 | | 59.212 |  |
|  |  | rs17586763 | T | C | 0.051 | -0.227 | 0.051 | 7.72E-06 | -0.044 | 0.047 | 0.354 | 0.005 | | 92.502 |  |
|  |  | rs1926302 | G | A | 0.226 | -0.108 | 0.023 | 7.50E-06 | 0.016 | 0.026 | 0.533 | 0.004 | | 74.980 |  |
|  |  | rs34187114 | C | A | 0.111 | -0.105 | 0.023 | 5.13E-06 | -0.002 | 0.033 | 0.958 | 0.002 | | 39.580 |  |
|  |  | rs35228298 | G | A | 0.161 | 0.093 | 0.020 | 7.30E-06 | -0.027 | 0.029 | 0.357 | 0.002 | | 43.310 |  |
|  |  | rs4541991 | T | C | 0.324 | -0.093 | 0.019 | 2.10E-06 | -0.018 | 0.023 | 0.430 | 0.004 | | 69.329 |  |
|  |  | rs607782 | C | T | 0.608 | 0.085 | 0.017 | 7.19E-07 | -0.025 | 0.022 | 0.258 | 0.003 | | 64.029 |  |
|  |  | rs62113381 | T | C | 0.131 | -0.105 | 0.023 | 5.54E-06 | 0.003 | 0.031 | 0.921 | 0.003 | | 46.511 |  |
|  |  | rs73011279 | T | C | 0.233 | -0.096 | 0.020 | 1.36E-06 | -0.014 | 0.025 | 0.575 | 0.003 | | 60.798 |  |
|  |  | rs75764681 | T | C | 0.045 | -0.283 | 0.060 | 1.94E-06 | -0.110 | 0.052 | 0.035 | 0.007 | | 127.282 |  |
|  |  | rs8113760 | G | A | 0.310 | 0.079 | 0.017 | 4.62E-06 | -0.012 | 0.023 | 0.601 | 0.003 | | 49.099 |  |
| ebi-a-GCST90017015 | Gut microbiota abundance (genus Holdemania id.2157) | rs10885477 | T | C | 0.050 | -0.135 | 0.030 | 8.60E-06 | 0.057 | 0.048 | 0.236 | 0.002 | | 31.833 |  |
|  |  | rs11080063 | G | A | 0.405 | -0.067 | 0.015 | 6.67E-06 | -0.007 | 0.022 | 0.764 | 0.002 | | 39.195 |  |
|  |  | rs111745969 | A | G | 0.136 | 0.121 | 0.027 | 3.71E-06 | 0.031 | 0.031 | 0.325 | 0.003 | | 63.054 |  |
|  |  | rs113593397 | A | G | 0.096 | -0.129 | 0.028 | 9.36E-06 | -0.048 | 0.036 | 0.181 | 0.003 | | 53.032 |  |
|  |  | rs116500994 | G | T | 0.047 | -0.138 | 0.029 | 2.34E-06 | 0.023 | 0.050 | 0.647 | 0.002 | | 31.430 |  |
|  |  | rs12701617 | A | G | 0.465 | -0.066 | 0.015 | 9.52E-06 | -0.018 | 0.021 | 0.385 | 0.002 | | 39.903 |  |
|  |  | rs1867876 | T | C | 0.298 | 0.084 | 0.016 | 2.74E-07 | -0.021 | 0.023 | 0.370 | 0.003 | | 54.645 |  |
|  |  | rs4146507 | C | T | 0.245 | 0.079 | 0.018 | 7.23E-06 | -0.017 | 0.025 | 0.490 | 0.002 | | 42.906 |  |
|  |  | rs73139538 | G | A | 0.030 | -0.149 | 0.033 | 7.77E-06 | -0.024 | 0.062 | 0.703 | 0.001 | | 23.626 |  |
|  |  | rs77293403 | A | G | 0.035 | 0.165 | 0.034 | 1.77E-06 | -0.094 | 0.057 | 0.099 | 0.002 | | 33.818 |  |
|  |  | rs80149660 | C | T | 0.044 | -0.233 | 0.052 | 6.04E-06 | -0.010 | 0.052 | 0.854 | 0.005 | | 83.835 |  |
|  |  | rs9500080 | C | T | 0.171 | 0.093 | 0.018 | 4.09E-07 | 0.000 | 0.028 | 0.996 | 0.002 | | 44.764 |  |
|  |  | rs9529719 | C | T | 0.676 | -0.074 | 0.016 | 5.97E-06 | 0.033 | 0.023 | 0.147 | 0.002 | | 44.111 |  |
|  |  | rs967319 | T | C | 0.240 | 0.079 | 0.018 | 8.38E-06 | 0.014 | 0.025 | 0.573 | 0.002 | | 41.677 |  |
| ebi-a-GCST90017016 | Gut microbiota abundance (genus Howardella id.2000) | rs10048062 | C | T | 0.088 | -0.147 | 0.034 | 8.59E-06 | 0.009 | 0.038 | 0.802 | 0.003 | | 64.259 |  |
|  |  | rs12452946 | A | G | 0.498 | -0.106 | 0.023 | 3.80E-06 | 0.038 | 0.021 | 0.073 | 0.006 | | 103.275 |  |
|  |  | rs1484873 | G | A | 0.848 | 0.228 | 0.046 | 2.56E-06 | -0.092 | 0.030 | 0.002 | 0.013 | | 248.440 |  |
|  |  | rs17167098 | G | A | 0.136 | -0.169 | 0.035 | 1.12E-06 | -0.044 | 0.031 | 0.154 | 0.007 | | 124.769 |  |
|  |  | rs2154047 | A | C | 0.912 | 0.193 | 0.042 | 9.97E-06 | 0.011 | 0.037 | 0.764 | 0.006 | | 110.132 |  |
|  |  | rs36081916 | T | C | 0.089 | -0.181 | 0.040 | 4.70E-06 | 0.022 | 0.038 | 0.568 | 0.005 | | 98.551 |  |
|  |  | rs3791893 | A | G | 0.144 | 0.147 | 0.034 | 9.50E-06 | 0.004 | 0.030 | 0.885 | 0.005 | | 97.975 |  |
|  |  | rs609430 | T | G | 0.350 | -0.112 | 0.024 | 3.34E-06 | 0.044 | 0.022 | 0.047 | 0.006 | | 105.308 |  |
|  |  | rs672217 | G | A | 0.199 | 0.164 | 0.035 | 3.52E-06 | -0.008 | 0.027 | 0.764 | 0.009 | | 158.579 |  |
| ebi-a-GCST90017017 | Gut microbiota abundance (genus Hungatella id.11306) | rs10044993 | A | C | 0.915 | -0.140 | 0.032 | 8.07E-06 | 0.039 | 0.038 | 0.305 | 0.003 | | 55.955 |  |
|  |  | rs13128780 | T | C | 0.188 | -0.150 | 0.031 | 1.75E-06 | 0.034 | 0.027 | 0.209 | 0.007 | | 126.325 |  |
|  |  | rs13249325 | T | G | 0.469 | -0.100 | 0.023 | 9.69E-06 | 0.018 | 0.021 | 0.388 | 0.005 | | 91.837 |  |
|  |  | rs17092615 | G | A | 0.139 | 0.152 | 0.034 | 7.38E-06 | -0.011 | 0.031 | 0.724 | 0.006 | | 102.355 |  |
|  |  | rs72759041 | G | T | 0.209 | -0.126 | 0.028 | 3.86E-06 | 0.027 | 0.026 | 0.306 | 0.005 | | 96.738 |  |
| ebi-a-GCST90017018 | Gut microbiota abundance (genus Intestinibacter id.11345) | rs10805326 | A | G | 0.706 | -0.078 | 0.014 | 3.55E-08 | -0.041 | 0.023 | 0.078 | 0.002 | | 45.855 |  |
|  |  | rs11109097 | T | C | 0.509 | -0.062 | 0.014 | 5.49E-06 | 0.028 | 0.021 | 0.197 | 0.002 | | 35.792 |  |
|  |  | rs118030283 | G | A | 0.045 | -0.152 | 0.032 | 2.67E-06 | -0.006 | 0.051 | 0.906 | 0.002 | | 36.560 |  |
|  |  | rs16938435 | T | C | 0.097 | -0.112 | 0.024 | 1.80E-06 | -0.047 | 0.036 | 0.193 | 0.002 | | 40.343 |  |
|  |  | rs2098844 | T | C | 0.621 | 0.058 | 0.013 | 6.79E-06 | 0.019 | 0.022 | 0.389 | 0.002 | | 28.611 |  |
|  |  | rs2702387 | G | A | 0.596 | -0.061 | 0.013 | 4.26E-06 | -0.008 | 0.022 | 0.716 | 0.002 | | 32.777 |  |
|  |  | rs4327025 | G | A | 0.186 | -0.081 | 0.015 | 1.64E-07 | 0.028 | 0.027 | 0.295 | 0.002 | | 36.491 |  |
|  |  | rs447950 | A | G | 0.376 | 0.063 | 0.014 | 5.64E-06 | 0.013 | 0.022 | 0.565 | 0.002 | | 34.026 |  |
|  |  | rs478972 | C | T | 0.908 | 0.143 | 0.030 | 1.82E-06 | 0.033 | 0.037 | 0.368 | 0.003 | | 62.339 |  |
|  |  | rs6062862 | A | G | 0.083 | 0.092 | 0.020 | 6.68E-06 | 0.028 | 0.038 | 0.462 | 0.001 | | 23.885 |  |
|  |  | rs62430350 | T | C | 0.037 | 0.151 | 0.035 | 6.84E-06 | -0.003 | 0.056 | 0.959 | 0.002 | | 29.517 |  |
|  |  | rs68093214 | C | T | 0.241 | 0.066 | 0.015 | 9.26E-06 | -0.043 | 0.025 | 0.081 | 0.002 | | 29.496 |  |
|  |  | rs6875660 | C | T | 0.061 | 0.089 | 0.019 | 3.06E-06 | -0.062 | 0.044 | 0.163 | 0.001 | | 16.654 |  |
|  |  | rs893394 | G | A | 0.453 | 0.058 | 0.013 | 7.85E-06 | -0.044 | 0.021 | 0.042 | 0.002 | | 30.977 |  |
|  |  | rs9348442 | C | T | 0.126 | 0.099 | 0.022 | 6.26E-06 | -0.055 | 0.032 | 0.088 | 0.002 | | 39.818 |  |
| ebi-a-GCST90017019 | Gut microbiota abundance (genus Intestinimonas id.2062) | rs10262702 | T | C | 0.124 | 0.092 | 0.019 | 2.06E-06 | -0.020 | 0.032 | 0.529 | 0.002 | | 33.659 |  |
|  |  | rs11258178 | A | G | 0.491 | 0.066 | 0.013 | 6.98E-07 | 0.003 | 0.021 | 0.883 | 0.002 | | 40.104 |  |
|  |  | rs12226153 | A | G | 0.014 | -0.151 | 0.031 | 5.12E-07 | -0.044 | 0.088 | 0.616 | 0.001 | | 11.785 |  |
|  |  | rs17067892 | C | T | 0.087 | 0.107 | 0.025 | 6.38E-06 | -0.004 | 0.037 | 0.908 | 0.002 | | 33.682 |  |
|  |  | rs1859797 | G | A | 0.550 | 0.060 | 0.013 | 4.12E-06 | -0.051 | 0.021 | 0.017 | 0.002 | | 33.146 |  |
|  |  | rs2276760 | A | G | 0.239 | -0.069 | 0.015 | 7.84E-06 | -0.013 | 0.025 | 0.599 | 0.002 | | 31.411 |  |
|  |  | rs2731794 | C | T | 0.034 | 0.121 | 0.026 | 1.92E-06 | 0.054 | 0.058 | 0.349 | 0.001 | | 17.631 |  |
|  |  | rs2930225 | T | G | 0.767 | -0.073 | 0.015 | 1.35E-06 | 0.025 | 0.025 | 0.312 | 0.002 | | 34.939 |  |
|  |  | rs4113676 | A | C | 0.015 | -0.219 | 0.049 | 7.42E-06 | 0.087 | 0.088 | 0.325 | 0.001 | | 25.585 |  |
|  |  | rs4784055 | T | C | 0.050 | -0.175 | 0.039 | 8.72E-07 | 0.101 | 0.048 | 0.037 | 0.003 | | 53.880 |  |
|  |  | rs62240188 | G | A | 0.094 | 0.130 | 0.027 | 2.20E-06 | 0.028 | 0.036 | 0.442 | 0.003 | | 52.792 |  |
|  |  | rs6934519 | C | T | 0.257 | 0.069 | 0.015 | 8.57E-06 | -0.010 | 0.024 | 0.675 | 0.002 | | 33.668 |  |
|  |  | rs716604 | A | G | 0.229 | 0.082 | 0.017 | 8.57E-07 | -0.017 | 0.025 | 0.511 | 0.002 | | 43.479 |  |
|  |  | rs7170984 | T | C | 0.282 | -0.066 | 0.014 | 2.98E-06 | -0.016 | 0.024 | 0.492 | 0.002 | | 32.248 |  |
|  |  | rs72982915 | C | T | 0.056 | 0.183 | 0.040 | 4.91E-06 | -0.039 | 0.046 | 0.396 | 0.004 | | 65.757 |  |
|  |  | rs9823439 | C | T | 0.538 | 0.058 | 0.013 | 9.86E-06 | -0.003 | 0.021 | 0.901 | 0.002 | | 30.922 |  |
| ebi-a-GCST90017020 | Gut microbiota abundance (genus Lachnoclostridium id.11308) | rs1031599 | T | G | 0.936 | 0.079 | 0.018 | 6.31E-06 | 0.045 | 0.043 | 0.301 | 0.001 | | 13.612 |  |
|  |  | rs12566975 | T | C | 0.452 | -0.047 | 0.011 | 9.57E-06 | 0.009 | 0.021 | 0.662 | 0.001 | | 19.930 |  |
|  |  | rs1528479 | A | G | 0.625 | 0.050 | 0.011 | 9.64E-06 | 0.007 | 0.022 | 0.754 | 0.001 | | 21.330 |  |
|  |  | rs1997204 | C | T | 0.955 | 0.108 | 0.024 | 5.97E-06 | -0.029 | 0.051 | 0.564 | 0.001 | | 18.350 |  |
|  |  | rs2385421 | A | G | 0.119 | 0.075 | 0.018 | 7.14E-06 | -0.010 | 0.033 | 0.765 | 0.001 | | 21.465 |  |
|  |  | rs3821998 | C | A | 0.105 | -0.086 | 0.019 | 6.72E-06 | -0.007 | 0.035 | 0.834 | 0.001 | | 25.812 |  |
|  |  | rs4738679 | A | G | 0.615 | 0.052 | 0.011 | 4.42E-06 | 0.024 | 0.022 | 0.271 | 0.001 | | 23.540 |  |
|  |  | rs6112314 | A | C | 0.332 | -0.056 | 0.011 | 2.43E-07 | 0.008 | 0.022 | 0.730 | 0.001 | | 25.685 |  |
|  |  | rs615997 | T | C | 0.510 | 0.051 | 0.011 | 2.03E-06 | -0.038 | 0.021 | 0.069 | 0.001 | | 24.034 |  |
|  |  | rs62285313 | A | G | 0.098 | 0.086 | 0.018 | 1.58E-06 | -0.064 | 0.036 | 0.070 | 0.001 | | 24.251 |  |
|  |  | rs72829893 | G | T | 0.103 | 0.117 | 0.027 | 5.58E-06 | 0.002 | 0.035 | 0.959 | 0.003 | | 46.799 |  |
|  |  | rs78068103 | A | G | 0.116 | 0.089 | 0.019 | 3.67E-06 | -0.040 | 0.033 | 0.225 | 0.002 | | 29.673 |  |
|  |  | rs789029 | C | T | 0.140 | -0.064 | 0.014 | 3.75E-06 | 0.062 | 0.030 | 0.039 | 0.001 | | 18.221 |  |
| ebi-a-GCST90017021 | Gut microbiota abundance (genus Lachnospiraceae FCS020 group id.11314) | rs10093861 | G | A | 0.407 | -0.057 | 0.012 | 3.06E-06 | -0.023 | 0.022 | 0.291 | 0.002 | | 28.699 |  |
|  |  | rs1254846 | A | G | 0.856 | -0.106 | 0.023 | 5.60E-06 | 0.018 | 0.032 | 0.561 | 0.003 | | 50.960 |  |
|  |  | rs1363769 | C | T | 0.968 | 0.201 | 0.045 | 1.58E-06 | 0.023 | 0.059 | 0.704 | 0.002 | | 45.843 |  |
|  |  | rs2322265 | C | T | 0.265 | -0.067 | 0.014 | 5.21E-06 | 0.031 | 0.024 | 0.204 | 0.002 | | 31.785 |  |
|  |  | rs2862811 | C | T | 0.699 | -0.056 | 0.012 | 3.92E-06 | 0.013 | 0.023 | 0.571 | 0.001 | | 24.660 |  |
|  |  | rs35035870 | T | C | 0.039 | -0.191 | 0.041 | 2.62E-06 | -0.165 | 0.054 | 0.002 | 0.003 | | 50.352 |  |
|  |  | rs3999074 | G | T | 0.494 | -0.055 | 0.012 | 6.55E-06 | -0.002 | 0.021 | 0.936 | 0.002 | | 27.834 |  |
|  |  | rs4452603 | T | G | 0.272 | 0.060 | 0.014 | 8.98E-06 | -0.015 | 0.024 | 0.544 | 0.001 | | 26.576 |  |
|  |  | rs7249113 | G | A | 0.299 | 0.068 | 0.013 | 3.72E-07 | 0.001 | 0.023 | 0.965 | 0.002 | | 35.547 |  |
|  |  | rs72793667 | A | G | 0.040 | -0.117 | 0.025 | 1.63E-06 | -0.019 | 0.054 | 0.728 | 0.001 | | 19.417 |  |
|  |  | rs9308097 | G | A | 0.507 | -0.055 | 0.012 | 7.47E-06 | 0.008 | 0.021 | 0.698 | 0.002 | | 28.160 |  |
|  |  | rs9788306 | C | T | 0.288 | -0.063 | 0.013 | 1.39E-06 | 0.017 | 0.023 | 0.463 | 0.002 | | 29.694 |  |
| ebi-a-GCST90017022 | Gut microbiota abundance (genus Lachnospiraceae NC2004 group id.11316) | rs117467633 | T | C | 0.040 | -0.170 | 0.038 | 9.13E-06 | -0.049 | 0.054 | 0.367 | 0.002 | | 40.778 |  |
|  |  | rs12127733 | G | A | 0.179 | 0.115 | 0.025 | 3.11E-06 | 0.010 | 0.028 | 0.710 | 0.004 | | 71.724 |  |
|  |  | rs12208226 | C | A | 0.106 | -0.155 | 0.034 | 9.75E-06 | -0.013 | 0.035 | 0.703 | 0.005 | | 83.878 |  |
|  |  | rs12863463 | G | A | 0.078 | -0.156 | 0.035 | 6.04E-06 | 0.007 | 0.039 | 0.856 | 0.004 | | 64.679 |  |
|  |  | rs17067076 | G | A | 0.115 | -0.155 | 0.035 | 5.61E-06 | -0.054 | 0.033 | 0.103 | 0.005 | | 89.893 |  |
|  |  | rs1928659 | T | C | 0.203 | 0.103 | 0.023 | 6.17E-06 | -0.069 | 0.026 | 0.009 | 0.003 | | 62.536 |  |
|  |  | rs1929743 | T | C | 0.336 | 0.084 | 0.019 | 9.06E-06 | -0.026 | 0.022 | 0.252 | 0.003 | | 57.567 |  |
|  |  | rs3756315 | A | G | 0.297 | -0.088 | 0.019 | 3.33E-06 | 0.013 | 0.023 | 0.565 | 0.003 | | 59.918 |  |
|  |  | rs6116753 | G | A | 0.178 | 0.099 | 0.021 | 2.92E-06 | 0.013 | 0.028 | 0.626 | 0.003 | | 53.208 |  |
| ebi-a-GCST90017023 | Gut microbiota abundance (genus Lachnospiraceae ND3007 group id.11317) | rs2861203 | G | A | 0.300 | 0.057 | 0.013 | 7.37E-06 | -0.013 | 0.023 | 0.576 | 0.001 | | 25.271 |  |
|  |  | rs72776675 | T | C | 0.172 | -0.065 | 0.015 | 8.72E-06 | 0.011 | 0.028 | 0.707 | 0.001 | | 21.875 |  |
|  |  | rs9932954 | A | G | 0.335 | -0.056 | 0.012 | 1.25E-06 | 0.010 | 0.022 | 0.666 | 0.001 | | 25.811 |  |
| ebi-a-GCST90017024 | Gut microbiota abundance (genus Lachnospiraceae NK4A136 group id.11319) | rs10952110 | G | T | 0.445 | 0.049 | 0.011 | 9.08E-06 | -0.003 | 0.021 | 0.874 | 0.001 | | 21.567 |  |
|  |  | rs11263806 | A | G | 0.341 | -0.052 | 0.012 | 5.07E-06 | 0.026 | 0.022 | 0.237 | 0.001 | | 22.708 |  |
|  |  | rs12611395 | G | A | 0.894 | 0.090 | 0.020 | 5.83E-06 | -0.010 | 0.034 | 0.763 | 0.002 | | 28.331 |  |
|  |  | rs160061 | G | A | 0.481 | -0.051 | 0.011 | 2.12E-06 | -0.020 | 0.021 | 0.342 | 0.001 | | 24.204 |  |
|  |  | rs28540839 | A | C | 0.476 | 0.051 | 0.011 | 9.34E-06 | -0.014 | 0.021 | 0.524 | 0.001 | | 23.662 |  |
|  |  | rs2880566 | T | C | 0.149 | 0.060 | 0.013 | 5.61E-06 | 0.050 | 0.030 | 0.095 | 0.001 | | 16.706 |  |
|  |  | rs4955932 | T | C | 0.388 | -0.049 | 0.011 | 7.05E-06 | -0.011 | 0.022 | 0.603 | 0.001 | | 21.122 |  |
|  |  | rs59805249 | T | C | 0.093 | 0.094 | 0.021 | 9.45E-06 | -0.024 | 0.037 | 0.504 | 0.001 | | 27.276 |  |
|  |  | rs68104925 | T | C | 0.307 | -0.055 | 0.012 | 2.37E-06 | -0.027 | 0.023 | 0.233 | 0.001 | | 23.540 |  |
|  |  | rs7073658 | G | T | 0.514 | 0.050 | 0.011 | 5.27E-06 | -0.005 | 0.021 | 0.831 | 0.001 | | 22.899 |  |
|  |  | rs73044693 | A | G | 0.070 | -0.108 | 0.023 | 3.57E-06 | -0.026 | 0.042 | 0.530 | 0.002 | | 27.605 |  |
|  |  | rs7616165 | G | T | 0.025 | -0.231 | 0.048 | 2.77E-06 | -0.009 | 0.067 | 0.888 | 0.003 | | 47.433 |  |
|  |  | rs76193507 | A | G | 0.086 | -0.230 | 0.050 | 2.93E-06 | -0.013 | 0.038 | 0.739 | 0.008 | | 152.784 |  |
|  |  | rs7832116 | A | G | 0.129 | -0.071 | 0.015 | 3.57E-06 | 0.050 | 0.032 | 0.119 | 0.001 | | 21.049 |  |
|  |  | rs954878 | A | G | 0.372 | -0.052 | 0.011 | 1.78E-06 | -0.027 | 0.022 | 0.221 | 0.001 | | 23.248 |  |
| ebi-a-GCST90017025 | Gut microbiota abundance (genus Lachnospiraceae UCG001 id.11321) | rs12131224 | C | T | 0.109 | 0.117 | 0.026 | 7.40E-06 | -0.004 | 0.034 | 0.917 | 0.003 | | 48.929 |  |
|  |  | rs2050911 | G | A | 0.338 | 0.075 | 0.015 | 1.11E-06 | -0.029 | 0.022 | 0.199 | 0.003 | | 46.453 |  |
|  |  | rs2371284 | C | T | 0.768 | 0.076 | 0.017 | 7.56E-06 | 0.023 | 0.025 | 0.369 | 0.002 | | 38.035 |  |
|  |  | rs437876 | T | C | 0.355 | 0.078 | 0.014 | 7.17E-08 | -0.004 | 0.022 | 0.858 | 0.003 | | 51.868 |  |
|  |  | rs4981345 | T | C | 0.330 | -0.068 | 0.015 | 6.09E-06 | 0.002 | 0.023 | 0.915 | 0.002 | | 37.769 |  |
|  |  | rs573933 | T | C | 0.107 | -0.108 | 0.023 | 3.11E-06 | -0.036 | 0.034 | 0.301 | 0.002 | | 40.755 |  |
|  |  | rs62496417 | T | G | 0.215 | -0.075 | 0.017 | 5.88E-06 | 0.013 | 0.026 | 0.622 | 0.002 | | 34.734 |  |
|  |  | rs7341608 | T | C | 0.134 | -0.078 | 0.018 | 9.48E-06 | -0.005 | 0.031 | 0.870 | 0.001 | | 26.265 |  |
|  |  | rs74034332 | G | A | 0.062 | 0.168 | 0.038 | 3.33E-06 | -0.036 | 0.044 | 0.413 | 0.003 | | 60.092 |  |
|  |  | rs78848836 | A | G | 0.104 | -0.119 | 0.026 | 3.38E-06 | 0.040 | 0.035 | 0.249 | 0.003 | | 48.335 |  |
|  |  | rs8104225 | A | G | 0.228 | 0.089 | 0.020 | 8.04E-06 | -0.081 | 0.026 | 0.002 | 0.003 | | 51.456 |  |
|  |  | rs9403580 | C | T | 0.129 | 0.108 | 0.023 | 3.47E-06 | 0.042 | 0.032 | 0.188 | 0.003 | | 47.951 |  |
|  |  | rs985416 | T | C | 0.820 | -0.097 | 0.018 | 1.46E-07 | -0.030 | 0.027 | 0.273 | 0.003 | | 51.126 |  |
| ebi-a-GCST90017026 | Gut microbiota abundance (genus Lachnospiraceae UCG004 id.11324) | rs11128180 | A | G | 0.233 | 0.065 | 0.014 | 4.52E-06 | -0.001 | 0.025 | 0.972 | 0.002 | | 27.563 |  |
|  |  | rs12072562 | T | C | 0.039 | 0.133 | 0.030 | 7.07E-06 | 0.013 | 0.054 | 0.815 | 0.001 | | 24.241 |  |
|  |  | rs12673420 | G | A | 0.499 | 0.055 | 0.012 | 2.98E-06 | -0.039 | 0.021 | 0.068 | 0.002 | | 28.220 |  |
|  |  | rs12747809 | A | G | 0.718 | 0.062 | 0.013 | 8.65E-07 | 0.003 | 0.023 | 0.914 | 0.002 | | 28.760 |  |
|  |  | rs12894272 | G | A | 0.663 | -0.058 | 0.013 | 4.34E-06 | -0.019 | 0.022 | 0.405 | 0.002 | | 27.592 |  |
|  |  | rs233486 | G | A | 0.860 | 0.080 | 0.018 | 6.28E-06 | -0.039 | 0.030 | 0.197 | 0.002 | | 28.206 |  |
|  |  | rs2444793 | T | C | 0.613 | 0.054 | 0.012 | 4.77E-06 | -0.021 | 0.022 | 0.322 | 0.001 | | 25.636 |  |
|  |  | rs2726805 | A | G | 0.471 | 0.055 | 0.012 | 6.30E-06 | -0.021 | 0.021 | 0.339 | 0.001 | | 27.521 |  |
|  |  | rs2882478 | G | A | 0.538 | -0.058 | 0.012 | 1.21E-06 | -0.012 | 0.021 | 0.568 | 0.002 | | 30.411 |  |
|  |  | rs35182105 | A | G | 0.054 | -0.110 | 0.024 | 4.87E-06 | -0.053 | 0.047 | 0.260 | 0.001 | | 22.549 |  |
|  |  | rs6656451 | T | C | 0.530 | 0.054 | 0.012 | 5.57E-06 | 0.001 | 0.021 | 0.974 | 0.001 | | 27.034 |  |
|  |  | rs7629954 | A | G | 0.045 | 0.108 | 0.024 | 5.77E-06 | -0.010 | 0.051 | 0.850 | 0.001 | | 18.687 |  |
| ebi-a-GCST90017027 | Gut microbiota abundance (genus Lachnospiraceae UCG008 id.11328) | rs10741777 | T | C | 0.308 | -0.097 | 0.019 | 7.69E-07 | 0.037 | 0.023 | 0.102 | 0.004 | | 74.448 |  |
|  |  | rs10793103 | T | C | 0.550 | -0.097 | 0.018 | 9.35E-08 | -0.017 | 0.021 | 0.434 | 0.005 | | 86.575 |  |
|  |  | rs10801803 | G | A | 0.144 | -0.117 | 0.024 | 1.40E-06 | -0.026 | 0.030 | 0.399 | 0.003 | | 61.946 |  |
|  |  | rs13024781 | T | C | 0.521 | -0.080 | 0.017 | 2.29E-06 | 0.028 | 0.021 | 0.184 | 0.003 | | 58.588 |  |
|  |  | rs57091572 | A | G | 0.132 | -0.110 | 0.024 | 2.86E-06 | 0.032 | 0.031 | 0.300 | 0.003 | | 51.334 |  |
|  |  | rs57254474 | G | A | 0.212 | 0.089 | 0.020 | 6.92E-06 | 0.010 | 0.026 | 0.697 | 0.003 | | 48.279 |  |
|  |  | rs61944774 | A | G | 0.053 | 0.180 | 0.039 | 6.34E-06 | 0.019 | 0.048 | 0.698 | 0.003 | | 59.782 |  |
|  |  | rs62277846 | C | T | 0.192 | 0.102 | 0.021 | 1.59E-06 | -0.020 | 0.027 | 0.456 | 0.003 | | 59.801 |  |
|  |  | rs67078837 | T | C | 0.418 | -0.085 | 0.017 | 7.68E-07 | 0.028 | 0.021 | 0.191 | 0.003 | | 64.037 |  |
|  |  | rs75356640 | G | A | 0.124 | 0.137 | 0.030 | 9.83E-06 | -0.054 | 0.033 | 0.100 | 0.004 | | 74.763 |  |
|  |  | rs955844 | A | C | 0.140 | 0.112 | 0.023 | 1.81E-06 | 0.000 | 0.031 | 0.993 | 0.003 | | 55.723 |  |
| ebi-a-GCST90017028 | Gut microbiota abundance (genus Lachnospiraceae UCG010 id.11330) | rs10414815 | C | T | 0.953 | -0.105 | 0.023 | 4.24E-06 | 0.016 | 0.050 | 0.744 | 0.001 | | 17.858 |  |
|  |  | rs11192447 | A | G | 0.050 | 0.127 | 0.024 | 4.69E-07 | -0.047 | 0.049 | 0.341 | 0.002 | | 28.199 |  |
|  |  | rs12346653 | C | T | 0.211 | 0.066 | 0.014 | 2.70E-06 | 0.011 | 0.026 | 0.671 | 0.001 | | 26.467 |  |
|  |  | rs17730011 | G | A | 0.215 | -0.070 | 0.016 | 7.85E-06 | -0.003 | 0.026 | 0.924 | 0.002 | | 30.618 |  |
|  |  | rs2833528 | T | C | 0.626 | 0.056 | 0.013 | 9.92E-06 | -0.025 | 0.022 | 0.245 | 0.001 | | 27.178 |  |
|  |  | rs336138 | G | T | 0.115 | 0.078 | 0.017 | 7.48E-06 | -0.070 | 0.033 | 0.034 | 0.001 | | 22.780 |  |
|  |  | rs4576377 | C | A | 0.633 | 0.057 | 0.013 | 7.63E-06 | 0.013 | 0.022 | 0.546 | 0.002 | | 27.919 |  |
|  |  | rs72894957 | G | A | 0.023 | 0.222 | 0.049 | 5.68E-06 | -0.050 | 0.071 | 0.481 | 0.002 | | 40.248 |  |
|  |  | rs74315802 | G | T | 0.184 | 0.087 | 0.018 | 3.19E-06 | 0.006 | 0.028 | 0.825 | 0.002 | | 41.442 |  |
|  |  | rs9981767 | A | C | 0.258 | 0.066 | 0.013 | 9.96E-07 | 0.021 | 0.024 | 0.394 | 0.002 | | 30.200 |  |
| ebi-a-GCST90017029 | Gut microbiota abundance (genus Lachnospira id.2004) | rs56791201 | C | TRUE | 0.639 | -0.052 | 0.011 | 2.93E-06 | -0.028 | 0.022 | 0.201 | 0.001 | | 22.758 |  |
| ebi-a-GCST90017030 | Gut microbiota abundance (genus Lactobacillus id.1837) | rs12693845 | C | T | 0.411 | -0.081 | 0.018 | 8.96E-06 | -0.005 | 0.022 | 0.837 | 0.003 | | 57.763 |  |
|  |  | rs1530559 | G | A | 0.463 | 0.080 | 0.018 | 4.93E-06 | 0.040 | 0.021 | 0.065 | 0.003 | | 59.136 |  |
|  |  | rs16861661 | G | A | 0.067 | -0.183 | 0.038 | 1.28E-06 | 0.030 | 0.043 | 0.483 | 0.004 | | 76.807 |  |
|  |  | rs62314653 | C | A | 0.059 | 0.188 | 0.039 | 2.24E-06 | 0.010 | 0.045 | 0.830 | 0.004 | | 72.508 |  |
|  |  | rs7399658 | G | A | 0.184 | -0.107 | 0.022 | 3.12E-06 | 0.012 | 0.028 | 0.679 | 0.003 | | 63.289 |  |
|  |  | rs75127669 | C | A | 0.073 | 0.140 | 0.031 | 6.83E-06 | 0.001 | 0.041 | 0.986 | 0.003 | | 48.599 |  |
|  |  | rs768253 | T | G | 0.440 | -0.079 | 0.017 | 4.25E-06 | 0.019 | 0.021 | 0.373 | 0.003 | | 56.849 |  |
|  |  | rs77478751 | A | G | 0.119 | -0.220 | 0.048 | 7.33E-06 | -0.021 | 0.033 | 0.532 | 0.010 | | 188.226 |  |
|  |  | rs921925 | A | C | 0.213 | 0.099 | 0.020 | 9.72E-07 | 0.001 | 0.026 | 0.964 | 0.003 | | 59.792 |  |
| ebi-a-GCST90017031 | Gut microbiota abundance (genus Lactococcus id.1851) | rs10417872 | G | T | 0.715 | -0.118 | 0.025 | 1.29E-06 | 0.020 | 0.023 | 0.395 | 0.006 | | 105.137 |  |
|  |  | rs123059 | C | T | 0.781 | 0.137 | 0.027 | 1.27E-06 | 0.029 | 0.026 | 0.259 | 0.006 | | 118.112 |  |
|  |  | rs12621813 | G | A | 0.267 | 0.108 | 0.024 | 6.61E-06 | -0.034 | 0.024 | 0.160 | 0.005 | | 84.689 |  |
|  |  | rs17168302 | G | A | 0.107 | 0.192 | 0.042 | 6.29E-06 | -0.051 | 0.035 | 0.145 | 0.007 | | 129.370 |  |
|  |  | rs2293361 | C | T | 0.054 | -0.199 | 0.043 | 1.40E-06 | 0.038 | 0.047 | 0.419 | 0.004 | | 74.374 |  |
|  |  | rs4766997 | C | T | 0.425 | 0.115 | 0.024 | 2.06E-06 | 0.008 | 0.021 | 0.715 | 0.006 | | 118.452 |  |
|  |  | rs55910161 | C | T | 0.112 | 0.146 | 0.031 | 2.36E-06 | 0.047 | 0.034 | 0.167 | 0.004 | | 78.727 |  |
|  |  | rs6674304 | C | T | 0.038 | 0.201 | 0.044 | 6.18E-06 | -0.110 | 0.056 | 0.049 | 0.003 | | 54.773 |  |
|  |  | rs7992246 | C | T | 0.609 | -0.104 | 0.023 | 4.45E-06 | 0.020 | 0.022 | 0.357 | 0.005 | | 95.339 |  |
| ebi-a-GCST90017032 | Gut microbiota abundance (genus Marvinbryantia id.2005) | rs11620597 | T | C | 0.024 | 0.119 | 0.027 | 7.80E-06 | 0.066 | 0.067 | 0.327 | 0.001 | | 12.507 |  |
|  |  | rs1187983 | C | T | 0.103 | -0.094 | 0.019 | 2.02E-06 | -0.049 | 0.035 | 0.161 | 0.002 | | 29.803 |  |
|  |  | rs146541147 | G | A | 0.034 | 0.119 | 0.027 | 6.86E-06 | 0.012 | 0.059 | 0.832 | 0.001 | | 16.821 |  |
|  |  | rs2724813 | G | A | 0.764 | 0.084 | 0.017 | 6.28E-07 | -0.016 | 0.025 | 0.528 | 0.003 | | 46.906 |  |
|  |  | rs2842896 | C | T | 0.587 | -0.065 | 0.013 | 7.25E-07 | -0.002 | 0.022 | 0.938 | 0.002 | | 37.581 |  |
|  |  | rs2863363 | G | A | 0.755 | -0.063 | 0.014 | 3.11E-06 | -0.005 | 0.025 | 0.848 | 0.001 | | 27.354 |  |
|  |  | rs3125832 | A | C | 0.226 | 0.068 | 0.015 | 5.03E-06 | 0.000 | 0.025 | 0.993 | 0.002 | | 29.673 |  |
|  |  | rs61884471 | G | A | 0.109 | 0.124 | 0.025 | 1.01E-06 | 0.033 | 0.034 | 0.339 | 0.003 | | 55.312 |  |
|  |  | rs72948274 | A | C | 0.064 | -0.126 | 0.027 | 3.26E-06 | 0.013 | 0.043 | 0.760 | 0.002 | | 34.918 |  |
|  |  | rs8006832 | G | T | 0.092 | -0.095 | 0.022 | 6.58E-06 | 0.037 | 0.037 | 0.317 | 0.002 | | 27.820 |  |
| ebi-a-GCST90017033 | Gut microbiota abundance (genus Methanobrevibacter id.123) | rs10202904 | G | T | 0.592 | 0.113 | 0.024 | 3.09E-06 | -0.024 | 0.022 | 0.263 | 0.006 | | 113.460 |  |
|  |  | rs1334944 | T | C | 0.278 | 0.115 | 0.026 | 7.61E-06 | -0.035 | 0.024 | 0.136 | 0.005 | | 98.256 |  |
|  |  | rs4802933 | G | A | 0.768 | 0.136 | 0.031 | 9.74E-06 | 0.003 | 0.025 | 0.895 | 0.007 | | 121.110 |  |
|  |  | rs6776814 | T | C | 0.020 | -0.189 | 0.042 | 8.05E-06 | 0.017 | 0.074 | 0.819 | 0.001 | | 26.294 |  |
|  |  | rs76029318 | T | C | 0.063 | 0.223 | 0.045 | 1.08E-06 | 0.008 | 0.043 | 0.846 | 0.006 | | 107.927 |  |
|  |  | rs894996 | C | A | 0.070 | 0.214 | 0.046 | 3.82E-06 | 0.028 | 0.041 | 0.492 | 0.006 | | 110.161 |  |
| ebi-a-GCST90017034 | Gut microbiota abundance (genus Odoribacter id.952) | rs10093869 | A | G | 0.423 | -0.058 | 0.013 | 3.67E-06 | 0.005 | 0.022 | 0.802 | 0.002 | | 29.928 |  |
|  |  | rs10423795 | T | C | 0.616 | -0.055 | 0.012 | 6.58E-06 | 0.022 | 0.022 | 0.315 | 0.001 | | 26.340 |  |
|  |  | rs28417404 | A | G | 0.096 | -0.073 | 0.016 | 3.68E-06 | 0.025 | 0.036 | 0.479 | 0.001 | | 16.893 |  |
|  |  | rs4793970 | A | G | 0.363 | -0.058 | 0.013 | 6.03E-06 | -0.040 | 0.022 | 0.070 | 0.002 | | 28.216 |  |
|  |  | rs6856150 | A | G | 0.877 | -0.088 | 0.019 | 6.06E-06 | -0.054 | 0.032 | 0.092 | 0.002 | | 30.758 |  |
|  |  | rs74553962 | T | G | 0.076 | 0.121 | 0.026 | 9.49E-06 | -0.049 | 0.040 | 0.229 | 0.002 | | 38.137 |  |
|  |  | rs77779484 | G | A | 0.061 | -0.133 | 0.027 | 6.56E-07 | -0.001 | 0.045 | 0.978 | 0.002 | | 37.442 |  |
| ebi-a-GCST90017035 | Gut microbiota abundance (genus Olsenella id.822) | rs1035588 | A | G | 0.374 | -0.108 | 0.024 | 4.86E-06 | 0.012 | 0.022 | 0.572 | 0.005 | | 100.940 |  |
|  |  | rs17148768 | G | A | 0.168 | 0.140 | 0.030 | 2.20E-06 | 0.006 | 0.028 | 0.825 | 0.006 | | 101.517 |  |
|  |  | rs2759329 | A | G | 0.631 | 0.111 | 0.024 | 3.43E-06 | -0.011 | 0.022 | 0.604 | 0.006 | | 106.063 |  |
|  |  | rs35225860 | A | G | 0.039 | -0.224 | 0.048 | 3.87E-06 | 0.001 | 0.054 | 0.991 | 0.004 | | 69.309 |  |
|  |  | rs61090148 | A | G | 0.432 | -0.105 | 0.023 | 6.44E-06 | 0.006 | 0.021 | 0.781 | 0.005 | | 99.322 |  |
|  |  | rs62112538 | C | T | 0.111 | -0.199 | 0.041 | 1.19E-06 | 0.012 | 0.034 | 0.715 | 0.008 | | 145.427 |  |
|  |  | rs72691585 | C | A | 0.134 | -0.249 | 0.052 | 2.95E-06 | -0.063 | 0.031 | 0.045 | 0.014 | | 267.560 |  |
|  |  | rs7540303 | C | T | 0.366 | 0.108 | 0.024 | 5.32E-06 | -0.028 | 0.022 | 0.200 | 0.005 | | 99.853 |  |
|  |  | rs8066522 | A | G | 0.668 | 0.107 | 0.024 | 9.70E-06 | -0.078 | 0.023 | 0.001 | 0.005 | | 92.759 |  |
|  |  | rs9460691 | C | A | 0.195 | 0.120 | 0.027 | 7.28E-06 | -0.007 | 0.027 | 0.794 | 0.005 | | 83.233 |  |
| ebi-a-GCST90017036 | Gut microbiota abundance (genus Oscillibacter id.2063) | rs11627628 | T | C | 0.076 | 0.144 | 0.029 | 1.01E-06 | 0.022 | 0.040 | 0.588 | 0.003 | | 53.838 |  |
|  |  | rs11990279 | T | C | 0.200 | -0.082 | 0.018 | 4.94E-06 | 0.017 | 0.030 | 0.564 | 0.002 | | 39.991 |  |
|  |  | rs12649930 | T | G | 0.100 | 0.122 | 0.026 | 4.09E-06 | 0.005 | 0.036 | 0.900 | 0.003 | | 48.929 |  |
|  |  | rs133832 | A | C | 0.283 | -0.080 | 0.016 | 1.15E-06 | -0.015 | 0.024 | 0.538 | 0.003 | | 47.178 |  |
|  |  | rs16866406 | A | G | 0.153 | 0.099 | 0.021 | 3.08E-06 | 0.000 | 0.029 | 0.991 | 0.003 | | 46.485 |  |
|  |  | rs16934185 | A | G | 0.103 | -0.130 | 0.028 | 4.38E-06 | 0.016 | 0.035 | 0.650 | 0.003 | | 57.211 |  |
|  |  | rs234108 | A | G | 0.396 | 0.075 | 0.015 | 9.16E-07 | 0.011 | 0.022 | 0.599 | 0.003 | | 49.401 |  |
|  |  | rs36095275 | C | T | 0.418 | -0.075 | 0.016 | 1.40E-06 | 0.035 | 0.022 | 0.105 | 0.003 | | 50.628 |  |
|  |  | rs4506202 | G | A | 0.469 | 0.071 | 0.015 | 3.21E-06 | 0.017 | 0.021 | 0.433 | 0.003 | | 46.326 |  |
|  |  | rs61883564 | A | G | 0.136 | -0.101 | 0.022 | 3.39E-06 | -0.012 | 0.031 | 0.709 | 0.002 | | 44.430 |  |
|  |  | rs75453768 | G | T | 0.098 | 0.122 | 0.027 | 5.35E-06 | 0.012 | 0.036 | 0.732 | 0.003 | | 48.499 |  |
|  |  | rs761240 | G | T | 0.949 | 0.177 | 0.039 | 2.04E-06 | 0.027 | 0.049 | 0.583 | 0.003 | | 55.347 |  |
|  |  | rs9393920 | G | A | 0.621 | 0.074 | 0.015 | 9.92E-07 | -0.044 | 0.022 | 0.049 | 0.003 | | 47.972 |  |
| ebi-a-GCST90017037 | Gut microbiota abundance (genus Oscillospira id.2064) | rs12206468 | G | A | 0.078 | -0.133 | 0.027 | 1.04E-06 | 0.041 | 0.039 | 0.293 | 0.003 | | 46.781 |  |
|  |  | rs12925026 | T | C | 0.057 | 0.136 | 0.031 | 9.31E-06 | -0.037 | 0.046 | 0.417 | 0.002 | | 36.177 |  |
|  |  | rs1954532 | C | T | 0.800 | 0.083 | 0.018 | 2.27E-06 | 0.053 | 0.027 | 0.048 | 0.002 | | 40.134 |  |
|  |  | rs28889936 | A | C | 0.096 | 0.114 | 0.025 | 3.37E-06 | -0.056 | 0.036 | 0.122 | 0.002 | | 41.387 |  |
|  |  | rs62422654 | C | T | 0.208 | 0.090 | 0.020 | 6.47E-06 | -0.064 | 0.026 | 0.014 | 0.003 | | 48.919 |  |
|  |  | rs72866977 | A | C | 0.079 | -0.131 | 0.028 | 5.63E-06 | 0.034 | 0.040 | 0.395 | 0.002 | | 45.702 |  |
|  |  | rs751183 | C | T | 0.819 | 0.077 | 0.017 | 6.85E-06 | -0.022 | 0.028 | 0.430 | 0.002 | | 32.614 |  |
|  |  | rs8076323 | A | G | 0.325 | 0.072 | 0.016 | 5.61E-06 | -0.029 | 0.023 | 0.192 | 0.002 | | 41.277 |  |
| ebi-a-GCST90017038 | Gut microbiota abundance (genus Oxalobacter id.2978) | rs10464997 | G | A | 0.185 | 0.138 | 0.029 | 3.30E-06 | -0.010 | 0.027 | 0.705 | 0.006 | | 105.309 |  |
|  |  | rs11108500 | A | G | 0.088 | -0.199 | 0.043 | 3.74E-06 | 0.058 | 0.038 | 0.126 | 0.006 | | 117.597 |  |
|  |  | rs111966731 | T | C | 0.086 | 0.213 | 0.047 | 7.30E-06 | -0.020 | 0.038 | 0.604 | 0.007 | | 132.309 |  |
|  |  | rs12002250 | A | C | 0.043 | 0.217 | 0.047 | 1.42E-06 | -0.004 | 0.052 | 0.947 | 0.004 | | 71.267 |  |
|  |  | rs1569853 | T | C | 0.121 | -0.138 | 0.030 | 3.65E-06 | -0.027 | 0.032 | 0.406 | 0.004 | | 74.674 |  |
|  |  | rs36057338 | G | T | 0.033 | 0.208 | 0.042 | 8.80E-07 | -0.042 | 0.059 | 0.480 | 0.003 | | 50.834 |  |
|  |  | rs3862635 | C | T | 0.091 | -0.172 | 0.039 | 9.19E-06 | -0.007 | 0.037 | 0.849 | 0.005 | | 90.208 |  |
|  |  | rs4428215 | G | A | 0.253 | 0.130 | 0.024 | 7.51E-08 | -0.015 | 0.024 | 0.535 | 0.006 | | 118.554 |  |
|  |  | rs6000536 | C | T | 0.169 | -0.131 | 0.025 | 2.06E-07 | 0.022 | 0.028 | 0.445 | 0.005 | | 88.683 |  |
|  |  | rs6993398 | G | A | 0.183 | 0.127 | 0.028 | 7.13E-06 | -0.002 | 0.027 | 0.936 | 0.005 | | 89.139 |  |
|  |  | rs736744 | T | C | 0.568 | -0.118 | 0.021 | 2.57E-08 | -0.018 | 0.021 | 0.401 | 0.007 | | 125.923 |  |
| ebi-a-GCST90017039 | Gut microbiota abundance (genus Parabacteroides id.954) | rs115602804 | G | A | 0.107 | 0.103 | 0.022 | 1.93E-06 | -0.018 | 0.034 | 0.603 | 0.002 | | 37.404 |  |
|  |  | rs4236095 | A | G | 0.897 | -0.076 | 0.016 | 1.93E-06 | -0.038 | 0.035 | 0.272 | 0.001 | | 19.728 |  |
|  |  | rs60884758 | C | T | 0.178 | -0.070 | 0.014 | 5.71E-07 | -0.029 | 0.028 | 0.293 | 0.001 | | 26.569 |  |
|  |  | rs6657302 | T | C | 0.063 | -0.105 | 0.023 | 9.76E-06 | 0.062 | 0.044 | 0.155 | 0.001 | | 23.756 |  |
|  |  | rs7298818 | C | T | 0.101 | 0.089 | 0.020 | 8.54E-06 | -0.004 | 0.035 | 0.917 | 0.001 | | 26.416 |  |
| ebi-a-GCST90017040 | Gut microbiota abundance (genus Paraprevotella id.962) | rs10842464 | C | T | 0.699 | 0.076 | 0.017 | 6.60E-06 | 0.010 | 0.023 | 0.661 | 0.002 | | 44.495 |  |
|  |  | rs140997932 | T | C | 0.055 | -0.162 | 0.035 | 2.11E-06 | 0.013 | 0.046 | 0.787 | 0.003 | | 50.536 |  |
|  |  | rs145020347 | A | G | 0.148 | -0.125 | 0.026 | 4.03E-06 | -0.015 | 0.030 | 0.625 | 0.004 | | 72.180 |  |
|  |  | rs17109926 | A | G | 0.272 | -0.099 | 0.022 | 6.75E-06 | -0.017 | 0.024 | 0.488 | 0.004 | | 71.133 |  |
|  |  | rs17785622 | A | G | 0.038 | 0.248 | 0.052 | 1.93E-06 | -0.036 | 0.056 | 0.523 | 0.004 | | 82.855 |  |
|  |  | rs2081023 | A | G | 0.143 | -0.123 | 0.024 | 2.64E-07 | 0.013 | 0.030 | 0.675 | 0.004 | | 67.646 |  |
|  |  | rs3008582 | T | C | 0.195 | 0.106 | 0.023 | 4.36E-06 | -0.034 | 0.027 | 0.213 | 0.004 | | 64.573 |  |
|  |  | rs3801748 | G | A | 0.363 | 0.078 | 0.017 | 5.20E-06 | -0.018 | 0.022 | 0.405 | 0.003 | | 51.674 |  |
|  |  | rs4756632 | G | T | 0.129 | -0.139 | 0.029 | 3.82E-06 | 0.018 | 0.032 | 0.578 | 0.004 | | 80.069 |  |
|  |  | rs4767113 | C | T | 0.328 | 0.088 | 0.018 | 2.14E-06 | -0.012 | 0.023 | 0.590 | 0.003 | | 63.141 |  |
|  |  | rs7240324 | T | G | 0.246 | -0.102 | 0.023 | 5.96E-06 | -0.020 | 0.025 | 0.416 | 0.004 | | 71.398 |  |
|  |  | rs9602779 | A | C | 0.242 | -0.107 | 0.022 | 6.93E-07 | 0.020 | 0.025 | 0.421 | 0.004 | | 76.839 |  |
|  |  | rs9900242 | A | G | 0.343 | -0.085 | 0.018 | 1.14E-06 | -0.008 | 0.022 | 0.709 | 0.003 | | 60.296 |  |
| ebi-a-GCST90017041 | Gut microbiota abundance (genus Parasutterella id.2892) | rs10899911 | A | G | 0.235 | -0.072 | 0.015 | 1.15E-06 | 0.003 | 0.025 | 0.890 | 0.002 | | 33.928 |  |
|  |  | rs11715853 | G | A | 0.295 | -0.066 | 0.015 | 6.23E-06 | 0.006 | 0.023 | 0.811 | 0.002 | | 33.605 |  |
|  |  | rs2090816 | C | A | 0.816 | -0.084 | 0.018 | 2.90E-06 | -0.002 | 0.028 | 0.932 | 0.002 | | 38.978 |  |
|  |  | rs35055552 | T | C | 0.141 | 0.110 | 0.024 | 3.35E-06 | 0.031 | 0.031 | 0.317 | 0.003 | | 53.407 |  |
|  |  | rs55877868 | A | C | 0.101 | -0.104 | 0.023 | 2.87E-06 | 0.015 | 0.035 | 0.668 | 0.002 | | 36.377 |  |
|  |  | rs62273907 | A | G | 0.067 | 0.229 | 0.050 | 5.88E-06 | -0.018 | 0.042 | 0.667 | 0.007 | | 122.080 |  |
|  |  | rs6809952 | G | A | 0.278 | -0.068 | 0.015 | 8.13E-06 | -0.043 | 0.024 | 0.077 | 0.002 | | 34.618 |  |
|  |  | rs6828768 | C | T | 0.468 | 0.064 | 0.013 | 1.78E-06 | -0.006 | 0.021 | 0.792 | 0.002 | | 37.113 |  |
|  |  | rs7303158 | C | T | 0.449 | 0.065 | 0.013 | 1.33E-06 | 0.028 | 0.021 | 0.194 | 0.002 | | 38.051 |  |
|  |  | rs7311004 | C | T | 0.556 | 0.062 | 0.014 | 5.92E-06 | 0.011 | 0.021 | 0.602 | 0.002 | | 34.588 |  |
|  |  | rs7572229 | A | G | 0.478 | -0.066 | 0.013 | 6.32E-07 | -0.007 | 0.021 | 0.735 | 0.002 | | 40.278 |  |
|  |  | rs78383039 | T | C | 0.041 | -0.146 | 0.030 | 1.57E-06 | 0.005 | 0.054 | 0.933 | 0.002 | | 30.808 |  |
|  |  | rs8039785 | G | T | 0.490 | -0.062 | 0.013 | 3.62E-06 | 0.028 | 0.021 | 0.187 | 0.002 | | 35.111 |  |
|  |  | rs823424 | G | A | 0.254 | -0.071 | 0.016 | 4.95E-06 | -0.029 | 0.024 | 0.235 | 0.002 | | 35.460 |  |
| ebi-a-GCST90017042 | Gut microbiota abundance (genus Peptococcus id.2037) | rs10031059 | C | T | 0.765 | 0.121 | 0.023 | 1.24E-07 | 0.006 | 0.025 | 0.820 | 0.005 | | 97.372 |  |
|  |  | rs11001941 | G | A | 0.087 | -0.196 | 0.039 | 1.33E-06 | -0.036 | 0.038 | 0.342 | 0.006 | | 112.340 |  |
|  |  | rs12069354 | C | T | 0.063 | 0.168 | 0.038 | 9.28E-06 | 0.008 | 0.043 | 0.851 | 0.003 | | 60.622 |  |
|  |  | rs2054133 | A | G | 0.634 | -0.090 | 0.019 | 2.14E-06 | 0.007 | 0.022 | 0.745 | 0.004 | | 68.460 |  |
|  |  | rs36121075 | A | G | 0.169 | -0.141 | 0.031 | 6.99E-06 | -0.033 | 0.029 | 0.245 | 0.006 | | 102.638 |  |
|  |  | rs413827 | G | A | 0.238 | 0.110 | 0.024 | 3.30E-06 | -0.023 | 0.025 | 0.361 | 0.004 | | 81.246 |  |
|  |  | rs5770862 | T | C | 0.093 | 0.162 | 0.036 | 3.22E-06 | -0.047 | 0.036 | 0.195 | 0.004 | | 81.466 |  |
|  |  | rs6918730 | A | G | 0.940 | -0.135 | 0.029 | 1.15E-06 | 0.029 | 0.044 | 0.507 | 0.002 | | 38.188 |  |
|  |  | rs7033353 | G | T | 0.477 | -0.090 | 0.019 | 2.22E-06 | 0.000 | 0.021 | 0.989 | 0.004 | | 74.669 |  |
|  |  | rs72850165 | T | C | 0.077 | -0.134 | 0.030 | 5.74E-06 | -0.008 | 0.039 | 0.835 | 0.003 | | 47.379 |  |
|  |  | rs74592222 | G | A | 0.116 | 0.138 | 0.030 | 8.55E-06 | -0.017 | 0.033 | 0.616 | 0.004 | | 71.696 |  |
|  |  | rs77681628 | C | T | 0.082 | 0.200 | 0.039 | 2.69E-07 | 0.028 | 0.039 | 0.465 | 0.006 | | 111.009 |  |
| ebi-a-GCST90017043 | Gut microbiota abundance (genus Phascolarctobacterium id.2168) | rs11929846 | T | C | 0.220 | -0.070 | 0.016 | 8.88E-06 | -0.003 | 0.026 | 0.918 | 0.002 | | 30.623 |  |
|  |  | rs12618201 | A | G | 0.442 | 0.064 | 0.014 | 3.38E-06 | -0.013 | 0.021 | 0.532 | 0.002 | | 37.310 |  |
|  |  | rs1264476 | G | T | 0.814 | -0.077 | 0.017 | 4.30E-06 | -0.022 | 0.027 | 0.423 | 0.002 | | 32.822 |  |
|  |  | rs56069061 | G | A | 0.068 | -0.111 | 0.023 | 1.87E-06 | 0.077 | 0.042 | 0.070 | 0.002 | | 28.834 |  |
|  |  | rs56157888 | A | C | 0.222 | 0.095 | 0.019 | 1.09E-06 | -0.018 | 0.026 | 0.483 | 0.003 | | 57.939 |  |
|  |  | rs74540770 | G | A | 0.081 | -0.121 | 0.026 | 3.60E-06 | 0.005 | 0.039 | 0.897 | 0.002 | | 39.851 |  |
|  |  | rs75882962 | T | C | 0.127 | 0.097 | 0.019 | 3.19E-07 | -0.079 | 0.032 | 0.013 | 0.002 | | 38.234 |  |
|  |  | rs7982713 | G | A | 0.283 | 0.073 | 0.016 | 9.72E-06 | 0.004 | 0.024 | 0.854 | 0.002 | | 39.434 |  |
| ebi-a-GCST90017044 | Gut microbiota abundance (genus Prevotella7 id.11182) | rs118038478 | A | G | 0.070 | 0.206 | 0.047 | 7.85E-06 | 0.027 | 0.041 | 0.507 | 0.006 | | 102.033 |  |
|  |  | rs12124567 | A | G | 0.203 | -0.121 | 0.028 | 9.49E-06 | 0.052 | 0.027 | 0.052 | 0.005 | | 87.828 |  |
|  |  | rs12195431 | T | C | 0.097 | 0.197 | 0.044 | 8.73E-06 | -0.012 | 0.036 | 0.745 | 0.007 | | 125.385 |  |
|  |  | rs2240542 | C | T | 0.265 | 0.121 | 0.026 | 4.84E-06 | 0.007 | 0.024 | 0.774 | 0.006 | | 104.927 |  |
|  |  | rs2918132 | T | C | 0.628 | 0.115 | 0.025 | 6.42E-06 | 0.016 | 0.022 | 0.455 | 0.006 | | 113.329 |  |
|  |  | rs430270 | A | C | 0.191 | 0.139 | 0.030 | 2.87E-06 | 0.013 | 0.027 | 0.626 | 0.006 | | 110.222 |  |
|  |  | rs57404562 | C | A | 0.129 | 0.155 | 0.032 | 6.22E-07 | -0.054 | 0.032 | 0.090 | 0.005 | | 99.971 |  |
|  |  | rs79263163 | A | C | 0.200 | -0.144 | 0.032 | 7.51E-06 | 0.009 | 0.026 | 0.729 | 0.007 | | 122.557 |  |
|  |  | rs9426434 | T | C | 0.351 | -0.124 | 0.028 | 9.72E-06 | -0.006 | 0.022 | 0.807 | 0.007 | | 128.598 |  |
|  |  | rs9608249 | A | G | 0.113 | -0.158 | 0.034 | 2.07E-06 | -0.050 | 0.033 | 0.135 | 0.005 | | 92.194 |  |
|  |  | rs9959718 | G | A | 0.203 | 0.133 | 0.028 | 1.90E-06 | 0.024 | 0.026 | 0.371 | 0.006 | | 105.743 |  |
| ebi-a-GCST90017045 | Gut microbiota abundance (genus Prevotella9 id.11183) | rs111509883 | T | C | 0.111 | 0.171 | 0.035 | 1.24E-06 | -0.026 | 0.034 | 0.443 | 0.006 | | 106.859 |  |
|  |  | rs11685699 | C | T | 0.078 | -0.141 | 0.030 | 2.03E-06 | -0.069 | 0.040 | 0.082 | 0.003 | | 52.769 |  |
|  |  | rs117271932 | A | G | 0.056 | 0.208 | 0.044 | 2.82E-06 | -0.014 | 0.046 | 0.752 | 0.005 | | 85.037 |  |
|  |  | rs12648235 | T | C | 0.237 | 0.079 | 0.018 | 7.39E-06 | 0.008 | 0.025 | 0.749 | 0.002 | | 40.995 |  |
|  |  | rs1304512 | G | A | 0.272 | 0.076 | 0.017 | 5.29E-06 | -0.030 | 0.024 | 0.202 | 0.002 | | 42.182 |  |
|  |  | rs2104588 | C | T | 0.944 | -0.106 | 0.024 | 8.13E-06 | 0.081 | 0.046 | 0.076 | 0.001 | | 21.774 |  |
|  |  | rs2495052 | A | G | 0.148 | 0.084 | 0.019 | 8.97E-06 | -0.019 | 0.030 | 0.522 | 0.002 | | 32.610 |  |
|  |  | rs2683313 | G | A | 0.686 | 0.072 | 0.015 | 1.69E-06 | -0.026 | 0.023 | 0.254 | 0.002 | | 41.578 |  |
|  |  | rs4968431 | G | T | 0.367 | 0.064 | 0.014 | 8.58E-06 | 0.039 | 0.022 | 0.075 | 0.002 | | 34.958 |  |
|  |  | rs7237249 | C | T | 0.188 | -0.082 | 0.018 | 8.93E-06 | 0.028 | 0.027 | 0.299 | 0.002 | | 38.103 |  |
|  |  | rs72815774 | T | C | 0.060 | -0.176 | 0.039 | 8.78E-06 | -0.012 | 0.047 | 0.794 | 0.003 | | 64.365 |  |
|  |  | rs746764 | T | C | 0.214 | -0.092 | 0.019 | 2.04E-06 | -0.022 | 0.026 | 0.405 | 0.003 | | 51.935 |  |
|  |  | rs7976209 | T | C | 0.155 | -0.087 | 0.020 | 7.28E-06 | 0.004 | 0.029 | 0.882 | 0.002 | | 36.516 |  |
|  |  | rs9428102 | A | G | 0.218 | -0.078 | 0.018 | 4.62E-06 | -0.009 | 0.025 | 0.719 | 0.002 | | 37.942 |  |
|  |  | rs9613013 | G | A | 0.123 | 0.092 | 0.020 | 6.10E-06 | -0.006 | 0.032 | 0.852 | 0.002 | | 33.301 |  |
| ebi-a-GCST90017046 | Gut microbiota abundance (genus Rikenellaceae RC9 gut group id.11191) | rs12501673 | A | G | 0.275 | 0.116 | 0.026 | 6.29E-06 | -0.005 | 0.024 | 0.852 | 0.005 | | 99.535 |  |
|  |  | rs17032291 | T | C | 0.126 | -0.170 | 0.037 | 6.61E-06 | -0.060 | 0.032 | 0.060 | 0.006 | | 117.127 |  |
|  |  | rs17582787 | A | G | 0.165 | -0.158 | 0.034 | 3.55E-06 | 0.005 | 0.028 | 0.849 | 0.007 | | 126.596 |  |
|  |  | rs2074881 | T | C | 0.132 | -0.142 | 0.032 | 9.45E-06 | 0.008 | 0.031 | 0.797 | 0.005 | | 85.411 |  |
|  |  | rs2900503 | T | G | 0.837 | 0.172 | 0.033 | 1.55E-07 | -0.055 | 0.029 | 0.053 | 0.008 | | 149.513 |  |
|  |  | rs2998141 | C | T | 0.765 | 0.136 | 0.029 | 4.42E-06 | -0.014 | 0.025 | 0.588 | 0.007 | | 123.325 |  |
|  |  | rs4270579 | A | G | 0.674 | 0.118 | 0.027 | 5.46E-06 | 0.017 | 0.023 | 0.460 | 0.006 | | 112.939 |  |
|  |  | rs4717843 | G | T | 0.473 | -0.119 | 0.026 | 4.72E-06 | 0.004 | 0.021 | 0.860 | 0.007 | | 131.221 |  |
|  |  | rs7712231 | A | G | 0.133 | 0.156 | 0.035 | 7.97E-06 | -0.017 | 0.031 | 0.588 | 0.006 | | 103.814 |  |
|  |  | rs80309088 | G | A | 0.119 | 0.174 | 0.038 | 4.56E-06 | -0.053 | 0.033 | 0.107 | 0.006 | | 117.225 |  |
|  |  | rs9887954 | G | A | 0.409 | -0.115 | 0.025 | 4.81E-06 | -0.026 | 0.022 | 0.238 | 0.006 | | 117.704 |  |
| ebi-a-GCST90017047 | Gut microbiota abundance (genus Romboutsia id.11347) | rs10279978 | A | G | 0.311 | -0.062 | 0.013 | 1.17E-06 | 0.008 | 0.023 | 0.716 | 0.002 | | 30.486 |  |
|  |  | rs11221428 | T | C | 0.226 | -0.073 | 0.016 | 6.49E-06 | -0.054 | 0.025 | 0.032 | 0.002 | | 33.982 |  |
|  |  | rs16843578 | C | T | 0.053 | -0.088 | 0.020 | 5.08E-06 | -0.043 | 0.047 | 0.363 | 0.001 | | 14.121 |  |
|  |  | rs28603357 | T | C | 0.020 | -0.215 | 0.047 | 8.52E-06 | 0.204 | 0.076 | 0.007 | 0.002 | | 33.376 |  |
|  |  | rs34302036 | G | A | 0.577 | -0.055 | 0.012 | 5.88E-06 | 0.048 | 0.021 | 0.025 | 0.001 | | 27.149 |  |
|  |  | rs61841503 | G | A | 0.129 | 0.093 | 0.017 | 4.00E-08 | 0.011 | 0.031 | 0.722 | 0.002 | | 35.695 |  |
|  |  | rs62504452 | A | G | 0.139 | -0.071 | 0.016 | 4.66E-06 | -0.037 | 0.031 | 0.223 | 0.001 | | 22.156 |  |
|  |  | rs7109293 | A | G | 0.117 | 0.092 | 0.021 | 6.98E-06 | -0.027 | 0.033 | 0.419 | 0.002 | | 32.112 |  |
|  |  | rs75200530 | T | G | 0.029 | -0.191 | 0.042 | 5.07E-06 | 0.017 | 0.063 | 0.781 | 0.002 | | 38.142 |  |
|  |  | rs75987356 | G | A | 0.078 | -0.130 | 0.028 | 6.71E-06 | -0.012 | 0.040 | 0.769 | 0.002 | | 44.639 |  |
|  |  | rs77702691 | A | G | 0.089 | -0.094 | 0.021 | 7.37E-06 | -0.060 | 0.037 | 0.105 | 0.001 | | 26.536 |  |
|  |  | rs9389266 | T | G | 0.175 | 0.072 | 0.016 | 9.38E-06 | 0.031 | 0.028 | 0.258 | 0.002 | | 27.732 |  |
|  |  | rs9567264 | C | T | 0.335 | 0.058 | 0.013 | 5.76E-06 | -0.017 | 0.022 | 0.462 | 0.001 | | 27.545 |  |
| ebi-a-GCST90017048 | Gut microbiota abundance (genus Roseburia id.2012) | rs12740451 | T | C | 0.135 | 0.070 | 0.015 | 7.34E-06 | 0.004 | 0.031 | 0.897 | 0.001 | | 20.862 |  |
|  |  | rs16910295 | T | C | 0.055 | -0.098 | 0.021 | 2.91E-06 | 0.004 | 0.047 | 0.931 | 0.001 | | 18.383 |  |
|  |  | rs2160994 | C | T | 0.665 | -0.055 | 0.011 | 9.70E-07 | 0.002 | 0.022 | 0.919 | 0.001 | | 24.811 |  |
|  |  | rs2943022 | T | C | 0.584 | 0.049 | 0.011 | 4.11E-06 | -0.016 | 0.021 | 0.461 | 0.001 | | 21.759 |  |
|  |  | rs302266 | T | C | 0.128 | -0.078 | 0.017 | 8.13E-06 | -0.015 | 0.032 | 0.638 | 0.001 | | 24.702 |  |
|  |  | rs329182 | T | C | 0.161 | 0.069 | 0.015 | 5.90E-06 | 0.047 | 0.029 | 0.101 | 0.001 | | 23.592 |  |
|  |  | rs55858165 | A | C | 0.038 | 0.179 | 0.040 | 9.99E-06 | -0.013 | 0.056 | 0.820 | 0.002 | | 42.977 |  |
|  |  | rs57466170 | C | T | 0.075 | 0.074 | 0.017 | 8.30E-06 | -0.002 | 0.040 | 0.956 | 0.001 | | 14.016 |  |
|  |  | rs6445851 | A | G | 0.627 | 0.050 | 0.011 | 3.53E-06 | -0.024 | 0.022 | 0.271 | 0.001 | | 21.236 |  |
|  |  | rs6930661 | C | T | 0.061 | -0.096 | 0.020 | 2.48E-06 | 0.029 | 0.045 | 0.522 | 0.001 | | 19.439 |  |
|  |  | rs75326254 | C | T | 0.059 | -0.105 | 0.023 | 7.50E-06 | 0.000 | 0.045 | 0.999 | 0.001 | | 22.293 |  |
|  |  | rs78753150 | A | C | 0.100 | 0.097 | 0.021 | 9.98E-06 | 0.000 | 0.036 | 0.998 | 0.002 | | 30.894 |  |
|  |  | rs9300744 | C | T | 0.179 | -0.059 | 0.013 | 4.75E-06 | 0.022 | 0.028 | 0.433 | 0.001 | | 18.699 |  |
| ebi-a-GCST90017049 | Gut microbiota abundance (genus Ruminiclostridium5 id.11355) | rs10827477 | A | G | 0.352 | -0.055 | 0.012 | 2.19E-06 | -0.047 | 0.022 | 0.033 | 0.001 | | 25.110 |  |
|  |  | rs113753996 | T | C | 0.183 | 0.082 | 0.017 | 3.99E-06 | -0.060 | 0.027 | 0.028 | 0.002 | | 36.929 |  |
|  |  | rs1223978 | C | T | 0.531 | -0.048 | 0.011 | 8.16E-06 | 0.011 | 0.021 | 0.613 | 0.001 | | 21.438 |  |
|  |  | rs1492620 | T | C | 0.131 | -0.083 | 0.018 | 3.53E-06 | -0.016 | 0.032 | 0.613 | 0.002 | | 28.899 |  |
|  |  | rs2482038 | C | A | 0.402 | 0.052 | 0.011 | 1.70E-06 | -0.020 | 0.022 | 0.361 | 0.001 | | 23.773 |  |
|  |  | rs2791343 | T | C | 0.397 | 0.052 | 0.011 | 5.54E-06 | 0.010 | 0.022 | 0.640 | 0.001 | | 23.511 |  |
|  |  | rs2833828 | G | A | 0.412 | 0.049 | 0.011 | 6.82E-06 | 0.018 | 0.022 | 0.400 | 0.001 | | 21.323 |  |
|  |  | rs4955951 | A | G | 0.117 | -0.071 | 0.017 | 9.96E-06 | -0.066 | 0.033 | 0.044 | 0.001 | | 19.309 |  |
|  |  | rs6121460 | G | A | 0.082 | 0.093 | 0.020 | 2.64E-06 | -0.040 | 0.039 | 0.303 | 0.001 | | 24.158 |  |
|  |  | rs79968837 | A | G | 0.051 | -0.095 | 0.019 | 1.15E-06 | 0.012 | 0.048 | 0.807 | 0.001 | | 15.896 |  |
|  |  | rs8053158 | G | A | 0.878 | 0.074 | 0.016 | 5.90E-06 | 0.075 | 0.033 | 0.021 | 0.001 | | 21.555 |  |
| ebi-a-GCST90017050 | Gut microbiota abundance (genus Ruminiclostridium6 id.11356) | rs10829821 | T | C | 0.090 | -0.098 | 0.022 | 3.47E-06 | 0.033 | 0.037 | 0.376 | 0.002 | | 28.765 |  |
|  |  | rs116969552 | A | G | 0.029 | -0.167 | 0.038 | 9.16E-06 | 0.070 | 0.064 | 0.271 | 0.002 | | 28.565 |  |
|  |  | rs11992182 | A | C | 0.223 | 0.063 | 0.014 | 4.65E-06 | 0.009 | 0.025 | 0.736 | 0.001 | | 24.849 |  |
|  |  | rs2548459 | C | T | 0.410 | 0.055 | 0.012 | 6.40E-06 | 0.009 | 0.021 | 0.692 | 0.001 | | 27.347 |  |
|  |  | rs35362464 | C | A | 0.140 | 0.072 | 0.017 | 8.99E-06 | -0.014 | 0.031 | 0.637 | 0.001 | | 22.966 |  |
|  |  | rs61060922 | T | G | 0.033 | 0.159 | 0.032 | 1.09E-06 | -0.098 | 0.059 | 0.097 | 0.002 | | 29.998 |  |
|  |  | rs663262 | C | T | 0.967 | 0.135 | 0.031 | 3.39E-06 | 0.027 | 0.059 | 0.652 | 0.001 | | 21.411 |  |
|  |  | rs67479537 | T | C | 0.048 | 0.119 | 0.026 | 9.30E-06 | -0.056 | 0.050 | 0.260 | 0.001 | | 23.644 |  |
|  |  | rs71414120 | T | G | 0.051 | 0.201 | 0.041 | 1.08E-06 | 0.066 | 0.048 | 0.175 | 0.004 | | 72.068 |  |
|  |  | rs72991535 | T | G | 0.036 | 0.136 | 0.030 | 4.95E-06 | 0.012 | 0.058 | 0.841 | 0.001 | | 23.127 |  |
|  |  | rs73176030 | T | C | 0.272 | 0.059 | 0.013 | 7.29E-06 | 0.016 | 0.024 | 0.488 | 0.001 | | 25.040 |  |
|  |  | rs77193512 | A | G | 0.248 | 0.074 | 0.015 | 1.30E-06 | 0.028 | 0.025 | 0.260 | 0.002 | | 37.160 |  |
|  |  | rs792058 | G | A | 0.437 | 0.055 | 0.013 | 8.58E-06 | 0.016 | 0.022 | 0.445 | 0.002 | | 27.753 |  |
|  |  | rs79968172 | G | A | 0.059 | 0.116 | 0.024 | 1.66E-06 | 0.006 | 0.045 | 0.898 | 0.001 | | 27.520 |  |
|  |  | rs9555756 | A | C | 0.084 | -0.080 | 0.018 | 7.10E-06 | 0.032 | 0.038 | 0.402 | 0.001 | | 18.349 |  |
| ebi-a-GCST90017051 | Gut microbiota abundance (genus Ruminiclostridium9 id.11357) | rs12040548 | G | T | 0.270 | 0.057 | 0.012 | 3.15E-06 | 0.010 | 0.024 | 0.662 | 0.001 | | 23.551 |  |
|  |  | rs6082461 | C | A | 0.794 | -0.059 | 0.013 | 4.87E-06 | -0.026 | 0.026 | 0.318 | 0.001 | | 20.646 |  |
|  |  | rs7137760 | C | T | 0.461 | 0.051 | 0.011 | 7.07E-06 | 0.017 | 0.021 | 0.433 | 0.001 | | 23.548 |  |
|  |  | rs74303178 | T | C | 0.317 | 0.053 | 0.012 | 7.92E-06 | -0.002 | 0.023 | 0.929 | 0.001 | | 22.557 |  |
|  |  | rs78191726 | T | C | 0.073 | 0.094 | 0.021 | 7.58E-06 | -0.067 | 0.041 | 0.102 | 0.001 | | 22.165 |  |
|  |  | rs918449 | G | A | 0.928 | 0.095 | 0.020 | 2.56E-06 | -0.006 | 0.041 | 0.881 | 0.001 | | 22.128 |  |
|  |  | rs9522712 | T | C | 0.149 | 0.070 | 0.015 | 4.66E-06 | -0.006 | 0.030 | 0.842 | 0.001 | | 22.823 |  |
|  |  | rs9809789 | C | T | 0.183 | -0.072 | 0.016 | 8.72E-06 | -0.009 | 0.028 | 0.750 | 0.002 | | 28.233 |  |
| ebi-a-GCST90017052 | Gut microbiota abundance (genus Ruminococcaceae NK4A214 group id.11358) | rs11241747 | T | C | 0.691 | -0.053 | 0.012 | 6.59E-06 | -0.017 | 0.023 | 0.455 | 0.001 | | 22.334 |  |
|  |  | rs11586410 | G | A | 0.158 | -0.086 | 0.017 | 3.66E-07 | -0.058 | 0.029 | 0.049 | 0.002 | | 36.428 |  |
|  |  | rs12642039 | C | T | 0.637 | 0.055 | 0.012 | 3.43E-06 | 0.012 | 0.022 | 0.591 | 0.001 | | 25.978 |  |
|  |  | rs12731 | A | G | 0.379 | -0.053 | 0.012 | 4.87E-06 | 0.021 | 0.022 | 0.326 | 0.001 | | 24.075 |  |
|  |  | rs13087692 | G | T | 0.703 | -0.057 | 0.013 | 8.69E-06 | -0.030 | 0.023 | 0.197 | 0.001 | | 25.315 |  |
|  |  | rs136761 | A | G | 0.637 | 0.059 | 0.012 | 8.15E-07 | -0.023 | 0.022 | 0.292 | 0.002 | | 29.313 |  |
|  |  | rs147475196 | A | G | 0.107 | -0.134 | 0.030 | 4.72E-06 | -0.015 | 0.035 | 0.664 | 0.003 | | 62.872 |  |
|  |  | rs35559912 | T | C | 0.121 | -0.093 | 0.020 | 4.89E-06 | 0.027 | 0.033 | 0.404 | 0.002 | | 33.442 |  |
|  |  | rs4814689 | C | T | 0.045 | -0.108 | 0.023 | 4.55E-06 | 0.056 | 0.051 | 0.271 | 0.001 | | 18.474 |  |
|  |  | rs5994253 | A | G | 0.142 | -0.081 | 0.016 | 2.35E-07 | 0.011 | 0.030 | 0.724 | 0.002 | | 29.442 |  |
|  |  | rs62027366 | T | C | 0.203 | 0.062 | 0.014 | 6.58E-06 | 0.019 | 0.026 | 0.478 | 0.001 | | 22.458 |  |
|  |  | rs6681678 | T | C | 0.966 | 0.100 | 0.024 | 9.05E-06 | 0.003 | 0.059 | 0.955 | 0.001 | | 11.958 |  |
|  |  | rs7573569 | T | C | 0.061 | 0.108 | 0.023 | 3.23E-06 | -0.041 | 0.044 | 0.356 | 0.001 | | 24.425 |  |
| ebi-a-GCST90017053 | Gut microbiota abundance (genus Ruminococcaceae UCG002 id.11360) | rs10916131 | C | T | 0.161 | -0.069 | 0.015 | 2.87E-06 | 0.017 | 0.029 | 0.553 | 0.001 | | 23.894 |  |
|  |  | rs10927423 | C | A | 0.178 | -0.071 | 0.015 | 8.50E-07 | -0.007 | 0.028 | 0.794 | 0.001 | | 27.393 |  |
|  |  | rs10964441 | G | A | 0.105 | -0.149 | 0.034 | 7.45E-06 | -0.030 | 0.035 | 0.385 | 0.004 | | 76.577 |  |
|  |  | rs113147300 | A | G | 0.137 | -0.076 | 0.016 | 7.69E-06 | -0.032 | 0.031 | 0.293 | 0.001 | | 24.976 |  |
|  |  | rs11607472 | A | G | 0.068 | -0.078 | 0.018 | 7.19E-06 | 0.005 | 0.042 | 0.899 | 0.001 | | 14.107 |  |
|  |  | rs116974815 | C | A | 0.069 | -0.190 | 0.040 | 2.03E-06 | 0.098 | 0.042 | 0.021 | 0.005 | | 85.068 |  |
|  |  | rs11750293 | G | T | 0.370 | -0.058 | 0.012 | 1.76E-06 | -0.009 | 0.022 | 0.674 | 0.002 | | 28.646 |  |
|  |  | rs12463378 | A | G | 0.302 | -0.052 | 0.011 | 2.96E-06 | -0.035 | 0.023 | 0.124 | 0.001 | | 21.103 |  |
|  |  | rs15256 | C | T | 0.118 | 0.073 | 0.017 | 9.46E-06 | -0.063 | 0.033 | 0.054 | 0.001 | | 20.452 |  |
|  |  | rs55793120 | T | C | 0.062 | 0.137 | 0.027 | 4.81E-07 | 0.021 | 0.045 | 0.638 | 0.002 | | 40.122 |  |
|  |  | rs56030423 | G | A | 0.072 | -0.098 | 0.022 | 6.30E-06 | -0.054 | 0.041 | 0.188 | 0.001 | | 23.649 |  |
|  |  | rs57079348 | T | G | 0.057 | -0.077 | 0.017 | 7.22E-06 | 0.003 | 0.045 | 0.948 | 0.001 | | 11.589 |  |
|  |  | rs6542556 | G | A | 0.619 | -0.051 | 0.011 | 7.86E-06 | 0.034 | 0.022 | 0.120 | 0.001 | | 22.505 |  |
|  |  | rs6793778 | T | C | 0.740 | 0.056 | 0.013 | 9.81E-06 | -0.011 | 0.024 | 0.646 | 0.001 | | 22.064 |  |
|  |  | rs7120052 | A | C | 0.189 | 0.062 | 0.014 | 1.97E-06 | -0.048 | 0.027 | 0.075 | 0.001 | | 21.945 |  |
|  |  | rs7155595 | C | A | 0.302 | 0.057 | 0.012 | 1.15E-06 | -0.009 | 0.023 | 0.704 | 0.001 | | 25.137 |  |
|  |  | rs7249614 | G | A | 0.608 | 0.049 | 0.011 | 9.07E-06 | 0.002 | 0.022 | 0.924 | 0.001 | | 21.243 |  |
|  |  | rs7342369 | A | C | 0.744 | 0.053 | 0.012 | 5.66E-06 | 0.004 | 0.024 | 0.875 | 0.001 | | 19.505 |  |
|  |  | rs76847269 | A | G | 0.026 | 0.164 | 0.036 | 5.17E-06 | 0.064 | 0.067 | 0.337 | 0.001 | | 24.743 |  |
|  |  | rs77564310 | A | C | 0.207 | -0.071 | 0.014 | 3.29E-07 | -0.060 | 0.026 | 0.022 | 0.002 | | 30.687 |  |
|  |  | rs79016051 | C | T | 0.132 | -0.089 | 0.019 | 2.34E-06 | 0.004 | 0.032 | 0.909 | 0.002 | | 33.070 |  |
|  |  | rs882348 | A | G | 0.117 | -0.080 | 0.018 | 5.45E-06 | 0.034 | 0.033 | 0.295 | 0.001 | | 24.261 |  |
| ebi-a-GCST90017054 | Gut microbiota abundance (genus Ruminococcaceae UCG003 id.11361) | rs10490280 | C | T | 0.188 | -0.067 | 0.014 | 4.16E-06 | -0.022 | 0.027 | 0.413 | 0.001 | | 25.336 |  |
|  |  | rs11243416 | T | C | 0.070 | -0.093 | 0.019 | 1.67E-06 | 0.029 | 0.042 | 0.484 | 0.001 | | 20.380 |  |
|  |  | rs11613919 | G | T | 0.221 | 0.073 | 0.016 | 1.63E-06 | 0.027 | 0.026 | 0.299 | 0.002 | | 33.467 |  |
|  |  | rs16959793 | A | C | 0.414 | -0.063 | 0.013 | 2.22E-06 | -0.021 | 0.022 | 0.327 | 0.002 | | 34.863 |  |
|  |  | rs2523124 | C | T | 0.599 | 0.055 | 0.012 | 5.78E-06 | -0.035 | 0.022 | 0.105 | 0.001 | | 26.367 |  |
|  |  | rs3013089 | G | A | 0.373 | -0.055 | 0.012 | 4.38E-06 | -0.016 | 0.022 | 0.459 | 0.001 | | 26.134 |  |
|  |  | rs4452755 | A | C | 0.338 | -0.063 | 0.013 | 3.29E-06 | -0.007 | 0.022 | 0.748 | 0.002 | | 33.099 |  |
|  |  | rs4532474 | G | A | 0.163 | 0.077 | 0.017 | 4.82E-06 | -0.014 | 0.029 | 0.616 | 0.002 | | 29.700 |  |
|  |  | rs646327 | G | A | 0.409 | 0.059 | 0.012 | 7.83E-07 | 0.007 | 0.021 | 0.738 | 0.002 | | 30.564 |  |
|  |  | rs6759615 | A | G | 0.098 | 0.103 | 0.020 | 7.86E-07 | 0.040 | 0.035 | 0.259 | 0.002 | | 34.034 |  |
|  |  | rs73341549 | T | C | 0.057 | -0.170 | 0.032 | 1.51E-07 | 0.042 | 0.045 | 0.353 | 0.003 | | 57.092 |  |
|  |  | rs78720113 | A | G | 0.079 | -0.115 | 0.025 | 7.59E-06 | 0.005 | 0.040 | 0.899 | 0.002 | | 35.388 |  |
| ebi-a-GCST90017055 | Gut microbiota abundance (genus Ruminococcaceae UCG004 id.11362) | rs10976229 | T | G | 0.129 | 0.096 | 0.021 | 7.04E-06 | 0.039 | 0.032 | 0.221 | 0.002 | | 38.113 |  |
|  |  | rs11961899 | G | A | 0.279 | -0.071 | 0.016 | 9.18E-06 | 0.031 | 0.024 | 0.191 | 0.002 | | 37.022 |  |
|  |  | rs12125734 | G | T | 0.093 | 0.134 | 0.026 | 2.09E-07 | 0.068 | 0.036 | 0.063 | 0.003 | | 55.948 |  |
|  |  | rs2248146 | T | C | 0.345 | 0.069 | 0.015 | 8.20E-06 | -0.011 | 0.022 | 0.629 | 0.002 | | 39.475 |  |
|  |  | rs3800154 | A | C | 0.265 | -0.080 | 0.018 | 6.12E-06 | -0.017 | 0.024 | 0.470 | 0.002 | | 45.544 |  |
|  |  | rs511258 | G | A | 0.187 | -0.076 | 0.016 | 4.52E-06 | -0.040 | 0.027 | 0.142 | 0.002 | | 32.022 |  |
|  |  | rs550351 | A | C | 0.425 | 0.079 | 0.018 | 9.43E-06 | 0.013 | 0.021 | 0.559 | 0.003 | | 55.520 |  |
|  |  | rs6769553 | A | G | 0.262 | 0.085 | 0.016 | 7.91E-08 | -0.041 | 0.024 | 0.086 | 0.003 | | 51.285 |  |
|  |  | rs7569771 | A | G | 0.248 | -0.076 | 0.017 | 8.12E-06 | 0.005 | 0.025 | 0.842 | 0.002 | | 39.503 |  |
|  |  | rs872501 | G | A | 0.099 | 0.116 | 0.026 | 5.81E-06 | -0.068 | 0.040 | 0.092 | 0.002 | | 44.362 |  |
|  |  | rs9818949 | T | G | 0.797 | -0.086 | 0.019 | 5.39E-06 | 0.003 | 0.027 | 0.900 | 0.002 | | 44.001 |  |
| ebi-a-GCST90017056 | Gut microbiota abundance (genus Ruminococcaceae UCG005 id.11363) | rs10873449 | C | T | 0.808 | -0.065 | 0.014 | 4.11E-06 | 0.004 | 0.027 | 0.888 | 0.001 | | 24.470 |  |
|  |  | rs10937802 | A | G | 0.880 | -0.076 | 0.017 | 8.17E-06 | 0.005 | 0.033 | 0.889 | 0.001 | | 22.199 |  |
|  |  | rs10950694 | C | T | 0.641 | -0.058 | 0.011 | 4.30E-07 | 0.010 | 0.022 | 0.641 | 0.002 | | 28.193 |  |
|  |  | rs114279581 | A | G | 0.075 | -0.147 | 0.032 | 3.22E-06 | -0.042 | 0.040 | 0.287 | 0.003 | | 55.005 |  |
|  |  | rs12288512 | A | G | 0.239 | 0.067 | 0.014 | 3.10E-06 | -0.009 | 0.025 | 0.722 | 0.002 | | 29.692 |  |
|  |  | rs12458218 | T | C | 0.180 | 0.068 | 0.014 | 2.41E-06 | 0.011 | 0.027 | 0.690 | 0.001 | | 24.879 |  |
|  |  | rs2893871 | G | A | 0.110 | -0.074 | 0.016 | 3.54E-06 | -0.017 | 0.034 | 0.614 | 0.001 | | 19.494 |  |
|  |  | rs34781347 | G | A | 0.071 | 0.189 | 0.039 | 6.05E-07 | 0.040 | 0.042 | 0.337 | 0.005 | | 86.905 |  |
|  |  | rs55793120 | T | C | 0.062 | 0.122 | 0.028 | 7.37E-06 | 0.021 | 0.045 | 0.638 | 0.002 | | 31.385 |  |
|  |  | rs72776570 | C | A | 0.098 | 0.087 | 0.020 | 5.36E-06 | 0.014 | 0.036 | 0.701 | 0.001 | | 24.513 |  |
|  |  | rs7449320 | C | A | 0.233 | 0.060 | 0.013 | 4.81E-06 | 0.013 | 0.025 | 0.608 | 0.001 | | 23.546 |  |
|  |  | rs7555878 | G | A | 0.753 | -0.059 | 0.013 | 2.81E-06 | -0.023 | 0.025 | 0.341 | 0.001 | | 23.509 |  |
|  |  | rs7586445 | G | A | 0.129 | 0.078 | 0.018 | 8.81E-06 | 0.005 | 0.032 | 0.887 | 0.001 | | 25.274 |  |
|  |  | rs898577 | C | T | 0.943 | 0.123 | 0.029 | 7.46E-06 | -0.027 | 0.046 | 0.562 | 0.002 | | 29.929 |  |
| ebi-a-GCST90017057 | Gut microbiota abundance (genus Ruminococcaceae UCG009 id.11366) | rs113006825 | T | C | 0.217 | -0.093 | 0.021 | 7.98E-06 | 0.043 | 0.026 | 0.101 | 0.003 | | 53.891 |  |
|  |  | rs12508214 | C | T | 0.339 | -0.077 | 0.017 | 4.75E-06 | -0.005 | 0.023 | 0.827 | 0.003 | | 49.421 |  |
|  |  | rs138460696 | A | G | 0.077 | 0.139 | 0.032 | 9.81E-06 | 0.000 | 0.040 | 0.997 | 0.003 | | 50.670 |  |
|  |  | rs1550196 | A | G | 0.900 | -0.131 | 0.026 | 1.13E-06 | 0.068 | 0.036 | 0.055 | 0.003 | | 56.482 |  |
|  |  | rs2058609 | G | A | 0.723 | -0.082 | 0.017 | 3.12E-06 | 0.002 | 0.024 | 0.933 | 0.003 | | 49.084 |  |
|  |  | rs2192926 | A | G | 0.328 | -0.089 | 0.019 | 4.88E-06 | -0.008 | 0.023 | 0.728 | 0.003 | | 64.367 |  |
|  |  | rs4079028 | C | T | 0.240 | 0.092 | 0.020 | 3.28E-06 | -0.044 | 0.025 | 0.076 | 0.003 | | 56.176 |  |
|  |  | rs4708333 | T | G | 0.341 | -0.084 | 0.017 | 1.56E-06 | 0.032 | 0.022 | 0.153 | 0.003 | | 58.352 |  |
|  |  | rs6952765 | G | A | 0.318 | 0.073 | 0.017 | 8.13E-06 | -0.021 | 0.023 | 0.348 | 0.002 | | 42.760 |  |
|  |  | rs758191 | T | G | 0.102 | 0.177 | 0.038 | 9.01E-06 | -0.012 | 0.036 | 0.746 | 0.006 | | 105.785 |  |
|  |  | rs78410648 | A | G | 0.109 | 0.121 | 0.028 | 9.67E-06 | 0.038 | 0.034 | 0.261 | 0.003 | | 52.374 |  |
|  |  | rs9558661 | T | C | 0.204 | -0.090 | 0.020 | 7.01E-06 | 0.000 | 0.027 | 0.993 | 0.003 | | 48.116 |  |
| ebi-a-GCST90017058 | Gut microbiota abundance (genus Ruminococcaceae UCG010 id.11367) | rs12597105 | A | G | 0.811 | -0.067 | 0.014 | 4.87E-06 | -0.012 | 0.027 | 0.666 | 0.001 | | 25.314 |  |
|  |  | rs2820282 | C | A | 0.599 | 0.059 | 0.013 | 2.85E-06 | -0.027 | 0.022 | 0.219 | 0.002 | | 30.950 |  |
|  |  | rs682403 | A | G | 0.527 | -0.059 | 0.012 | 2.37E-06 | -0.058 | 0.021 | 0.006 | 0.002 | | 31.679 |  |
|  |  | rs6958419 | C | T | 0.465 | -0.059 | 0.012 | 2.84E-06 | 0.019 | 0.021 | 0.381 | 0.002 | | 31.355 |  |
|  |  | rs73218807 | G | A | 0.081 | -0.166 | 0.037 | 6.43E-06 | -0.082 | 0.039 | 0.034 | 0.004 | | 76.085 |  |
|  |  | rs7441445 | T | C | 0.487 | 0.057 | 0.013 | 6.80E-06 | 0.040 | 0.021 | 0.061 | 0.002 | | 29.766 |  |
| ebi-a-GCST90017059 | Gut microbiota abundance (genus Ruminococcaceae UCG011 id.11368) | rs10274562 | C | T | 0.380 | 0.111 | 0.024 | 6.50E-06 | -0.016 | 0.022 | 0.475 | 0.006 | | 106.947 |  |
|  |  | rs12636310 | G | A | 0.252 | 0.133 | 0.028 | 2.81E-06 | -0.011 | 0.025 | 0.645 | 0.007 | | 122.435 |  |
|  |  | rs12724320 | C | T | 0.382 | -0.121 | 0.025 | 1.52E-06 | 0.061 | 0.022 | 0.005 | 0.007 | | 127.447 |  |
|  |  | rs1416041 | A | C | 0.209 | -0.182 | 0.034 | 7.04E-08 | -0.004 | 0.026 | 0.876 | 0.011 | | 203.465 |  |
|  |  | rs2729556 | T | C | 0.523 | 0.109 | 0.023 | 3.19E-06 | -0.019 | 0.021 | 0.364 | 0.006 | | 109.555 |  |
|  |  | rs4490371 | T | C | 0.413 | -0.112 | 0.025 | 7.75E-06 | 0.013 | 0.022 | 0.562 | 0.006 | | 111.854 |  |
|  |  | rs79113084 | C | T | 0.108 | -0.152 | 0.032 | 2.06E-06 | 0.013 | 0.035 | 0.717 | 0.004 | | 82.244 |  |
|  |  | rs9729514 | G | A | 0.907 | -0.185 | 0.039 | 2.37E-06 | -0.018 | 0.036 | 0.618 | 0.006 | | 106.522 |  |
| ebi-a-GCST90017060 | Gut microbiota abundance (genus Ruminococcaceae UCG013 id.11370) | rs11581881 | C | T | 0.237 | 0.066 | 0.014 | 4.73E-06 | -0.034 | 0.025 | 0.183 | 0.002 | | 29.016 |  |
|  |  | rs12189346 | G | A | 0.187 | 0.068 | 0.015 | 1.68E-06 | -0.004 | 0.027 | 0.871 | 0.001 | | 26.176 |  |
|  |  | rs12336782 | T | C | 0.078 | -0.086 | 0.019 | 8.60E-06 | -0.033 | 0.040 | 0.403 | 0.001 | | 19.295 |  |
|  |  | rs12485353 | G | A | 0.248 | -0.061 | 0.013 | 4.19E-06 | 0.000 | 0.025 | 0.989 | 0.001 | | 25.298 |  |
|  |  | rs12781711 | C | T | 0.250 | -0.066 | 0.012 | 2.55E-08 | -0.027 | 0.024 | 0.259 | 0.002 | | 29.637 |  |
|  |  | rs16918863 | A | C | 0.063 | 0.111 | 0.024 | 4.15E-06 | 0.073 | 0.043 | 0.091 | 0.001 | | 27.067 |  |
|  |  | rs2730183 | G | A | 0.399 | -0.049 | 0.011 | 8.44E-06 | 0.025 | 0.022 | 0.246 | 0.001 | | 21.024 |  |
|  |  | rs4385846 | T | G | 0.806 | -0.060 | 0.013 | 6.46E-06 | 0.027 | 0.027 | 0.310 | 0.001 | | 20.551 |  |
|  |  | rs75088940 | T | C | 0.071 | -0.094 | 0.020 | 2.55E-06 | 0.018 | 0.042 | 0.671 | 0.001 | | 21.440 |  |
|  |  | rs76973485 | G | T | 0.041 | 0.195 | 0.042 | 3.35E-06 | -0.104 | 0.054 | 0.051 | 0.003 | | 54.354 |  |
|  |  | rs7784330 | A | G | 0.643 | 0.050 | 0.011 | 8.16E-06 | -0.014 | 0.022 | 0.520 | 0.001 | | 20.923 |  |
|  |  | rs9313055 | T | C | 0.081 | 0.105 | 0.023 | 9.55E-06 | -0.002 | 0.039 | 0.953 | 0.002 | | 30.220 |  |
| ebi-a-GCST90017061 | Gut microbiota abundance (genus Ruminococcaceae UCG014 id.11371) | rs10495392 | T | C | 0.927 | 0.082 | 0.019 | 9.96E-06 | 0.016 | 0.040 | 0.697 | 0.001 | | 16.988 |  |
|  |  | rs10791168 | G | A | 0.815 | 0.066 | 0.015 | 9.76E-06 | 0.003 | 0.028 | 0.915 | 0.001 | | 24.439 |  |
|  |  | rs10941294 | C | T | 0.054 | -0.122 | 0.026 | 2.40E-06 | 0.021 | 0.047 | 0.653 | 0.002 | | 28.096 |  |
|  |  | rs115777838 | T | C | 0.113 | -0.188 | 0.039 | 4.62E-07 | 0.027 | 0.034 | 0.424 | 0.007 | | 131.550 |  |
|  |  | rs12638134 | T | G | 0.417 | 0.058 | 0.012 | 1.21E-06 | -0.005 | 0.021 | 0.825 | 0.002 | | 30.300 |  |
|  |  | rs34402072 | C | T | 0.147 | -0.069 | 0.016 | 9.80E-06 | 0.050 | 0.030 | 0.097 | 0.001 | | 21.746 |  |
|  |  | rs56105232 | G | A | 0.063 | 0.139 | 0.030 | 2.91E-06 | -0.013 | 0.045 | 0.769 | 0.002 | | 41.831 |  |
|  |  | rs72809222 | T | C | 0.212 | 0.067 | 0.014 | 2.41E-06 | 0.004 | 0.026 | 0.878 | 0.002 | | 27.672 |  |
|  |  | rs73186226 | G | A | 0.075 | -0.099 | 0.022 | 6.72E-06 | -0.034 | 0.041 | 0.411 | 0.001 | | 25.287 |  |
|  |  | rs853612 | A | G | 0.396 | -0.053 | 0.012 | 9.75E-06 | -0.046 | 0.022 | 0.034 | 0.001 | | 24.502 |  |
|  |  | rs995642 | C | T | 0.239 | 0.060 | 0.013 | 1.90E-06 | -0.001 | 0.025 | 0.978 | 0.001 | | 24.091 |  |
| ebi-a-GCST90017062 | Gut microbiota abundance (genus Ruminococcus1 id.11373) | rs10167839 | A | G | 0.361 | 0.052 | 0.012 | 8.09E-06 | -0.012 | 0.022 | 0.573 | 0.001 | | 22.859 |  |
|  |  | rs11783695 | G | T | 0.159 | -0.073 | 0.016 | 4.73E-06 | -0.009 | 0.029 | 0.751 | 0.001 | | 26.482 |  |
|  |  | rs17781867 | C | T | 0.069 | 0.100 | 0.021 | 1.96E-06 | -0.023 | 0.042 | 0.577 | 0.001 | | 23.711 |  |
|  |  | rs3819978 | C | T | 0.078 | -0.115 | 0.026 | 8.74E-06 | 0.142 | 0.040 | 0.000 | 0.002 | | 34.821 |  |
|  |  | rs6105066 | T | C | 0.277 | -0.061 | 0.013 | 5.06E-06 | -0.047 | 0.024 | 0.050 | 0.001 | | 27.077 |  |
|  |  | rs6493760 | T | C | 0.643 | -0.054 | 0.012 | 3.38E-06 | -0.009 | 0.022 | 0.691 | 0.001 | | 24.156 |  |
|  |  | rs7117576 | A | G | 0.091 | 0.083 | 0.017 | 6.48E-07 | 0.024 | 0.037 | 0.511 | 0.001 | | 20.963 |  |
|  |  | rs7583465 | C | T | 0.440 | 0.053 | 0.011 | 2.56E-06 | -0.003 | 0.021 | 0.875 | 0.001 | | 25.190 |  |
|  |  | rs78572139 | G | A | 0.102 | 0.125 | 0.028 | 5.23E-06 | -0.023 | 0.035 | 0.525 | 0.003 | | 52.719 |  |
|  |  | rs78613526 | G | A | 0.053 | 0.167 | 0.037 | 5.11E-06 | -0.104 | 0.049 | 0.034 | 0.003 | | 51.392 |  |
| ebi-a-GCST90017063 | Gut microbiota abundance (genus Ruminococcus2 id.11374) | rs12406309 | A | C | 0.221 | -0.063 | 0.014 | 9.79E-06 | -0.019 | 0.026 | 0.451 | 0.001 | | 25.249 |  |
|  |  | rs12986628 | T | C | 0.807 | -0.067 | 0.014 | 2.14E-06 | -0.043 | 0.027 | 0.110 | 0.001 | | 25.348 |  |
|  |  | rs1819812 | G | T | 0.053 | 0.084 | 0.018 | 5.28E-06 | 0.074 | 0.048 | 0.120 | 0.001 | | 13.085 |  |
|  |  | rs2368224 | T | G | 0.049 | 0.200 | 0.044 | 3.63E-06 | -0.066 | 0.049 | 0.185 | 0.004 | | 67.871 |  |
|  |  | rs2846589 | T | G | 0.576 | -0.052 | 0.012 | 7.59E-06 | 0.067 | 0.022 | 0.002 | 0.001 | | 24.453 |  |
|  |  | rs2997412 | G | A | 0.720 | 0.057 | 0.012 | 4.22E-06 | 0.010 | 0.024 | 0.674 | 0.001 | | 23.912 |  |
|  |  | rs4400279 | A | G | 0.324 | 0.055 | 0.012 | 5.80E-06 | 0.043 | 0.023 | 0.059 | 0.001 | | 23.969 |  |
|  |  | rs4799823 | C | T | 0.186 | 0.084 | 0.018 | 5.40E-06 | -0.009 | 0.028 | 0.748 | 0.002 | | 39.049 |  |
|  |  | rs55707116 | C | A | 0.075 | 0.087 | 0.019 | 8.01E-06 | 0.025 | 0.040 | 0.535 | 0.001 | | 19.050 |  |
|  |  | rs58681734 | A | G | 0.212 | 0.072 | 0.016 | 4.18E-06 | -0.004 | 0.026 | 0.872 | 0.002 | | 32.206 |  |
|  |  | rs61791565 | T | C | 0.506 | -0.052 | 0.012 | 6.79E-06 | 0.012 | 0.021 | 0.581 | 0.001 | | 25.159 |  |
|  |  | rs75140805 | T | G | 0.180 | 0.084 | 0.018 | 3.95E-06 | 0.008 | 0.028 | 0.769 | 0.002 | | 38.037 |  |
|  |  | rs7635831 | G | A | 0.377 | 0.062 | 0.013 | 1.98E-06 | -0.001 | 0.022 | 0.958 | 0.002 | | 33.006 |  |
|  |  | rs7693984 | G | A | 0.044 | -0.103 | 0.024 | 9.42E-06 | -0.017 | 0.051 | 0.743 | 0.001 | | 16.452 |  |
|  |  | rs78120384 | A | G | 0.093 | -0.193 | 0.039 | 3.31E-07 | 0.006 | 0.037 | 0.880 | 0.006 | | 115.415 |  |
| ebi-a-GCST90017064 | Gut microbiota abundance (genus Ruminococcus gauvreauii group id.11342) | rs10931481 | A | G | 0.711 | -0.061 | 0.013 | 3.38E-06 | 0.012 | 0.023 | 0.617 | 0.002 | | 28.094 |  |
|  |  | rs12079579 | A | G | 0.085 | 0.096 | 0.021 | 5.04E-06 | 0.012 | 0.038 | 0.753 | 0.001 | | 25.947 |  |
|  |  | rs12539819 | C | T | 0.069 | 0.111 | 0.024 | 4.49E-06 | 0.021 | 0.043 | 0.620 | 0.002 | | 28.971 |  |
|  |  | rs1391597 | C | T | 0.394 | 0.059 | 0.012 | 1.86E-06 | 0.048 | 0.022 | 0.028 | 0.002 | | 30.571 |  |
|  |  | rs2047242 | A | G | 0.231 | -0.068 | 0.013 | 2.46E-07 | -0.006 | 0.025 | 0.824 | 0.002 | | 29.796 |  |
|  |  | rs2105937 | G | A | 0.652 | -0.058 | 0.013 | 5.10E-06 | -0.003 | 0.022 | 0.888 | 0.002 | | 28.076 |  |
|  |  | rs2166943 | C | A | 0.581 | -0.057 | 0.012 | 5.28E-06 | -0.010 | 0.021 | 0.625 | 0.002 | | 28.752 |  |
|  |  | rs289410 | A | G | 0.723 | 0.065 | 0.014 | 2.27E-06 | -0.002 | 0.024 | 0.930 | 0.002 | | 31.588 |  |
|  |  | rs431418 | G | A | 0.906 | 0.095 | 0.021 | 5.54E-06 | -0.062 | 0.036 | 0.086 | 0.002 | | 28.184 |  |
|  |  | rs71386687 | T | G | 0.117 | 0.121 | 0.024 | 2.91E-07 | -0.046 | 0.034 | 0.169 | 0.003 | | 55.512 |  |
|  |  | rs73802842 | C | A | 0.239 | 0.074 | 0.017 | 7.48E-06 | 0.006 | 0.025 | 0.819 | 0.002 | | 36.265 |  |
|  |  | rs9870933 | G | A | 0.604 | -0.062 | 0.013 | 8.49E-07 | 0.034 | 0.022 | 0.116 | 0.002 | | 33.957 |  |
| ebi-a-GCST90017065 | Gut microbiota abundance (genus Ruminococcus gnavus group id.14376) | rs11597105 | A | G | 0.197 | 0.115 | 0.025 | 6.95E-06 | -0.007 | 0.027 | 0.783 | 0.004 | | 76.700 |  |
|  |  | rs11864644 | T | C | 0.116 | -0.140 | 0.032 | 5.01E-06 | -0.068 | 0.033 | 0.043 | 0.004 | | 73.914 |  |
|  |  | rs12136548 | C | T | 0.287 | 0.090 | 0.020 | 3.10E-06 | -0.002 | 0.023 | 0.920 | 0.003 | | 61.245 |  |
|  |  | rs12989336 | G | A | 0.280 | -0.085 | 0.019 | 7.12E-06 | 0.051 | 0.024 | 0.029 | 0.003 | | 53.181 |  |
|  |  | rs13163520 | G | A | 0.184 | -0.127 | 0.023 | 5.61E-08 | 0.021 | 0.027 | 0.436 | 0.005 | | 89.669 |  |
|  |  | rs2909242 | A | C | 0.669 | 0.091 | 0.018 | 7.41E-07 | -0.014 | 0.023 | 0.533 | 0.004 | | 67.507 |  |
|  |  | rs3124783 | G | A | 0.871 | 0.116 | 0.025 | 2.67E-06 | -0.010 | 0.031 | 0.756 | 0.003 | | 55.683 |  |
|  |  | rs4388134 | T | C | 0.722 | 0.090 | 0.020 | 9.12E-06 | -0.005 | 0.024 | 0.834 | 0.003 | | 60.450 |  |
|  |  | rs62167033 | T | C | 0.041 | 0.185 | 0.040 | 3.50E-06 | -0.117 | 0.053 | 0.027 | 0.003 | | 49.655 |  |
|  |  | rs78399089 | T | C | 0.115 | 0.144 | 0.033 | 6.63E-06 | -0.033 | 0.033 | 0.319 | 0.004 | | 78.094 |  |
|  |  | rs934940 | A | C | 0.133 | -0.105 | 0.023 | 2.74E-06 | 0.011 | 0.031 | 0.735 | 0.003 | | 46.845 |  |
|  |  | rs9872758 | T | C | 0.523 | 0.085 | 0.018 | 1.66E-06 | 0.033 | 0.021 | 0.115 | 0.004 | | 66.227 |  |
| ebi-a-GCST90017066 | Gut microbiota abundance (genus Ruminococcus torques group id.14377) | rs10904297 | A | G | 0.021 | -0.168 | 0.039 | 2.69E-06 | -0.058 | 0.073 | 0.425 | 0.001 | | 21.494 |  |
|  |  | rs10967781 | C | A | 0.289 | 0.051 | 0.011 | 8.37E-06 | 0.005 | 0.023 | 0.831 | 0.001 | | 19.448 |  |
|  |  | rs12434631 | A | G | 0.106 | 0.075 | 0.015 | 2.77E-06 | 0.011 | 0.035 | 0.754 | 0.001 | | 19.381 |  |
|  |  | rs1475330 | C | T | 0.761 | -0.052 | 0.012 | 8.13E-06 | -0.024 | 0.025 | 0.328 | 0.001 | | 18.314 |  |
|  |  | rs35866622 | T | C | 0.339 | -0.061 | 0.011 | 2.21E-08 | 0.000 | 0.022 | 0.996 | 0.002 | | 30.822 |  |
|  |  | rs4073731 | T | C | 0.163 | 0.065 | 0.014 | 4.05E-06 | 0.016 | 0.029 | 0.576 | 0.001 | | 21.250 |  |
|  |  | rs77034621 | T | G | 0.017 | -0.152 | 0.034 | 6.07E-06 | -0.038 | 0.081 | 0.641 | 0.001 | | 13.998 |  |
|  |  | rs8080469 | G | A | 0.490 | 0.049 | 0.011 | 3.50E-06 | 0.009 | 0.021 | 0.663 | 0.001 | | 22.099 |  |
|  |  | rs8141465 | G | A | 0.359 | -0.048 | 0.011 | 9.65E-06 | 0.035 | 0.022 | 0.107 | 0.001 | | 19.539 |  |
| ebi-a-GCST90017067 | Gut microbiota abundance (genus Sellimonas id.14369) | rs113379006 | T | C | 0.176 | -0.163 | 0.036 | 7.21E-06 | 0.072 | 0.028 | 0.011 | 0.008 | | 142.119 |  |
|  |  | rs13417181 | T | C | 0.261 | 0.167 | 0.034 | 7.62E-07 | -0.001 | 0.026 | 0.983 | 0.011 | | 198.051 |  |
|  |  | rs2016057 | C | A | 0.614 | 0.126 | 0.026 | 1.03E-06 | -0.059 | 0.022 | 0.007 | 0.008 | | 138.842 |  |
|  |  | rs2187447 | A | C | 0.061 | 0.243 | 0.053 | 3.98E-06 | 0.024 | 0.044 | 0.584 | 0.007 | | 124.573 |  |
|  |  | rs2371572 | A | C | 0.466 | 0.127 | 0.025 | 4.46E-07 | 0.022 | 0.021 | 0.305 | 0.008 | | 149.222 |  |
|  |  | rs41816 | A | G | 0.301 | 0.132 | 0.029 | 8.39E-06 | 0.014 | 0.023 | 0.556 | 0.007 | | 135.863 |  |
|  |  | rs4600608 | G | A | 0.789 | 0.137 | 0.030 | 4.95E-06 | 0.065 | 0.026 | 0.013 | 0.006 | | 115.457 |  |
|  |  | rs553697 | C | T | 0.813 | 0.154 | 0.034 | 6.13E-06 | -0.008 | 0.028 | 0.760 | 0.007 | | 132.958 |  |
|  |  | rs56203279 | T | C | 0.336 | -0.124 | 0.027 | 3.72E-06 | -0.051 | 0.022 | 0.024 | 0.007 | | 126.702 |  |
| ebi-a-GCST90017068 | Gut microbiota abundance (genus Senegalimassilia id.11160) | rs10036909 | C | T | 0.041 | 0.186 | 0.040 | 8.05E-06 | 0.078 | 0.054 | 0.147 | 0.003 | | 49.195 |  |
|  |  | rs11787826 | C | A | 0.409 | 0.081 | 0.017 | 2.63E-06 | 0.018 | 0.021 | 0.409 | 0.003 | | 58.802 |  |
|  |  | rs1990708 | A | C | 0.079 | -0.110 | 0.025 | 8.91E-06 | 0.042 | 0.039 | 0.276 | 0.002 | | 31.954 |  |
|  |  | rs2017373 | C | T | 0.363 | 0.078 | 0.018 | 9.50E-06 | 0.017 | 0.022 | 0.444 | 0.003 | | 52.018 |  |
|  |  | rs7225245 | A | G | 0.571 | -0.079 | 0.017 | 4.18E-06 | -0.015 | 0.022 | 0.493 | 0.003 | | 56.483 |  |
| ebi-a-GCST90017069 | Gut microbiota abundance (genus Slackia id.825) | rs10409783 | G | A | 0.710 | -0.095 | 0.021 | 7.70E-06 | 0.001 | 0.024 | 0.982 | 0.004 | | 68.541 |  |
|  |  | rs12440440 | A | G | 0.342 | 0.090 | 0.019 | 2.63E-06 | 0.033 | 0.022 | 0.135 | 0.004 | | 67.378 |  |
|  |  | rs16894137 | C | T | 0.130 | -0.123 | 0.026 | 2.71E-06 | -0.022 | 0.031 | 0.480 | 0.003 | | 62.881 |  |
|  |  | rs35156985 | T | C | 0.042 | -0.156 | 0.035 | 8.06E-06 | 0.024 | 0.053 | 0.651 | 0.002 | | 35.865 |  |
|  |  | rs4492265 | G | A | 0.696 | 0.091 | 0.019 | 2.41E-06 | 0.012 | 0.023 | 0.610 | 0.003 | | 63.944 |  |
|  |  | rs8901 | C | T | 0.294 | 0.093 | 0.019 | 6.07E-07 | -0.055 | 0.023 | 0.018 | 0.004 | | 66.682 |  |
| ebi-a-GCST90017070 | Gut microbiota abundance (genus Streptococcus id.1853) | rs10028567 | C | T | 0.121 | -0.092 | 0.019 | 7.30E-06 | -0.037 | 0.033 | 0.253 | 0.002 | | 33.089 |  |
|  |  | rs10448310 | A | G | 0.361 | -0.052 | 0.011 | 3.31E-06 | 0.028 | 0.022 | 0.201 | 0.001 | | 22.715 |  |
|  |  | rs11110281 | T | C | 0.048 | -0.138 | 0.023 | 2.58E-09 | 0.005 | 0.049 | 0.922 | 0.002 | | 31.787 |  |
|  |  | rs11720390 | G | A | 0.062 | 0.107 | 0.023 | 3.59E-06 | 0.028 | 0.044 | 0.528 | 0.001 | | 24.452 |  |
|  |  | rs11764382 | A | G | 0.140 | -0.070 | 0.014 | 1.29E-06 | 0.030 | 0.030 | 0.327 | 0.001 | | 21.311 |  |
|  |  | rs17708276 | A | G | 0.109 | -0.079 | 0.017 | 3.04E-06 | -0.057 | 0.036 | 0.111 | 0.001 | | 22.481 |  |
|  |  | rs1918540 | A | G | 0.814 | -0.060 | 0.013 | 2.44E-06 | -0.019 | 0.027 | 0.495 | 0.001 | | 19.813 |  |
|  |  | rs2370083 | G | T | 0.065 | -0.082 | 0.019 | 9.75E-06 | -0.020 | 0.043 | 0.642 | 0.001 | | 14.786 |  |
|  |  | rs4968759 | A | G | 0.486 | -0.052 | 0.011 | 3.78E-06 | 0.000 | 0.021 | 0.993 | 0.001 | | 24.341 |  |
|  |  | rs57646748 | G | A | 0.039 | -0.091 | 0.020 | 5.48E-06 | 0.008 | 0.054 | 0.882 | 0.001 | | 11.229 |  |
|  |  | rs6806351 | T | C | 0.219 | -0.063 | 0.014 | 4.94E-06 | -0.052 | 0.026 | 0.041 | 0.001 | | 25.261 |  |
|  |  | rs71481756 | T | G | 0.066 | 0.093 | 0.021 | 6.51E-06 | -0.020 | 0.043 | 0.640 | 0.001 | | 19.605 |  |
|  |  | rs7916711 | A | G | 0.138 | 0.103 | 0.022 | 2.72E-06 | -0.012 | 0.031 | 0.703 | 0.003 | | 46.360 |  |
|  |  | rs9903102 | C | A | 0.198 | -0.071 | 0.016 | 4.18E-06 | -0.023 | 0.027 | 0.383 | 0.002 | | 29.408 |  |
| ebi-a-GCST90017071 | Gut microbiota abundance (genus Subdoligranulum id.2070) | rs10065321 | T | C | 0.415 | -0.051 | 0.011 | 2.10E-06 | -0.023 | 0.021 | 0.279 | 0.001 | | 23.439 |  |
|  |  | rs10497836 | T | C | 0.788 | 0.052 | 0.012 | 8.38E-06 | 0.066 | 0.026 | 0.011 | 0.001 | | 16.831 |  |
|  |  | rs12590825 | C | A | 0.331 | -0.053 | 0.011 | 2.08E-06 | 0.004 | 0.022 | 0.852 | 0.001 | | 22.433 |  |
|  |  | rs1667315 | G | A | 0.392 | 0.049 | 0.011 | 6.72E-06 | 0.004 | 0.022 | 0.854 | 0.001 | | 20.580 |  |
|  |  | rs2114677 | C | T | 0.116 | -0.104 | 0.023 | 2.72E-06 | -0.032 | 0.033 | 0.337 | 0.002 | | 40.968 |  |
|  |  | rs2171249 | C | T | 0.074 | 0.107 | 0.023 | 4.51E-06 | 0.022 | 0.041 | 0.585 | 0.002 | | 28.731 |  |
|  |  | rs3761728 | G | T | 0.748 | 0.054 | 0.012 | 3.87E-06 | 0.062 | 0.024 | 0.011 | 0.001 | | 20.452 |  |
|  |  | rs4347804 | G | A | 0.968 | -0.166 | 0.036 | 2.18E-06 | -0.038 | 0.060 | 0.524 | 0.002 | | 31.193 |  |
|  |  | rs6555306 | C | T | 0.859 | 0.074 | 0.016 | 2.81E-06 | -0.020 | 0.030 | 0.512 | 0.001 | | 24.481 |  |
|  |  | rs75158211 | T | C | 0.148 | -0.072 | 0.016 | 7.52E-06 | 0.018 | 0.030 | 0.542 | 0.001 | | 24.195 |  |
|  |  | rs76528319 | G | T | 0.082 | -0.143 | 0.031 | 7.41E-06 | -0.011 | 0.039 | 0.767 | 0.003 | | 56.922 |  |
| ebi-a-GCST90017072 | Gut microbiota abundance (genus Sutterella id.2896) | rs1145877 | G | A | 0.862 | 0.074 | 0.016 | 7.20E-06 | 0.028 | 0.031 | 0.364 | 0.001 | | 23.601 |  |
|  |  | rs11591622 | T | G | 0.162 | -0.069 | 0.015 | 6.50E-06 | -0.040 | 0.029 | 0.164 | 0.001 | | 23.624 |  |
|  |  | rs13173038 | A | G | 0.248 | -0.072 | 0.015 | 2.73E-06 | 0.011 | 0.025 | 0.653 | 0.002 | | 35.337 |  |
|  |  | rs143438747 | T | C | 0.076 | -0.146 | 0.031 | 3.28E-06 | -0.071 | 0.040 | 0.078 | 0.003 | | 54.736 |  |
|  |  | rs2050185 | A | G | 0.625 | -0.058 | 0.013 | 7.97E-06 | 0.005 | 0.022 | 0.805 | 0.002 | | 28.484 |  |
|  |  | rs2321387 | G | A | 0.431 | -0.059 | 0.012 | 1.87E-06 | 0.016 | 0.021 | 0.452 | 0.002 | | 31.661 |  |
|  |  | rs2613606 | T | C | 0.582 | 0.056 | 0.012 | 7.20E-06 | 0.012 | 0.021 | 0.590 | 0.002 | | 27.712 |  |
|  |  | rs607327 | T | C | 0.627 | -0.058 | 0.013 | 6.63E-06 | -0.004 | 0.022 | 0.847 | 0.002 | | 28.750 |  |
|  |  | rs62501473 | G | A | 0.264 | 0.069 | 0.015 | 5.52E-06 | 0.062 | 0.024 | 0.010 | 0.002 | | 34.385 |  |
|  |  | rs7499539 | A | G | 0.262 | 0.062 | 0.013 | 2.36E-06 | 0.008 | 0.024 | 0.735 | 0.001 | | 27.072 |  |
|  |  | rs7638039 | T | C | 0.247 | 0.065 | 0.014 | 8.66E-06 | -0.012 | 0.025 | 0.635 | 0.002 | | 28.478 |  |
|  |  | rs9350083 | T | G | 0.355 | -0.059 | 0.013 | 8.23E-06 | -0.009 | 0.022 | 0.677 | 0.002 | | 29.571 |  |
| ebi-a-GCST90017073 | Gut microbiota abundance (genus Terrisporobacter id.11348) | rs1883097 | C | T | 0.038 | 0.226 | 0.045 | 4.16E-07 | 0.023 | 0.055 | 0.677 | 0.004 | | 68.559 |  |
|  |  | rs2569953 | C | A | 0.585 | 0.078 | 0.017 | 8.95E-06 | 0.032 | 0.021 | 0.139 | 0.003 | | 53.726 |  |
|  |  | rs2872237 | A | C | 0.601 | 0.081 | 0.018 | 3.97E-06 | -0.019 | 0.022 | 0.381 | 0.003 | | 58.558 |  |
|  |  | rs58405430 | G | T | 0.060 | 0.135 | 0.030 | 7.94E-06 | 0.093 | 0.044 | 0.037 | 0.002 | | 37.492 |  |
|  |  | rs7184125 | T | C | 0.292 | 0.091 | 0.021 | 8.48E-06 | -0.004 | 0.024 | 0.859 | 0.003 | | 63.361 |  |
| ebi-a-GCST90017074 | Gut microbiota abundance (genus Turicibacter id.2162) | rs11054680 | T | C | 0.172 | -0.105 | 0.023 | 2.31E-06 | 0.029 | 0.028 | 0.308 | 0.003 | | 57.573 |  |
|  |  | rs11666533 | C | T | 0.084 | -0.112 | 0.025 | 7.37E-06 | 0.032 | 0.038 | 0.411 | 0.002 | | 35.427 |  |
|  |  | rs12603364 | T | C | 0.151 | 0.111 | 0.023 | 8.67E-07 | 0.024 | 0.030 | 0.408 | 0.003 | | 57.906 |  |
|  |  | rs149744580 | A | G | 0.053 | 0.170 | 0.032 | 7.01E-08 | 0.008 | 0.047 | 0.870 | 0.003 | | 53.613 |  |
|  |  | rs2834977 | T | C | 0.152 | -0.096 | 0.021 | 3.96E-06 | -0.047 | 0.030 | 0.110 | 0.002 | | 43.647 |  |
|  |  | rs2952020 | A | G | 0.751 | 0.076 | 0.017 | 5.63E-06 | -0.010 | 0.025 | 0.691 | 0.002 | | 39.565 |  |
|  |  | rs3734633 | G | A | 0.058 | -0.121 | 0.027 | 5.32E-06 | 0.015 | 0.045 | 0.742 | 0.002 | | 29.336 |  |
|  |  | rs4869133 | G | A | 0.177 | 0.131 | 0.027 | 2.55E-06 | 0.008 | 0.028 | 0.779 | 0.005 | | 92.532 |  |
|  |  | rs55756211 | T | C | 0.073 | -0.115 | 0.024 | 2.81E-06 | 0.000 | 0.041 | 0.993 | 0.002 | | 32.794 |  |
|  |  | rs7199484 | G | A | 0.306 | -0.073 | 0.016 | 5.77E-06 | 0.034 | 0.023 | 0.143 | 0.002 | | 41.763 |  |
| ebi-a-GCST90017075 | Gut microbiota abundance (genus Tyzzerella3 id.11335) | rs10898797 | C | T | 0.109 | 0.122 | 0.027 | 8.85E-06 | -0.013 | 0.034 | 0.708 | 0.003 | | 53.502 |  |
|  |  | rs112102233 | A | G | 0.047 | -0.216 | 0.048 | 6.18E-06 | 0.027 | 0.050 | 0.594 | 0.004 | | 76.650 |  |
|  |  | rs1232220 | T | G | 0.899 | 0.144 | 0.032 | 7.91E-06 | 0.020 | 0.036 | 0.578 | 0.004 | | 69.310 |  |
|  |  | rs17706273 | T | C | 0.078 | -0.140 | 0.027 | 5.88E-07 | 0.005 | 0.039 | 0.894 | 0.003 | | 52.411 |  |
|  |  | rs191093 | G | A | 0.109 | 0.159 | 0.035 | 6.76E-06 | 0.023 | 0.034 | 0.509 | 0.005 | | 90.136 |  |
|  |  | rs4904512 | T | C | 0.129 | -0.117 | 0.025 | 3.09E-06 | -0.019 | 0.032 | 0.547 | 0.003 | | 56.807 |  |
|  |  | rs55799124 | A | G | 0.277 | -0.114 | 0.024 | 1.34E-06 | -0.040 | 0.024 | 0.096 | 0.005 | | 96.529 |  |
|  |  | rs67476743 | T | G | 0.273 | 0.132 | 0.022 | 3.74E-09 | -0.007 | 0.024 | 0.778 | 0.007 | | 127.946 |  |
|  |  | rs6920448 | C | T | 0.093 | -0.141 | 0.031 | 4.15E-06 | -0.003 | 0.036 | 0.925 | 0.003 | | 61.986 |  |
|  |  | rs7019909 | T | C | 0.121 | 0.144 | 0.030 | 1.76E-06 | 0.011 | 0.033 | 0.746 | 0.004 | | 81.131 |  |
|  |  | rs7333521 | T | C | 0.032 | -0.207 | 0.045 | 4.88E-06 | 0.107 | 0.059 | 0.068 | 0.003 | | 48.989 |  |
|  |  | rs75091807 | G | T | 0.063 | -0.185 | 0.038 | 1.71E-06 | -0.054 | 0.044 | 0.221 | 0.004 | | 73.962 |  |
|  |  | rs7561370 | C | T | 0.850 | -0.131 | 0.029 | 1.52E-06 | 0.016 | 0.030 | 0.593 | 0.004 | | 80.889 |  |
| ebi-a-GCST90017088 | Gut microbiota abundance (genus Veillonella id.2198) | rs1882878 | A | G | 0.298 | -0.077 | 0.016 | 2.98E-06 | -0.017 | 0.023 | 0.453 | 0.002 | | 45.516 |  |
|  |  | rs2013594 | C | T | 0.598 | 0.072 | 0.016 | 3.42E-06 | -0.035 | 0.022 | 0.110 | 0.002 | | 45.913 |  |
|  |  | rs55807413 | A | G | 0.101 | 0.107 | 0.024 | 5.51E-06 | 0.006 | 0.035 | 0.865 | 0.002 | | 38.340 |  |
|  |  | rs62376424 | C | T | 0.305 | -0.076 | 0.016 | 3.65E-06 | 0.008 | 0.023 | 0.723 | 0.002 | | 45.312 |  |
|  |  | rs6656807 | G | A | 0.611 | -0.070 | 0.015 | 5.50E-06 | -0.015 | 0.022 | 0.486 | 0.002 | | 43.178 |  |
|  |  | rs742016 | A | G | 0.334 | -0.069 | 0.015 | 4.66E-06 | 0.004 | 0.022 | 0.846 | 0.002 | | 38.786 |  |
| ebi-a-GCST90017089 | Gut microbiota abundance (genus Victivallis id.2256) | rs11899949 | G | A | 0.312 | 0.131 | 0.028 | 2.77E-06 | -0.012 | 0.023 | 0.600 | 0.007 | | 135.180 |  |
|  |  | rs12512543 | A | C | 0.085 | -0.178 | 0.037 | 2.54E-06 | -0.013 | 0.038 | 0.735 | 0.005 | | 91.233 |  |
|  |  | rs173120 | C | T | 0.810 | -0.134 | 0.029 | 7.65E-06 | 0.011 | 0.027 | 0.677 | 0.006 | | 101.830 |  |
|  |  | rs1882775 | A | G | 0.183 | -0.138 | 0.031 | 8.73E-06 | 0.013 | 0.028 | 0.643 | 0.006 | | 105.349 |  |
|  |  | rs2546432 | C | T | 0.544 | 0.111 | 0.025 | 9.93E-06 | -0.009 | 0.021 | 0.670 | 0.006 | | 112.399 |  |
|  |  | rs342302 | A | G | 0.138 | -0.153 | 0.035 | 8.16E-06 | -0.068 | 0.031 | 0.027 | 0.006 | | 102.307 |  |
|  |  | rs4764863 | G | A | 0.552 | 0.122 | 0.025 | 8.22E-07 | 0.036 | 0.021 | 0.090 | 0.007 | | 135.041 |  |
|  |  | rs4895919 | C | T | 0.519 | 0.117 | 0.025 | 2.75E-06 | -0.013 | 0.021 | 0.551 | 0.007 | | 126.018 |  |
|  |  | rs56349194 | A | G | 0.129 | -0.159 | 0.032 | 6.26E-07 | -0.002 | 0.031 | 0.944 | 0.006 | | 103.838 |  |
|  |  | rs911666 | T | C | 0.303 | -0.119 | 0.026 | 7.65E-06 | -0.044 | 0.023 | 0.054 | 0.006 | | 109.577 |  |
| ebi-a-GCST90016908 | Gut microbiota abundance (class Actinobacteria id.419) | rs11655079 | T | C | 0.186 | -0.056 | 0.012 | 5.93E-06 | 0.026 | 0.027 | 0.345 | 0.001 | | 17.487 |  |
|  |  | rs11745923 | G | T | 0.393 | 0.056 | 0.012 | 1.58E-06 | 0.016 | 0.022 | 0.458 | 0.002 | | 27.859 |  |
|  |  | rs12049045 | A | G | 0.383 | 0.051 | 0.011 | 8.63E-06 | -0.025 | 0.022 | 0.251 | 0.001 | | 22.613 |  |
|  |  | rs134366 | A | G | 0.932 | -0.112 | 0.024 | 1.50E-06 | 0.014 | 0.042 | 0.744 | 0.002 | | 29.298 |  |
|  |  | rs1376754 | G | A | 0.494 | 0.051 | 0.011 | 6.71E-06 | 0.038 | 0.021 | 0.073 | 0.001 | | 23.805 |  |
|  |  | rs1515761 | C | T | 0.926 | -0.076 | 0.017 | 4.96E-06 | 0.048 | 0.040 | 0.237 | 0.001 | | 14.707 |  |
|  |  | rs182549 | T | C | 0.598 | -0.111 | 0.012 | 3.79E-20 | -0.048 | 0.022 | 0.026 | 0.006 | | 110.295 |  |
|  |  | rs4945008 | G | A | 0.608 | -0.054 | 0.012 | 5.39E-06 | -0.022 | 0.022 | 0.314 | 0.001 | | 25.595 |  |
|  |  | rs6660520 | G | A | 0.729 | -0.071 | 0.013 | 1.11E-07 | 0.008 | 0.024 | 0.746 | 0.002 | | 36.769 |  |
|  |  | rs72767435 | T | C | 0.055 | -0.126 | 0.027 | 2.57E-06 | -0.041 | 0.047 | 0.382 | 0.002 | | 30.295 |  |
|  |  | rs7322849 | T | C | 0.092 | 0.094 | 0.019 | 6.21E-07 | 0.055 | 0.037 | 0.140 | 0.001 | | 27.413 |  |
|  |  | rs80083040 | T | G | 0.044 | 0.156 | 0.035 | 8.62E-06 | 0.021 | 0.051 | 0.677 | 0.002 | | 37.818 |  |
|  |  | rs8047955 | G | A | 0.669 | -0.058 | 0.012 | 8.34E-07 | 0.020 | 0.022 | 0.379 | 0.001 | | 26.984 |  |
|  |  | rs857444 | C | T | 0.369 | 0.051 | 0.012 | 8.92E-06 | -0.035 | 0.022 | 0.108 | 0.001 | | 22.189 |  |
|  |  | rs961091 | G | A | 0.371 | 0.050 | 0.011 | 8.68E-06 | 0.018 | 0.022 | 0.423 | 0.001 | | 21.539 |  |
| ebi-a-GCST90016909 | Gut microbiota abundance (class Alphaproteobacteria id.2379) | rs140912403 | C | T | 0.058 | -0.161 | 0.032 | 6.20E-07 | -0.050 | 0.045 | 0.265 | 0.003 | | 51.930 |  |
|  |  | rs34569731 | G | A | 0.576 | -0.071 | 0.016 | 7.38E-06 | 0.008 | 0.022 | 0.721 | 0.002 | | 44.652 |  |
|  |  | rs55876211 | C | T | 0.254 | -0.081 | 0.018 | 7.54E-06 | 0.015 | 0.024 | 0.531 | 0.002 | | 45.543 |  |
|  |  | rs62285697 | C | T | 0.254 | 0.081 | 0.018 | 9.76E-06 | -0.032 | 0.025 | 0.199 | 0.002 | | 45.115 |  |
|  |  | rs76784716 | A | G | 0.114 | 0.133 | 0.027 | 5.09E-07 | 0.010 | 0.034 | 0.773 | 0.004 | | 66.032 |  |
|  |  | rs7960664 | A | G | 0.915 | -0.097 | 0.022 | 8.84E-06 | 0.041 | 0.038 | 0.274 | 0.001 | | 26.965 |  |
|  |  | rs9813022 | A | G | 0.376 | -0.075 | 0.015 | 1.05E-06 | -0.003 | 0.022 | 0.909 | 0.003 | | 48.672 |  |
| ebi-a-GCST90016910 | Gut microbiota abundance (class Bacilli id.1673) | rs11110282 | A | G | 0.048 | -0.101 | 0.022 | 4.85E-06 | 0.004 | 0.049 | 0.931 | 0.001 | | 17.214 |  |
|  |  | rs11730038 | G | A | 0.294 | -0.063 | 0.013 | 1.96E-06 | -0.010 | 0.023 | 0.660 | 0.002 | | 30.306 |  |
|  |  | rs12797734 | T | C | 0.262 | 0.057 | 0.013 | 7.21E-06 | 0.026 | 0.025 | 0.295 | 0.001 | | 23.245 |  |
|  |  | rs13068444 | A | G | 0.177 | 0.060 | 0.014 | 9.53E-06 | -0.002 | 0.028 | 0.942 | 0.001 | | 19.329 |  |
|  |  | rs1595463 | C | A | 0.539 | 0.048 | 0.011 | 7.97E-06 | -0.006 | 0.021 | 0.780 | 0.001 | | 20.746 |  |
|  |  | rs28564647 | T | G | 0.167 | -0.061 | 0.014 | 7.81E-06 | -0.013 | 0.028 | 0.642 | 0.001 | | 19.222 |  |
|  |  | rs2952251 | G | A | 0.771 | 0.060 | 0.012 | 1.08E-06 | 0.016 | 0.027 | 0.548 | 0.001 | | 23.232 |  |
|  |  | rs34989881 | A | G | 0.049 | 0.111 | 0.025 | 6.55E-06 | -0.061 | 0.050 | 0.216 | 0.001 | | 20.931 |  |
|  |  | rs35344081 | G | A | 0.263 | 0.062 | 0.013 | 1.01E-06 | 0.008 | 0.024 | 0.725 | 0.001 | | 27.292 |  |
|  |  | rs4028634 | C | T | 0.635 | -0.052 | 0.011 | 2.21E-06 | -0.001 | 0.022 | 0.956 | 0.001 | | 23.072 |  |
|  |  | rs4459992 | T | C | 0.327 | 0.054 | 0.012 | 4.30E-06 | 0.033 | 0.023 | 0.143 | 0.001 | | 23.220 |  |
|  |  | rs57872228 | C | T | 0.117 | -0.071 | 0.015 | 9.22E-07 | -0.030 | 0.033 | 0.362 | 0.001 | | 19.321 |  |
|  |  | rs694949 | A | G | 0.095 | -0.081 | 0.018 | 7.60E-06 | -0.058 | 0.036 | 0.109 | 0.001 | | 20.851 |  |
|  |  | rs74663707 | C | T | 0.066 | 0.098 | 0.022 | 8.46E-06 | 0.019 | 0.043 | 0.655 | 0.001 | | 21.729 |  |
|  |  | rs7666190 | A | C | 0.884 | 0.104 | 0.025 | 8.47E-06 | -0.032 | 0.033 | 0.329 | 0.002 | | 40.742 |  |
|  |  | rs77558518 | A | G | 0.096 | -0.107 | 0.022 | 1.34E-06 | -0.015 | 0.036 | 0.674 | 0.002 | | 36.599 |  |
|  |  | rs78938557 | T | C | 0.030 | 0.108 | 0.023 | 1.07E-06 | 0.031 | 0.063 | 0.625 | 0.001 | | 12.483 |  |
|  |  | rs9581006 | T | C | 0.963 | -0.225 | 0.047 | 1.79E-06 | 0.084 | 0.056 | 0.134 | 0.004 | | 66.058 |  |
| ebi-a-GCST90016911 | Gut microbiota abundance (class Bacteroidia id.912) | rs11146701 | A | G | 0.343 | 0.047 | 0.011 | 7.08E-06 | -0.034 | 0.028 | 0.232 | 0.001 | | 18.567 |  |
|  |  | rs17343978 | A | C | 0.204 | -0.055 | 0.012 | 8.36E-06 | 0.018 | 0.026 | 0.482 | 0.001 | | 18.189 |  |
|  |  | rs2032750 | C | T | 0.539 | 0.051 | 0.011 | 1.92E-06 | 0.010 | 0.021 | 0.634 | 0.001 | | 23.587 |  |
|  |  | rs2363574 | T | C | 0.962 | 0.223 | 0.051 | 9.93E-06 | -0.065 | 0.056 | 0.248 | 0.004 | | 66.930 |  |
|  |  | rs4146051 | G | A | 0.917 | 0.107 | 0.025 | 8.76E-06 | 0.000 | 0.039 | 0.995 | 0.002 | | 32.206 |  |
|  |  | rs4916508 | A | G | 0.576 | 0.047 | 0.011 | 8.47E-06 | -0.003 | 0.021 | 0.903 | 0.001 | | 19.528 |  |
|  |  | rs55773148 | G | A | 0.053 | -0.122 | 0.024 | 3.90E-07 | -0.012 | 0.047 | 0.792 | 0.001 | | 27.240 |  |
|  |  | rs62531359 | T | G | 0.184 | 0.066 | 0.015 | 9.09E-06 | 0.013 | 0.028 | 0.631 | 0.001 | | 23.708 |  |
|  |  | rs62575403 | C | T | 0.042 | 0.140 | 0.031 | 7.06E-06 | 0.030 | 0.053 | 0.569 | 0.002 | | 29.171 |  |
|  |  | rs72706335 | T | C | 0.026 | -0.222 | 0.049 | 7.66E-06 | -0.055 | 0.067 | 0.415 | 0.002 | | 45.211 |  |
|  |  | rs73975615 | G | A | 0.008 | -0.207 | 0.044 | 1.22E-06 | -0.166 | 0.124 | 0.180 | 0.001 | | 11.723 |  |
|  |  | rs7631304 | G | A | 0.149 | -0.065 | 0.013 | 8.37E-07 | -0.019 | 0.030 | 0.523 | 0.001 | | 19.371 |  |
|  |  | rs79585701 | A | C | 0.142 | 0.065 | 0.015 | 9.99E-06 | 0.020 | 0.030 | 0.521 | 0.001 | | 18.670 |  |
|  |  | rs929878 | T | C | 0.787 | 0.055 | 0.012 | 4.73E-06 | -0.008 | 0.026 | 0.769 | 0.001 | | 18.530 |  |
| ebi-a-GCST90016912 | Gut microbiota abundance (class Betaproteobacteria id.2867) | rs11128180 | A | G | 0.233 | 0.059 | 0.013 | 3.67E-06 | -0.001 | 0.025 | 0.972 | 0.001 | | 23.052 |  |
|  |  | rs1511453 | A | G | 0.058 | 0.092 | 0.020 | 4.76E-06 | 0.038 | 0.045 | 0.406 | 0.001 | | 17.150 |  |
|  |  | rs1928341 | G | A | 0.595 | -0.053 | 0.011 | 2.02E-06 | -0.022 | 0.022 | 0.318 | 0.001 | | 24.442 |  |
|  |  | rs2321387 | G | A | 0.431 | -0.049 | 0.011 | 5.80E-06 | 0.016 | 0.021 | 0.452 | 0.001 | | 22.023 |  |
|  |  | rs2613606 | T | C | 0.582 | 0.051 | 0.011 | 2.20E-06 | 0.012 | 0.021 | 0.590 | 0.001 | | 23.529 |  |
|  |  | rs320161 | G | A | 0.770 | -0.057 | 0.013 | 7.33E-06 | 0.000 | 0.025 | 0.998 | 0.001 | | 21.304 |  |
|  |  | rs4033856 | T | C | 0.914 | -0.083 | 0.017 | 5.17E-07 | 0.005 | 0.038 | 0.896 | 0.001 | | 20.061 |  |
|  |  | rs56386628 | C | T | 0.258 | -0.062 | 0.014 | 5.87E-06 | -0.020 | 0.024 | 0.412 | 0.001 | | 27.198 |  |
|  |  | rs6087811 | T | G | 0.102 | -0.098 | 0.020 | 7.44E-07 | 0.049 | 0.035 | 0.165 | 0.002 | | 32.060 |  |
|  |  | rs62395635 | T | C | 0.062 | 0.110 | 0.024 | 2.94E-06 | 0.044 | 0.044 | 0.317 | 0.001 | | 25.832 |  |
|  |  | rs75242906 | C | T | 0.084 | -0.121 | 0.028 | 9.27E-06 | -0.092 | 0.038 | 0.017 | 0.002 | | 41.350 |  |
| ebi-a-GCST90016913 | Gut microbiota abundance (class Clostridia id.1859) | rs10774377 | G | A | 0.564 | -0.053 | 0.011 | 3.24E-06 | 0.005 | 0.021 | 0.806 | 0.001 | | 25.042 |  |
|  |  | rs112334273 | G | A | 0.278 | 0.064 | 0.013 | 3.81E-07 | -0.030 | 0.024 | 0.203 | 0.002 | | 30.298 |  |
|  |  | rs13105690 | C | T | 0.715 | 0.053 | 0.012 | 8.78E-06 | 0.010 | 0.024 | 0.683 | 0.001 | | 20.964 |  |
|  |  | rs13179700 | C | T | 0.656 | -0.051 | 0.011 | 3.37E-06 | 0.007 | 0.022 | 0.767 | 0.001 | | 21.711 |  |
|  |  | rs1842454 | G | A | 0.188 | -0.055 | 0.013 | 8.72E-06 | 0.021 | 0.027 | 0.440 | 0.001 | | 16.752 |  |
|  |  | rs2273429 | A | G | 0.105 | -0.072 | 0.015 | 4.52E-06 | 0.053 | 0.035 | 0.128 | 0.001 | | 18.010 |  |
|  |  | rs6797343 | T | G | 0.809 | -0.059 | 0.013 | 9.36E-06 | 0.034 | 0.027 | 0.210 | 0.001 | | 19.910 |  |
|  |  | rs6814436 | C | T | 0.859 | -0.074 | 0.015 | 9.65E-07 | 0.004 | 0.030 | 0.907 | 0.001 | | 24.328 |  |
|  |  | rs6815608 | C | T | 0.849 | -0.104 | 0.021 | 4.02E-07 | 0.006 | 0.030 | 0.847 | 0.003 | | 50.941 |  |
|  |  | rs72738886 | T | C | 0.076 | 0.087 | 0.019 | 8.24E-06 | 0.006 | 0.040 | 0.882 | 0.001 | | 19.374 |  |
|  |  | rs72915163 | T | C | 0.219 | -0.058 | 0.012 | 1.34E-06 | 0.060 | 0.026 | 0.020 | 0.001 | | 21.195 |  |
|  |  | rs992074 | T | C | 0.977 | -0.256 | 0.051 | 8.78E-07 | -0.063 | 0.071 | 0.371 | 0.003 | | 54.018 |  |
| ebi-a-GCST90016914 | Gut microbiota abundance (class Coriobacteriia id.809) | rs11250875 | T | C | 0.214 | 0.061 | 0.013 | 4.83E-06 | 0.021 | 0.026 | 0.422 | 0.001 | | 22.780 |  |
|  |  | rs11656361 | A | C | 0.182 | 0.077 | 0.018 | 8.02E-06 | 0.005 | 0.028 | 0.857 | 0.002 | | 32.685 |  |
|  |  | rs12974142 | G | A | 0.070 | 0.079 | 0.018 | 8.51E-06 | 0.024 | 0.041 | 0.570 | 0.001 | | 14.974 |  |
|  |  | rs13307134 | T | C | 0.833 | -0.057 | 0.013 | 7.80E-06 | -0.049 | 0.028 | 0.083 | 0.001 | | 16.310 |  |
|  |  | rs1397793 | A | G | 0.690 | 0.050 | 0.011 | 9.77E-06 | -0.035 | 0.023 | 0.134 | 0.001 | | 19.533 |  |
|  |  | rs1816223 | G | A | 0.799 | 0.059 | 0.013 | 4.84E-06 | -0.071 | 0.026 | 0.007 | 0.001 | | 20.244 |  |
|  |  | rs240104 | T | C | 0.285 | -0.060 | 0.013 | 1.52E-06 | -0.007 | 0.024 | 0.782 | 0.001 | | 27.237 |  |
|  |  | rs2442778 | A | G | 0.948 | 0.116 | 0.026 | 9.03E-06 | -0.104 | 0.048 | 0.029 | 0.001 | | 24.398 |  |
|  |  | rs3025411 | A | G | 0.107 | 0.093 | 0.021 | 8.27E-06 | -0.014 | 0.035 | 0.697 | 0.002 | | 30.035 |  |
|  |  | rs34739816 | G | T | 0.058 | 0.097 | 0.021 | 3.88E-06 | 0.075 | 0.045 | 0.096 | 0.001 | | 18.737 |  |
|  |  | rs67561917 | A | G | 0.183 | -0.071 | 0.015 | 5.39E-06 | -0.020 | 0.028 | 0.471 | 0.002 | | 27.966 |  |
|  |  | rs719099 | A | G | 0.099 | 0.078 | 0.016 | 5.43E-07 | 0.051 | 0.036 | 0.148 | 0.001 | | 19.822 |  |
|  |  | rs8010111 | A | G | 0.919 | 0.103 | 0.023 | 6.90E-06 | 0.010 | 0.038 | 0.795 | 0.002 | | 29.124 |  |
| ebi-a-GCST90016915 | Gut microbiota abundance (class Deltaproteobacteria id.3087) | rs1035691 | G | A | 0.596 | 0.055 | 0.012 | 9.65E-06 | -0.014 | 0.022 | 0.507 | 0.001 | | 26.927 |  |
|  |  | rs112381107 | C | T | 0.060 | 0.207 | 0.046 | 4.63E-06 | -0.061 | 0.045 | 0.170 | 0.005 | | 88.672 |  |
|  |  | rs11599763 | C | T | 0.592 | 0.054 | 0.012 | 3.94E-06 | 0.007 | 0.022 | 0.733 | 0.001 | | 26.266 |  |
|  |  | rs17084793 | G | A | 0.146 | -0.071 | 0.016 | 5.69E-06 | 0.036 | 0.030 | 0.232 | 0.001 | | 23.170 |  |
|  |  | rs17791387 | A | G | 0.095 | -0.074 | 0.015 | 1.60E-06 | 0.056 | 0.036 | 0.123 | 0.001 | | 17.148 |  |
|  |  | rs2692012 | G | A | 0.945 | -0.110 | 0.025 | 3.14E-06 | 0.069 | 0.047 | 0.138 | 0.001 | | 23.315 |  |
|  |  | rs2838334 | G | A | 0.344 | 0.056 | 0.012 | 5.45E-06 | 0.000 | 0.022 | 0.999 | 0.001 | | 26.148 |  |
|  |  | rs3935584 | C | T | 0.532 | -0.052 | 0.012 | 7.50E-06 | -0.004 | 0.021 | 0.857 | 0.001 | | 25.059 |  |
|  |  | rs4506934 | C | T | 0.118 | -0.094 | 0.020 | 3.59E-06 | -0.034 | 0.033 | 0.304 | 0.002 | | 33.589 |  |
|  |  | rs55744759 | A | G | 0.112 | -0.078 | 0.017 | 7.31E-06 | -0.013 | 0.033 | 0.689 | 0.001 | | 22.254 |  |
|  |  | rs6058181 | C | T | 0.162 | 0.083 | 0.017 | 3.40E-07 | -0.032 | 0.029 | 0.273 | 0.002 | | 33.993 |  |
|  |  | rs62020470 | A | G | 0.180 | -0.059 | 0.013 | 4.85E-06 | -0.017 | 0.028 | 0.538 | 0.001 | | 18.538 |  |
|  |  | rs9928243 | C | A | 0.471 | -0.054 | 0.012 | 5.02E-06 | 0.044 | 0.021 | 0.037 | 0.001 | | 26.534 |  |
| ebi-a-GCST90016916 | Gut microbiota abundance (class Erysipelotrichia id.2147) | rs1074800 | G | A | 0.581 | -0.049 | 0.011 | 6.15E-06 | 0.009 | 0.022 | 0.673 | 0.001 | | 21.674 |  |
|  |  | rs10781552 | C | T | 0.281 | -0.055 | 0.012 | 2.33E-06 | 0.029 | 0.024 | 0.220 | 0.001 | | 22.609 |  |
|  |  | rs17530232 | A | G | 0.053 | 0.103 | 0.022 | 2.79E-06 | 0.007 | 0.047 | 0.880 | 0.001 | | 19.513 |  |
|  |  | rs1884466 | C | T | 0.520 | -0.048 | 0.011 | 9.53E-06 | -0.009 | 0.021 | 0.684 | 0.001 | | 20.710 |  |
|  |  | rs2300774 | A | G | 0.561 | -0.052 | 0.011 | 8.95E-07 | 0.015 | 0.021 | 0.495 | 0.001 | | 24.848 |  |
|  |  | rs290833 | T | G | 0.493 | -0.050 | 0.011 | 8.03E-06 | 0.029 | 0.021 | 0.178 | 0.001 | | 22.706 |  |
|  |  | rs35161940 | T | C | 0.107 | -0.081 | 0.017 | 1.85E-06 | 0.031 | 0.034 | 0.361 | 0.001 | | 22.815 |  |
|  |  | rs4078432 | T | C | 0.827 | 0.061 | 0.013 | 4.23E-06 | -0.030 | 0.028 | 0.293 | 0.001 | | 19.477 |  |
|  |  | rs56970041 | T | G | 0.061 | 0.072 | 0.016 | 5.40E-06 | 0.017 | 0.044 | 0.700 | 0.001 | | 11.067 |  |
|  |  | rs62504403 | C | T | 0.195 | 0.068 | 0.013 | 1.12E-07 | -0.002 | 0.027 | 0.933 | 0.001 | | 26.782 |  |
|  |  | rs7234058 | T | C | 0.090 | -0.095 | 0.019 | 9.12E-07 | -0.019 | 0.037 | 0.607 | 0.001 | | 27.034 |  |
|  |  | rs7826267 | G | T | 0.931 | 0.084 | 0.020 | 9.28E-06 | 0.035 | 0.043 | 0.416 | 0.001 | | 16.669 |  |
|  |  | rs8003149 | C | T | 0.333 | 0.054 | 0.012 | 4.08E-06 | 0.012 | 0.022 | 0.591 | 0.001 | | 23.661 |  |
| ebi-a-GCST90016917 | Gut microbiota abundance (class Gammaproteobacteria id.3303) | rs11181912 | G | A | 0.323 | -0.058 | 0.012 | 9.95E-07 | 0.038 | 0.023 | 0.091 | 0.001 | | 26.944 |  |
|  |  | rs12404135 | A | G | 0.077 | -0.079 | 0.017 | 8.89E-06 | -0.027 | 0.039 | 0.496 | 0.001 | | 16.240 |  |
|  |  | rs6706173 | A | C | 0.137 | 0.074 | 0.015 | 1.99E-07 | 0.055 | 0.031 | 0.074 | 0.001 | | 23.967 |  |
|  |  | rs75101789 | C | T | 0.087 | 0.073 | 0.016 | 8.79E-06 | 0.093 | 0.037 | 0.013 | 0.001 | | 15.515 |  |
|  |  | rs79795896 | A | G | 0.047 | -0.159 | 0.035 | 7.92E-06 | 0.054 | 0.050 | 0.282 | 0.002 | | 41.498 |  |
|  |  | rs9494710 | C | T | 0.322 | -0.055 | 0.012 | 4.55E-06 | -0.011 | 0.023 | 0.637 | 0.001 | | 24.236 |  |
| ebi-a-GCST90016918 | Gut microbiota abundance (class Lentisphaeria id.2250) | rs1002941 | A | G | 0.755 | -0.105 | 0.023 | 8.15E-06 | 0.000 | 0.025 | 0.997 | 0.004 | | 75.241 |  |
|  |  | rs11770843 | C | T | 0.328 | 0.109 | 0.023 | 1.91E-06 | -0.034 | 0.023 | 0.129 | 0.005 | | 97.382 |  |
|  |  | rs17114848 | G | A | 0.099 | 0.152 | 0.032 | 4.06E-06 | 0.074 | 0.036 | 0.038 | 0.004 | | 76.261 |  |
|  |  | rs2031282 | A | G | 0.184 | 0.122 | 0.027 | 4.38E-06 | -0.008 | 0.028 | 0.764 | 0.004 | | 82.829 |  |
|  |  | rs2825714 | A | G | 0.168 | -0.137 | 0.029 | 1.72E-06 | 0.012 | 0.028 | 0.677 | 0.005 | | 97.494 |  |
|  |  | rs62570196 | C | T | 0.042 | -0.216 | 0.044 | 1.08E-06 | 0.023 | 0.053 | 0.658 | 0.004 | | 69.287 |  |
|  |  | rs72640280 | A | G | 0.055 | 0.220 | 0.049 | 5.18E-06 | 0.006 | 0.046 | 0.889 | 0.005 | | 93.335 |  |
|  |  | rs77599476 | A | G | 0.056 | 0.230 | 0.048 | 1.86E-06 | -0.087 | 0.046 | 0.059 | 0.006 | | 103.300 |  |
| ebi-a-GCST90016919 | Gut microbiota abundance (class Melainabacteria id.1589) | rs10148250 | A | G | 0.583 | -0.086 | 0.019 | 8.67E-06 | 0.012 | 0.022 | 0.582 | 0.004 | | 66.486 |  |
|  |  | rs10738747 | A | G | 0.565 | -0.081 | 0.018 | 9.96E-06 | -0.002 | 0.021 | 0.920 | 0.003 | | 60.014 |  |
|  |  | rs11150282 | T | C | 0.360 | 0.099 | 0.020 | 6.03E-07 | -0.007 | 0.022 | 0.750 | 0.005 | | 83.139 |  |
|  |  | rs113884518 | T | C | 0.024 | -0.205 | 0.045 | 8.06E-06 | 0.019 | 0.068 | 0.784 | 0.002 | | 36.521 |  |
|  |  | rs28678345 | T | C | 0.049 | 0.215 | 0.047 | 6.69E-06 | -0.015 | 0.049 | 0.760 | 0.004 | | 79.246 |  |
|  |  | rs367480 | A | G | 0.642 | 0.084 | 0.019 | 8.20E-06 | -0.037 | 0.022 | 0.090 | 0.003 | | 59.364 |  |
|  |  | rs4129395 | G | A | 0.485 | 0.090 | 0.019 | 1.48E-06 | -0.033 | 0.021 | 0.121 | 0.004 | | 73.869 |  |
|  |  | rs789069 | A | C | 0.141 | -0.104 | 0.023 | 6.85E-06 | 0.002 | 0.030 | 0.959 | 0.003 | | 47.774 |  |
|  |  | rs79790072 | T | C | 0.032 | 0.227 | 0.049 | 3.29E-06 | 0.065 | 0.061 | 0.283 | 0.003 | | 57.731 |  |
|  |  | rs9864379 | T | C | 0.150 | -0.160 | 0.029 | 5.36E-08 | -0.008 | 0.030 | 0.801 | 0.006 | | 119.896 |  |
| ebi-a-GCST90016920 | Gut microbiota abundance (class Methanobacteria id.119) | rs10202904 | G | T | 0.592 | 0.122 | 0.024 | 3.01E-07 | -0.024 | 0.022 | 0.263 | 0.007 | | 132.297 |  |
|  |  | rs10424197 | A | G | 0.743 | 0.111 | 0.025 | 9.28E-06 | -0.016 | 0.024 | 0.501 | 0.005 | | 87.104 |  |
|  |  | rs4257531 | G | A | 0.106 | 0.164 | 0.036 | 7.44E-06 | -0.024 | 0.035 | 0.487 | 0.005 | | 94.137 |  |
|  |  | rs56131665 | G | A | 0.107 | 0.179 | 0.039 | 6.18E-06 | 0.020 | 0.034 | 0.564 | 0.006 | | 112.919 |  |
|  |  | rs6508769 | C | T | 0.853 | -0.154 | 0.034 | 8.23E-06 | 0.011 | 0.030 | 0.704 | 0.006 | | 108.783 |  |
|  |  | rs6776814 | T | C | 0.020 | -0.200 | 0.041 | 1.63E-06 | 0.017 | 0.074 | 0.819 | 0.002 | | 29.335 |  |
|  |  | rs73068003 | G | T | 0.096 | -0.158 | 0.035 | 8.45E-06 | 0.052 | 0.036 | 0.144 | 0.004 | | 80.073 |  |
|  |  | rs73457410 | A | G | 0.064 | 0.215 | 0.044 | 1.41E-06 | 0.010 | 0.043 | 0.809 | 0.006 | | 101.879 |  |
|  |  | rs75208022 | C | T | 0.093 | -0.227 | 0.049 | 5.92E-06 | -0.018 | 0.036 | 0.615 | 0.009 | | 161.898 |  |
|  |  | rs894996 | C | A | 0.070 | 0.217 | 0.045 | 1.88E-06 | 0.028 | 0.041 | 0.492 | 0.006 | | 113.062 |  |
| ebi-a-GCST90016921 | Gut microbiota abundance (class Mollicutes id.3920) | rs10108398 | G | A | 0.276 | 0.077 | 0.015 | 1.09E-06 | -0.002 | 0.024 | 0.947 | 0.002 | | 43.409 |  |
|  |  | rs11890098 | A | G | 0.278 | 0.074 | 0.015 | 9.57E-07 | -0.003 | 0.024 | 0.908 | 0.002 | | 40.890 |  |
|  |  | rs12566890 | T | G | 0.132 | -0.101 | 0.023 | 3.65E-06 | -0.007 | 0.032 | 0.825 | 0.002 | | 43.037 |  |
|  |  | rs17214486 | C | A | 0.323 | 0.061 | 0.014 | 6.61E-06 | -0.033 | 0.023 | 0.145 | 0.002 | | 29.901 |  |
|  |  | rs2464826 | A | C | 0.113 | 0.094 | 0.021 | 8.39E-06 | -0.004 | 0.034 | 0.913 | 0.002 | | 32.834 |  |
|  |  | rs28537087 | G | A | 0.249 | 0.082 | 0.019 | 8.07E-06 | -0.009 | 0.025 | 0.721 | 0.003 | | 46.318 |  |
|  |  | rs3768491 | G | A | 0.712 | 0.068 | 0.015 | 4.23E-06 | 0.023 | 0.023 | 0.321 | 0.002 | | 34.971 |  |
|  |  | rs4885016 | C | T | 0.865 | 0.082 | 0.018 | 7.27E-06 | 0.000 | 0.031 | 0.995 | 0.002 | | 28.779 |  |
|  |  | rs6043847 | T | C | 0.059 | -0.115 | 0.025 | 4.55E-06 | 0.073 | 0.045 | 0.106 | 0.001 | | 26.883 |  |
|  |  | rs72901605 | T | C | 0.115 | -0.084 | 0.018 | 3.26E-06 | 0.021 | 0.033 | 0.525 | 0.001 | | 26.452 |  |
|  |  | rs74603314 | T | C | 0.040 | 0.222 | 0.046 | 1.56E-06 | -0.007 | 0.054 | 0.898 | 0.004 | | 69.646 |  |
|  |  | rs78169027 | A | G | 0.061 | -0.108 | 0.024 | 5.88E-06 | 0.032 | 0.045 | 0.476 | 0.001 | | 24.835 |  |
| ebi-a-GCST90016922 | Gut microbiota abundance (class Negativicutes id.2164) | rs1135612 | G | A | 0.211 | 0.053 | 0.012 | 9.26E-06 | 0.000 | 0.026 | 0.995 | 0.001 | | 17.153 |  |
|  |  | rs13086907 | G | A | 0.222 | 0.063 | 0.013 | 1.95E-06 | -0.027 | 0.026 | 0.298 | 0.001 | | 24.826 |  |
|  |  | rs1643968 | T | C | 0.358 | -0.057 | 0.011 | 4.15E-07 | -0.020 | 0.022 | 0.372 | 0.001 | | 26.976 |  |
|  |  | rs1649999 | A | G | 0.097 | 0.075 | 0.017 | 7.58E-06 | 0.037 | 0.036 | 0.305 | 0.001 | | 18.018 |  |
|  |  | rs2834062 | A | G | 0.313 | 0.049 | 0.011 | 8.44E-06 | -0.030 | 0.023 | 0.194 | 0.001 | | 18.879 |  |
|  |  | rs4463806 | C | T | 0.808 | 0.054 | 0.013 | 7.81E-06 | 0.026 | 0.027 | 0.342 | 0.001 | | 16.851 |  |
|  |  | rs4722181 | T | G | 0.471 | 0.050 | 0.011 | 2.00E-06 | -0.003 | 0.021 | 0.897 | 0.001 | | 22.996 |  |
|  |  | rs60274479 | T | C | 0.198 | -0.066 | 0.013 | 1.16E-06 | -0.023 | 0.027 | 0.395 | 0.001 | | 25.395 |  |
|  |  | rs61249479 | A | C | 0.155 | 0.078 | 0.017 | 2.95E-06 | -0.036 | 0.029 | 0.220 | 0.002 | | 29.068 |  |
|  |  | rs71405394 | G | A | 0.068 | -0.114 | 0.024 | 2.17E-06 | 0.047 | 0.042 | 0.268 | 0.002 | | 30.510 |  |
|  |  | rs73232831 | G | A | 0.035 | -0.152 | 0.031 | 1.87E-06 | 0.021 | 0.057 | 0.720 | 0.002 | | 28.466 |  |
|  |  | rs9423647 | G | A | 0.558 | 0.048 | 0.011 | 6.06E-06 | 0.024 | 0.021 | 0.262 | 0.001 | | 20.705 |  |
| ebi-a-GCST90016923 | Gut microbiota abundance (class Verrucomicrobiae id.4029) | rs111862613 | T | C | 0.178 | 0.091 | 0.020 | 3.74E-06 | 0.013 | 0.029 | 0.662 | 0.002 | | 44.212 |  |
|  |  | rs117107102 | A | G | 0.048 | 0.205 | 0.043 | 2.92E-06 | 0.026 | 0.049 | 0.603 | 0.004 | | 69.938 |  |
|  |  | rs11729256 | T | C | 0.170 | 0.075 | 0.015 | 6.73E-07 | 0.004 | 0.028 | 0.875 | 0.002 | | 29.099 |  |
|  |  | rs12908520 | G | A | 0.426 | 0.062 | 0.013 | 2.17E-06 | 0.017 | 0.021 | 0.429 | 0.002 | | 34.425 |  |
|  |  | rs2602429 | T | C | 0.739 | -0.075 | 0.016 | 2.58E-06 | -0.015 | 0.024 | 0.532 | 0.002 | | 39.582 |  |
|  |  | rs4242783 | A | G | 0.717 | -0.069 | 0.015 | 2.64E-06 | 0.017 | 0.024 | 0.471 | 0.002 | | 35.457 |  |
|  |  | rs4936098 | G | A | 0.651 | -0.065 | 0.014 | 1.12E-06 | 0.005 | 0.022 | 0.832 | 0.002 | | 35.143 |  |
|  |  | rs61779207 | G | A | 0.224 | -0.076 | 0.017 | 6.72E-06 | -0.010 | 0.026 | 0.707 | 0.002 | | 36.674 |  |
|  |  | rs74542928 | T | C | 0.048 | 0.112 | 0.024 | 1.63E-06 | -0.023 | 0.049 | 0.640 | 0.001 | | 21.084 |  |
|  |  | rs9349825 | A | G | 0.190 | -0.070 | 0.015 | 2.54E-06 | 0.044 | 0.027 | 0.103 | 0.002 | | 27.975 |  |
|  |  | rs941682 | G | A | 0.274 | -0.063 | 0.014 | 9.61E-06 | 0.028 | 0.024 | 0.237 | 0.002 | | 29.115 |  |
| ebi-a-GCST90016924 | Gut microbiota abundance (family Acidaminococcaceae id.2166) | rs262812 | T | C | 0.305 | -0.066 | 0.014 | 3.25E-06 | -0.014 | 0.023 | 0.556 | 0.002 | | 33.588 |  |
|  |  | rs2933324 | G | A | 0.773 | 0.066 | 0.014 | 2.24E-06 | 0.052 | 0.025 | 0.040 | 0.002 | | 28.312 |  |
|  |  | rs45497800 | T | C | 0.143 | -0.118 | 0.026 | 5.86E-06 | 0.030 | 0.030 | 0.318 | 0.003 | | 62.652 |  |
|  |  | rs6589457 | G | A | 0.950 | -0.166 | 0.035 | 2.32E-06 | 0.049 | 0.048 | 0.315 | 0.003 | | 48.171 |  |
|  |  | rs6923842 | T | C | 0.116 | -0.080 | 0.017 | 2.21E-06 | 0.067 | 0.033 | 0.044 | 0.001 | | 23.867 |  |
|  |  | rs74540770 | G | A | 0.081 | -0.109 | 0.024 | 7.09E-06 | 0.005 | 0.039 | 0.897 | 0.002 | | 32.300 |  |
|  |  | rs78702810 | T | C | 0.105 | -0.144 | 0.032 | 9.16E-06 | -0.011 | 0.035 | 0.761 | 0.004 | | 71.564 |  |
| ebi-a-GCST90016925 | Gut microbiota abundance (family Actinomycetaceae id.421) | rs2889192 | T | G | 0.851 | -0.089 | 0.020 | 3.64E-06 | 0.005 | 0.030 | 0.879 | 0.002 | | 36.706 |  |
|  |  | rs34583783 | G | T | 0.061 | 0.124 | 0.026 | 5.48E-06 | -0.032 | 0.044 | 0.471 | 0.002 | | 32.072 |  |
|  |  | rs35011108 | A | G | 0.068 | 0.242 | 0.050 | 1.83E-06 | -0.038 | 0.043 | 0.371 | 0.007 | | 136.474 |  |
|  |  | rs4073240 | G | A | 0.381 | 0.075 | 0.016 | 6.05E-06 | 0.011 | 0.022 | 0.617 | 0.003 | | 48.449 |  |
|  |  | rs58484246 | T | C | 0.366 | 0.076 | 0.017 | 6.12E-06 | 0.009 | 0.022 | 0.700 | 0.003 | | 49.454 |  |
| ebi-a-GCST90016926 | Gut microbiota abundance (family Alcaligenaceae id.2875) | rs112135816 | T | G | 0.061 | -0.078 | 0.017 | 5.28E-06 | -0.067 | 0.044 | 0.131 | 0.001 | | 12.607 |  |
|  |  | rs1153990 | A | G | 0.315 | -0.059 | 0.013 | 5.97E-06 | 0.013 | 0.023 | 0.558 | 0.001 | | 27.334 |  |
|  |  | rs147968 | C | T | 0.461 | 0.049 | 0.011 | 9.13E-06 | 0.038 | 0.021 | 0.076 | 0.001 | | 21.862 |  |
|  |  | rs28480294 | C | T | 0.643 | 0.052 | 0.012 | 6.61E-06 | -0.017 | 0.022 | 0.439 | 0.001 | | 22.533 |  |
|  |  | rs4033856 | T | C | 0.914 | -0.082 | 0.017 | 1.03E-06 | 0.005 | 0.038 | 0.896 | 0.001 | | 19.388 |  |
|  |  | rs62191117 | A | G | 0.205 | 0.068 | 0.013 | 2.76E-07 | 0.003 | 0.026 | 0.923 | 0.002 | | 28.033 |  |
|  |  | rs62395635 | T | C | 0.062 | 0.111 | 0.024 | 3.35E-06 | 0.044 | 0.044 | 0.317 | 0.001 | | 26.230 |  |
|  |  | rs6969323 | A | C | 0.220 | -0.059 | 0.013 | 3.89E-06 | -0.002 | 0.026 | 0.940 | 0.001 | | 22.164 |  |
|  |  | rs74776516 | T | G | 0.067 | -0.094 | 0.021 | 6.85E-06 | -0.065 | 0.042 | 0.126 | 0.001 | | 20.373 |  |
|  |  | rs7638039 | T | C | 0.247 | 0.060 | 0.013 | 2.70E-06 | -0.012 | 0.025 | 0.635 | 0.001 | | 24.864 |  |
|  |  | rs9537886 | A | C | 0.429 | -0.057 | 0.011 | 2.35E-07 | 0.019 | 0.021 | 0.383 | 0.002 | | 29.319 |  |
| ebi-a-GCST90016927 | Gut microbiota abundance (family Bacteroidaceae id.917) | rs11585893 | A | G | 0.247 | -0.074 | 0.015 | 1.80E-06 | -0.045 | 0.025 | 0.071 | 0.002 | | 37.506 |  |
|  |  | rs13207588 | A | G | 0.195 | -0.059 | 0.013 | 7.48E-06 | -0.023 | 0.027 | 0.402 | 0.001 | | 20.234 |  |
|  |  | rs1340391 | T | C | 0.135 | -0.059 | 0.013 | 6.73E-06 | -0.001 | 0.031 | 0.963 | 0.001 | | 15.022 |  |
|  |  | rs17619981 | T | G | 0.135 | 0.088 | 0.019 | 2.69E-06 | 0.018 | 0.031 | 0.561 | 0.002 | | 33.280 |  |
|  |  | rs2023437 | T | C | 0.124 | -0.078 | 0.017 | 5.02E-06 | 0.007 | 0.032 | 0.834 | 0.001 | | 24.432 |  |
|  |  | rs66474973 | G | T | 0.101 | 0.081 | 0.016 | 6.81E-07 | -0.004 | 0.035 | 0.907 | 0.001 | | 22.069 |  |
|  |  | rs66710942 | T | C | 0.475 | -0.049 | 0.011 | 5.86E-06 | -0.057 | 0.021 | 0.007 | 0.001 | | 21.810 |  |
|  |  | rs6795673 | C | T | 0.479 | 0.054 | 0.011 | 3.38E-07 | -0.004 | 0.021 | 0.846 | 0.001 | | 26.585 |  |
|  |  | rs9507307 | C | T | 0.245 | 0.060 | 0.013 | 2.13E-06 | -0.052 | 0.025 | 0.034 | 0.001 | | 24.814 |  |
| ebi-a-GCST90016928 | Gut microbiota abundance (family Bacteroidales S24 7group id.11173) | rs10872669 | G | A | 0.908 | 0.123 | 0.028 | 9.49E-06 | -0.006 | 0.037 | 0.862 | 0.003 | | 46.341 |  |
|  |  | rs12748533 | G | T | 0.289 | -0.082 | 0.017 | 2.59E-06 | -0.015 | 0.024 | 0.527 | 0.003 | | 50.909 |  |
|  |  | rs17043785 | T | C | 0.088 | -0.176 | 0.035 | 5.12E-07 | -0.045 | 0.037 | 0.226 | 0.005 | | 91.847 |  |
|  |  | rs1850003 | A | G | 0.232 | 0.084 | 0.018 | 2.41E-06 | -0.019 | 0.025 | 0.448 | 0.003 | | 46.265 |  |
|  |  | rs61508842 | T | C | 0.088 | 0.123 | 0.027 | 7.83E-06 | 0.022 | 0.037 | 0.562 | 0.002 | | 44.153 |  |
|  |  | rs738193 | T | C | 0.354 | 0.085 | 0.017 | 3.82E-07 | -0.019 | 0.022 | 0.380 | 0.003 | | 60.400 |  |
|  |  | rs78609301 | A | G | 0.301 | -0.087 | 0.020 | 7.09E-06 | -0.004 | 0.023 | 0.860 | 0.003 | | 58.175 |  |
|  |  | rs941000 | T | C | 0.636 | -0.085 | 0.016 | 3.16E-07 | 0.018 | 0.022 | 0.406 | 0.003 | | 61.589 |  |
| ebi-a-GCST90016929 | Gut microbiota abundance (family Bifidobacteriaceae id.433) | rs10831953 | G | A | 0.300 | 0.054 | 0.012 | 9.95E-06 | 0.053 | 0.023 | 0.020 | 0.001 | | 22.277 |  |
|  |  | rs12446429 | T | C | 0.195 | 0.081 | 0.019 | 8.53E-06 | -0.007 | 0.028 | 0.810 | 0.002 | | 37.875 |  |
|  |  | rs13020688 | G | A | 0.305 | 0.058 | 0.012 | 1.57E-06 | -0.001 | 0.023 | 0.962 | 0.001 | | 26.567 |  |
|  |  | rs182549 | T | C | 0.598 | -0.117 | 0.013 | 5.94E-20 | -0.048 | 0.022 | 0.026 | 0.007 | | 121.688 |  |
|  |  | rs4957061 | T | C | 0.423 | 0.057 | 0.012 | 1.15E-06 | -0.006 | 0.022 | 0.787 | 0.002 | | 29.109 |  |
|  |  | rs540489 | T | G | 0.176 | -0.063 | 0.014 | 5.37E-06 | -0.015 | 0.028 | 0.592 | 0.001 | | 21.305 |  |
|  |  | rs55888705 | A | G | 0.282 | 0.054 | 0.012 | 8.66E-06 | -0.020 | 0.023 | 0.390 | 0.001 | | 21.404 |  |
|  |  | rs6899771 | A | G | 0.101 | -0.091 | 0.020 | 7.28E-06 | 0.000 | 0.035 | 0.992 | 0.002 | | 27.783 |  |
|  |  | rs7174549 | T | C | 0.641 | -0.055 | 0.012 | 6.87E-06 | 0.011 | 0.022 | 0.619 | 0.001 | | 25.714 |  |
|  |  | rs7322849 | T | C | 0.092 | 0.111 | 0.020 | 1.74E-08 | 0.055 | 0.037 | 0.140 | 0.002 | | 37.664 |  |
|  |  | rs73797465 | T | G | 0.115 | -0.094 | 0.021 | 4.85E-06 | -0.022 | 0.034 | 0.516 | 0.002 | | 33.226 |  |
|  |  | rs857444 | C | T | 0.369 | 0.055 | 0.012 | 3.82E-06 | -0.035 | 0.022 | 0.108 | 0.001 | | 26.232 |  |
| ebi-a-GCST90016931 | Gut microbiota abundance (family Clostridiaceae1 id.1869) | rs10875374 | T | C | 0.529 | 0.054 | 0.012 | 8.10E-06 | -0.007 | 0.021 | 0.753 | 0.001 | | 26.365 |  |
|  |  | rs12186080 | G | A | 0.165 | 0.075 | 0.016 | 5.34E-06 | -0.031 | 0.029 | 0.274 | 0.002 | | 28.306 |  |
|  |  | rs12341505 | G | A | 0.094 | 0.081 | 0.018 | 4.54E-06 | -0.073 | 0.036 | 0.043 | 0.001 | | 20.648 |  |
|  |  | rs2795528 | G | A | 0.056 | -0.181 | 0.039 | 3.81E-06 | 0.010 | 0.047 | 0.825 | 0.003 | | 63.556 |  |
|  |  | rs2817172 | C | T | 0.396 | 0.056 | 0.012 | 5.27E-06 | -0.030 | 0.022 | 0.166 | 0.002 | | 27.887 |  |
|  |  | rs4723021 | T | C | 0.068 | -0.106 | 0.024 | 7.42E-06 | 0.054 | 0.042 | 0.203 | 0.001 | | 26.326 |  |
|  |  | rs550843 | T | C | 0.282 | -0.073 | 0.017 | 7.09E-06 | 0.026 | 0.024 | 0.269 | 0.002 | | 40.208 |  |
|  |  | rs56188186 | A | G | 0.048 | 0.097 | 0.022 | 8.24E-06 | -0.114 | 0.049 | 0.021 | 0.001 | | 15.779 |  |
|  |  | rs62397761 | A | G | 0.320 | 0.062 | 0.014 | 9.08E-06 | 0.005 | 0.023 | 0.842 | 0.002 | | 30.324 |  |
|  |  | rs881532 | G | A | 0.505 | 0.053 | 0.012 | 7.90E-06 | 0.000 | 0.021 | 0.988 | 0.001 | | 26.086 |  |
| ebi-a-GCST90016932 | Gut microbiota abundance (family Clostridiales vadin BB60 group id.11286) | rs10517600 | G | T | 0.381 | -0.063 | 0.014 | 6.83E-06 | 0.007 | 0.022 | 0.752 | 0.002 | | 34.074 |  |
|  |  | rs10904722 | C | T | 0.239 | -0.067 | 0.015 | 5.05E-06 | 0.022 | 0.025 | 0.364 | 0.002 | | 30.158 |  |
|  |  | rs118104867 | C | T | 0.069 | 0.214 | 0.046 | 3.44E-06 | 0.006 | 0.042 | 0.879 | 0.006 | | 109.598 |  |
|  |  | rs13409132 | A | G | 0.036 | -0.165 | 0.035 | 4.37E-06 | -0.059 | 0.056 | 0.294 | 0.002 | | 34.455 |  |
|  |  | rs17121075 | G | A | 0.214 | 0.077 | 0.017 | 7.91E-06 | -0.010 | 0.026 | 0.714 | 0.002 | | 36.628 |  |
|  |  | rs2191834 | T | G | 0.760 | -0.075 | 0.016 | 2.50E-06 | -0.011 | 0.025 | 0.670 | 0.002 | | 37.343 |  |
|  |  | rs28691777 | C | T | 0.043 | 0.137 | 0.027 | 6.96E-07 | -0.044 | 0.051 | 0.391 | 0.002 | | 28.692 |  |
|  |  | rs34088226 | A | G | 0.058 | -0.118 | 0.027 | 7.66E-06 | 0.027 | 0.045 | 0.556 | 0.002 | | 28.042 |  |
|  |  | rs55682560 | C | T | 0.078 | -0.132 | 0.026 | 4.97E-07 | 0.000 | 0.039 | 0.996 | 0.002 | | 45.522 |  |
|  |  | rs6588624 | A | G | 0.483 | 0.066 | 0.014 | 1.79E-06 | -0.023 | 0.021 | 0.271 | 0.002 | | 40.262 |  |
|  |  | rs66714985 | A | C | 0.101 | 0.117 | 0.025 | 4.85E-06 | 0.007 | 0.035 | 0.833 | 0.002 | | 45.789 |  |
|  |  | rs7226487 | A | G | 0.453 | -0.064 | 0.014 | 3.58E-06 | 0.004 | 0.021 | 0.835 | 0.002 | | 37.724 |  |
|  |  | rs7538034 | T | G | 0.164 | -0.079 | 0.017 | 2.37E-06 | 0.033 | 0.029 | 0.244 | 0.002 | | 31.055 |  |
|  |  | rs7725895 | A | G | 0.120 | -0.116 | 0.024 | 3.94E-06 | -0.029 | 0.033 | 0.382 | 0.003 | | 52.542 |  |
|  |  | rs989682 | A | G | 0.231 | 0.070 | 0.016 | 6.85E-06 | 0.019 | 0.025 | 0.461 | 0.002 | | 32.138 |  |
| ebi-a-GCST90016933 | Gut microbiota abundance (family Coriobacteriaceae id.811) | rs11250875 | T | C | 0.214 | 0.061 | 0.013 | 4.83E-06 | 0.021 | 0.026 | 0.422 | 0.001 | | 22.780 |  |
|  |  | rs11656361 | A | C | 0.182 | 0.077 | 0.018 | 8.02E-06 | 0.005 | 0.028 | 0.857 | 0.002 | | 32.685 |  |
|  |  | rs12974142 | G | A | 0.070 | 0.079 | 0.018 | 8.51E-06 | 0.024 | 0.041 | 0.570 | 0.001 | | 14.974 |  |
|  |  | rs13307134 | T | C | 0.833 | -0.057 | 0.013 | 7.80E-06 | -0.049 | 0.028 | 0.083 | 0.001 | | 16.310 |  |
|  |  | rs1397793 | A | G | 0.690 | 0.050 | 0.011 | 9.77E-06 | -0.035 | 0.023 | 0.134 | 0.001 | | 19.533 |  |
|  |  | rs1816223 | G | A | 0.799 | 0.059 | 0.013 | 4.84E-06 | -0.071 | 0.026 | 0.007 | 0.001 | | 20.244 |  |
|  |  | rs240104 | T | C | 0.285 | -0.060 | 0.013 | 1.52E-06 | -0.007 | 0.024 | 0.782 | 0.001 | | 27.237 |  |
|  |  | rs2442778 | A | G | 0.948 | 0.116 | 0.026 | 9.03E-06 | -0.104 | 0.048 | 0.029 | 0.001 | | 24.398 |  |
|  |  | rs3025411 | A | G | 0.107 | 0.093 | 0.021 | 8.27E-06 | -0.014 | 0.035 | 0.697 | 0.002 | | 30.035 |  |
|  |  | rs34739816 | G | T | 0.058 | 0.097 | 0.021 | 3.88E-06 | 0.075 | 0.045 | 0.096 | 0.001 | | 18.737 |  |
|  |  | rs67561917 | A | G | 0.183 | -0.071 | 0.015 | 5.39E-06 | -0.020 | 0.028 | 0.471 | 0.002 | | 27.966 |  |
|  |  | rs719099 | A | G | 0.099 | 0.078 | 0.016 | 5.43E-07 | 0.051 | 0.036 | 0.148 | 0.001 | | 19.822 |  |
|  |  | rs8010111 | A | G | 0.919 | 0.103 | 0.023 | 6.90E-06 | 0.010 | 0.038 | 0.795 | 0.002 | | 29.124 |  |
| ebi-a-GCST90016934 | Gut microbiota abundance (family Defluviitaleaceae id.1924) | rs112893842 | T | C | 0.090 | 0.111 | 0.023 | 2.75E-06 | -0.043 | 0.037 | 0.243 | 0.002 | | 36.832 |  |
|  |  | rs1582238 | C | T | 0.641 | -0.080 | 0.017 | 1.69E-06 | -0.020 | 0.022 | 0.366 | 0.003 | | 54.449 |  |
|  |  | rs17051335 | C | T | 0.102 | -0.134 | 0.029 | 4.58E-06 | -0.009 | 0.035 | 0.794 | 0.003 | | 60.911 |  |
|  |  | rs1908593 | T | C | 0.559 | 0.070 | 0.016 | 7.86E-06 | 0.033 | 0.022 | 0.121 | 0.002 | | 44.687 |  |
|  |  | rs4344384 | T | G | 0.504 | -0.071 | 0.016 | 5.86E-06 | 0.018 | 0.021 | 0.386 | 0.003 | | 46.178 |  |
|  |  | rs4677103 | A | G | 0.181 | 0.098 | 0.020 | 9.42E-07 | 0.027 | 0.028 | 0.329 | 0.003 | | 52.132 |  |
|  |  | rs540220 | C | T | 0.911 | 0.124 | 0.029 | 9.48E-06 | 0.056 | 0.037 | 0.128 | 0.002 | | 45.710 |  |
|  |  | rs55658617 | T | C | 0.035 | 0.177 | 0.036 | 1.41E-06 | -0.015 | 0.058 | 0.798 | 0.002 | | 39.115 |  |
|  |  | rs72731813 | C | T | 0.049 | -0.150 | 0.029 | 2.76E-07 | 0.023 | 0.049 | 0.640 | 0.002 | | 38.161 |  |
|  |  | rs9608282 | T | G | 0.034 | 0.139 | 0.030 | 4.61E-06 | 0.038 | 0.059 | 0.523 | 0.001 | | 23.116 |  |
|  |  | rs9725395 | A | G | 0.115 | -0.138 | 0.030 | 3.41E-06 | 0.007 | 0.033 | 0.839 | 0.004 | | 71.624 |  |
| ebi-a-GCST90016935 | Gut microbiota abundance (family Desulfovibrionaceae id.3169) | rs112381107 | C | T | 0.060 | 0.211 | 0.046 | 2.82E-06 | -0.061 | 0.045 | 0.170 | 0.005 | | 92.898 |  |
|  |  | rs11599763 | C | T | 0.592 | 0.056 | 0.012 | 2.50E-06 | 0.007 | 0.022 | 0.733 | 0.001 | | 27.411 |  |
|  |  | rs17791387 | A | G | 0.095 | -0.073 | 0.015 | 2.10E-06 | 0.056 | 0.036 | 0.123 | 0.001 | | 16.840 |  |
|  |  | rs2692012 | G | A | 0.945 | -0.114 | 0.025 | 1.56E-06 | 0.069 | 0.047 | 0.138 | 0.001 | | 24.993 |  |
|  |  | rs2838334 | G | A | 0.344 | 0.057 | 0.012 | 3.82E-06 | 0.000 | 0.022 | 0.999 | 0.001 | | 27.060 |  |
|  |  | rs3935584 | C | T | 0.532 | -0.053 | 0.012 | 6.78E-06 | -0.004 | 0.021 | 0.857 | 0.001 | | 25.285 |  |
|  |  | rs4506934 | C | T | 0.118 | -0.094 | 0.020 | 3.16E-06 | -0.034 | 0.033 | 0.304 | 0.002 | | 34.084 |  |
|  |  | rs6058181 | C | T | 0.162 | 0.083 | 0.017 | 2.70E-07 | -0.032 | 0.029 | 0.273 | 0.002 | | 34.742 |  |
|  |  | rs72647048 | T | C | 0.112 | -0.077 | 0.017 | 9.61E-06 | -0.013 | 0.033 | 0.701 | 0.001 | | 21.691 |  |
|  |  | rs9928243 | C | A | 0.471 | -0.054 | 0.012 | 4.48E-06 | 0.044 | 0.021 | 0.037 | 0.001 | | 26.859 |  |
| ebi-a-GCST90016936 | Gut microbiota abundance (family Enterobacteriaceae id.3469) | rs11026530 | T | C | 0.150 | 0.082 | 0.019 | 9.43E-06 | 0.041 | 0.030 | 0.172 | 0.002 | | 31.665 |  |
|  |  | rs2374342 | C | A | 0.415 | 0.058 | 0.013 | 4.52E-06 | 0.024 | 0.022 | 0.256 | 0.002 | | 30.300 |  |
|  |  | rs35673018 | G | A | 0.090 | 0.090 | 0.020 | 7.63E-06 | -0.042 | 0.037 | 0.250 | 0.001 | | 24.373 |  |
|  |  | rs504442 | T | G | 0.105 | 0.084 | 0.019 | 5.17E-06 | 0.049 | 0.035 | 0.158 | 0.001 | | 24.424 |  |
|  |  | rs62210023 | A | G | 0.345 | 0.061 | 0.013 | 3.13E-06 | 0.033 | 0.022 | 0.143 | 0.002 | | 30.563 |  |
|  |  | rs78143293 | A | G | 0.122 | -0.085 | 0.017 | 1.20E-06 | 0.078 | 0.032 | 0.016 | 0.002 | | 28.369 |  |
|  |  | rs79757635 | C | A | 0.137 | 0.076 | 0.017 | 9.32E-06 | -0.049 | 0.031 | 0.117 | 0.001 | | 24.957 |  |
| ebi-a-GCST90016937 | Gut microbiota abundance (family Erysipelotrichaceae id.2149) | rs1074800 | G | A | 0.581 | -0.049 | 0.011 | 6.15E-06 | 0.009 | 0.022 | 0.673 | 0.001 | | 21.674 |  |
|  |  | rs10781552 | C | T | 0.281 | -0.055 | 0.012 | 2.33E-06 | 0.029 | 0.024 | 0.220 | 0.001 | | 22.609 |  |
|  |  | rs17530232 | A | G | 0.053 | 0.103 | 0.022 | 2.79E-06 | 0.007 | 0.047 | 0.880 | 0.001 | | 19.513 |  |
|  |  | rs1884466 | C | T | 0.520 | -0.048 | 0.011 | 9.53E-06 | -0.009 | 0.021 | 0.684 | 0.001 | | 20.710 |  |
|  |  | rs2300774 | A | G | 0.561 | -0.052 | 0.011 | 8.95E-07 | 0.015 | 0.021 | 0.495 | 0.001 | | 24.848 |  |
|  |  | rs290833 | T | G | 0.493 | -0.050 | 0.011 | 8.03E-06 | 0.029 | 0.021 | 0.178 | 0.001 | | 22.706 |  |
|  |  | rs35161940 | T | C | 0.107 | -0.081 | 0.017 | 1.85E-06 | 0.031 | 0.034 | 0.361 | 0.001 | | 22.815 |  |
|  |  | rs4078432 | T | C | 0.827 | 0.061 | 0.013 | 4.23E-06 | -0.030 | 0.028 | 0.293 | 0.001 | | 19.477 |  |
|  |  | rs56970041 | T | G | 0.061 | 0.072 | 0.016 | 5.40E-06 | 0.017 | 0.044 | 0.700 | 0.001 | | 11.067 |  |
|  |  | rs62504403 | C | T | 0.195 | 0.068 | 0.013 | 1.12E-07 | -0.002 | 0.027 | 0.933 | 0.001 | | 26.782 |  |
|  |  | rs7234058 | T | C | 0.090 | -0.095 | 0.019 | 9.12E-07 | -0.019 | 0.037 | 0.607 | 0.001 | | 27.034 |  |
|  |  | rs7826267 | G | T | 0.931 | 0.084 | 0.020 | 9.28E-06 | 0.035 | 0.043 | 0.416 | 0.001 | | 16.669 |  |
|  |  | rs8003149 | C | T | 0.333 | 0.054 | 0.012 | 4.08E-06 | 0.012 | 0.022 | 0.591 | 0.001 | | 23.661 |  |
| ebi-a-GCST90016938 | Gut microbiota abundance (family Family XI id.1936) | rs10759623 | C | T | 0.227 | -0.162 | 0.032 | 5.78E-07 | 0.015 | 0.025 | 0.545 | 0.009 | | 170.656 |  |
|  |  | rs11547158 | A | G | 0.135 | -0.178 | 0.037 | 2.70E-06 | -0.036 | 0.031 | 0.252 | 0.007 | | 135.660 |  |
|  |  | rs17379710 | T | C | 0.410 | -0.116 | 0.025 | 3.97E-06 | 0.018 | 0.022 | 0.401 | 0.007 | | 120.972 |  |
|  |  | rs2155352 | A | G | 0.236 | -0.151 | 0.030 | 6.63E-07 | 0.024 | 0.025 | 0.330 | 0.008 | | 150.869 |  |
|  |  | rs2156611 | T | C | 0.501 | -0.112 | 0.025 | 9.43E-06 | 0.004 | 0.021 | 0.861 | 0.006 | | 116.754 |  |
|  |  | rs3733511 | A | G | 0.299 | 0.128 | 0.027 | 3.39E-06 | -0.020 | 0.023 | 0.400 | 0.007 | | 127.398 |  |
|  |  | rs488164 | G | T | 0.646 | -0.118 | 0.026 | 4.80E-06 | -0.002 | 0.022 | 0.930 | 0.006 | | 117.588 |  |
|  |  | rs697771 | A | G | 0.457 | -0.118 | 0.025 | 3.19E-06 | -0.012 | 0.021 | 0.578 | 0.007 | | 126.891 |  |
| ebi-a-GCST90016939 | Gut microbiota abundance (family Family XIII id.1957) | rs10404377 | A | C | 0.579 | 0.050 | 0.011 | 6.99E-06 | -0.011 | 0.022 | 0.627 | 0.001 | | 22.658 |  |
|  |  | rs118170811 | A | G | 0.031 | 0.152 | 0.032 | 1.80E-06 | 0.021 | 0.061 | 0.733 | 0.001 | | 25.211 |  |
|  |  | rs12904405 | A | G | 0.096 | -0.085 | 0.019 | 7.67E-06 | 0.037 | 0.036 | 0.305 | 0.001 | | 23.033 |  |
|  |  | rs3098182 | T | G | 0.481 | -0.051 | 0.011 | 4.04E-06 | 0.064 | 0.021 | 0.002 | 0.001 | | 23.706 |  |
|  |  | rs482905 | G | T | 0.248 | 0.060 | 0.013 | 3.72E-06 | 0.007 | 0.024 | 0.780 | 0.001 | | 24.385 |  |
|  |  | rs6501525 | A | G | 0.340 | 0.056 | 0.012 | 1.24E-06 | 0.003 | 0.022 | 0.904 | 0.001 | | 25.998 |  |
|  |  | rs66753613 | G | A | 0.184 | 0.065 | 0.014 | 8.08E-06 | -0.011 | 0.028 | 0.694 | 0.001 | | 23.387 |  |
|  |  | rs6797051 | C | T | 0.095 | -0.081 | 0.017 | 4.89E-06 | -0.002 | 0.036 | 0.967 | 0.001 | | 20.447 |  |
|  |  | rs7514702 | T | C | 0.151 | -0.066 | 0.014 | 3.92E-06 | -0.029 | 0.030 | 0.330 | 0.001 | | 20.661 |  |
| ebi-a-GCST90016940 | Gut microbiota abundance (family Lachnospiraceae id.1987) | rs10402491 | C | T | 0.168 | 0.066 | 0.015 | 7.58E-06 | 0.012 | 0.028 | 0.661 | 0.001 | | 22.516 |  |
|  |  | rs11139361 | C | T | 0.678 | -0.049 | 0.011 | 4.26E-06 | 0.005 | 0.023 | 0.822 | 0.001 | | 19.601 |  |
|  |  | rs112040820 | A | G | 0.261 | 0.055 | 0.012 | 2.42E-06 | 0.059 | 0.024 | 0.015 | 0.001 | | 21.397 |  |
|  |  | rs11841382 | G | T | 0.083 | -0.072 | 0.017 | 9.58E-06 | -0.012 | 0.038 | 0.746 | 0.001 | | 14.440 |  |
|  |  | rs11979110 | T | C | 0.464 | -0.050 | 0.011 | 1.82E-06 | 0.008 | 0.021 | 0.717 | 0.001 | | 22.915 |  |
|  |  | rs1205443 | A | G | 0.352 | 0.050 | 0.011 | 7.29E-06 | -0.001 | 0.022 | 0.962 | 0.001 | | 21.057 |  |
|  |  | rs12760724 | A | C | 0.329 | -0.048 | 0.011 | 7.27E-06 | -0.005 | 0.023 | 0.829 | 0.001 | | 19.016 |  |
|  |  | rs13005175 | G | A | 0.956 | -0.099 | 0.022 | 8.37E-06 | -0.048 | 0.051 | 0.349 | 0.001 | | 15.405 |  |
|  |  | rs2159863 | A | G | 0.181 | -0.059 | 0.013 | 3.70E-06 | 0.016 | 0.028 | 0.566 | 0.001 | | 18.646 |  |
|  |  | rs2910921 | C | T | 0.971 | -0.160 | 0.036 | 8.42E-06 | 0.054 | 0.063 | 0.386 | 0.001 | | 26.488 |  |
|  |  | rs3127230 | C | T | 0.304 | -0.050 | 0.011 | 6.20E-06 | -0.029 | 0.023 | 0.216 | 0.001 | | 19.704 |  |
|  |  | rs35524804 | T | C | 0.214 | -0.061 | 0.013 | 2.45E-06 | 0.004 | 0.026 | 0.866 | 0.001 | | 22.794 |  |
|  |  | rs7359994 | C | T | 0.597 | 0.050 | 0.011 | 5.36E-06 | 0.003 | 0.022 | 0.906 | 0.001 | | 22.365 |  |
|  |  | rs79086868 | T | C | 0.101 | 0.078 | 0.016 | 3.01E-06 | -0.031 | 0.035 | 0.386 | 0.001 | | 20.095 |  |
|  |  | rs959845 | T | C | 0.573 | 0.049 | 0.011 | 5.17E-06 | -0.033 | 0.022 | 0.132 | 0.001 | | 21.894 |  |
|  |  | rs9929145 | G | A | 0.049 | -0.126 | 0.025 | 2.84E-07 | -0.079 | 0.049 | 0.105 | 0.001 | | 27.121 |  |
| ebi-a-GCST90016941 | Gut microbiota abundance (family Lactobacillaceae id.1836) | rs1530559 | G | A | 0.463 | 0.077 | 0.018 | 9.65E-06 | 0.040 | 0.021 | 0.065 | 0.003 | | 54.572 |  |
|  |  | rs16861661 | G | A | 0.067 | -0.193 | 0.038 | 2.70E-07 | 0.030 | 0.043 | 0.483 | 0.005 | | 85.588 |  |
|  |  | rs62314653 | C | A | 0.059 | 0.177 | 0.039 | 6.59E-06 | 0.010 | 0.045 | 0.830 | 0.004 | | 64.514 |  |
|  |  | rs74599091 | A | G | 0.019 | 0.192 | 0.043 | 7.70E-06 | -0.005 | 0.079 | 0.946 | 0.001 | | 24.522 |  |
|  |  | rs768253 | T | G | 0.440 | -0.079 | 0.017 | 3.61E-06 | 0.019 | 0.021 | 0.373 | 0.003 | | 56.905 |  |
|  |  | rs77478751 | A | G | 0.119 | -0.219 | 0.047 | 5.96E-06 | -0.021 | 0.033 | 0.532 | 0.010 | | 187.004 |  |
|  |  | rs921925 | A | C | 0.213 | 0.100 | 0.020 | 5.77E-07 | 0.001 | 0.026 | 0.964 | 0.003 | | 61.552 |  |
|  |  | rs9345899 | A | G | 0.105 | -0.124 | 0.028 | 9.45E-06 | 0.027 | 0.035 | 0.432 | 0.003 | | 53.095 |  |
| ebi-a-GCST90016942 | Gut microbiota abundance (family Methanobacteriaceae id.121) | rs10202904 | G | T | 0.592 | 0.122 | 0.024 | 3.01E-07 | -0.024 | 0.022 | 0.263 | 0.007 | | 132.297 |  |
|  |  | rs10424197 | A | G | 0.743 | 0.111 | 0.025 | 9.28E-06 | -0.016 | 0.024 | 0.501 | 0.005 | | 87.104 |  |
|  |  | rs4257531 | G | A | 0.106 | 0.164 | 0.036 | 7.44E-06 | -0.024 | 0.035 | 0.487 | 0.005 | | 94.137 |  |
|  |  | rs56131665 | G | A | 0.107 | 0.179 | 0.039 | 6.18E-06 | 0.020 | 0.034 | 0.564 | 0.006 | | 112.919 |  |
|  |  | rs6508769 | C | T | 0.853 | -0.154 | 0.034 | 8.23E-06 | 0.011 | 0.030 | 0.704 | 0.006 | | 108.783 |  |
|  |  | rs6776814 | T | C | 0.020 | -0.200 | 0.041 | 1.63E-06 | 0.017 | 0.074 | 0.819 | 0.002 | | 29.335 |  |
|  |  | rs73068003 | G | T | 0.096 | -0.158 | 0.035 | 8.45E-06 | 0.052 | 0.036 | 0.144 | 0.004 | | 80.073 |  |
|  |  | rs73457410 | A | G | 0.064 | 0.215 | 0.044 | 1.41E-06 | 0.010 | 0.043 | 0.809 | 0.006 | | 101.879 |  |
|  |  | rs75208022 | C | T | 0.093 | -0.227 | 0.049 | 5.92E-06 | -0.018 | 0.036 | 0.615 | 0.009 | | 161.898 |  |
|  |  | rs894996 | C | A | 0.070 | 0.217 | 0.045 | 1.88E-06 | 0.028 | 0.041 | 0.492 | 0.006 | | 113.062 |  |
| ebi-a-GCST90016943 | Gut microbiota abundance (family Oxalobacteraceae id.2966) | rs111966731 | T | C | 0.086 | 0.204 | 0.045 | 4.56E-06 | -0.020 | 0.038 | 0.604 | 0.007 | | 121.198 |  |
|  |  | rs11246212 | C | T | 0.861 | -0.136 | 0.029 | 4.51E-06 | -0.003 | 0.031 | 0.922 | 0.004 | | 81.878 |  |
|  |  | rs12002250 | A | C | 0.043 | 0.196 | 0.045 | 5.53E-06 | -0.004 | 0.052 | 0.947 | 0.003 | | 58.120 |  |
|  |  | rs1569853 | T | C | 0.121 | -0.140 | 0.028 | 7.45E-07 | -0.027 | 0.032 | 0.406 | 0.004 | | 76.824 |  |
|  |  | rs17138946 | G | T | 0.066 | -0.189 | 0.043 | 8.09E-06 | -0.014 | 0.042 | 0.742 | 0.004 | | 81.984 |  |
|  |  | rs36057338 | G | T | 0.033 | 0.182 | 0.040 | 6.26E-06 | -0.042 | 0.059 | 0.480 | 0.002 | | 38.782 |  |
|  |  | rs4428215 | G | A | 0.253 | 0.126 | 0.023 | 4.88E-08 | -0.015 | 0.024 | 0.535 | 0.006 | | 110.135 |  |
|  |  | rs561239 | A | G | 0.210 | 0.106 | 0.024 | 7.19E-06 | 0.044 | 0.026 | 0.090 | 0.004 | | 68.006 |  |
|  |  | rs6000536 | C | T | 0.169 | -0.118 | 0.024 | 7.39E-07 | 0.022 | 0.028 | 0.445 | 0.004 | | 72.368 |  |
|  |  | rs62435498 | C | A | 0.103 | 0.182 | 0.040 | 7.46E-06 | -0.077 | 0.035 | 0.029 | 0.006 | | 111.947 |  |
|  |  | rs736744 | T | C | 0.568 | -0.106 | 0.020 | 1.49E-07 | -0.018 | 0.021 | 0.401 | 0.005 | | 100.994 |  |
|  |  | rs7993559 | A | C | 0.554 | -0.092 | 0.020 | 5.04E-06 | -0.014 | 0.022 | 0.524 | 0.004 | | 77.188 |  |
|  |  | rs80330081 | A | C | 0.105 | -0.188 | 0.042 | 6.64E-06 | -0.041 | 0.035 | 0.237 | 0.007 | | 121.538 |  |
|  |  | rs934049 | G | A | 0.211 | 0.110 | 0.024 | 4.21E-06 | -0.007 | 0.026 | 0.803 | 0.004 | | 73.945 |  |
| ebi-a-GCST90016944 | Gut microbiota abundance (family Pasteurellaceae id.3689) | rs10965428 | C | A | 0.056 | -0.120 | 0.026 | 4.29E-06 | 0.056 | 0.046 | 0.223 | 0.002 | | 28.099 |  |
|  |  | rs111582866 | G | A | 0.087 | -0.114 | 0.026 | 7.07E-06 | -0.005 | 0.038 | 0.892 | 0.002 | | 37.874 |  |
|  |  | rs12050685 | A | G | 0.280 | -0.067 | 0.015 | 9.19E-06 | -0.033 | 0.023 | 0.160 | 0.002 | | 33.368 |  |
|  |  | rs16970009 | A | G | 0.020 | 0.187 | 0.043 | 7.32E-06 | 0.002 | 0.075 | 0.982 | 0.001 | | 25.448 |  |
|  |  | rs35510 | A | G | 0.043 | 0.123 | 0.026 | 4.02E-06 | -0.030 | 0.052 | 0.567 | 0.001 | | 22.584 |  |
|  |  | rs4822728 | T | C | 0.483 | 0.069 | 0.015 | 4.72E-06 | 0.054 | 0.021 | 0.012 | 0.002 | | 43.137 |  |
|  |  | rs6972479 | A | G | 0.205 | -0.078 | 0.018 | 7.75E-06 | 0.059 | 0.026 | 0.025 | 0.002 | | 36.652 |  |
|  |  | rs72756943 | G | A | 0.066 | 0.140 | 0.030 | 3.35E-06 | -0.008 | 0.043 | 0.844 | 0.002 | | 44.456 |  |
|  |  | rs73139353 | A | C | 0.088 | -0.223 | 0.048 | 8.71E-06 | 0.022 | 0.038 | 0.559 | 0.008 | | 146.924 |  |
|  |  | rs76022354 | C | T | 0.050 | 0.243 | 0.050 | 1.83E-06 | 0.000 | 0.049 | 0.997 | 0.006 | | 102.982 |  |
|  |  | rs78909003 | T | C | 0.055 | -0.241 | 0.050 | 2.05E-06 | -0.019 | 0.047 | 0.688 | 0.006 | | 111.210 |  |
|  |  | rs9382510 | C | T | 0.265 | -0.088 | 0.017 | 2.48E-07 | 0.020 | 0.024 | 0.415 | 0.003 | | 55.710 |  |
|  |  | rs9895850 | T | C | 0.044 | -0.176 | 0.041 | 9.08E-06 | -0.054 | 0.052 | 0.300 | 0.003 | | 48.051 |  |
|  |  | rs9938097 | C | T | 0.611 | 0.071 | 0.016 | 8.23E-06 | 0.035 | 0.022 | 0.108 | 0.002 | | 44.097 |  |
| ebi-a-GCST90016945 | Gut microbiota abundance (family Peptococcaceae id.2024) | rs117452796 | A | G | 0.031 | -0.258 | 0.055 | 3.15E-06 | -0.046 | 0.061 | 0.445 | 0.004 | | 74.749 |  |
|  |  | rs12144792 | C | T | 0.376 | 0.064 | 0.014 | 5.82E-06 | 0.009 | 0.022 | 0.671 | 0.002 | | 35.745 |  |
|  |  | rs12634826 | T | G | 0.382 | -0.074 | 0.015 | 1.01E-06 | 0.015 | 0.022 | 0.509 | 0.003 | | 47.512 |  |
|  |  | rs12992764 | T | G | 0.430 | 0.068 | 0.014 | 1.46E-06 | -0.004 | 0.022 | 0.837 | 0.002 | | 42.177 |  |
|  |  | rs150600492 | A | C | 0.058 | 0.136 | 0.029 | 2.31E-06 | -0.051 | 0.046 | 0.269 | 0.002 | | 36.798 |  |
|  |  | rs35703006 | G | T | 0.245 | 0.081 | 0.016 | 4.95E-07 | 0.016 | 0.025 | 0.508 | 0.002 | | 44.851 |  |
|  |  | rs4990837 | G | A | 0.803 | -0.091 | 0.019 | 1.74E-06 | 0.045 | 0.027 | 0.096 | 0.003 | | 48.138 |  |
|  |  | rs75430375 | C | T | 0.046 | -0.148 | 0.032 | 3.41E-06 | 0.065 | 0.050 | 0.196 | 0.002 | | 34.987 |  |
|  |  | rs75898026 | A | G | 0.211 | -0.082 | 0.017 | 2.02E-06 | 0.021 | 0.026 | 0.425 | 0.002 | | 41.298 |  |
| ebi-a-GCST90016946 | Gut microbiota abundance (family Peptostreptococcaceae id.2042) | rs10805326 | A | G | 0.706 | -0.057 | 0.012 | 4.03E-06 | -0.041 | 0.023 | 0.078 | 0.001 | | 24.475 |  |
|  |  | rs117020988 | C | T | 0.080 | 0.182 | 0.037 | 1.03E-06 | 0.013 | 0.039 | 0.749 | 0.005 | | 90.663 |  |
|  |  | rs12377846 | C | A | 0.029 | -0.252 | 0.051 | 7.26E-07 | 0.004 | 0.063 | 0.946 | 0.004 | | 64.913 |  |
|  |  | rs12986312 | T | G | 0.299 | 0.057 | 0.013 | 5.77E-06 | 0.026 | 0.023 | 0.267 | 0.001 | | 25.189 |  |
|  |  | rs1467258 | G | A | 0.188 | 0.073 | 0.016 | 7.90E-06 | 0.009 | 0.027 | 0.756 | 0.002 | | 29.520 |  |
|  |  | rs1520207 | T | C | 0.501 | -0.053 | 0.011 | 3.17E-06 | -0.025 | 0.021 | 0.244 | 0.001 | | 25.370 |  |
|  |  | rs4692811 | C | T | 0.341 | 0.064 | 0.013 | 4.21E-07 | -0.024 | 0.022 | 0.278 | 0.002 | | 34.036 |  |
|  |  | rs59865771 | C | T | 0.341 | -0.057 | 0.013 | 7.69E-06 | 0.002 | 0.022 | 0.927 | 0.001 | | 27.176 |  |
|  |  | rs61841503 | G | A | 0.129 | 0.092 | 0.016 | 9.80E-09 | 0.011 | 0.031 | 0.722 | 0.002 | | 34.989 |  |
|  |  | rs6721459 | G | A | 0.407 | -0.051 | 0.011 | 5.08E-06 | -0.015 | 0.022 | 0.499 | 0.001 | | 23.028 |  |
|  |  | rs76982728 | T | C | 0.021 | 0.124 | 0.027 | 3.24E-06 | -0.022 | 0.073 | 0.765 | 0.001 | | 11.775 |  |
|  |  | rs77540684 | T | G | 0.096 | 0.107 | 0.025 | 8.14E-06 | 0.031 | 0.036 | 0.384 | 0.002 | | 36.309 |  |
|  |  | rs9573937 | A | G | 0.165 | -0.069 | 0.014 | 1.71E-06 | 0.020 | 0.029 | 0.487 | 0.001 | | 24.308 |  |
| ebi-a-GCST90016947 | Gut microbiota abundance (family Porphyromonadaceae id.943) | rs10762312 | A | G | 0.690 | 0.052 | 0.012 | 8.70E-06 | 0.019 | 0.023 | 0.414 | 0.001 | | 21.559 |  |
|  |  | rs10858364 | G | T | 0.251 | 0.055 | 0.012 | 4.31E-06 | 0.019 | 0.025 | 0.442 | 0.001 | | 21.139 |  |
|  |  | rs17065783 | A | G | 0.158 | -0.059 | 0.012 | 1.79E-06 | 0.047 | 0.029 | 0.100 | 0.001 | | 17.086 |  |
|  |  | rs1980561 | A | G | 0.476 | -0.049 | 0.011 | 8.95E-06 | -0.008 | 0.021 | 0.695 | 0.001 | | 21.567 |  |
|  |  | rs35233670 | T | C | 0.525 | -0.047 | 0.011 | 7.91E-06 | 0.019 | 0.021 | 0.364 | 0.001 | | 20.537 |  |
|  |  | rs35961441 | A | C | 0.038 | 0.092 | 0.021 | 8.37E-06 | -0.031 | 0.055 | 0.571 | 0.001 | | 11.232 |  |
|  |  | rs6953849 | A | G | 0.179 | 0.072 | 0.015 | 2.44E-06 | 0.023 | 0.028 | 0.403 | 0.002 | | 27.791 |  |
|  |  | rs7330827 | T | C | 0.060 | -0.104 | 0.024 | 8.05E-06 | -0.040 | 0.045 | 0.376 | 0.001 | | 22.207 |  |
|  |  | rs864093 | A | C | 0.211 | -0.053 | 0.012 | 9.60E-06 | 0.012 | 0.026 | 0.642 | 0.001 | | 17.034 |  |
| ebi-a-GCST90016948 | Gut microbiota abundance (family Prevotellaceae id.960) | rs12057990 | C | T | 0.269 | 0.059 | 0.013 | 8.97E-06 | -0.009 | 0.024 | 0.695 | 0.001 | | 25.123 |  |
|  |  | rs12118202 | T | C | 0.182 | -0.075 | 0.015 | 5.54E-07 | 0.023 | 0.027 | 0.393 | 0.002 | | 30.862 |  |
|  |  | rs13069367 | A | C | 0.408 | -0.054 | 0.012 | 7.39E-06 | -0.061 | 0.022 | 0.005 | 0.001 | | 25.836 |  |
|  |  | rs148376875 | T | G | 0.153 | 0.085 | 0.018 | 2.08E-06 | -0.013 | 0.030 | 0.668 | 0.002 | | 33.975 |  |
|  |  | rs2206482 | T | G | 0.395 | -0.057 | 0.012 | 1.30E-06 | 0.014 | 0.022 | 0.514 | 0.002 | | 28.441 |  |
|  |  | rs2278540 | G | A | 0.364 | 0.055 | 0.012 | 8.44E-06 | -0.032 | 0.022 | 0.146 | 0.001 | | 26.104 |  |
|  |  | rs34660375 | A | G | 0.154 | -0.081 | 0.018 | 7.40E-06 | 0.007 | 0.030 | 0.808 | 0.002 | | 31.136 |  |
|  |  | rs3758087 | C | T | 0.723 | -0.056 | 0.012 | 8.61E-06 | -0.046 | 0.024 | 0.051 | 0.001 | | 23.390 |  |
|  |  | rs3860225 | A | G | 0.091 | 0.084 | 0.017 | 5.50E-07 | 0.079 | 0.037 | 0.032 | 0.001 | | 21.312 |  |
|  |  | rs4493272 | T | C | 0.477 | -0.060 | 0.012 | 3.02E-07 | 0.028 | 0.021 | 0.191 | 0.002 | | 33.551 |  |
|  |  | rs4685827 | T | C | 0.241 | -0.068 | 0.015 | 2.77E-06 | 0.040 | 0.025 | 0.103 | 0.002 | | 31.066 |  |
|  |  | rs7252711 | G | A | 0.891 | 0.074 | 0.016 | 5.57E-06 | -0.019 | 0.034 | 0.570 | 0.001 | | 19.635 |  |
|  |  | rs7975087 | C | A | 0.176 | -0.060 | 0.014 | 7.59E-06 | -0.061 | 0.028 | 0.029 | 0.001 | | 19.235 |  |
|  |  | rs912860 | A | G | 0.977 | 0.229 | 0.048 | 9.30E-07 | 0.019 | 0.070 | 0.783 | 0.002 | | 43.523 |  |
|  |  | rs9586501 | G | A | 0.262 | 0.059 | 0.013 | 2.59E-06 | -0.022 | 0.024 | 0.350 | 0.001 | | 24.957 |  |
|  |  | rs9958960 | G | A | 0.154 | -0.091 | 0.017 | 1.06E-07 | -0.053 | 0.029 | 0.070 | 0.002 | | 39.990 |  |
| ebi-a-GCST90016949 | Gut microbiota abundance (family Rhodospirillaceae id.2717) | rs1035406 | G | A | 0.116 | -0.114 | 0.025 | 5.84E-06 | -0.001 | 0.033 | 0.972 | 0.003 | | 48.648 |  |
|  |  | rs11591293 | G | T | 0.424 | 0.074 | 0.016 | 2.67E-06 | -0.006 | 0.021 | 0.774 | 0.003 | | 49.594 |  |
|  |  | rs13336560 | C | T | 0.545 | -0.070 | 0.016 | 9.17E-06 | -0.022 | 0.022 | 0.317 | 0.002 | | 44.899 |  |
|  |  | rs1549633 | A | C | 0.116 | 0.100 | 0.022 | 4.70E-06 | 0.059 | 0.033 | 0.075 | 0.002 | | 37.578 |  |
|  |  | rs1923415 | A | G | 0.088 | -0.100 | 0.023 | 9.64E-06 | 0.034 | 0.037 | 0.360 | 0.002 | | 29.455 |  |
|  |  | rs3754624 | C | T | 0.177 | 0.097 | 0.020 | 1.71E-06 | 0.031 | 0.028 | 0.259 | 0.003 | | 50.529 |  |
|  |  | rs4278423 | T | C | 0.062 | 0.108 | 0.024 | 3.12E-06 | 0.036 | 0.044 | 0.415 | 0.001 | | 24.714 |  |
|  |  | rs55876211 | C | T | 0.254 | -0.091 | 0.020 | 2.87E-06 | 0.015 | 0.024 | 0.531 | 0.003 | | 58.026 |  |
|  |  | rs61933850 | G | A | 0.139 | 0.165 | 0.036 | 7.23E-06 | -0.039 | 0.031 | 0.217 | 0.006 | | 119.856 |  |
|  |  | rs6679026 | T | C | 0.101 | 0.112 | 0.025 | 9.95E-06 | -0.066 | 0.035 | 0.060 | 0.002 | | 41.783 |  |
|  |  | rs7001029 | C | T | 0.093 | 0.117 | 0.026 | 5.35E-06 | 0.042 | 0.037 | 0.255 | 0.002 | | 42.532 |  |
|  |  | rs72714493 | A | G | 0.153 | 0.082 | 0.018 | 7.35E-06 | -0.051 | 0.030 | 0.081 | 0.002 | | 31.701 |  |
|  |  | rs74354280 | C | T | 0.279 | -0.091 | 0.020 | 6.67E-06 | -0.023 | 0.024 | 0.328 | 0.003 | | 61.556 |  |
|  |  | rs76784716 | A | G | 0.114 | 0.136 | 0.029 | 1.49E-06 | 0.010 | 0.034 | 0.773 | 0.004 | | 68.824 |  |
|  |  | rs9813022 | A | G | 0.376 | -0.084 | 0.016 | 2.53E-07 | -0.003 | 0.022 | 0.909 | 0.003 | | 61.243 |  |
| ebi-a-GCST90016950 | Gut microbiota abundance (family Rikenellaceae id.967) | rs10217435 | C | T | 0.140 | -0.088 | 0.020 | 6.51E-06 | -0.008 | 0.031 | 0.804 | 0.002 | | 34.636 |  |
|  |  | rs10832801 | A | C | 0.284 | -0.053 | 0.012 | 7.50E-06 | 0.034 | 0.024 | 0.154 | 0.001 | | 21.352 |  |
|  |  | rs1939881 | G | A | 0.059 | -0.106 | 0.021 | 5.64E-07 | 0.019 | 0.045 | 0.676 | 0.001 | | 22.668 |  |
|  |  | rs2447496 | A | G | 0.732 | 0.055 | 0.012 | 6.09E-06 | 0.040 | 0.024 | 0.094 | 0.001 | | 21.753 |  |
|  |  | rs2833282 | G | A | 0.153 | 0.071 | 0.016 | 4.31E-06 | 0.015 | 0.030 | 0.612 | 0.001 | | 24.042 |  |
|  |  | rs35909684 | A | C | 0.073 | -0.085 | 0.019 | 7.10E-06 | -0.010 | 0.040 | 0.814 | 0.001 | | 18.068 |  |
|  |  | rs36021379 | A | G | 0.159 | -0.066 | 0.014 | 7.20E-06 | 0.030 | 0.029 | 0.305 | 0.001 | | 21.097 |  |
|  |  | rs4264350 | T | C | 0.460 | -0.053 | 0.011 | 1.35E-06 | -0.029 | 0.021 | 0.167 | 0.001 | | 25.278 |  |
|  |  | rs59663348 | G | A | 0.246 | 0.057 | 0.013 | 6.12E-06 | -0.005 | 0.025 | 0.825 | 0.001 | | 22.225 |  |
|  |  | rs62532512 | A | C | 0.568 | 0.050 | 0.011 | 2.76E-06 | 0.000 | 0.021 | 0.987 | 0.001 | | 22.908 |  |
|  |  | rs6744030 | C | T | 0.214 | 0.070 | 0.016 | 9.32E-06 | 0.019 | 0.026 | 0.468 | 0.002 | | 29.997 |  |
|  |  | rs6837275 | A | G | 0.301 | 0.057 | 0.012 | 1.45E-06 | 0.035 | 0.023 | 0.136 | 0.001 | | 25.127 |  |
|  |  | rs74474130 | T | G | 0.036 | 0.138 | 0.030 | 3.61E-06 | -0.010 | 0.058 | 0.858 | 0.001 | | 23.994 |  |
|  |  | rs77885767 | C | T | 0.046 | -0.156 | 0.034 | 2.85E-06 | -0.005 | 0.050 | 0.922 | 0.002 | | 39.390 |  |
|  |  | rs9389714 | C | T | 0.088 | -0.064 | 0.014 | 8.79E-06 | 0.011 | 0.037 | 0.768 | 0.001 | | 11.960 |  |
|  |  | rs9578457 | G | A | 0.048 | -0.141 | 0.032 | 3.99E-06 | -0.086 | 0.049 | 0.083 | 0.002 | | 33.767 |  |
|  |  | rs9603208 | G | T | 0.101 | 0.082 | 0.016 | 1.92E-07 | -0.003 | 0.035 | 0.942 | 0.001 | | 22.404 |  |
| ebi-a-GCST90016951 | Gut microbiota abundance (family Ruminococcaceae id.2050) | rs10093275 | T | C | 0.669 | -0.053 | 0.012 | 5.35E-06 | -0.013 | 0.023 | 0.568 | 0.001 | | 23.247 |  |
|  |  | rs10166469 | C | T | 0.746 | 0.053 | 0.012 | 8.52E-06 | 0.028 | 0.024 | 0.250 | 0.001 | | 19.775 |  |
|  |  | rs1612733 | T | C | 0.055 | 0.109 | 0.024 | 4.22E-06 | -0.034 | 0.047 | 0.464 | 0.001 | | 22.365 |  |
|  |  | rs17376049 | T | C | 0.109 | 0.085 | 0.017 | 7.30E-07 | 0.025 | 0.034 | 0.467 | 0.001 | | 25.620 |  |
|  |  | rs2113833 | C | T | 0.967 | -0.169 | 0.036 | 1.14E-06 | -0.038 | 0.059 | 0.526 | 0.002 | | 33.251 |  |
|  |  | rs3009418 | A | C | 0.953 | -0.093 | 0.021 | 8.69E-06 | -0.030 | 0.050 | 0.553 | 0.001 | | 14.203 |  |
|  |  | rs55793120 | T | C | 0.062 | 0.138 | 0.027 | 1.44E-07 | 0.021 | 0.045 | 0.638 | 0.002 | | 40.605 |  |
|  |  | rs56199908 | T | C | 0.052 | -0.199 | 0.041 | 1.66E-06 | -0.010 | 0.048 | 0.826 | 0.004 | | 72.589 |  |
|  |  | rs76724913 | T | G | 0.097 | 0.090 | 0.020 | 9.60E-06 | 0.033 | 0.036 | 0.356 | 0.001 | | 26.237 |  |
| ebi-a-GCST90016952 | Gut microbiota abundance (family Streptococcaceae id.1850) | rs10028567 | C | T | 0.121 | -0.093 | 0.019 | 3.72E-06 | -0.037 | 0.033 | 0.253 | 0.002 | | 34.021 |  |
|  |  | rs11110281 | T | C | 0.048 | -0.131 | 0.023 | 1.40E-08 | 0.005 | 0.049 | 0.922 | 0.002 | | 28.644 |  |
|  |  | rs16950051 | A | G | 0.066 | 0.107 | 0.024 | 5.34E-06 | 0.061 | 0.043 | 0.153 | 0.001 | | 25.749 |  |
|  |  | rs2370083 | G | T | 0.065 | -0.084 | 0.018 | 4.26E-06 | -0.020 | 0.043 | 0.642 | 0.001 | | 15.740 |  |
|  |  | rs2952251 | G | A | 0.771 | 0.064 | 0.013 | 3.72E-07 | 0.016 | 0.027 | 0.548 | 0.001 | | 26.479 |  |
|  |  | rs35344081 | G | A | 0.263 | 0.061 | 0.013 | 2.64E-06 | 0.008 | 0.024 | 0.725 | 0.001 | | 26.402 |  |
|  |  | rs4968759 | A | G | 0.486 | -0.054 | 0.011 | 8.92E-07 | 0.000 | 0.021 | 0.993 | 0.001 | | 27.155 |  |
|  |  | rs57646748 | G | A | 0.039 | -0.088 | 0.020 | 7.88E-06 | 0.008 | 0.054 | 0.882 | 0.001 | | 10.559 |  |
|  |  | rs6806351 | T | C | 0.219 | -0.062 | 0.014 | 6.94E-06 | -0.052 | 0.026 | 0.041 | 0.001 | | 24.107 |  |
|  |  | rs77968078 | G | A | 0.061 | -0.099 | 0.022 | 7.93E-06 | -0.006 | 0.044 | 0.895 | 0.001 | | 20.704 |  |
|  |  | rs7916711 | A | G | 0.138 | 0.096 | 0.022 | 6.33E-06 | -0.012 | 0.031 | 0.703 | 0.002 | | 40.315 |  |
|  |  | rs957755 | T | G | 0.140 | -0.064 | 0.014 | 7.42E-06 | 0.031 | 0.030 | 0.314 | 0.001 | | 18.189 |  |
|  |  | rs9903102 | C | A | 0.198 | -0.069 | 0.015 | 4.92E-06 | -0.023 | 0.027 | 0.383 | 0.002 | | 28.056 |  |
| ebi-a-GCST90016956 | Gut microbiota abundance (family Veillonellaceae id.2172) | rs111810795 | C | T | 0.098 | -0.087 | 0.018 | 1.73E-06 | 0.025 | 0.035 | 0.476 | 0.001 | | 24.371 |  |
|  |  | rs114889439 | A | G | 0.035 | -0.254 | 0.054 | 6.19E-06 | -0.124 | 0.058 | 0.033 | 0.004 | | 79.060 |  |
|  |  | rs11700976 | C | A | 0.493 | 0.050 | 0.011 | 9.25E-06 | -0.031 | 0.021 | 0.150 | 0.001 | | 23.255 |  |
|  |  | rs12186441 | G | A | 0.042 | 0.208 | 0.045 | 4.53E-06 | -0.025 | 0.053 | 0.639 | 0.003 | | 64.215 |  |
|  |  | rs12668619 | A | G | 0.329 | 0.055 | 0.012 | 2.57E-06 | -0.006 | 0.023 | 0.798 | 0.001 | | 24.779 |  |
|  |  | rs12741784 | C | T | 0.258 | -0.062 | 0.012 | 1.28E-07 | 0.007 | 0.024 | 0.785 | 0.001 | | 27.128 |  |
|  |  | rs1442060 | A | G | 0.458 | 0.051 | 0.011 | 4.51E-06 | -0.001 | 0.021 | 0.982 | 0.001 | | 24.089 |  |
|  |  | rs1693340 | T | C | 0.072 | 0.082 | 0.018 | 9.25E-06 | -0.004 | 0.041 | 0.931 | 0.001 | | 16.479 |  |
|  |  | rs2175069 | G | A | 0.627 | 0.053 | 0.011 | 4.64E-06 | 0.005 | 0.022 | 0.835 | 0.001 | | 23.828 |  |
|  |  | rs2561116 | T | G | 0.058 | -0.084 | 0.019 | 7.89E-06 | 0.032 | 0.045 | 0.477 | 0.001 | | 14.094 |  |
|  |  | rs2585520 | G | T | 0.038 | -0.090 | 0.020 | 5.27E-06 | -0.029 | 0.055 | 0.596 | 0.001 | | 11.073 |  |
|  |  | rs4263802 | A | G | 0.672 | -0.051 | 0.011 | 7.45E-06 | 0.009 | 0.023 | 0.691 | 0.001 | | 21.019 |  |
|  |  | rs4461038 | G | A | 0.709 | 0.055 | 0.012 | 3.73E-06 | -0.066 | 0.023 | 0.004 | 0.001 | | 23.344 |  |
|  |  | rs4797169 | T | C | 0.237 | 0.059 | 0.013 | 4.49E-06 | -0.008 | 0.025 | 0.761 | 0.001 | | 22.930 |  |
|  |  | rs61264131 | A | C | 0.097 | 0.202 | 0.046 | 6.75E-06 | 0.001 | 0.036 | 0.987 | 0.007 | | 132.338 |  |
|  |  | rs6692542 | G | A | 0.635 | -0.053 | 0.012 | 8.68E-06 | -0.006 | 0.022 | 0.795 | 0.001 | | 24.317 |  |
|  |  | rs6909981 | C | T | 0.122 | -0.064 | 0.014 | 5.48E-06 | 0.001 | 0.032 | 0.975 | 0.001 | | 16.017 |  |
|  |  | rs79535861 | A | C | 0.091 | 0.101 | 0.021 | 1.58E-06 | -0.051 | 0.037 | 0.167 | 0.002 | | 30.935 |  |
|  |  | rs9345168 | A | C | 0.558 | -0.051 | 0.011 | 8.49E-06 | -0.024 | 0.021 | 0.259 | 0.001 | | 23.474 |  |
| ebi-a-GCST90016957 | Gut microbiota abundance (family Verrucomicrobiaceae id.4036) | rs111862613 | T | C | 0.178 | 0.091 | 0.020 | 3.73E-06 | 0.013 | 0.029 | 0.662 | 0.002 | | 44.220 |  |
|  |  | rs117107102 | A | G | 0.048 | 0.205 | 0.043 | 2.92E-06 | 0.026 | 0.049 | 0.603 | 0.004 | | 69.938 |  |
|  |  | rs11729256 | T | C | 0.170 | 0.075 | 0.015 | 6.73E-07 | 0.004 | 0.028 | 0.875 | 0.002 | | 29.099 |  |
|  |  | rs12908520 | G | A | 0.426 | 0.062 | 0.013 | 2.15E-06 | 0.017 | 0.021 | 0.429 | 0.002 | | 34.445 |  |
|  |  | rs2602429 | T | C | 0.739 | -0.075 | 0.016 | 2.70E-06 | -0.015 | 0.024 | 0.532 | 0.002 | | 39.441 |  |
|  |  | rs4242783 | A | G | 0.717 | -0.069 | 0.015 | 2.75E-06 | 0.017 | 0.024 | 0.471 | 0.002 | | 35.323 |  |
|  |  | rs4936098 | G | A | 0.651 | -0.065 | 0.014 | 1.13E-06 | 0.005 | 0.022 | 0.832 | 0.002 | | 35.127 |  |
|  |  | rs61779207 | G | A | 0.224 | -0.076 | 0.017 | 6.63E-06 | -0.010 | 0.026 | 0.707 | 0.002 | | 36.723 |  |
|  |  | rs74542928 | T | C | 0.048 | 0.112 | 0.024 | 1.65E-06 | -0.023 | 0.049 | 0.640 | 0.001 | | 21.069 |  |
|  |  | rs9349825 | A | G | 0.190 | -0.070 | 0.015 | 2.51E-06 | 0.044 | 0.027 | 0.103 | 0.002 | | 28.000 |  |
|  |  | rs941682 | G | A | 0.274 | -0.063 | 0.014 | 9.58E-06 | 0.028 | 0.024 | 0.237 | 0.002 | | 29.124 |  |
| ebi-a-GCST90016958 | Gut microbiota abundance (family Victivallaceae id.2255) | rs11671100 | A | C | 0.205 | -0.160 | 0.035 | 4.08E-06 | 0.006 | 0.027 | 0.822 | 0.008 | | 154.597 |  |
|  |  | rs11764871 | G | T | 0.305 | 0.127 | 0.026 | 7.49E-07 | -0.037 | 0.023 | 0.110 | 0.007 | | 126.384 |  |
|  |  | rs2944282 | T | C | 0.279 | -0.124 | 0.026 | 1.57E-06 | 0.007 | 0.024 | 0.767 | 0.006 | | 114.562 |  |
|  |  | rs34962571 | A | C | 0.101 | -0.187 | 0.042 | 6.25E-06 | -0.023 | 0.035 | 0.507 | 0.006 | | 117.230 |  |
|  |  | rs4396289 | C | T | 0.129 | -0.153 | 0.029 | 1.54E-07 | -0.002 | 0.031 | 0.946 | 0.005 | | 96.614 |  |
|  |  | rs61702987 | T | C | 0.109 | 0.146 | 0.030 | 3.08E-06 | -0.009 | 0.034 | 0.791 | 0.004 | | 75.781 |  |
|  |  | rs62570196 | C | T | 0.042 | -0.246 | 0.048 | 2.70E-07 | 0.023 | 0.053 | 0.658 | 0.005 | | 89.753 |  |
|  |  | rs6545794 | A | G | 0.120 | -0.198 | 0.041 | 5.97E-07 | 0.018 | 0.033 | 0.580 | 0.008 | | 152.660 |  |
|  |  | rs67832247 | C | T | 0.337 | -0.123 | 0.027 | 3.24E-06 | -0.001 | 0.023 | 0.970 | 0.007 | | 125.876 |  |
|  |  | rs7077363 | G | A | 0.163 | 0.149 | 0.032 | 2.83E-06 | -0.008 | 0.029 | 0.781 | 0.006 | | 111.567 |  |
|  |  | rs7314815 | G | A | 0.552 | 0.101 | 0.023 | 6.40E-06 | 0.037 | 0.021 | 0.086 | 0.005 | | 93.169 |  |
|  |  | rs7627405 | C | T | 0.202 | -0.134 | 0.030 | 8.19E-06 | -0.026 | 0.027 | 0.325 | 0.006 | | 107.068 |  |
| ebi-a-GCST90017090 | Gut microbiota abundance (order Actinomycetales id.420) | rs2889192 | T | G | 0.851 | -0.088 | 0.019 | 3.97E-06 | 0.005 | 0.030 | 0.879 | 0.002 | | 36.418 |  |
|  |  | rs34583783 | G | T | 0.061 | 0.124 | 0.026 | 5.54E-06 | -0.032 | 0.044 | 0.471 | 0.002 | | 32.037 |  |
|  |  | rs35011108 | A | G | 0.068 | 0.242 | 0.050 | 1.88E-06 | -0.038 | 0.043 | 0.371 | 0.007 | | 136.156 |  |
|  |  | rs4073240 | G | A | 0.381 | 0.075 | 0.016 | 5.68E-06 | 0.011 | 0.022 | 0.617 | 0.003 | | 48.734 |  |
|  |  | rs58484246 | T | C | 0.366 | 0.076 | 0.017 | 6.67E-06 | 0.009 | 0.022 | 0.700 | 0.003 | | 49.048 |  |
| ebi-a-GCST90017091 | Gut microbiota abundance (order Bacillales id.1674) | rs10233278 | T | C | 0.555 | -0.116 | 0.025 | 3.51E-06 | -0.018 | 0.021 | 0.406 | 0.007 | | 123.359 |  |
|  |  | rs10410917 | C | T | 0.389 | -0.115 | 0.025 | 5.57E-06 | 0.022 | 0.022 | 0.307 | 0.006 | | 115.468 |  |
|  |  | rs11034576 | A | G | 0.124 | 0.206 | 0.045 | 8.86E-06 | -0.027 | 0.032 | 0.397 | 0.009 | | 169.884 |  |
|  |  | rs11207728 | A | G | 0.837 | 0.145 | 0.032 | 5.73E-06 | 0.023 | 0.029 | 0.415 | 0.006 | | 105.151 |  |
|  |  | rs11844714 | A | G | 0.208 | -0.143 | 0.032 | 5.06E-06 | -0.052 | 0.026 | 0.048 | 0.007 | | 124.728 |  |
|  |  | rs1287018 | G | A | 0.186 | 0.141 | 0.032 | 9.87E-06 | 0.008 | 0.027 | 0.784 | 0.006 | | 111.310 |  |
|  |  | rs4617108 | G | A | 0.914 | -0.249 | 0.053 | 1.98E-06 | -0.038 | 0.038 | 0.322 | 0.010 | | 179.914 |  |
|  |  | rs62640857 | A | G | 0.226 | 0.148 | 0.033 | 4.49E-06 | -0.022 | 0.025 | 0.390 | 0.008 | | 142.160 |  |
|  |  | rs74420793 | A | G | 0.104 | -0.164 | 0.035 | 3.07E-06 | 0.037 | 0.034 | 0.276 | 0.005 | | 93.089 |  |
| ebi-a-GCST90017092 | Gut microbiota abundance (order Bacteroidales id.913) | rs11146701 | A | G | 0.343 | 0.047 | 0.011 | 7.08E-06 | -0.034 | 0.028 | 0.232 | 0.001 | | 18.567 |  |
|  |  | rs17343978 | A | C | 0.204 | -0.055 | 0.012 | 8.36E-06 | 0.018 | 0.026 | 0.482 | 0.001 | | 18.189 |  |
|  |  | rs2032750 | C | T | 0.539 | 0.051 | 0.011 | 1.92E-06 | 0.010 | 0.021 | 0.634 | 0.001 | | 23.587 |  |
|  |  | rs2363574 | T | C | 0.962 | 0.223 | 0.051 | 9.93E-06 | -0.065 | 0.056 | 0.248 | 0.004 | | 66.930 |  |
|  |  | rs4146051 | G | A | 0.917 | 0.107 | 0.025 | 8.76E-06 | 0.000 | 0.039 | 0.995 | 0.002 | | 32.206 |  |
|  |  | rs4916508 | A | G | 0.576 | 0.047 | 0.011 | 8.47E-06 | -0.003 | 0.021 | 0.903 | 0.001 | | 19.528 |  |
|  |  | rs55773148 | G | A | 0.053 | -0.122 | 0.024 | 3.90E-07 | -0.012 | 0.047 | 0.792 | 0.001 | | 27.240 |  |
|  |  | rs62531359 | T | G | 0.184 | 0.066 | 0.015 | 9.09E-06 | 0.013 | 0.028 | 0.631 | 0.001 | | 23.708 |  |
|  |  | rs62575403 | C | T | 0.042 | 0.140 | 0.031 | 7.06E-06 | 0.030 | 0.053 | 0.569 | 0.002 | | 29.171 |  |
|  |  | rs72706335 | T | C | 0.026 | -0.222 | 0.049 | 7.66E-06 | -0.055 | 0.067 | 0.415 | 0.002 | | 45.211 |  |
|  |  | rs73975615 | G | A | 0.008 | -0.207 | 0.044 | 1.22E-06 | -0.166 | 0.124 | 0.180 | 0.001 | | 11.723 |  |
|  |  | rs7631304 | G | A | 0.149 | -0.065 | 0.013 | 8.37E-07 | -0.019 | 0.030 | 0.523 | 0.001 | | 19.371 |  |
|  |  | rs79585701 | A | C | 0.142 | 0.065 | 0.015 | 9.99E-06 | 0.020 | 0.030 | 0.521 | 0.001 | | 18.670 |  |
|  |  | rs929878 | T | C | 0.787 | 0.055 | 0.012 | 4.73E-06 | -0.008 | 0.026 | 0.769 | 0.001 | | 18.530 |  |
| ebi-a-GCST90017093 | Gut microbiota abundance (order Bifidobacteriales id.432) | rs10831953 | G | A | 0.300 | 0.054 | 0.012 | 9.95E-06 | 0.053 | 0.023 | 0.020 | 0.001 | | 22.277 |  |
|  |  | rs12446429 | T | C | 0.195 | 0.081 | 0.019 | 8.53E-06 | -0.007 | 0.028 | 0.810 | 0.002 | | 37.875 |  |
|  |  | rs13020688 | G | A | 0.305 | 0.058 | 0.012 | 1.57E-06 | -0.001 | 0.023 | 0.962 | 0.001 | | 26.567 |  |
|  |  | rs182549 | T | C | 0.598 | -0.117 | 0.013 | 5.94E-20 | -0.048 | 0.022 | 0.026 | 0.007 | | 121.688 |  |
|  |  | rs4957061 | T | C | 0.423 | 0.057 | 0.012 | 1.15E-06 | -0.006 | 0.022 | 0.787 | 0.002 | | 29.109 |  |
|  |  | rs540489 | T | G | 0.176 | -0.063 | 0.014 | 5.37E-06 | -0.015 | 0.028 | 0.592 | 0.001 | | 21.305 |  |
|  |  | rs55888705 | A | G | 0.282 | 0.054 | 0.012 | 8.66E-06 | -0.020 | 0.023 | 0.390 | 0.001 | | 21.404 |  |
|  |  | rs6899771 | A | G | 0.101 | -0.091 | 0.020 | 7.28E-06 | 0.000 | 0.035 | 0.992 | 0.002 | | 27.783 |  |
|  |  | rs7174549 | T | C | 0.641 | -0.055 | 0.012 | 6.87E-06 | 0.011 | 0.022 | 0.619 | 0.001 | | 25.714 |  |
|  |  | rs7322849 | T | C | 0.092 | 0.111 | 0.020 | 1.74E-08 | 0.055 | 0.037 | 0.140 | 0.002 | | 37.664 |  |
|  |  | rs73797465 | T | G | 0.115 | -0.094 | 0.021 | 4.85E-06 | -0.022 | 0.034 | 0.516 | 0.002 | | 33.226 |  |
|  |  | rs857444 | C | T | 0.369 | 0.055 | 0.012 | 3.82E-06 | -0.035 | 0.022 | 0.108 | 0.001 | | 26.232 |  |
| ebi-a-GCST90017094 | Gut microbiota abundance (order Burkholderiales id.2874) | rs1511453 | A | G | 0.058 | 0.091 | 0.020 | 8.00E-06 | 0.038 | 0.045 | 0.406 | 0.001 | | 16.688 |  |
|  |  | rs1928341 | G | A | 0.595 | -0.051 | 0.011 | 4.52E-06 | -0.022 | 0.022 | 0.318 | 0.001 | | 22.813 |  |
|  |  | rs2321387 | G | A | 0.431 | -0.051 | 0.011 | 3.26E-06 | 0.016 | 0.021 | 0.452 | 0.001 | | 23.281 |  |
|  |  | rs2613606 | T | C | 0.582 | 0.050 | 0.011 | 4.13E-06 | 0.012 | 0.021 | 0.590 | 0.001 | | 22.301 |  |
|  |  | rs4033856 | T | C | 0.914 | -0.083 | 0.017 | 5.67E-07 | 0.005 | 0.038 | 0.896 | 0.001 | | 20.111 |  |
|  |  | rs6087811 | T | G | 0.102 | -0.102 | 0.020 | 2.88E-07 | 0.049 | 0.035 | 0.165 | 0.002 | | 34.583 |  |
|  |  | rs62191117 | A | G | 0.205 | 0.068 | 0.013 | 2.79E-07 | 0.003 | 0.026 | 0.923 | 0.002 | | 27.680 |  |
|  |  | rs62395635 | T | C | 0.062 | 0.110 | 0.024 | 2.90E-06 | 0.044 | 0.044 | 0.317 | 0.001 | | 25.912 |  |
|  |  | rs75242906 | C | T | 0.084 | -0.121 | 0.028 | 9.75E-06 | -0.092 | 0.038 | 0.017 | 0.002 | | 41.276 |  |
|  |  | rs7638039 | T | C | 0.247 | 0.058 | 0.013 | 4.84E-06 | -0.012 | 0.025 | 0.635 | 0.001 | | 23.054 |  |
| ebi-a-GCST90017095 | Gut microbiota abundance (order Clostridiales id.1863) | rs10774377 | G | A | 0.564 | -0.052 | 0.011 | 3.81E-06 | 0.005 | 0.021 | 0.806 | 0.001 | | 24.701 |  |
|  |  | rs112334273 | G | A | 0.278 | 0.064 | 0.013 | 4.07E-07 | -0.030 | 0.024 | 0.203 | 0.002 | | 30.144 |  |
|  |  | rs13105690 | C | T | 0.715 | 0.053 | 0.012 | 9.37E-06 | 0.010 | 0.024 | 0.683 | 0.001 | | 20.832 |  |
|  |  | rs13179700 | C | T | 0.656 | -0.051 | 0.011 | 3.52E-06 | 0.007 | 0.022 | 0.767 | 0.001 | | 21.627 |  |
|  |  | rs1842454 | G | A | 0.188 | -0.054 | 0.013 | 9.92E-06 | 0.021 | 0.027 | 0.440 | 0.001 | | 16.537 |  |
|  |  | rs2273429 | A | G | 0.105 | -0.073 | 0.015 | 4.17E-06 | 0.053 | 0.035 | 0.128 | 0.001 | | 18.117 |  |
|  |  | rs290772 | G | A | 0.054 | 0.084 | 0.020 | 1.00E-05 | -0.013 | 0.047 | 0.785 | 0.001 | | 13.244 |  |
|  |  | rs6442336 | T | C | 0.748 | 0.055 | 0.012 | 9.63E-06 | -0.045 | 0.024 | 0.066 | 0.001 | | 20.764 |  |
|  |  | rs6814436 | C | T | 0.859 | -0.074 | 0.015 | 9.06E-07 | 0.004 | 0.030 | 0.907 | 0.001 | | 24.451 |  |
|  |  | rs6815608 | C | T | 0.849 | -0.104 | 0.021 | 3.72E-07 | 0.006 | 0.030 | 0.847 | 0.003 | | 51.223 |  |
|  |  | rs72738886 | T | C | 0.076 | 0.087 | 0.019 | 8.42E-06 | 0.006 | 0.040 | 0.882 | 0.001 | | 19.336 |  |
|  |  | rs72915163 | T | C | 0.219 | -0.058 | 0.012 | 1.39E-06 | 0.060 | 0.026 | 0.020 | 0.001 | | 21.138 |  |
|  |  | rs992074 | T | C | 0.977 | -0.255 | 0.051 | 8.95E-07 | -0.063 | 0.071 | 0.371 | 0.003 | | 53.934 |  |
| ebi-a-GCST90017096 | Gut microbiota abundance (order Coriobacteriales id.810) | rs11250875 | T | C | 0.214 | 0.061 | 0.013 | 4.83E-06 | 0.021 | 0.026 | 0.422 | 0.001 | | 22.780 |  |
|  |  | rs11656361 | A | C | 0.182 | 0.077 | 0.018 | 8.02E-06 | 0.005 | 0.028 | 0.857 | 0.002 | | 32.685 |  |
|  |  | rs12974142 | G | A | 0.070 | 0.079 | 0.018 | 8.51E-06 | 0.024 | 0.041 | 0.570 | 0.001 | | 14.974 |  |
|  |  | rs13307134 | T | C | 0.833 | -0.057 | 0.013 | 7.80E-06 | -0.049 | 0.028 | 0.083 | 0.001 | | 16.310 |  |
|  |  | rs1397793 | A | G | 0.690 | 0.050 | 0.011 | 9.77E-06 | -0.035 | 0.023 | 0.134 | 0.001 | | 19.533 |  |
|  |  | rs1816223 | G | A | 0.799 | 0.059 | 0.013 | 4.84E-06 | -0.071 | 0.026 | 0.007 | 0.001 | | 20.244 |  |
|  |  | rs240104 | T | C | 0.285 | -0.060 | 0.013 | 1.52E-06 | -0.007 | 0.024 | 0.782 | 0.001 | | 27.237 |  |
|  |  | rs2442778 | A | G | 0.948 | 0.116 | 0.026 | 9.03E-06 | -0.104 | 0.048 | 0.029 | 0.001 | | 24.398 |  |
|  |  | rs3025411 | A | G | 0.107 | 0.093 | 0.021 | 8.27E-06 | -0.014 | 0.035 | 0.697 | 0.002 | | 30.035 |  |
|  |  | rs34739816 | G | T | 0.058 | 0.097 | 0.021 | 3.88E-06 | 0.075 | 0.045 | 0.096 | 0.001 | | 18.737 |  |
|  |  | rs67561917 | A | G | 0.183 | -0.071 | 0.015 | 5.39E-06 | -0.020 | 0.028 | 0.471 | 0.002 | | 27.966 |  |
|  |  | rs719099 | A | G | 0.099 | 0.078 | 0.016 | 5.43E-07 | 0.051 | 0.036 | 0.148 | 0.001 | | 19.822 |  |
|  |  | rs8010111 | A | G | 0.919 | 0.103 | 0.023 | 6.90E-06 | 0.010 | 0.038 | 0.795 | 0.002 | | 29.124 |  |
| ebi-a-GCST90017097 | Gut microbiota abundance (order Desulfovibrionales id.3156) | rs112381107 | C | T | 0.060 | 0.210 | 0.046 | 3.22E-06 | -0.061 | 0.045 | 0.170 | 0.005 | | 91.714 |  |
|  |  | rs11599763 | C | T | 0.592 | 0.055 | 0.012 | 2.61E-06 | 0.007 | 0.022 | 0.733 | 0.001 | | 27.305 |  |
|  |  | rs17791387 | A | G | 0.095 | -0.073 | 0.015 | 2.25E-06 | 0.056 | 0.036 | 0.123 | 0.001 | | 16.759 |  |
|  |  | rs186073 | T | C | 0.394 | 0.053 | 0.012 | 8.74E-06 | -0.010 | 0.022 | 0.632 | 0.001 | | 24.567 |  |
|  |  | rs2692012 | G | A | 0.945 | -0.112 | 0.025 | 2.27E-06 | 0.069 | 0.047 | 0.138 | 0.001 | | 24.130 |  |
|  |  | rs2838334 | G | A | 0.344 | 0.057 | 0.012 | 4.17E-06 | 0.000 | 0.022 | 0.999 | 0.001 | | 26.834 |  |
|  |  | rs3935584 | C | T | 0.532 | -0.052 | 0.012 | 7.20E-06 | -0.004 | 0.021 | 0.857 | 0.001 | | 25.153 |  |
|  |  | rs4506934 | C | T | 0.118 | -0.095 | 0.020 | 2.43E-06 | -0.034 | 0.033 | 0.304 | 0.002 | | 34.812 |  |
|  |  | rs6058181 | C | T | 0.162 | 0.084 | 0.017 | 2.53E-07 | -0.032 | 0.029 | 0.273 | 0.002 | | 34.911 |  |
|  |  | rs62020470 | A | G | 0.180 | -0.057 | 0.013 | 7.51E-06 | -0.017 | 0.028 | 0.538 | 0.001 | | 17.799 |  |
|  |  | rs72647048 | T | C | 0.112 | -0.077 | 0.017 | 9.00E-06 | -0.013 | 0.033 | 0.701 | 0.001 | | 21.825 |  |
|  |  | rs9928243 | C | A | 0.471 | -0.054 | 0.012 | 3.97E-06 | 0.044 | 0.021 | 0.037 | 0.001 | | 27.155 |  |
| ebi-a-GCST90017098 | Gut microbiota abundance (order Enterobacteriales id.3468) | rs11026530 | T | C | 0.150 | 0.082 | 0.019 | 9.43E-06 | 0.041 | 0.030 | 0.172 | 0.002 | | 31.665 |  |
|  |  | rs2374342 | C | A | 0.415 | 0.058 | 0.013 | 4.52E-06 | 0.024 | 0.022 | 0.256 | 0.002 | | 30.300 |  |
|  |  | rs35673018 | G | A | 0.090 | 0.090 | 0.020 | 7.63E-06 | -0.042 | 0.037 | 0.250 | 0.001 | | 24.373 |  |
|  |  | rs504442 | T | G | 0.105 | 0.084 | 0.019 | 5.17E-06 | 0.049 | 0.035 | 0.158 | 0.001 | | 24.424 |  |
|  |  | rs62210023 | A | G | 0.345 | 0.061 | 0.013 | 3.13E-06 | 0.033 | 0.022 | 0.143 | 0.002 | | 30.563 |  |
|  |  | rs78143293 | A | G | 0.122 | -0.085 | 0.017 | 1.20E-06 | 0.078 | 0.032 | 0.016 | 0.002 | | 28.369 |  |
|  |  | rs79757635 | C | A | 0.137 | 0.076 | 0.017 | 9.32E-06 | -0.049 | 0.031 | 0.117 | 0.001 | | 24.957 |  |
| ebi-a-GCST90017099 | Gut microbiota abundance (order Erysipelotrichales id.2148) | rs1074800 | G | A | 0.581 | -0.049 | 0.011 | 6.15E-06 | 0.009 | 0.022 | 0.673 | 0.001 | | 21.674 |  |
|  |  | rs10781552 | C | T | 0.281 | -0.055 | 0.012 | 2.33E-06 | 0.029 | 0.024 | 0.220 | 0.001 | | 22.609 |  |
|  |  | rs17530232 | A | G | 0.053 | 0.103 | 0.022 | 2.79E-06 | 0.007 | 0.047 | 0.880 | 0.001 | | 19.513 |  |
|  |  | rs1884466 | C | T | 0.520 | -0.048 | 0.011 | 9.53E-06 | -0.009 | 0.021 | 0.684 | 0.001 | | 20.710 |  |
|  |  | rs2300774 | A | G | 0.561 | -0.052 | 0.011 | 8.95E-07 | 0.015 | 0.021 | 0.495 | 0.001 | | 24.848 |  |
|  |  | rs290833 | T | G | 0.493 | -0.050 | 0.011 | 8.03E-06 | 0.029 | 0.021 | 0.178 | 0.001 | | 22.706 |  |
|  |  | rs35161940 | T | C | 0.107 | -0.081 | 0.017 | 1.85E-06 | 0.031 | 0.034 | 0.361 | 0.001 | | 22.815 |  |
|  |  | rs4078432 | T | C | 0.827 | 0.061 | 0.013 | 4.23E-06 | -0.030 | 0.028 | 0.293 | 0.001 | | 19.477 |  |
|  |  | rs56970041 | T | G | 0.061 | 0.072 | 0.016 | 5.40E-06 | 0.017 | 0.044 | 0.700 | 0.001 | | 11.067 |  |
|  |  | rs62504403 | C | T | 0.195 | 0.068 | 0.013 | 1.12E-07 | -0.002 | 0.027 | 0.933 | 0.001 | | 26.782 |  |
|  |  | rs7234058 | T | C | 0.090 | -0.095 | 0.019 | 9.12E-07 | -0.019 | 0.037 | 0.607 | 0.001 | | 27.034 |  |
|  |  | rs7826267 | G | T | 0.931 | 0.084 | 0.020 | 9.28E-06 | 0.035 | 0.043 | 0.416 | 0.001 | | 16.669 |  |
|  |  | rs8003149 | C | T | 0.333 | 0.054 | 0.012 | 4.08E-06 | 0.012 | 0.022 | 0.591 | 0.001 | | 23.661 |  |
| ebi-a-GCST90017100 | Gut microbiota abundance (order Gastranaerophilales id.1591) | rs11150282 | T | C | 0.360 | 0.098 | 0.020 | 7.36E-07 | -0.007 | 0.022 | 0.750 | 0.004 | | 81.939 |  |
|  |  | rs113884518 | T | C | 0.024 | -0.206 | 0.046 | 7.74E-06 | 0.019 | 0.068 | 0.784 | 0.002 | | 36.720 |  |
|  |  | rs28678345 | T | C | 0.049 | 0.213 | 0.047 | 8.06E-06 | -0.015 | 0.049 | 0.760 | 0.004 | | 77.944 |  |
|  |  | rs367480 | A | G | 0.642 | 0.084 | 0.019 | 7.52E-06 | -0.037 | 0.022 | 0.090 | 0.003 | | 59.944 |  |
|  |  | rs4129395 | G | A | 0.485 | 0.090 | 0.019 | 1.22E-06 | -0.033 | 0.021 | 0.121 | 0.004 | | 75.212 |  |
|  |  | rs789069 | A | C | 0.141 | -0.104 | 0.023 | 6.50E-06 | 0.002 | 0.030 | 0.959 | 0.003 | | 48.322 |  |
|  |  | rs79790072 | T | C | 0.032 | 0.226 | 0.049 | 3.54E-06 | 0.065 | 0.061 | 0.283 | 0.003 | | 57.415 |  |
|  |  | rs8028558 | A | G | 0.358 | 0.083 | 0.019 | 9.78E-06 | 0.008 | 0.022 | 0.706 | 0.003 | | 58.919 |  |
|  |  | rs9864379 | T | C | 0.150 | -0.161 | 0.029 | 4.66E-08 | -0.008 | 0.030 | 0.801 | 0.007 | | 121.087 |  |
| ebi-a-GCST90017101 | Gut microbiota abundance (order Lactobacillales id.1800) | rs11110282 | A | G | 0.048 | -0.102 | 0.022 | 3.96E-06 | 0.004 | 0.049 | 0.931 | 0.001 | | 17.625 |  |
|  |  | rs11627423 | C | A | 0.367 | 0.050 | 0.011 | 5.09E-06 | -0.001 | 0.022 | 0.951 | 0.001 | | 21.260 |  |
|  |  | rs11730038 | G | A | 0.294 | -0.061 | 0.013 | 5.10E-06 | -0.010 | 0.023 | 0.660 | 0.002 | | 27.914 |  |
|  |  | rs12797734 | T | C | 0.262 | 0.057 | 0.013 | 7.77E-06 | 0.026 | 0.025 | 0.295 | 0.001 | | 23.173 |  |
|  |  | rs1595463 | C | A | 0.539 | 0.048 | 0.011 | 7.44E-06 | -0.006 | 0.021 | 0.780 | 0.001 | | 20.998 |  |
|  |  | rs2370083 | G | T | 0.065 | -0.081 | 0.018 | 8.33E-06 | -0.020 | 0.043 | 0.642 | 0.001 | | 14.375 |  |
|  |  | rs2952251 | G | A | 0.771 | 0.063 | 0.012 | 3.36E-07 | 0.016 | 0.027 | 0.548 | 0.001 | | 25.658 |  |
|  |  | rs34989881 | A | G | 0.049 | 0.113 | 0.025 | 4.09E-06 | -0.061 | 0.050 | 0.216 | 0.001 | | 21.855 |  |
|  |  | rs35344081 | G | A | 0.263 | 0.064 | 0.013 | 4.16E-07 | 0.008 | 0.024 | 0.725 | 0.002 | | 29.352 |  |
|  |  | rs4028634 | C | T | 0.635 | -0.053 | 0.011 | 1.35E-06 | -0.001 | 0.022 | 0.956 | 0.001 | | 24.176 |  |
|  |  | rs57872228 | C | T | 0.117 | -0.069 | 0.015 | 2.58E-06 | -0.030 | 0.033 | 0.362 | 0.001 | | 17.921 |  |
|  |  | rs74663707 | C | T | 0.066 | 0.098 | 0.022 | 8.40E-06 | 0.019 | 0.043 | 0.655 | 0.001 | | 21.882 |  |
|  |  | rs77558518 | A | G | 0.096 | -0.106 | 0.022 | 1.67E-06 | -0.015 | 0.036 | 0.674 | 0.002 | | 36.061 |  |
|  |  | rs78938557 | T | C | 0.030 | 0.106 | 0.023 | 2.31E-06 | 0.031 | 0.063 | 0.625 | 0.001 | | 11.917 |  |
|  |  | rs9581006 | T | C | 0.963 | -0.226 | 0.047 | 1.77E-06 | 0.084 | 0.056 | 0.134 | 0.004 | | 66.365 |  |
| ebi-a-GCST90017102 | Gut microbiota abundance (order Methanobacteriales id.120) | rs10202904 | G | T | 0.592 | 0.122 | 0.024 | 3.01E-07 | -0.024 | 0.022 | 0.263 | 0.007 | | 132.297 |  |
|  |  | rs10424197 | A | G | 0.743 | 0.111 | 0.025 | 9.28E-06 | -0.016 | 0.024 | 0.501 | 0.005 | | 87.104 |  |
|  |  | rs4257531 | G | A | 0.106 | 0.164 | 0.036 | 7.44E-06 | -0.024 | 0.035 | 0.487 | 0.005 | | 94.137 |  |
|  |  | rs56131665 | G | A | 0.107 | 0.179 | 0.039 | 6.18E-06 | 0.020 | 0.034 | 0.564 | 0.006 | | 112.919 |  |
|  |  | rs6508769 | C | T | 0.853 | -0.154 | 0.034 | 8.23E-06 | 0.011 | 0.030 | 0.704 | 0.006 | | 108.783 |  |
|  |  | rs6776814 | T | C | 0.020 | -0.200 | 0.041 | 1.63E-06 | 0.017 | 0.074 | 0.819 | 0.002 | | 29.335 |  |
|  |  | rs73068003 | G | T | 0.096 | -0.158 | 0.035 | 8.45E-06 | 0.052 | 0.036 | 0.144 | 0.004 | | 80.073 |  |
|  |  | rs73457410 | A | G | 0.064 | 0.215 | 0.044 | 1.41E-06 | 0.010 | 0.043 | 0.809 | 0.006 | | 101.879 |  |
|  |  | rs75208022 | C | T | 0.093 | -0.227 | 0.049 | 5.92E-06 | -0.018 | 0.036 | 0.615 | 0.009 | | 161.898 |  |
|  |  | rs894996 | C | A | 0.070 | 0.217 | 0.045 | 1.88E-06 | 0.028 | 0.041 | 0.492 | 0.006 | | 113.062 |  |
| ebi-a-GCST90017103 | Gut microbiota abundance (order Mollicutes RF9 id.11579) | rs11779863 | G | A | 0.160 | -0.077 | 0.017 | 6.69E-06 | 0.042 | 0.029 | 0.154 | 0.002 | | 29.424 |  |
|  |  | rs12566890 | T | G | 0.132 | -0.103 | 0.024 | 8.11E-06 | -0.007 | 0.032 | 0.825 | 0.002 | | 44.749 |  |
|  |  | rs13100746 | C | T | 0.444 | 0.064 | 0.014 | 7.29E-06 | -0.013 | 0.021 | 0.552 | 0.002 | | 37.040 |  |
|  |  | rs17235252 | T | C | 0.119 | -0.122 | 0.026 | 2.16E-06 | -0.019 | 0.033 | 0.567 | 0.003 | | 57.579 |  |
|  |  | rs3932485 | C | T | 0.391 | 0.063 | 0.014 | 9.93E-06 | -0.024 | 0.022 | 0.269 | 0.002 | | 34.307 |  |
|  |  | rs515984 | C | T | 0.886 | 0.088 | 0.019 | 6.61E-06 | 0.047 | 0.033 | 0.155 | 0.002 | | 28.457 |  |
|  |  | rs638542 | A | G | 0.712 | 0.071 | 0.016 | 5.17E-06 | 0.021 | 0.023 | 0.370 | 0.002 | | 37.530 |  |
|  |  | rs74603314 | T | C | 0.040 | 0.231 | 0.049 | 2.28E-06 | -0.007 | 0.054 | 0.898 | 0.004 | | 75.525 |  |
|  |  | rs76373661 | G | A | 0.159 | 0.091 | 0.020 | 5.16E-06 | -0.024 | 0.029 | 0.419 | 0.002 | | 40.439 |  |
|  |  | rs7706512 | A | G | 0.479 | -0.066 | 0.014 | 2.27E-06 | 0.006 | 0.021 | 0.794 | 0.002 | | 39.633 |  |
|  |  | rs7801843 | A | G | 0.152 | -0.087 | 0.019 | 9.47E-06 | -0.023 | 0.030 | 0.440 | 0.002 | | 35.868 |  |
|  |  | rs7853673 | A | G | 0.538 | 0.062 | 0.014 | 6.73E-06 | 0.003 | 0.021 | 0.899 | 0.002 | | 35.612 |  |
|  |  | rs949341 | A | G | 0.721 | -0.066 | 0.015 | 7.73E-06 | -0.008 | 0.024 | 0.745 | 0.002 | | 31.841 |  |
| ebi-a-GCST90017104 | Gut microbiota abundance (order NB1n id.3953) | rs11251024 | G | A | 0.264 | 0.104 | 0.021 | 6.63E-07 | -0.010 | 0.024 | 0.684 | 0.004 | | 77.780 |  |
|  |  | rs11606187 | A | G | 0.145 | -0.155 | 0.033 | 3.31E-06 | 0.013 | 0.030 | 0.670 | 0.006 | | 108.986 |  |
|  |  | rs13385922 | T | C | 0.347 | 0.093 | 0.020 | 3.97E-06 | -0.053 | 0.022 | 0.017 | 0.004 | | 72.091 |  |
|  |  | rs166849 | A | G | 0.586 | -0.091 | 0.020 | 7.74E-06 | 0.028 | 0.021 | 0.187 | 0.004 | | 74.146 |  |
|  |  | rs2172426 | T | C | 0.596 | 0.102 | 0.020 | 3.17E-07 | -0.022 | 0.022 | 0.312 | 0.005 | | 92.551 |  |
|  |  | rs267959 | G | A | 0.723 | -0.099 | 0.021 | 2.62E-06 | 0.027 | 0.024 | 0.247 | 0.004 | | 72.151 |  |
|  |  | rs4383094 | C | T | 0.858 | -0.149 | 0.032 | 4.28E-06 | 0.010 | 0.031 | 0.749 | 0.005 | | 100.115 |  |
|  |  | rs60583455 | T | C | 0.366 | 0.109 | 0.021 | 2.60E-07 | -0.053 | 0.022 | 0.016 | 0.006 | | 101.490 |  |
|  |  | rs60775321 | T | C | 0.290 | -0.096 | 0.021 | 7.10E-06 | -0.007 | 0.023 | 0.764 | 0.004 | | 70.244 |  |
|  |  | rs72671304 | T | C | 0.074 | 0.172 | 0.037 | 3.80E-06 | -0.070 | 0.040 | 0.085 | 0.004 | | 75.037 |  |
|  |  | rs7911787 | G | T | 0.032 | -0.223 | 0.047 | 3.39E-06 | -0.002 | 0.060 | 0.979 | 0.003 | | 56.741 |  |
|  |  | rs8126061 | T | C | 0.126 | -0.159 | 0.035 | 7.36E-06 | 0.026 | 0.032 | 0.417 | 0.006 | | 102.714 |  |
|  |  | rs9542068 | T | C | 0.335 | 0.099 | 0.022 | 6.52E-06 | 0.024 | 0.022 | 0.288 | 0.004 | | 80.594 |  |
| ebi-a-GCST90017105 | Gut microbiota abundance (order Pasteurellales id.3688) | rs10965428 | C | A | 0.056 | -0.120 | 0.026 | 4.29E-06 | 0.056 | 0.046 | 0.223 | 0.002 | | 28.099 |  |
|  |  | rs111582866 | G | A | 0.087 | -0.114 | 0.026 | 7.07E-06 | -0.005 | 0.038 | 0.892 | 0.002 | | 37.874 |  |
|  |  | rs12050685 | A | G | 0.280 | -0.067 | 0.015 | 9.19E-06 | -0.033 | 0.023 | 0.160 | 0.002 | | 33.368 |  |
|  |  | rs16970009 | A | G | 0.020 | 0.187 | 0.043 | 7.32E-06 | 0.002 | 0.075 | 0.982 | 0.001 | | 25.448 |  |
|  |  | rs35510 | A | G | 0.043 | 0.123 | 0.026 | 4.02E-06 | -0.030 | 0.052 | 0.567 | 0.001 | | 22.584 |  |
|  |  | rs4822728 | T | C | 0.483 | 0.069 | 0.015 | 4.72E-06 | 0.054 | 0.021 | 0.012 | 0.002 | | 43.137 |  |
|  |  | rs6972479 | A | G | 0.205 | -0.078 | 0.018 | 7.75E-06 | 0.059 | 0.026 | 0.025 | 0.002 | | 36.652 |  |
|  |  | rs72756943 | G | A | 0.066 | 0.140 | 0.030 | 3.35E-06 | -0.008 | 0.043 | 0.844 | 0.002 | | 44.456 |  |
|  |  | rs73139353 | A | C | 0.088 | -0.223 | 0.048 | 8.71E-06 | 0.022 | 0.038 | 0.559 | 0.008 | | 146.924 |  |
|  |  | rs76022354 | C | T | 0.050 | 0.243 | 0.050 | 1.83E-06 | 0.000 | 0.049 | 0.997 | 0.006 | | 102.982 |  |
|  |  | rs78909003 | T | C | 0.055 | -0.241 | 0.050 | 2.05E-06 | -0.019 | 0.047 | 0.688 | 0.006 | | 111.210 |  |
|  |  | rs9382510 | C | T | 0.265 | -0.088 | 0.017 | 2.48E-07 | 0.020 | 0.024 | 0.415 | 0.003 | | 55.710 |  |
|  |  | rs9895850 | T | C | 0.044 | -0.176 | 0.041 | 9.08E-06 | -0.054 | 0.052 | 0.300 | 0.003 | | 48.051 |  |
|  |  | rs9938097 | C | T | 0.611 | 0.071 | 0.016 | 8.23E-06 | 0.035 | 0.022 | 0.108 | 0.002 | | 44.097 |  |
| ebi-a-GCST90017106 | Gut microbiota abundance (order Rhodospirillales id.2667) | rs1035406 | G | A | 0.116 | -0.115 | 0.025 | 4.07E-06 | -0.001 | 0.033 | 0.972 | 0.003 | | 49.672 |  |
|  |  | rs11591293 | G | T | 0.424 | 0.072 | 0.016 | 4.69E-06 | -0.006 | 0.021 | 0.774 | 0.003 | | 46.898 |  |
|  |  | rs11630875 | T | C | 0.127 | 0.095 | 0.020 | 3.70E-06 | 0.020 | 0.032 | 0.538 | 0.002 | | 36.400 |  |
|  |  | rs13336560 | C | T | 0.545 | -0.070 | 0.016 | 9.75E-06 | -0.022 | 0.022 | 0.317 | 0.002 | | 44.081 |  |
|  |  | rs1549633 | A | C | 0.116 | 0.100 | 0.022 | 3.88E-06 | 0.059 | 0.033 | 0.075 | 0.002 | | 37.384 |  |
|  |  | rs3730086 | A | G | 0.250 | 0.080 | 0.018 | 7.98E-06 | 0.029 | 0.024 | 0.232 | 0.002 | | 44.203 |  |
|  |  | rs3754624 | C | T | 0.177 | 0.094 | 0.020 | 2.68E-06 | 0.031 | 0.028 | 0.259 | 0.003 | | 47.438 |  |
|  |  | rs4278423 | T | C | 0.062 | 0.105 | 0.023 | 3.98E-06 | 0.036 | 0.044 | 0.415 | 0.001 | | 23.660 |  |
|  |  | rs55876211 | C | T | 0.254 | -0.087 | 0.020 | 7.87E-06 | 0.015 | 0.024 | 0.531 | 0.003 | | 52.200 |  |
|  |  | rs61933850 | G | A | 0.139 | 0.165 | 0.036 | 7.00E-06 | -0.039 | 0.031 | 0.217 | 0.006 | | 119.644 |  |
|  |  | rs7001029 | C | T | 0.093 | 0.121 | 0.026 | 2.83E-06 | 0.042 | 0.037 | 0.255 | 0.002 | | 45.008 |  |
|  |  | rs76784716 | A | G | 0.114 | 0.136 | 0.028 | 1.31E-06 | 0.010 | 0.034 | 0.773 | 0.004 | | 68.814 |  |
|  |  | rs77304857 | C | A | 0.194 | -0.100 | 0.022 | 6.02E-06 | 0.006 | 0.027 | 0.838 | 0.003 | | 57.165 |  |
|  |  | rs9813022 | A | G | 0.376 | -0.083 | 0.016 | 3.07E-07 | -0.003 | 0.022 | 0.909 | 0.003 | | 59.555 |  |
| ebi-a-GCST90017107 | Gut microbiota abundance (order Selenomonadales id.2165) | rs1135612 | G | A | 0.211 | 0.053 | 0.012 | 9.26E-06 | 0.000 | 0.026 | 0.995 | 0.001 | | 17.153 |  |
|  |  | rs13086907 | G | A | 0.222 | 0.063 | 0.013 | 1.95E-06 | -0.027 | 0.026 | 0.298 | 0.001 | | 24.826 |  |
|  |  | rs1643968 | T | C | 0.358 | -0.057 | 0.011 | 4.15E-07 | -0.020 | 0.022 | 0.372 | 0.001 | | 26.976 |  |
|  |  | rs1649999 | A | G | 0.097 | 0.075 | 0.017 | 7.58E-06 | 0.037 | 0.036 | 0.305 | 0.001 | | 18.018 |  |
|  |  | rs2834062 | A | G | 0.313 | 0.049 | 0.011 | 8.44E-06 | -0.030 | 0.023 | 0.194 | 0.001 | | 18.879 |  |
|  |  | rs4463806 | C | T | 0.808 | 0.054 | 0.013 | 7.81E-06 | 0.026 | 0.027 | 0.342 | 0.001 | | 16.851 |  |
|  |  | rs4722181 | T | G | 0.471 | 0.050 | 0.011 | 2.00E-06 | -0.003 | 0.021 | 0.897 | 0.001 | | 22.996 |  |
|  |  | rs60274479 | T | C | 0.198 | -0.066 | 0.013 | 1.16E-06 | -0.023 | 0.027 | 0.395 | 0.001 | | 25.395 |  |
|  |  | rs61249479 | A | C | 0.155 | 0.078 | 0.017 | 2.95E-06 | -0.036 | 0.029 | 0.220 | 0.002 | | 29.068 |  |
|  |  | rs71405394 | G | A | 0.068 | -0.114 | 0.024 | 2.17E-06 | 0.047 | 0.042 | 0.268 | 0.002 | | 30.510 |  |
|  |  | rs73232831 | G | A | 0.035 | -0.152 | 0.031 | 1.87E-06 | 0.021 | 0.057 | 0.720 | 0.002 | | 28.466 |  |
|  |  | rs9423647 | G | A | 0.558 | 0.048 | 0.011 | 6.06E-06 | 0.024 | 0.021 | 0.262 | 0.001 | | 20.705 |  |
| ebi-a-GCST90017108 | Gut microbiota abundance (order Verrucomicrobiales id.4030) | rs111862613 | T | C | 0.178 | 0.091 | 0.020 | 3.74E-06 | 0.013 | 0.029 | 0.662 | 0.002 | | 44.212 |  |
|  |  | rs117107102 | A | G | 0.048 | 0.205 | 0.043 | 2.92E-06 | 0.026 | 0.049 | 0.603 | 0.004 | | 69.938 |  |
|  |  | rs11729256 | T | C | 0.170 | 0.075 | 0.015 | 6.73E-07 | 0.004 | 0.028 | 0.875 | 0.002 | | 29.099 |  |
|  |  | rs12908520 | G | A | 0.426 | 0.062 | 0.013 | 2.17E-06 | 0.017 | 0.021 | 0.429 | 0.002 | | 34.425 |  |
|  |  | rs2602429 | T | C | 0.739 | -0.075 | 0.016 | 2.58E-06 | -0.015 | 0.024 | 0.532 | 0.002 | | 39.582 |  |
|  |  | rs4242783 | A | G | 0.717 | -0.069 | 0.015 | 2.64E-06 | 0.017 | 0.024 | 0.471 | 0.002 | | 35.457 |  |
|  |  | rs4936098 | G | A | 0.651 | -0.065 | 0.014 | 1.12E-06 | 0.005 | 0.022 | 0.832 | 0.002 | | 35.143 |  |
|  |  | rs61779207 | G | A | 0.224 | -0.076 | 0.017 | 6.72E-06 | -0.010 | 0.026 | 0.707 | 0.002 | | 36.674 |  |
|  |  | rs74542928 | T | C | 0.048 | 0.112 | 0.024 | 1.63E-06 | -0.023 | 0.049 | 0.640 | 0.001 | | 21.084 |  |
|  |  | rs9349825 | A | G | 0.190 | -0.070 | 0.015 | 2.54E-06 | 0.044 | 0.027 | 0.103 | 0.002 | | 27.975 |  |
|  |  | rs941682 | G | A | 0.274 | -0.063 | 0.014 | 9.61E-06 | 0.028 | 0.024 | 0.237 | 0.002 | | 29.115 |  |
| ebi-a-GCST90017109 | Gut microbiota abundance (order Victivallales id.2254) | rs1002941 | A | G | 0.755 | -0.105 | 0.023 | 8.15E-06 | 0.000 | 0.025 | 0.997 | 0.004 | | 75.241 |  |
|  |  | rs11770843 | C | T | 0.328 | 0.109 | 0.023 | 1.91E-06 | -0.034 | 0.023 | 0.129 | 0.005 | | 97.382 |  |
|  |  | rs17114848 | G | A | 0.099 | 0.152 | 0.032 | 4.06E-06 | 0.074 | 0.036 | 0.038 | 0.004 | | 76.261 |  |
|  |  | rs2031282 | A | G | 0.184 | 0.122 | 0.027 | 4.38E-06 | -0.008 | 0.028 | 0.764 | 0.004 | | 82.829 |  |
|  |  | rs2825714 | A | G | 0.168 | -0.137 | 0.029 | 1.72E-06 | 0.012 | 0.028 | 0.677 | 0.005 | | 97.494 |  |
|  |  | rs62570196 | C | T | 0.042 | -0.216 | 0.044 | 1.08E-06 | 0.023 | 0.053 | 0.658 | 0.004 | | 69.287 |  |
|  |  | rs72640280 | A | G | 0.055 | 0.220 | 0.049 | 5.18E-06 | 0.006 | 0.046 | 0.889 | 0.005 | | 93.335 |  |
|  |  | rs77599476 | A | G | 0.056 | 0.230 | 0.048 | 1.86E-06 | -0.087 | 0.046 | 0.059 | 0.006 | | 103.300 |  |
| ebi-a-GCST90017110 | Gut microbiota abundance (phylum Actinobacteria id.400) | rs11766971 | C | T | 0.448 | -0.048 | 0.011 | 9.40E-06 | 0.017 | 0.021 | 0.425 | 0.001 | | 20.608 |  |
|  |  | rs12528285 | C | T | 0.109 | 0.081 | 0.018 | 5.69E-06 | 0.000 | 0.034 | 0.991 | 0.001 | | 23.425 |  |
|  |  | rs13192624 | T | C | 0.244 | -0.052 | 0.012 | 9.33E-06 | 0.011 | 0.025 | 0.665 | 0.001 | | 18.488 |  |
|  |  | rs1397793 | A | G | 0.690 | 0.052 | 0.011 | 3.74E-06 | -0.035 | 0.023 | 0.134 | 0.001 | | 21.340 |  |
|  |  | rs4429415 | C | T | 0.445 | 0.058 | 0.011 | 2.05E-07 | 0.018 | 0.021 | 0.389 | 0.002 | | 30.728 |  |
|  |  | rs55888705 | A | G | 0.282 | 0.053 | 0.011 | 1.31E-06 | -0.020 | 0.023 | 0.390 | 0.001 | | 21.204 |  |
|  |  | rs6496870 | C | T | 0.642 | -0.051 | 0.011 | 4.62E-06 | 0.011 | 0.022 | 0.604 | 0.001 | | 22.029 |  |
|  |  | rs6743026 | T | C | 0.193 | 0.059 | 0.013 | 9.88E-06 | 0.000 | 0.027 | 0.992 | 0.001 | | 19.877 |  |
|  |  | rs74037001 | G | A | 0.089 | -0.082 | 0.017 | 6.71E-07 | -0.021 | 0.037 | 0.566 | 0.001 | | 19.920 |  |
|  |  | rs75211493 | G | A | 0.072 | 0.084 | 0.018 | 9.27E-06 | -0.016 | 0.041 | 0.690 | 0.001 | | 17.369 |  |
|  |  | rs7570971 | A | C | 0.412 | 0.087 | 0.011 | 1.41E-14 | 0.038 | 0.022 | 0.074 | 0.004 | | 66.959 |  |
|  |  | rs80124826 | T | C | 0.027 | -0.124 | 0.028 | 8.75E-06 | -0.002 | 0.065 | 0.979 | 0.001 | | 14.985 |  |
|  |  | rs8047955 | G | A | 0.618 | -0.052 | 0.011 | 2.66E-06 | 0.028 | 0.022 | 0.203 | 0.001 | | 23.555 |  |
|  |  | rs857444 | C | T | 0.369 | 0.051 | 0.011 | 3.80E-06 | -0.035 | 0.022 | 0.108 | 0.001 | | 21.995 |  |
|  |  | rs9833771 | C | T | 0.482 | -0.049 | 0.011 | 4.07E-06 | -0.007 | 0.021 | 0.738 | 0.001 | | 22.018 |  |
| ebi-a-GCST90017111 | Gut microbiota abundance (phylum Bacteroidetes id.905) | rs17343978 | A | C | 0.204 | -0.056 | 0.012 | 7.22E-06 | 0.018 | 0.026 | 0.482 | 0.001 | | 18.452 |  |
|  |  | rs2032750 | C | T | 0.539 | 0.051 | 0.011 | 1.71E-06 | 0.010 | 0.021 | 0.634 | 0.001 | | 23.816 |  |
|  |  | rs62531359 | T | G | 0.184 | 0.066 | 0.015 | 8.42E-06 | 0.013 | 0.028 | 0.631 | 0.001 | | 23.858 |  |
|  |  | rs62575403 | C | T | 0.042 | 0.145 | 0.031 | 2.96E-06 | 0.030 | 0.053 | 0.569 | 0.002 | | 31.454 |  |
|  |  | rs6586324 | T | C | 0.426 | 0.048 | 0.011 | 7.37E-06 | -0.011 | 0.021 | 0.594 | 0.001 | | 20.493 |  |
|  |  | rs72706335 | T | C | 0.026 | -0.223 | 0.049 | 7.13E-06 | -0.055 | 0.067 | 0.415 | 0.002 | | 45.531 |  |
|  |  | rs73512608 | G | A | 0.053 | -0.123 | 0.024 | 2.54E-07 | -0.012 | 0.047 | 0.792 | 0.002 | | 27.965 |  |
|  |  | rs73846128 | A | G | 0.149 | -0.066 | 0.013 | 4.78E-07 | -0.019 | 0.030 | 0.522 | 0.001 | | 20.497 |  |
|  |  | rs73975615 | G | A | 0.008 | -0.207 | 0.044 | 1.20E-06 | -0.166 | 0.124 | 0.180 | 0.001 | | 11.740 |  |
|  |  | rs7999780 | G | A | 0.274 | 0.054 | 0.012 | 9.47E-06 | -0.022 | 0.024 | 0.360 | 0.001 | | 21.592 |  |
|  |  | rs929878 | T | C | 0.787 | 0.054 | 0.012 | 6.51E-06 | -0.008 | 0.026 | 0.769 | 0.001 | | 17.958 |  |
| ebi-a-GCST90017112 | Gut microbiota abundance (phylum Cyanobacteria id.1500) | rs12555298 | G | A | 0.173 | 0.097 | 0.022 | 8.09E-06 | 0.037 | 0.028 | 0.184 | 0.003 | | 50.017 |  |
|  |  | rs2585223 | T | C | 0.123 | 0.111 | 0.025 | 8.86E-06 | 0.006 | 0.032 | 0.861 | 0.003 | | 49.163 |  |
|  |  | rs584122 | T | C | 0.941 | 0.152 | 0.033 | 4.23E-06 | -0.018 | 0.045 | 0.688 | 0.003 | | 47.250 |  |
|  |  | rs61972390 | T | C | 0.137 | 0.107 | 0.024 | 9.11E-06 | -0.040 | 0.031 | 0.197 | 0.003 | | 50.135 |  |
|  |  | rs7148504 | T | G | 0.600 | -0.080 | 0.018 | 6.62E-06 | 0.018 | 0.022 | 0.408 | 0.003 | | 56.579 |  |
|  |  | rs76531781 | T | C | 0.040 | -0.232 | 0.049 | 2.87E-06 | 0.025 | 0.054 | 0.638 | 0.004 | | 76.049 |  |
|  |  | rs789068 | G | A | 0.141 | -0.111 | 0.021 | 1.57E-07 | 0.002 | 0.030 | 0.958 | 0.003 | | 55.311 |  |
|  |  | rs9864379 | T | C | 0.150 | -0.139 | 0.027 | 2.03E-07 | -0.008 | 0.030 | 0.801 | 0.005 | | 90.558 |  |
| ebi-a-GCST90017113 | Gut microbiota abundance (phylum Euryarchaeota id.55) | rs10202904 | G | T | 0.592 | 0.116 | 0.023 | 6.19E-07 | -0.024 | 0.022 | 0.263 | 0.007 | | 119.979 |  |
|  |  | rs11022995 | A | G | 0.515 | 0.104 | 0.023 | 7.73E-06 | 0.011 | 0.021 | 0.594 | 0.005 | | 99.099 |  |
|  |  | rs34928225 | T | C | 0.093 | 0.200 | 0.043 | 4.33E-06 | 0.027 | 0.036 | 0.465 | 0.007 | | 124.250 |  |
|  |  | rs45498998 | G | A | 0.155 | -0.132 | 0.029 | 5.32E-06 | -0.015 | 0.029 | 0.601 | 0.005 | | 83.798 |  |
|  |  | rs56131665 | G | A | 0.107 | 0.177 | 0.039 | 5.30E-06 | 0.020 | 0.034 | 0.564 | 0.006 | | 110.514 |  |
|  |  | rs6064552 | T | C | 0.183 | -0.124 | 0.028 | 9.34E-06 | -0.032 | 0.027 | 0.243 | 0.005 | | 84.136 |  |
|  |  | rs6508769 | C | T | 0.853 | -0.151 | 0.034 | 8.12E-06 | 0.011 | 0.030 | 0.704 | 0.006 | | 104.563 |  |
|  |  | rs7015093 | G | A | 0.261 | -0.118 | 0.026 | 7.20E-06 | 0.045 | 0.024 | 0.062 | 0.005 | | 99.218 |  |
|  |  | rs76029318 | T | C | 0.063 | 0.215 | 0.044 | 1.05E-06 | 0.008 | 0.043 | 0.846 | 0.005 | | 100.292 |  |
|  |  | rs7635189 | A | G | 0.711 | -0.120 | 0.026 | 4.64E-06 | -0.003 | 0.024 | 0.902 | 0.006 | | 109.181 |  |
|  |  | rs77658038 | A | C | 0.204 | -0.160 | 0.034 | 4.75E-06 | -0.023 | 0.026 | 0.390 | 0.008 | | 154.272 |  |
|  |  | rs894996 | C | A | 0.070 | 0.204 | 0.044 | 5.12E-06 | 0.028 | 0.041 | 0.492 | 0.005 | | 99.435 |  |
| ebi-a-GCST90017114 | Gut microbiota abundance (phylum Firmicutes id.1672) | rs112334273 | G | A | 0.278 | 0.063 | 0.013 | 9.26E-07 | -0.030 | 0.024 | 0.203 | 0.002 | | 29.028 |  |
|  |  | rs2273429 | A | G | 0.105 | -0.070 | 0.015 | 9.26E-06 | 0.053 | 0.035 | 0.128 | 0.001 | | 16.935 |  |
|  |  | rs2332027 | A | G | 0.405 | 0.048 | 0.010 | 4.05E-06 | -0.011 | 0.022 | 0.612 | 0.001 | | 20.610 |  |
|  |  | rs2547978 | G | A | 0.626 | -0.047 | 0.011 | 8.57E-06 | 0.002 | 0.022 | 0.930 | 0.001 | | 18.646 |  |
|  |  | rs3792064 | G | A | 0.053 | 0.090 | 0.018 | 6.75E-07 | -0.081 | 0.047 | 0.085 | 0.001 | | 14.742 |  |
|  |  | rs3852931 | T | C | 0.611 | 0.048 | 0.011 | 4.53E-06 | 0.024 | 0.022 | 0.278 | 0.001 | | 20.282 |  |
|  |  | rs4750583 | G | A | 0.811 | -0.062 | 0.014 | 5.79E-06 | 0.009 | 0.027 | 0.749 | 0.001 | | 21.322 |  |
|  |  | rs56199908 | T | C | 0.052 | -0.186 | 0.041 | 8.67E-06 | -0.010 | 0.048 | 0.826 | 0.003 | | 63.307 |  |
|  |  | rs6814436 | C | T | 0.859 | -0.068 | 0.015 | 6.80E-06 | 0.004 | 0.030 | 0.907 | 0.001 | | 20.552 |  |
|  |  | rs6815608 | C | T | 0.849 | -0.094 | 0.021 | 7.24E-06 | 0.006 | 0.030 | 0.847 | 0.002 | | 41.377 |  |
|  |  | rs7247191 | T | C | 0.099 | -0.071 | 0.016 | 4.73E-06 | -0.022 | 0.035 | 0.542 | 0.001 | | 16.717 |  |
|  |  | rs72738886 | T | C | 0.076 | 0.086 | 0.019 | 7.68E-06 | 0.006 | 0.040 | 0.882 | 0.001 | | 19.304 |  |
|  |  | rs72771021 | C | T | 0.069 | -0.141 | 0.031 | 5.12E-06 | 0.018 | 0.042 | 0.662 | 0.003 | | 47.269 |  |
|  |  | rs8085381 | A | G | 0.817 | -0.065 | 0.015 | 8.67E-06 | -0.022 | 0.027 | 0.416 | 0.001 | | 23.004 |  |
|  |  | rs992074 | T | C | 0.977 | -0.233 | 0.051 | 8.52E-06 | -0.063 | 0.071 | 0.371 | 0.002 | | 45.041 |  |
| ebi-a-GCST90017115 | Gut microbiota abundance (phylum Lentisphaerae id.2238) | rs1002941 | A | G | 0.755 | -0.108 | 0.023 | 4.31E-06 | 0.000 | 0.025 | 0.997 | 0.004 | | 79.082 |  |
|  |  | rs11770843 | C | T | 0.328 | 0.112 | 0.023 | 1.14E-06 | -0.034 | 0.023 | 0.129 | 0.006 | | 101.843 |  |
|  |  | rs17114848 | G | A | 0.099 | 0.149 | 0.032 | 6.77E-06 | 0.074 | 0.036 | 0.038 | 0.004 | | 72.878 |  |
|  |  | rs2031282 | A | G | 0.184 | 0.120 | 0.027 | 5.86E-06 | -0.008 | 0.028 | 0.764 | 0.004 | | 80.191 |  |
|  |  | rs2825714 | A | G | 0.168 | -0.138 | 0.029 | 1.50E-06 | 0.012 | 0.028 | 0.677 | 0.005 | | 98.662 |  |
|  |  | rs60995569 | T | G | 0.110 | -0.161 | 0.034 | 9.19E-06 | 0.018 | 0.034 | 0.590 | 0.005 | | 92.879 |  |
|  |  | rs62570196 | C | T | 0.042 | -0.217 | 0.044 | 9.64E-07 | 0.023 | 0.053 | 0.658 | 0.004 | | 69.836 |  |
|  |  | rs72640280 | A | G | 0.055 | 0.220 | 0.049 | 5.19E-06 | 0.006 | 0.046 | 0.889 | 0.005 | | 93.317 |  |
|  |  | rs77599476 | A | G | 0.056 | 0.230 | 0.048 | 1.90E-06 | -0.087 | 0.046 | 0.059 | 0.006 | | 103.020 |  |
| ebi-a-GCST90017116 | Gut microbiota abundance (phylum Proteobacteria id.2375) | rs10750258 | C | A | 0.634 | 0.049 | 0.011 | 8.72E-06 | 0.001 | 0.022 | 0.960 | 0.001 | | 20.568 |  |
|  |  | rs11126162 | T | C | 0.083 | -0.077 | 0.019 | 9.26E-06 | 0.013 | 0.038 | 0.734 | 0.001 | | 16.597 |  |
|  |  | rs11715072 | G | A | 0.301 | -0.052 | 0.012 | 6.90E-06 | -0.002 | 0.023 | 0.930 | 0.001 | | 20.820 |  |
|  |  | rs12150865 | C | T | 0.486 | 0.051 | 0.011 | 1.54E-06 | -0.019 | 0.021 | 0.378 | 0.001 | | 24.013 |  |
|  |  | rs12467198 | C | T | 0.448 | 0.050 | 0.011 | 6.31E-06 | 0.007 | 0.021 | 0.738 | 0.001 | | 22.544 |  |
|  |  | rs2347697 | G | T | 0.309 | 0.050 | 0.011 | 4.27E-06 | -0.010 | 0.023 | 0.665 | 0.001 | | 19.774 |  |
|  |  | rs2532663 | A | G | 0.898 | 0.126 | 0.026 | 7.47E-07 | -0.041 | 0.035 | 0.242 | 0.003 | | 53.084 |  |
|  |  | rs3890996 | G | T | 0.536 | 0.047 | 0.011 | 6.95E-06 | 0.055 | 0.021 | 0.010 | 0.001 | | 20.568 |  |
|  |  | rs4340090 | C | T | 0.118 | -0.067 | 0.015 | 9.99E-06 | -0.009 | 0.033 | 0.785 | 0.001 | | 17.044 |  |
|  |  | rs6707783 | C | T | 0.091 | 0.085 | 0.019 | 8.09E-06 | 0.054 | 0.037 | 0.146 | 0.001 | | 21.966 |  |
|  |  | rs72771021 | C | T | 0.069 | 0.142 | 0.031 | 7.18E-06 | 0.018 | 0.042 | 0.662 | 0.003 | | 47.516 |  |
|  |  | rs922773 | C | T | 0.101 | -0.080 | 0.016 | 3.68E-07 | -0.018 | 0.035 | 0.611 | 0.001 | | 21.616 |  |
| ebi-a-GCST90017117 | Gut microbiota abundance (phylum Tenericutes id.3919) | rs10108398 | G | A | 0.276 | 0.077 | 0.015 | 1.09E-06 | -0.002 | 0.024 | 0.947 | 0.002 | | 43.409 |  |
|  |  | rs11890098 | A | G | 0.278 | 0.074 | 0.015 | 9.57E-07 | -0.003 | 0.024 | 0.908 | 0.002 | | 40.890 |  |
|  |  | rs12566890 | T | G | 0.132 | -0.101 | 0.023 | 3.65E-06 | -0.007 | 0.032 | 0.825 | 0.002 | | 43.037 |  |
|  |  | rs17214486 | C | A | 0.323 | 0.061 | 0.014 | 6.61E-06 | -0.033 | 0.023 | 0.145 | 0.002 | | 29.901 |  |
|  |  | rs2464826 | A | C | 0.113 | 0.094 | 0.021 | 8.39E-06 | -0.004 | 0.034 | 0.913 | 0.002 | | 32.834 |  |
|  |  | rs28537087 | G | A | 0.249 | 0.082 | 0.019 | 8.07E-06 | -0.009 | 0.025 | 0.721 | 0.003 | | 46.318 |  |
|  |  | rs3768491 | G | A | 0.712 | 0.068 | 0.015 | 4.23E-06 | 0.023 | 0.023 | 0.321 | 0.002 | | 34.971 |  |
|  |  | rs4885016 | C | T | 0.865 | 0.082 | 0.018 | 7.27E-06 | 0.000 | 0.031 | 0.995 | 0.002 | | 28.779 |  |
|  |  | rs6043847 | T | C | 0.059 | -0.115 | 0.025 | 4.55E-06 | 0.073 | 0.045 | 0.106 | 0.001 | | 26.883 |  |
|  |  | rs72901605 | T | C | 0.115 | -0.084 | 0.018 | 3.26E-06 | 0.021 | 0.033 | 0.525 | 0.001 | | 26.452 |  |
|  |  | rs74603314 | T | C | 0.040 | 0.222 | 0.046 | 1.56E-06 | -0.007 | 0.054 | 0.898 | 0.004 | | 69.646 |  |
|  |  | rs78169027 | A | G | 0.061 | -0.108 | 0.024 | 5.88E-06 | 0.032 | 0.045 | 0.476 | 0.001 | | 24.835 |  |
| ebi-a-GCST90017118 | Gut microbiota abundance (phylum Verrucomicrobia id.3982) | rs11252894 | A | C | 0.241 | 0.078 | 0.016 | 1.11E-06 | -0.020 | 0.025 | 0.433 | 0.002 | | 41.334 |  |
|  |  | rs117107102 | A | G | 0.048 | 0.204 | 0.043 | 2.68E-06 | 0.026 | 0.049 | 0.603 | 0.004 | | 69.664 |  |
|  |  | rs11729256 | T | C | 0.170 | 0.070 | 0.015 | 2.23E-06 | 0.004 | 0.028 | 0.875 | 0.001 | | 25.139 |  |
|  |  | rs12512971 | A | C | 0.079 | 0.171 | 0.040 | 9.81E-06 | 0.024 | 0.039 | 0.538 | 0.004 | | 78.069 |  |
|  |  | rs12908520 | G | A | 0.426 | 0.059 | 0.013 | 3.40E-06 | 0.017 | 0.021 | 0.429 | 0.002 | | 31.801 |  |
|  |  | rs2602429 | T | C | 0.739 | -0.076 | 0.015 | 8.71E-07 | -0.015 | 0.024 | 0.532 | 0.002 | | 41.441 |  |
|  |  | rs3995795 | C | T | 0.402 | 0.061 | 0.014 | 9.72E-06 | 0.035 | 0.022 | 0.110 | 0.002 | | 32.962 |  |
|  |  | rs45598138 | C | A | 0.025 | -0.144 | 0.031 | 2.19E-06 | -0.100 | 0.067 | 0.138 | 0.001 | | 18.610 |  |
|  |  | rs61779207 | G | A | 0.224 | -0.076 | 0.016 | 5.28E-06 | -0.010 | 0.026 | 0.707 | 0.002 | | 36.379 |  |
|  |  | rs74542928 | T | C | 0.048 | 0.116 | 0.023 | 4.08E-07 | -0.023 | 0.049 | 0.640 | 0.001 | | 22.550 |  |
|  |  | rs76430504 | T | C | 0.048 | -0.118 | 0.025 | 3.50E-06 | 0.029 | 0.049 | 0.557 | 0.001 | | 23.359 |  |
|  |  | rs9349825 | A | G | 0.190 | -0.066 | 0.014 | 6.27E-06 | 0.044 | 0.027 | 0.103 | 0.001 | | 24.599 |  |

| Supplementary Table 4: All results of MR analysIs. | | | | | | | | | |
| --- | --- | --- | --- | --- | --- | --- | --- | --- | --- |
| id.exposure | Gut microbiota | id.outcome | method | nsnp | se | pval | or | or_lci95 | or_uci95 |
| ebi-a-GCST90016959 | Gut microbiota abundance (genus Actinomyces id.423) | finn-b-N14_CALCUKIDUR | Inverse variance weighted | 7 | 0.116 | 0.580 | 1.066 | 0.850 | 1.337 |
| ebi-a-GCST90016959 | Gut microbiota abundance (genus Actinomyces id.423) | finn-b-N14_CALCUKIDUR | MR Egger | 7 | 0.310 | 0.766 | 1.102 | 0.600 | 2.023 |
| ebi-a-GCST90016959 | Gut microbiota abundance (genus Actinomyces id.423) | finn-b-N14_CALCUKIDUR | Weighted median | 7 | 0.128 | 0.873 | 1.021 | 0.795 | 1.311 |
| ebi-a-GCST90016959 | Gut microbiota abundance (genus Actinomyces id.423) | finn-b-N14_CALCUKIDUR | Maximum likelihood | 7 | 0.103 | 0.503 | 1.071 | 0.876 | 1.311 |
| ebi-a-GCST90016960 | Gut microbiota abundance (genus Adlercreutzia id.812) | finn-b-N14_CALCUKIDUR | Inverse variance weighted | 8 | 0.105 | 0.424 | 0.919 | 0.748 | 1.130 |
| ebi-a-GCST90016960 | Gut microbiota abundance (genus Adlercreutzia id.812) | finn-b-N14_CALCUKIDUR | MR Egger | 8 | 0.473 | 0.426 | 1.498 | 0.593 | 3.782 |
| ebi-a-GCST90016960 | Gut microbiota abundance (genus Adlercreutzia id.812) | finn-b-N14_CALCUKIDUR | Weighted median | 8 | 0.136 | 0.241 | 0.853 | 0.654 | 1.113 |
| ebi-a-GCST90016960 | Gut microbiota abundance (genus Adlercreutzia id.812) | finn-b-N14_CALCUKIDUR | Maximum likelihood | 8 | 0.107 | 0.424 | 0.918 | 0.744 | 1.132 |
| ebi-a-GCST90016961 | Gut microbiota abundance (genus Akkermansia id.4037) | finn-b-N14_CALCUKIDUR | Inverse variance weighted | 11 | 0.101 | 0.848 | 0.981 | 0.804 | 1.196 |
| ebi-a-GCST90016961 | Gut microbiota abundance (genus Akkermansia id.4037) | finn-b-N14_CALCUKIDUR | MR Egger | 11 | 0.338 | 0.531 | 1.246 | 0.643 | 2.416 |
| ebi-a-GCST90016961 | Gut microbiota abundance (genus Akkermansia id.4037) | finn-b-N14_CALCUKIDUR | Weighted median | 11 | 0.136 | 0.417 | 1.116 | 0.856 | 1.456 |
| ebi-a-GCST90016961 | Gut microbiota abundance (genus Akkermansia id.4037) | finn-b-N14_CALCUKIDUR | Maximum likelihood | 11 | 0.103 | 0.852 | 0.981 | 0.802 | 1.199 |
| ebi-a-GCST90016962 | Gut microbiota abundance (genus Alistipes id.968) | finn-b-N14_CALCUKIDUR | Inverse variance weighted | 13 | 0.126 | 0.855 | 1.023 | 0.799 | 1.311 |
| ebi-a-GCST90016962 | Gut microbiota abundance (genus Alistipes id.968) | finn-b-N14_CALCUKIDUR | MR Egger | 13 | 0.609 | 0.651 | 0.753 | 0.228 | 2.484 |
| ebi-a-GCST90016962 | Gut microbiota abundance (genus Alistipes id.968) | finn-b-N14_CALCUKIDUR | Weighted median | 13 | 0.177 | 0.743 | 0.944 | 0.667 | 1.336 |
| ebi-a-GCST90016962 | Gut microbiota abundance (genus Alistipes id.968) | finn-b-N14_CALCUKIDUR | Maximum likelihood | 13 | 0.128 | 0.850 | 1.025 | 0.797 | 1.318 |
| ebi-a-GCST90016963 | Gut microbiota abundance (genus Allisonella id.2174) | finn-b-N14_CALCUKIDUR | Inverse variance weighted | 8 | 0.078 | 0.829 | 1.017 | 0.873 | 1.185 |
| ebi-a-GCST90016963 | Gut microbiota abundance (genus Allisonella id.2174) | finn-b-N14_CALCUKIDUR | MR Egger | 8 | 0.569 | 0.932 | 1.052 | 0.344 | 3.211 |
| ebi-a-GCST90016963 | Gut microbiota abundance (genus Allisonella id.2174) | finn-b-N14_CALCUKIDUR | Weighted median | 8 | 0.087 | 0.456 | 1.067 | 0.899 | 1.267 |
| ebi-a-GCST90016963 | Gut microbiota abundance (genus Allisonella id.2174) | finn-b-N14_CALCUKIDUR | Maximum likelihood | 8 | 0.064 | 0.779 | 1.018 | 0.898 | 1.155 |
| ebi-a-GCST90016964 | Gut microbiota abundance (genus Alloprevotella id.961) | finn-b-N14_CALCUKIDUR | Inverse variance weighted | 6 | 0.072 | 0.888 | 1.010 | 0.877 | 1.163 |
| ebi-a-GCST90016964 | Gut microbiota abundance (genus Alloprevotella id.961) | finn-b-N14_CALCUKIDUR | MR Egger | 6 | 0.671 | 0.349 | 0.491 | 0.132 | 1.829 |
| ebi-a-GCST90016964 | Gut microbiota abundance (genus Alloprevotella id.961) | finn-b-N14_CALCUKIDUR | Weighted median | 6 | 0.086 | 0.919 | 0.991 | 0.837 | 1.173 |
| ebi-a-GCST90016964 | Gut microbiota abundance (genus Alloprevotella id.961) | finn-b-N14_CALCUKIDUR | Maximum likelihood | 6 | 0.073 | 0.887 | 1.010 | 0.876 | 1.165 |
| ebi-a-GCST90016965 | Gut microbiota abundance (genus Anaerofilum id.2053) | finn-b-N14_CALCUKIDUR | Inverse variance weighted | 11 | 0.070 | 0.874 | 1.011 | 0.882 | 1.160 |
| ebi-a-GCST90016965 | Gut microbiota abundance (genus Anaerofilum id.2053) | finn-b-N14_CALCUKIDUR | MR Egger | 11 | 0.381 | 0.573 | 0.800 | 0.379 | 1.689 |
| ebi-a-GCST90016965 | Gut microbiota abundance (genus Anaerofilum id.2053) | finn-b-N14_CALCUKIDUR | Weighted median | 11 | 0.096 | 0.751 | 1.031 | 0.854 | 1.244 |
| ebi-a-GCST90016965 | Gut microbiota abundance (genus Anaerofilum id.2053) | finn-b-N14_CALCUKIDUR | Maximum likelihood | 11 | 0.071 | 0.869 | 1.012 | 0.880 | 1.163 |
| ebi-a-GCST90016966 | Gut microbiota abundance (genus Anaerostipes id.1991) | finn-b-N14_CALCUKIDUR | Inverse variance weighted | 13 | 0.118 | 0.899 | 0.985 | 0.782 | 1.241 |
| ebi-a-GCST90016966 | Gut microbiota abundance (genus Anaerostipes id.1991) | finn-b-N14_CALCUKIDUR | MR Egger | 13 | 0.428 | 0.262 | 1.658 | 0.717 | 3.835 |
| ebi-a-GCST90016966 | Gut microbiota abundance (genus Anaerostipes id.1991) | finn-b-N14_CALCUKIDUR | Weighted median | 13 | 0.160 | 0.769 | 0.954 | 0.697 | 1.306 |
| ebi-a-GCST90016966 | Gut microbiota abundance (genus Anaerostipes id.1991) | finn-b-N14_CALCUKIDUR | Maximum likelihood | 13 | 0.120 | 0.900 | 0.985 | 0.779 | 1.245 |
| ebi-a-GCST90016967 | Gut microbiota abundance (genus Anaerotruncus id.2054) | finn-b-N14_CALCUKIDUR | Inverse variance weighted | 13 | 0.138 | 0.671 | 1.060 | 0.809 | 1.389 |
| ebi-a-GCST90016967 | Gut microbiota abundance (genus Anaerotruncus id.2054) | finn-b-N14_CALCUKIDUR | MR Egger | 13 | 0.421 | 0.967 | 1.018 | 0.446 | 2.322 |
| ebi-a-GCST90016967 | Gut microbiota abundance (genus Anaerotruncus id.2054) | finn-b-N14_CALCUKIDUR | Weighted median | 13 | 0.157 | 0.999 | 1.000 | 0.736 | 1.360 |
| ebi-a-GCST90016967 | Gut microbiota abundance (genus Anaerotruncus id.2054) | finn-b-N14_CALCUKIDUR | Maximum likelihood | 13 | 0.117 | 0.599 | 1.063 | 0.846 | 1.336 |
| ebi-a-GCST90016968 | Gut microbiota abundance (genus Bacteroides id.918) | finn-b-N14_CALCUKIDUR | Inverse variance weighted | 9 | 0.184 | 0.387 | 1.173 | 0.818 | 1.683 |
| ebi-a-GCST90016968 | Gut microbiota abundance (genus Bacteroides id.918) | finn-b-N14_CALCUKIDUR | MR Egger | 9 | 1.006 | 0.774 | 0.741 | 0.103 | 5.319 |
| ebi-a-GCST90016968 | Gut microbiota abundance (genus Bacteroides id.918) | finn-b-N14_CALCUKIDUR | Weighted median | 9 | 0.190 | 0.800 | 1.049 | 0.723 | 1.521 |
| ebi-a-GCST90016968 | Gut microbiota abundance (genus Bacteroides id.918) | finn-b-N14_CALCUKIDUR | Maximum likelihood | 9 | 0.141 | 0.217 | 1.191 | 0.903 | 1.570 |
| ebi-a-GCST90016969 | Gut microbiota abundance (genus Barnesiella id.944) | finn-b-N14_CALCUKIDUR | Inverse variance weighted | 13 | 0.119 | 0.002 | 0.695 | 0.551 | 0.877 |
| ebi-a-GCST90016969 | Gut microbiota abundance (genus Barnesiella id.944) | finn-b-N14_CALCUKIDUR | MR Egger | 13 | 0.501 | 0.647 | 0.790 | 0.296 | 2.108 |
| ebi-a-GCST90016969 | Gut microbiota abundance (genus Barnesiella id.944) | finn-b-N14_CALCUKIDUR | Weighted median | 13 | 0.149 | 0.030 | 0.724 | 0.541 | 0.969 |
| ebi-a-GCST90016969 | Gut microbiota abundance (genus Barnesiella id.944) | finn-b-N14_CALCUKIDUR | Maximum likelihood | 13 | 0.111 | 0.001 | 0.690 | 0.554 | 0.858 |
| ebi-a-GCST90016970 | Gut microbiota abundance (genus Bifidobacterium id.436) | finn-b-N14_CALCUKIDUR | Inverse variance weighted | 13 | 0.094 | 0.662 | 1.042 | 0.867 | 1.252 |
| ebi-a-GCST90016970 | Gut microbiota abundance (genus Bifidobacterium id.436) | finn-b-N14_CALCUKIDUR | MR Egger | 13 | 0.224 | 0.065 | 1.585 | 1.021 | 2.460 |
| ebi-a-GCST90016970 | Gut microbiota abundance (genus Bifidobacterium id.436) | finn-b-N14_CALCUKIDUR | Weighted median | 13 | 0.135 | 0.918 | 0.986 | 0.757 | 1.285 |
| ebi-a-GCST90016970 | Gut microbiota abundance (genus Bifidobacterium id.436) | finn-b-N14_CALCUKIDUR | Maximum likelihood | 13 | 0.092 | 0.636 | 1.044 | 0.873 | 1.250 |
| ebi-a-GCST90016971 | Gut microbiota abundance (genus Bilophila id.3170) | finn-b-N14_CALCUKIDUR | Inverse variance weighted | 13 | 0.105 | 0.670 | 1.046 | 0.851 | 1.285 |
| ebi-a-GCST90016971 | Gut microbiota abundance (genus Bilophila id.3170) | finn-b-N14_CALCUKIDUR | MR Egger | 13 | 0.516 | 0.764 | 0.853 | 0.310 | 2.347 |
| ebi-a-GCST90016971 | Gut microbiota abundance (genus Bilophila id.3170) | finn-b-N14_CALCUKIDUR | Weighted median | 13 | 0.145 | 0.182 | 1.213 | 0.913 | 1.610 |
| ebi-a-GCST90016971 | Gut microbiota abundance (genus Bilophila id.3170) | finn-b-N14_CALCUKIDUR | Maximum likelihood | 13 | 0.107 | 0.659 | 1.048 | 0.850 | 1.293 |
| ebi-a-GCST90016972 | Gut microbiota abundance (genus Blautia id.1992) | finn-b-N14_CALCUKIDUR | Inverse variance weighted | 2 | 0.304 | 0.075 | 0.581 | 0.320 | 1.055 |
| ebi-a-GCST90016972 | Gut microbiota abundance (genus Blautia id.1992) | finn-b-N14_CALCUKIDUR | Maximum likelihood | 2 | 0.317 | 0.086 | 0.581 | 0.312 | 1.081 |
| ebi-a-GCST90016973 | Gut microbiota abundance (genus Butyricicoccus id.2055) | finn-b-N14_CALCUKIDUR | Inverse variance weighted | 8 | 0.129 | 0.253 | 0.863 | 0.671 | 1.111 |
| ebi-a-GCST90016973 | Gut microbiota abundance (genus Butyricicoccus id.2055) | finn-b-N14_CALCUKIDUR | MR Egger | 8 | 0.253 | 0.342 | 0.770 | 0.469 | 1.264 |
| ebi-a-GCST90016973 | Gut microbiota abundance (genus Butyricicoccus id.2055) | finn-b-N14_CALCUKIDUR | Weighted median | 8 | 0.179 | 0.159 | 0.777 | 0.547 | 1.104 |
| ebi-a-GCST90016973 | Gut microbiota abundance (genus Butyricicoccus id.2055) | finn-b-N14_CALCUKIDUR | Maximum likelihood | 8 | 0.131 | 0.251 | 0.860 | 0.665 | 1.113 |
| ebi-a-GCST90016974 | Gut microbiota abundance (genus Butyricimonas id.945) | finn-b-N14_CALCUKIDUR | Inverse variance weighted | 13 | 0.105 | 0.642 | 1.050 | 0.855 | 1.290 |
| ebi-a-GCST90016974 | Gut microbiota abundance (genus Butyricimonas id.945) | finn-b-N14_CALCUKIDUR | MR Egger | 13 | 0.385 | 0.694 | 0.856 | 0.403 | 1.819 |
| ebi-a-GCST90016974 | Gut microbiota abundance (genus Butyricimonas id.945) | finn-b-N14_CALCUKIDUR | Weighted median | 13 | 0.129 | 0.667 | 0.946 | 0.735 | 1.217 |
| ebi-a-GCST90016974 | Gut microbiota abundance (genus Butyricimonas id.945) | finn-b-N14_CALCUKIDUR | Maximum likelihood | 13 | 0.100 | 0.609 | 1.053 | 0.865 | 1.281 |
| ebi-a-GCST90016975 | Gut microbiota abundance (genus Butyrivibrio id.1993) | finn-b-N14_CALCUKIDUR | Inverse variance weighted | 15 | 0.059 | 0.811 | 0.986 | 0.878 | 1.107 |
| ebi-a-GCST90016975 | Gut microbiota abundance (genus Butyrivibrio id.1993) | finn-b-N14_CALCUKIDUR | MR Egger | 15 | 0.258 | 0.520 | 1.186 | 0.715 | 1.969 |
| ebi-a-GCST90016975 | Gut microbiota abundance (genus Butyrivibrio id.1993) | finn-b-N14_CALCUKIDUR | Weighted median | 15 | 0.066 | 0.596 | 0.965 | 0.848 | 1.100 |
| ebi-a-GCST90016975 | Gut microbiota abundance (genus Butyrivibrio id.1993) | finn-b-N14_CALCUKIDUR | Maximum likelihood | 15 | 0.049 | 0.764 | 0.985 | 0.895 | 1.085 |
| ebi-a-GCST90016976 | Gut microbiota abundance (genus Candidatus Soleaferrea id.11350) | finn-b-N14_CALCUKIDUR | Inverse variance weighted | 11 | 0.086 | 0.377 | 0.927 | 0.783 | 1.097 |
| ebi-a-GCST90016976 | Gut microbiota abundance (genus Candidatus Soleaferrea id.11350) | finn-b-N14_CALCUKIDUR | MR Egger | 11 | 0.424 | 0.122 | 0.485 | 0.211 | 1.113 |
| ebi-a-GCST90016976 | Gut microbiota abundance (genus Candidatus Soleaferrea id.11350) | finn-b-N14_CALCUKIDUR | Weighted median | 11 | 0.106 | 0.148 | 0.858 | 0.697 | 1.056 |
| ebi-a-GCST90016976 | Gut microbiota abundance (genus Candidatus Soleaferrea id.11350) | finn-b-N14_CALCUKIDUR | Maximum likelihood | 11 | 0.077 | 0.300 | 0.923 | 0.793 | 1.074 |
| ebi-a-GCST90016977 | Gut microbiota abundance (genus Catenibacterium id.2153) | finn-b-N14_CALCUKIDUR | Inverse variance weighted | 4 | 0.110 | 0.608 | 0.945 | 0.762 | 1.173 |
| ebi-a-GCST90016977 | Gut microbiota abundance (genus Catenibacterium id.2153) | finn-b-N14_CALCUKIDUR | MR Egger | 4 | 1.523 | 0.550 | 0.338 | 0.017 | 6.684 |
| ebi-a-GCST90016977 | Gut microbiota abundance (genus Catenibacterium id.2153) | finn-b-N14_CALCUKIDUR | Weighted median | 4 | 0.121 | 0.729 | 0.959 | 0.756 | 1.217 |
| ebi-a-GCST90016977 | Gut microbiota abundance (genus Catenibacterium id.2153) | finn-b-N14_CALCUKIDUR | Maximum likelihood | 4 | 0.097 | 0.538 | 0.942 | 0.780 | 1.139 |
| ebi-a-GCST90016978 | Gut microbiota abundance (genus Christensenellaceae R 7group id.11283) | finn-b-N14_CALCUKIDUR | Inverse variance weighted | 10 | 0.175 | 0.123 | 1.310 | 0.930 | 1.845 |
| ebi-a-GCST90016978 | Gut microbiota abundance (genus Christensenellaceae R 7group id.11283) | finn-b-N14_CALCUKIDUR | MR Egger | 10 | 0.465 | 0.035 | 3.240 | 1.301 | 8.066 |
| ebi-a-GCST90016978 | Gut microbiota abundance (genus Christensenellaceae R 7group id.11283) | finn-b-N14_CALCUKIDUR | Weighted median | 10 | 0.189 | 0.049 | 1.450 | 1.002 | 2.099 |
| ebi-a-GCST90016978 | Gut microbiota abundance (genus Christensenellaceae R 7group id.11283) | finn-b-N14_CALCUKIDUR | Maximum likelihood | 10 | 0.139 | 0.034 | 1.343 | 1.022 | 1.764 |
| ebi-a-GCST90016979 | Gut microbiota abundance (genus Clostridium innocuum group id.14397) | finn-b-N14_CALCUKIDUR | Inverse variance weighted | 9 | 0.081 | 0.818 | 0.981 | 0.837 | 1.151 |
| ebi-a-GCST90016979 | Gut microbiota abundance (genus Clostridium innocuum group id.14397) | finn-b-N14_CALCUKIDUR | MR Egger | 9 | 0.421 | 0.972 | 1.015 | 0.445 | 2.316 |
| ebi-a-GCST90016979 | Gut microbiota abundance (genus Clostridium innocuum group id.14397) | finn-b-N14_CALCUKIDUR | Weighted median | 9 | 0.091 | 0.938 | 0.993 | 0.832 | 1.186 |
| ebi-a-GCST90016979 | Gut microbiota abundance (genus Clostridium innocuum group id.14397) | finn-b-N14_CALCUKIDUR | Maximum likelihood | 9 | 0.069 | 0.773 | 0.980 | 0.857 | 1.122 |
| ebi-a-GCST90016980 | Gut microbiota abundance (genus Clostridium sensustricto1 id.1873) | finn-b-N14_CALCUKIDUR | Inverse variance weighted | 7 | 0.122 | 0.038 | 0.777 | 0.612 | 0.986 |
| ebi-a-GCST90016980 | Gut microbiota abundance (genus Clostridium sensustricto1 id.1873) | finn-b-N14_CALCUKIDUR | MR Egger | 7 | 0.311 | 0.514 | 1.244 | 0.676 | 2.291 |
| ebi-a-GCST90016980 | Gut microbiota abundance (genus Clostridium sensustricto1 id.1873) | finn-b-N14_CALCUKIDUR | Weighted median | 7 | 0.168 | 0.270 | 0.831 | 0.598 | 1.154 |
| ebi-a-GCST90016980 | Gut microbiota abundance (genus Clostridium sensustricto1 id.1873) | finn-b-N14_CALCUKIDUR | Maximum likelihood | 7 | 0.129 | 0.039 | 0.767 | 0.595 | 0.987 |
| ebi-a-GCST90016981 | Gut microbiota abundance (genus Collinsella id.815) | finn-b-N14_CALCUKIDUR | Inverse variance weighted | 9 | 0.151 | 0.864 | 1.026 | 0.763 | 1.379 |
| ebi-a-GCST90016981 | Gut microbiota abundance (genus Collinsella id.815) | finn-b-N14_CALCUKIDUR | MR Egger | 9 | 0.579 | 0.513 | 0.671 | 0.216 | 2.086 |
| ebi-a-GCST90016981 | Gut microbiota abundance (genus Collinsella id.815) | finn-b-N14_CALCUKIDUR | Weighted median | 9 | 0.198 | 0.991 | 0.998 | 0.677 | 1.471 |
| ebi-a-GCST90016981 | Gut microbiota abundance (genus Collinsella id.815) | finn-b-N14_CALCUKIDUR | Maximum likelihood | 9 | 0.140 | 0.845 | 1.028 | 0.780 | 1.353 |
| ebi-a-GCST90016982 | Gut microbiota abundance (genus Coprobacter id.949) | finn-b-N14_CALCUKIDUR | Inverse variance weighted | 10 | 0.082 | 0.391 | 1.073 | 0.913 | 1.262 |
| ebi-a-GCST90016982 | Gut microbiota abundance (genus Coprobacter id.949) | finn-b-N14_CALCUKIDUR | MR Egger | 10 | 0.308 | 0.386 | 1.327 | 0.725 | 2.427 |
| ebi-a-GCST90016982 | Gut microbiota abundance (genus Coprobacter id.949) | finn-b-N14_CALCUKIDUR | Weighted median | 10 | 0.105 | 0.482 | 1.077 | 0.876 | 1.323 |
| ebi-a-GCST90016982 | Gut microbiota abundance (genus Coprobacter id.949) | finn-b-N14_CALCUKIDUR | Maximum likelihood | 10 | 0.083 | 0.385 | 1.075 | 0.913 | 1.266 |
| ebi-a-GCST90016983 | Gut microbiota abundance (genus Coprococcus1 id.11301) | finn-b-N14_CALCUKIDUR | Inverse variance weighted | 11 | 0.113 | 0.807 | 1.028 | 0.824 | 1.283 |
| ebi-a-GCST90016983 | Gut microbiota abundance (genus Coprococcus1 id.11301) | finn-b-N14_CALCUKIDUR | MR Egger | 11 | 0.279 | 0.464 | 0.808 | 0.468 | 1.395 |
| ebi-a-GCST90016983 | Gut microbiota abundance (genus Coprococcus1 id.11301) | finn-b-N14_CALCUKIDUR | Weighted median | 11 | 0.151 | 0.929 | 0.987 | 0.734 | 1.327 |
| ebi-a-GCST90016983 | Gut microbiota abundance (genus Coprococcus1 id.11301) | finn-b-N14_CALCUKIDUR | Maximum likelihood | 11 | 0.115 | 0.802 | 1.029 | 0.822 | 1.289 |
| ebi-a-GCST90016984 | Gut microbiota abundance (genus Coprococcus2 id.11302) | finn-b-N14_CALCUKIDUR | Inverse variance weighted | 8 | 0.179 | 0.520 | 1.122 | 0.790 | 1.593 |
| ebi-a-GCST90016984 | Gut microbiota abundance (genus Coprococcus2 id.11302) | finn-b-N14_CALCUKIDUR | MR Egger | 8 | 1.210 | 0.096 | 10.859 | 1.013 | 116.451 |
| ebi-a-GCST90016984 | Gut microbiota abundance (genus Coprococcus2 id.11302) | finn-b-N14_CALCUKIDUR | Weighted median | 8 | 0.167 | 0.709 | 0.940 | 0.678 | 1.303 |
| ebi-a-GCST90016984 | Gut microbiota abundance (genus Coprococcus2 id.11302) | finn-b-N14_CALCUKIDUR | Maximum likelihood | 8 | 0.130 | 0.331 | 1.134 | 0.880 | 1.462 |
| ebi-a-GCST90016985 | Gut microbiota abundance (genus Coprococcus3 id.11303) | finn-b-N14_CALCUKIDUR | Inverse variance weighted | 9 | 0.140 | 0.464 | 1.108 | 0.842 | 1.457 |
| ebi-a-GCST90016985 | Gut microbiota abundance (genus Coprococcus3 id.11303) | finn-b-N14_CALCUKIDUR | MR Egger | 9 | 0.768 | 0.576 | 0.638 | 0.142 | 2.874 |
| ebi-a-GCST90016985 | Gut microbiota abundance (genus Coprococcus3 id.11303) | finn-b-N14_CALCUKIDUR | Weighted median | 9 | 0.180 | 0.542 | 1.116 | 0.784 | 1.589 |
| ebi-a-GCST90016985 | Gut microbiota abundance (genus Coprococcus3 id.11303) | finn-b-N14_CALCUKIDUR | Maximum likelihood | 9 | 0.141 | 0.461 | 1.110 | 0.842 | 1.463 |
| ebi-a-GCST90016986 | Gut microbiota abundance (genus Defluviitaleaceae UCG011 id.11287) | finn-b-N14_CALCUKIDUR | Inverse variance weighted | 9 | 0.101 | 0.929 | 0.991 | 0.814 | 1.207 |
| ebi-a-GCST90016986 | Gut microbiota abundance (genus Defluviitaleaceae UCG011 id.11287) | finn-b-N14_CALCUKIDUR | MR Egger | 9 | 0.363 | 0.783 | 0.901 | 0.442 | 1.837 |
| ebi-a-GCST90016986 | Gut microbiota abundance (genus Defluviitaleaceae UCG011 id.11287) | finn-b-N14_CALCUKIDUR | Weighted median | 9 | 0.128 | 0.764 | 0.962 | 0.748 | 1.238 |
| ebi-a-GCST90016986 | Gut microbiota abundance (genus Defluviitaleaceae UCG011 id.11287) | finn-b-N14_CALCUKIDUR | Maximum likelihood | 9 | 0.102 | 0.929 | 0.991 | 0.812 | 1.210 |
| ebi-a-GCST90016987 | Gut microbiota abundance (genus Desulfovibrio id.3173) | finn-b-N14_CALCUKIDUR | Inverse variance weighted | 10 | 0.096 | 0.877 | 1.015 | 0.841 | 1.226 |
| ebi-a-GCST90016987 | Gut microbiota abundance (genus Desulfovibrio id.3173) | finn-b-N14_CALCUKIDUR | MR Egger | 10 | 0.302 | 0.564 | 1.199 | 0.664 | 2.166 |
| ebi-a-GCST90016987 | Gut microbiota abundance (genus Desulfovibrio id.3173) | finn-b-N14_CALCUKIDUR | Weighted median | 10 | 0.130 | 0.956 | 0.993 | 0.770 | 1.280 |
| ebi-a-GCST90016987 | Gut microbiota abundance (genus Desulfovibrio id.3173) | finn-b-N14_CALCUKIDUR | Maximum likelihood | 10 | 0.097 | 0.873 | 1.016 | 0.839 | 1.230 |
| ebi-a-GCST90016988 | Gut microbiota abundance (genus Dialister id.2183) | finn-b-N14_CALCUKIDUR | Inverse variance weighted | 11 | 0.106 | 0.110 | 1.184 | 0.962 | 1.456 |
| ebi-a-GCST90016988 | Gut microbiota abundance (genus Dialister id.2183) | finn-b-N14_CALCUKIDUR | MR Egger | 11 | 0.434 | 0.977 | 1.013 | 0.433 | 2.371 |
| ebi-a-GCST90016988 | Gut microbiota abundance (genus Dialister id.2183) | finn-b-N14_CALCUKIDUR | Weighted median | 11 | 0.143 | 0.204 | 1.199 | 0.906 | 1.585 |
| ebi-a-GCST90016988 | Gut microbiota abundance (genus Dialister id.2183) | finn-b-N14_CALCUKIDUR | Maximum likelihood | 11 | 0.108 | 0.109 | 1.189 | 0.962 | 1.470 |
| ebi-a-GCST90016989 | Gut microbiota abundance (genus Dorea id.1997) | finn-b-N14_CALCUKIDUR | Inverse variance weighted | 10 | 0.135 | 0.218 | 1.181 | 0.906 | 1.540 |
| ebi-a-GCST90016989 | Gut microbiota abundance (genus Dorea id.1997) | finn-b-N14_CALCUKIDUR | MR Egger | 10 | 0.377 | 0.975 | 1.012 | 0.484 | 2.118 |
| ebi-a-GCST90016989 | Gut microbiota abundance (genus Dorea id.1997) | finn-b-N14_CALCUKIDUR | Weighted median | 10 | 0.188 | 0.455 | 1.151 | 0.796 | 1.665 |
| ebi-a-GCST90016989 | Gut microbiota abundance (genus Dorea id.1997) | finn-b-N14_CALCUKIDUR | Maximum likelihood | 10 | 0.138 | 0.215 | 1.187 | 0.906 | 1.555 |
| ebi-a-GCST90016990 | Gut microbiota abundance (genus Eggerthella id.819) | finn-b-N14_CALCUKIDUR | Inverse variance weighted | 10 | 0.075 | 0.568 | 1.044 | 0.901 | 1.209 |
| ebi-a-GCST90016990 | Gut microbiota abundance (genus Eggerthella id.819) | finn-b-N14_CALCUKIDUR | MR Egger | 10 | 0.346 | 0.822 | 0.923 | 0.469 | 1.817 |
| ebi-a-GCST90016990 | Gut microbiota abundance (genus Eggerthella id.819) | finn-b-N14_CALCUKIDUR | Weighted median | 10 | 0.099 | 0.637 | 1.048 | 0.863 | 1.274 |
| ebi-a-GCST90016990 | Gut microbiota abundance (genus Eggerthella id.819) | finn-b-N14_CALCUKIDUR | Maximum likelihood | 10 | 0.076 | 0.568 | 1.044 | 0.900 | 1.212 |
| ebi-a-GCST90016991 | Gut microbiota abundance (genus Eisenbergiella id.11304) | finn-b-N14_CALCUKIDUR | Inverse variance weighted | 11 | 0.088 | 0.365 | 0.924 | 0.778 | 1.097 |
| ebi-a-GCST90016991 | Gut microbiota abundance (genus Eisenbergiella id.11304) | finn-b-N14_CALCUKIDUR | MR Egger | 11 | 0.681 | 0.879 | 1.112 | 0.293 | 4.223 |
| ebi-a-GCST90016991 | Gut microbiota abundance (genus Eisenbergiella id.11304) | finn-b-N14_CALCUKIDUR | Weighted median | 11 | 0.106 | 0.374 | 0.910 | 0.740 | 1.120 |
| ebi-a-GCST90016991 | Gut microbiota abundance (genus Eisenbergiella id.11304) | finn-b-N14_CALCUKIDUR | Maximum likelihood | 11 | 0.074 | 0.273 | 0.922 | 0.797 | 1.066 |
| ebi-a-GCST90016992 | Gut microbiota abundance (genus Enterorhabdus id.820) | finn-b-N14_CALCUKIDUR | Inverse variance weighted | 6 | 0.115 | 0.137 | 1.186 | 0.947 | 1.485 |
| ebi-a-GCST90016992 | Gut microbiota abundance (genus Enterorhabdus id.820) | finn-b-N14_CALCUKIDUR | MR Egger | 6 | 0.301 | 0.398 | 1.329 | 0.737 | 2.399 |
| ebi-a-GCST90016992 | Gut microbiota abundance (genus Enterorhabdus id.820) | finn-b-N14_CALCUKIDUR | Weighted median | 6 | 0.146 | 0.212 | 1.199 | 0.902 | 1.595 |
| ebi-a-GCST90016992 | Gut microbiota abundance (genus Enterorhabdus id.820) | finn-b-N14_CALCUKIDUR | Maximum likelihood | 6 | 0.116 | 0.139 | 1.187 | 0.946 | 1.490 |
| ebi-a-GCST90016993 | Gut microbiota abundance (genus Erysipelatoclostridium id.11381) | finn-b-N14_CALCUKIDUR | Inverse variance weighted | 15 | 0.080 | 0.787 | 1.022 | 0.873 | 1.196 |
| ebi-a-GCST90016993 | Gut microbiota abundance (genus Erysipelatoclostridium id.11381) | finn-b-N14_CALCUKIDUR | MR Egger | 15 | 0.315 | 0.115 | 0.588 | 0.317 | 1.089 |
| ebi-a-GCST90016993 | Gut microbiota abundance (genus Erysipelatoclostridium id.11381) | finn-b-N14_CALCUKIDUR | Weighted median | 15 | 0.109 | 0.829 | 1.024 | 0.826 | 1.269 |
| ebi-a-GCST90016993 | Gut microbiota abundance (genus Erysipelatoclostridium id.11381) | finn-b-N14_CALCUKIDUR | Maximum likelihood | 15 | 0.081 | 0.784 | 1.023 | 0.872 | 1.199 |
| ebi-a-GCST90016994 | Gut microbiota abundance (genus Erysipelotrichaceae UCG003 id.11384) | finn-b-N14_CALCUKIDUR | Wald ratio | 1 | 0.317 | 0.698 | 0.884 | 0.475 | 1.646 |
| ebi-a-GCST90016995 | Gut microbiota abundance (genus Escherichia Shigella id.3504) | finn-b-N14_CALCUKIDUR | Inverse variance weighted | 10 | 0.126 | 0.632 | 1.062 | 0.830 | 1.359 |
| ebi-a-GCST90016995 | Gut microbiota abundance (genus Escherichia Shigella id.3504) | finn-b-N14_CALCUKIDUR | MR Egger | 10 | 0.403 | 0.459 | 1.369 | 0.621 | 3.015 |
| ebi-a-GCST90016995 | Gut microbiota abundance (genus Escherichia Shigella id.3504) | finn-b-N14_CALCUKIDUR | Weighted median | 10 | 0.162 | 0.426 | 1.137 | 0.829 | 1.561 |
| ebi-a-GCST90016995 | Gut microbiota abundance (genus Escherichia Shigella id.3504) | finn-b-N14_CALCUKIDUR | Maximum likelihood | 10 | 0.120 | 0.592 | 1.066 | 0.843 | 1.348 |
| ebi-a-GCST90016996 | Gut microbiota abundance (genus Eubacterium brachy group id.11296) | finn-b-N14_CALCUKIDUR | Inverse variance weighted | 10 | 0.065 | 0.771 | 0.981 | 0.863 | 1.115 |
| ebi-a-GCST90016996 | Gut microbiota abundance (genus Eubacterium brachy group id.11296) | finn-b-N14_CALCUKIDUR | MR Egger | 10 | 0.262 | 0.598 | 0.866 | 0.518 | 1.448 |
| ebi-a-GCST90016996 | Gut microbiota abundance (genus Eubacterium brachy group id.11296) | finn-b-N14_CALCUKIDUR | Weighted median | 10 | 0.090 | 0.913 | 0.990 | 0.831 | 1.181 |
| ebi-a-GCST90016996 | Gut microbiota abundance (genus Eubacterium brachy group id.11296) | finn-b-N14_CALCUKIDUR | Maximum likelihood | 10 | 0.067 | 0.766 | 0.980 | 0.860 | 1.117 |
| ebi-a-GCST90016997 | Gut microbiota abundance (genus Eubacterium coprostanoligenes group id.11375) | finn-b-N14_CALCUKIDUR | Inverse variance weighted | 13 | 0.120 | 0.230 | 1.156 | 0.913 | 1.463 |
| ebi-a-GCST90016997 | Gut microbiota abundance (genus Eubacterium coprostanoligenes group id.11375) | finn-b-N14_CALCUKIDUR | MR Egger | 13 | 0.472 | 0.071 | 2.567 | 1.018 | 6.471 |
| ebi-a-GCST90016997 | Gut microbiota abundance (genus Eubacterium coprostanoligenes group id.11375) | finn-b-N14_CALCUKIDUR | Weighted median | 13 | 0.169 | 0.834 | 0.965 | 0.693 | 1.344 |
| ebi-a-GCST90016997 | Gut microbiota abundance (genus Eubacterium coprostanoligenes group id.11375) | finn-b-N14_CALCUKIDUR | Maximum likelihood | 13 | 0.123 | 0.229 | 1.159 | 0.911 | 1.475 |
| ebi-a-GCST90016998 | Gut microbiota abundance (genus Eubacterium eligens group id.14372) | finn-b-N14_CALCUKIDUR | Inverse variance weighted | 6 | 0.168 | 0.889 | 1.024 | 0.737 | 1.422 |
| ebi-a-GCST90016998 | Gut microbiota abundance (genus Eubacterium eligens group id.14372) | finn-b-N14_CALCUKIDUR | MR Egger | 6 | 0.666 | 0.583 | 0.672 | 0.182 | 2.481 |
| ebi-a-GCST90016998 | Gut microbiota abundance (genus Eubacterium eligens group id.14372) | finn-b-N14_CALCUKIDUR | Weighted median | 6 | 0.195 | 0.686 | 0.924 | 0.631 | 1.354 |
| ebi-a-GCST90016998 | Gut microbiota abundance (genus Eubacterium eligens group id.14372) | finn-b-N14_CALCUKIDUR | Maximum likelihood | 6 | 0.159 | 0.879 | 1.025 | 0.751 | 1.398 |
| ebi-a-GCST90016999 | Gut microbiota abundance (genus Eubacterium fissicatena group id.14373) | finn-b-N14_CALCUKIDUR | Inverse variance weighted | 9 | 0.069 | 0.685 | 0.972 | 0.849 | 1.114 |
| ebi-a-GCST90016999 | Gut microbiota abundance (genus Eubacterium fissicatena group id.14373) | finn-b-N14_CALCUKIDUR | MR Egger | 9 | 0.360 | 0.397 | 1.384 | 0.683 | 2.802 |
| ebi-a-GCST90016999 | Gut microbiota abundance (genus Eubacterium fissicatena group id.14373) | finn-b-N14_CALCUKIDUR | Weighted median | 9 | 0.087 | 0.715 | 0.969 | 0.816 | 1.150 |
| ebi-a-GCST90016999 | Gut microbiota abundance (genus Eubacterium fissicatena group id.14373) | finn-b-N14_CALCUKIDUR | Maximum likelihood | 9 | 0.070 | 0.685 | 0.972 | 0.847 | 1.115 |
| ebi-a-GCST90017000 | Gut microbiota abundance (genus Eubacterium hallii group id.11338) | finn-b-N14_CALCUKIDUR | Inverse variance weighted | 15 | 0.096 | 0.213 | 1.127 | 0.934 | 1.359 |
| ebi-a-GCST90017000 | Gut microbiota abundance (genus Eubacterium hallii group id.11338) | finn-b-N14_CALCUKIDUR | MR Egger | 15 | 0.195 | 0.124 | 1.377 | 0.939 | 2.018 |
| ebi-a-GCST90017000 | Gut microbiota abundance (genus Eubacterium hallii group id.11338) | finn-b-N14_CALCUKIDUR | Weighted median | 15 | 0.121 | 0.068 | 1.246 | 0.984 | 1.578 |
| ebi-a-GCST90017000 | Gut microbiota abundance (genus Eubacterium hallii group id.11338) | finn-b-N14_CALCUKIDUR | Maximum likelihood | 15 | 0.090 | 0.174 | 1.131 | 0.947 | 1.350 |
| ebi-a-GCST90017001 | Gut microbiota abundance (genus Eubacterium nodatum group id.11297) | finn-b-N14_CALCUKIDUR | Inverse variance weighted | 11 | 0.088 | 0.248 | 1.106 | 0.932 | 1.313 |
| ebi-a-GCST90017001 | Gut microbiota abundance (genus Eubacterium nodatum group id.11297) | finn-b-N14_CALCUKIDUR | MR Egger | 11 | 0.407 | 0.533 | 1.302 | 0.586 | 2.893 |
| ebi-a-GCST90017001 | Gut microbiota abundance (genus Eubacterium nodatum group id.11297) | finn-b-N14_CALCUKIDUR | Weighted median | 11 | 0.080 | 0.580 | 1.046 | 0.893 | 1.224 |
| ebi-a-GCST90017001 | Gut microbiota abundance (genus Eubacterium nodatum group id.11297) | finn-b-N14_CALCUKIDUR | Maximum likelihood | 11 | 0.060 | 0.064 | 1.117 | 0.993 | 1.256 |
| ebi-a-GCST90017002 | Gut microbiota abundance (genus Eubacterium oxidoreducens group id.11339) | finn-b-N14_CALCUKIDUR | Inverse variance weighted | 5 | 0.156 | 0.207 | 1.217 | 0.897 | 1.653 |
| ebi-a-GCST90017002 | Gut microbiota abundance (genus Eubacterium oxidoreducens group id.11339) | finn-b-N14_CALCUKIDUR | MR Egger | 5 | 0.644 | 0.878 | 0.898 | 0.254 | 3.174 |
| ebi-a-GCST90017002 | Gut microbiota abundance (genus Eubacterium oxidoreducens group id.11339) | finn-b-N14_CALCUKIDUR | Weighted median | 5 | 0.160 | 0.018 | 1.458 | 1.066 | 1.994 |
| ebi-a-GCST90017002 | Gut microbiota abundance (genus Eubacterium oxidoreducens group id.11339) | finn-b-N14_CALCUKIDUR | Maximum likelihood | 5 | 0.110 | 0.053 | 1.237 | 0.997 | 1.534 |
| ebi-a-GCST90017003 | Gut microbiota abundance (genus Eubacterium rectale group id.14374) | finn-b-N14_CALCUKIDUR | Inverse variance weighted | 8 | 0.208 | 0.901 | 1.026 | 0.683 | 1.541 |
| ebi-a-GCST90017003 | Gut microbiota abundance (genus Eubacterium rectale group id.14374) | finn-b-N14_CALCUKIDUR | MR Egger | 8 | 0.752 | 0.339 | 2.183 | 0.500 | 9.537 |
| ebi-a-GCST90017003 | Gut microbiota abundance (genus Eubacterium rectale group id.14374) | finn-b-N14_CALCUKIDUR | Weighted median | 8 | 0.195 | 0.263 | 0.803 | 0.548 | 1.178 |
| ebi-a-GCST90017003 | Gut microbiota abundance (genus Eubacterium rectale group id.14374) | finn-b-N14_CALCUKIDUR | Maximum likelihood | 8 | 0.151 | 0.851 | 1.029 | 0.766 | 1.383 |
| ebi-a-GCST90017004 | Gut microbiota abundance (genus Eubacterium ruminantium group id.11340) | finn-b-N14_CALCUKIDUR | Inverse variance weighted | 18 | 0.063 | 0.176 | 1.089 | 0.963 | 1.232 |
| ebi-a-GCST90017004 | Gut microbiota abundance (genus Eubacterium ruminantium group id.11340) | finn-b-N14_CALCUKIDUR | MR Egger | 18 | 0.213 | 0.892 | 1.030 | 0.678 | 1.563 |
| ebi-a-GCST90017004 | Gut microbiota abundance (genus Eubacterium ruminantium group id.11340) | finn-b-N14_CALCUKIDUR | Weighted median | 18 | 0.090 | 0.275 | 1.103 | 0.925 | 1.316 |
| ebi-a-GCST90017004 | Gut microbiota abundance (genus Eubacterium ruminantium group id.11340) | finn-b-N14_CALCUKIDUR | Maximum likelihood | 18 | 0.064 | 0.164 | 1.094 | 0.964 | 1.240 |
| ebi-a-GCST90017005 | Gut microbiota abundance (genus Eubacterium ventriosum group id.11341) | finn-b-N14_CALCUKIDUR | Inverse variance weighted | 15 | 0.101 | 0.219 | 1.132 | 0.929 | 1.380 |
| ebi-a-GCST90017005 | Gut microbiota abundance (genus Eubacterium ventriosum group id.11341) | finn-b-N14_CALCUKIDUR | MR Egger | 15 | 0.451 | 0.426 | 1.449 | 0.598 | 3.507 |
| ebi-a-GCST90017005 | Gut microbiota abundance (genus Eubacterium ventriosum group id.11341) | finn-b-N14_CALCUKIDUR | Weighted median | 15 | 0.129 | 0.302 | 1.143 | 0.887 | 1.473 |
| ebi-a-GCST90017005 | Gut microbiota abundance (genus Eubacterium ventriosum group id.11341) | finn-b-N14_CALCUKIDUR | Maximum likelihood | 15 | 0.102 | 0.207 | 1.138 | 0.931 | 1.389 |
| ebi-a-GCST90017006 | Gut microbiota abundance (genus Eubacterium xylanophilum group id.14375) | finn-b-N14_CALCUKIDUR | Inverse variance weighted | 9 | 0.112 | 0.042 | 1.256 | 1.008 | 1.566 |
| ebi-a-GCST90017006 | Gut microbiota abundance (genus Eubacterium xylanophilum group id.14375) | finn-b-N14_CALCUKIDUR | MR Egger | 9 | 0.356 | 0.776 | 1.111 | 0.552 | 2.234 |
| ebi-a-GCST90017006 | Gut microbiota abundance (genus Eubacterium xylanophilum group id.14375) | finn-b-N14_CALCUKIDUR | Weighted median | 9 | 0.153 | 0.274 | 1.182 | 0.876 | 1.594 |
| ebi-a-GCST90017006 | Gut microbiota abundance (genus Eubacterium xylanophilum group id.14375) | finn-b-N14_CALCUKIDUR | Maximum likelihood | 9 | 0.116 | 0.040 | 1.269 | 1.011 | 1.592 |
| ebi-a-GCST90017007 | Gut microbiota abundance (genus Faecalibacterium id.2057) | finn-b-N14_CALCUKIDUR | Inverse variance weighted | 9 | 0.129 | 0.362 | 1.125 | 0.874 | 1.448 |
| ebi-a-GCST90017007 | Gut microbiota abundance (genus Faecalibacterium id.2057) | finn-b-N14_CALCUKIDUR | MR Egger | 9 | 0.261 | 0.986 | 0.995 | 0.596 | 1.662 |
| ebi-a-GCST90017007 | Gut microbiota abundance (genus Faecalibacterium id.2057) | finn-b-N14_CALCUKIDUR | Weighted median | 9 | 0.146 | 0.224 | 1.194 | 0.897 | 1.590 |
| ebi-a-GCST90017007 | Gut microbiota abundance (genus Faecalibacterium id.2057) | finn-b-N14_CALCUKIDUR | Maximum likelihood | 9 | 0.105 | 0.242 | 1.131 | 0.920 | 1.389 |
| ebi-a-GCST90017008 | Gut microbiota abundance (genus Family XIII AD3011 group id.11293) | finn-b-N14_CALCUKIDUR | Inverse variance weighted | 13 | 0.121 | 0.557 | 0.931 | 0.735 | 1.181 |
| ebi-a-GCST90017008 | Gut microbiota abundance (genus Family XIII AD3011 group id.11293) | finn-b-N14_CALCUKIDUR | MR Egger | 13 | 0.577 | 0.352 | 0.571 | 0.184 | 1.769 |
| ebi-a-GCST90017008 | Gut microbiota abundance (genus Family XIII AD3011 group id.11293) | finn-b-N14_CALCUKIDUR | Weighted median | 13 | 0.148 | 0.843 | 0.971 | 0.727 | 1.297 |
| ebi-a-GCST90017008 | Gut microbiota abundance (genus Family XIII AD3011 group id.11293) | finn-b-N14_CALCUKIDUR | Maximum likelihood | 13 | 0.109 | 0.518 | 0.932 | 0.752 | 1.154 |
| ebi-a-GCST90017009 | Gut microbiota abundance (genus Family XIII UCG001 id.11294) | finn-b-N14_CALCUKIDUR | Inverse variance weighted | 8 | 0.123 | 0.835 | 1.026 | 0.807 | 1.305 |
| ebi-a-GCST90017009 | Gut microbiota abundance (genus Family XIII UCG001 id.11294) | finn-b-N14_CALCUKIDUR | MR Egger | 8 | 0.376 | 0.396 | 1.410 | 0.675 | 2.946 |
| ebi-a-GCST90017009 | Gut microbiota abundance (genus Family XIII UCG001 id.11294) | finn-b-N14_CALCUKIDUR | Weighted median | 8 | 0.152 | 0.809 | 1.038 | 0.770 | 1.398 |
| ebi-a-GCST90017009 | Gut microbiota abundance (genus Family XIII UCG001 id.11294) | finn-b-N14_CALCUKIDUR | Maximum likelihood | 8 | 0.124 | 0.833 | 1.027 | 0.805 | 1.310 |
| ebi-a-GCST90017010 | Gut microbiota abundance (genus Flavonifractor id.2059) | finn-b-N14_CALCUKIDUR | Inverse variance weighted | 5 | 0.144 | 0.018 | 0.711 | 0.536 | 0.944 |
| ebi-a-GCST90017010 | Gut microbiota abundance (genus Flavonifractor id.2059) | finn-b-N14_CALCUKIDUR | MR Egger | 5 | 0.576 | 0.211 | 0.401 | 0.130 | 1.241 |
| ebi-a-GCST90017010 | Gut microbiota abundance (genus Flavonifractor id.2059) | finn-b-N14_CALCUKIDUR | Weighted median | 5 | 0.193 | 0.098 | 0.727 | 0.498 | 1.061 |
| ebi-a-GCST90017010 | Gut microbiota abundance (genus Flavonifractor id.2059) | finn-b-N14_CALCUKIDUR | Maximum likelihood | 5 | 0.150 | 0.019 | 0.703 | 0.524 | 0.943 |
| ebi-a-GCST90017011 | Gut microbiota abundance (genus Fusicatenibacter id.11305) | finn-b-N14_CALCUKIDUR | Inverse variance weighted | 18 | 0.128 | 0.993 | 1.001 | 0.779 | 1.286 |
| ebi-a-GCST90017011 | Gut microbiota abundance (genus Fusicatenibacter id.11305) | finn-b-N14_CALCUKIDUR | MR Egger | 18 | 0.480 | 0.367 | 1.561 | 0.609 | 4.001 |
| ebi-a-GCST90017011 | Gut microbiota abundance (genus Fusicatenibacter id.11305) | finn-b-N14_CALCUKIDUR | Weighted median | 18 | 0.153 | 0.879 | 1.024 | 0.758 | 1.383 |
| ebi-a-GCST90017011 | Gut microbiota abundance (genus Fusicatenibacter id.11305) | finn-b-N14_CALCUKIDUR | Maximum likelihood | 18 | 0.104 | 0.991 | 1.001 | 0.817 | 1.227 |
| ebi-a-GCST90017012 | Gut microbiota abundance (genus Gordonibacter id.821) | finn-b-N14_CALCUKIDUR | Inverse variance weighted | 12 | 0.057 | 0.802 | 0.986 | 0.881 | 1.103 |
| ebi-a-GCST90017012 | Gut microbiota abundance (genus Gordonibacter id.821) | finn-b-N14_CALCUKIDUR | MR Egger | 12 | 0.247 | 0.667 | 1.116 | 0.688 | 1.809 |
| ebi-a-GCST90017012 | Gut microbiota abundance (genus Gordonibacter id.821) | finn-b-N14_CALCUKIDUR | Weighted median | 12 | 0.078 | 0.970 | 0.997 | 0.856 | 1.162 |
| ebi-a-GCST90017012 | Gut microbiota abundance (genus Gordonibacter id.821) | finn-b-N14_CALCUKIDUR | Maximum likelihood | 12 | 0.058 | 0.801 | 0.985 | 0.879 | 1.105 |
| ebi-a-GCST90017013 | Gut microbiota abundance (genus Haemophilus id.3698) | finn-b-N14_CALCUKIDUR | Inverse variance weighted | 9 | 0.090 | 0.406 | 1.078 | 0.903 | 1.287 |
| ebi-a-GCST90017013 | Gut microbiota abundance (genus Haemophilus id.3698) | finn-b-N14_CALCUKIDUR | MR Egger | 9 | 0.209 | 0.800 | 0.946 | 0.628 | 1.426 |
| ebi-a-GCST90017013 | Gut microbiota abundance (genus Haemophilus id.3698) | finn-b-N14_CALCUKIDUR | Weighted median | 9 | 0.112 | 0.676 | 1.048 | 0.841 | 1.305 |
| ebi-a-GCST90017013 | Gut microbiota abundance (genus Haemophilus id.3698) | finn-b-N14_CALCUKIDUR | Maximum likelihood | 9 | 0.089 | 0.383 | 1.081 | 0.908 | 1.287 |
| ebi-a-GCST90017014 | Gut microbiota abundance (genus Holdemanella id.11393) | finn-b-N14_CALCUKIDUR | Inverse variance weighted | 11 | 0.076 | 0.653 | 1.035 | 0.892 | 1.200 |
| ebi-a-GCST90017014 | Gut microbiota abundance (genus Holdemanella id.11393) | finn-b-N14_CALCUKIDUR | MR Egger | 11 | 0.217 | 0.037 | 1.698 | 1.110 | 2.597 |
| ebi-a-GCST90017014 | Gut microbiota abundance (genus Holdemanella id.11393) | finn-b-N14_CALCUKIDUR | Weighted median | 11 | 0.107 | 0.902 | 1.013 | 0.822 | 1.250 |
| ebi-a-GCST90017014 | Gut microbiota abundance (genus Holdemanella id.11393) | finn-b-N14_CALCUKIDUR | Maximum likelihood | 11 | 0.078 | 0.634 | 1.038 | 0.891 | 1.208 |
| ebi-a-GCST90017015 | Gut microbiota abundance (genus Holdemania id.2157) | finn-b-N14_CALCUKIDUR | Inverse variance weighted | 14 | 0.081 | 0.762 | 0.976 | 0.833 | 1.144 |
| ebi-a-GCST90017015 | Gut microbiota abundance (genus Holdemania id.2157) | finn-b-N14_CALCUKIDUR | MR Egger | 14 | 0.239 | 0.921 | 0.976 | 0.611 | 1.559 |
| ebi-a-GCST90017015 | Gut microbiota abundance (genus Holdemania id.2157) | finn-b-N14_CALCUKIDUR | Weighted median | 14 | 0.113 | 0.764 | 1.035 | 0.829 | 1.291 |
| ebi-a-GCST90017015 | Gut microbiota abundance (genus Holdemania id.2157) | finn-b-N14_CALCUKIDUR | Maximum likelihood | 14 | 0.083 | 0.760 | 0.975 | 0.829 | 1.147 |
| ebi-a-GCST90017016 | Gut microbiota abundance (genus Howardella id.2000) | finn-b-N14_CALCUKIDUR | Inverse variance weighted | 9 | 0.081 | 0.076 | 0.867 | 0.740 | 1.015 |
| ebi-a-GCST90017016 | Gut microbiota abundance (genus Howardella id.2000) | finn-b-N14_CALCUKIDUR | MR Egger | 9 | 0.339 | 0.964 | 0.984 | 0.507 | 1.913 |
| ebi-a-GCST90017016 | Gut microbiota abundance (genus Howardella id.2000) | finn-b-N14_CALCUKIDUR | Weighted median | 9 | 0.096 | 0.522 | 0.940 | 0.779 | 1.135 |
| ebi-a-GCST90017016 | Gut microbiota abundance (genus Howardella id.2000) | finn-b-N14_CALCUKIDUR | Maximum likelihood | 9 | 0.064 | 0.017 | 0.859 | 0.758 | 0.973 |
| ebi-a-GCST90017017 | Gut microbiota abundance (genus Hungatella id.11306) | finn-b-N14_CALCUKIDUR | Inverse variance weighted | 5 | 0.093 | 0.044 | 0.829 | 0.690 | 0.995 |
| ebi-a-GCST90017017 | Gut microbiota abundance (genus Hungatella id.11306) | finn-b-N14_CALCUKIDUR | MR Egger | 5 | 0.567 | 0.826 | 0.873 | 0.288 | 2.650 |
| ebi-a-GCST90017017 | Gut microbiota abundance (genus Hungatella id.11306) | finn-b-N14_CALCUKIDUR | Weighted median | 5 | 0.114 | 0.066 | 0.811 | 0.648 | 1.014 |
| ebi-a-GCST90017017 | Gut microbiota abundance (genus Hungatella id.11306) | finn-b-N14_CALCUKIDUR | Maximum likelihood | 5 | 0.096 | 0.048 | 0.828 | 0.686 | 0.999 |
| ebi-a-GCST90017018 | Gut microbiota abundance (genus Intestinibacter id.11345) | finn-b-N14_CALCUKIDUR | Inverse variance weighted | 15 | 0.111 | 0.732 | 0.963 | 0.774 | 1.197 |
| ebi-a-GCST90017018 | Gut microbiota abundance (genus Intestinibacter id.11345) | finn-b-N14_CALCUKIDUR | MR Egger | 15 | 0.363 | 0.436 | 1.339 | 0.657 | 2.729 |
| ebi-a-GCST90017018 | Gut microbiota abundance (genus Intestinibacter id.11345) | finn-b-N14_CALCUKIDUR | Weighted median | 15 | 0.139 | 0.416 | 1.119 | 0.853 | 1.469 |
| ebi-a-GCST90017018 | Gut microbiota abundance (genus Intestinibacter id.11345) | finn-b-N14_CALCUKIDUR | Maximum likelihood | 15 | 0.092 | 0.658 | 0.960 | 0.802 | 1.150 |
| ebi-a-GCST90017019 | Gut microbiota abundance (genus Intestinimonas id.2062) | finn-b-N14_CALCUKIDUR | Inverse variance weighted | 16 | 0.085 | 0.099 | 0.870 | 0.737 | 1.027 |
| ebi-a-GCST90017019 | Gut microbiota abundance (genus Intestinimonas id.2062) | finn-b-N14_CALCUKIDUR | MR Egger | 16 | 0.234 | 0.398 | 0.816 | 0.516 | 1.290 |
| ebi-a-GCST90017019 | Gut microbiota abundance (genus Intestinimonas id.2062) | finn-b-N14_CALCUKIDUR | Weighted median | 16 | 0.116 | 0.171 | 0.853 | 0.679 | 1.071 |
| ebi-a-GCST90017019 | Gut microbiota abundance (genus Intestinimonas id.2062) | finn-b-N14_CALCUKIDUR | Maximum likelihood | 16 | 0.086 | 0.105 | 0.869 | 0.734 | 1.030 |
| ebi-a-GCST90017020 | Gut microbiota abundance (genus Lachnoclostridium id.11308) | finn-b-N14_CALCUKIDUR | Inverse variance weighted | 13 | 0.118 | 0.115 | 0.829 | 0.658 | 1.046 |
| ebi-a-GCST90017020 | Gut microbiota abundance (genus Lachnoclostridium id.11308) | finn-b-N14_CALCUKIDUR | MR Egger | 13 | 0.421 | 0.644 | 0.819 | 0.359 | 1.869 |
| ebi-a-GCST90017020 | Gut microbiota abundance (genus Lachnoclostridium id.11308) | finn-b-N14_CALCUKIDUR | Weighted median | 13 | 0.164 | 0.413 | 0.874 | 0.633 | 1.207 |
| ebi-a-GCST90017020 | Gut microbiota abundance (genus Lachnoclostridium id.11308) | finn-b-N14_CALCUKIDUR | Maximum likelihood | 13 | 0.118 | 0.117 | 0.832 | 0.660 | 1.047 |
| ebi-a-GCST90017021 | Gut microbiota abundance (genus Lachnospiraceae FCS020 group id.11314) | finn-b-N14_CALCUKIDUR | Inverse variance weighted | 12 | 0.114 | 0.683 | 1.047 | 0.838 | 1.309 |
| ebi-a-GCST90017021 | Gut microbiota abundance (genus Lachnospiraceae FCS020 group id.11314) | finn-b-N14_CALCUKIDUR | MR Egger | 12 | 0.269 | 0.065 | 1.746 | 1.030 | 2.960 |
| ebi-a-GCST90017021 | Gut microbiota abundance (genus Lachnospiraceae FCS020 group id.11314) | finn-b-N14_CALCUKIDUR | Weighted median | 12 | 0.143 | 0.725 | 0.951 | 0.718 | 1.259 |
| ebi-a-GCST90017021 | Gut microbiota abundance (genus Lachnospiraceae FCS020 group id.11314) | finn-b-N14_CALCUKIDUR | Maximum likelihood | 12 | 0.106 | 0.628 | 1.052 | 0.856 | 1.295 |
| ebi-a-GCST90017022 | Gut microbiota abundance (genus Lachnospiraceae NC2004 group id.11316) | finn-b-N14_CALCUKIDUR | Inverse variance weighted | 9 | 0.105 | 0.894 | 0.986 | 0.803 | 1.211 |
| ebi-a-GCST90017022 | Gut microbiota abundance (genus Lachnospiraceae NC2004 group id.11316) | finn-b-N14_CALCUKIDUR | MR Egger | 9 | 0.357 | 0.087 | 2.033 | 1.009 | 4.097 |
| ebi-a-GCST90017022 | Gut microbiota abundance (genus Lachnospiraceae NC2004 group id.11316) | finn-b-N14_CALCUKIDUR | Weighted median | 9 | 0.116 | 0.461 | 1.089 | 0.867 | 1.368 |
| ebi-a-GCST90017022 | Gut microbiota abundance (genus Lachnospiraceae NC2004 group id.11316) | finn-b-N14_CALCUKIDUR | Maximum likelihood | 9 | 0.087 | 0.867 | 0.985 | 0.831 | 1.169 |
| ebi-a-GCST90017023 | Gut microbiota abundance (genus Lachnospiraceae ND3007 group id.11317) | finn-b-N14_CALCUKIDUR | Inverse variance weighted | 3 | 0.238 | 0.427 | 0.828 | 0.519 | 1.320 |
| ebi-a-GCST90017023 | Gut microbiota abundance (genus Lachnospiraceae ND3007 group id.11317) | finn-b-N14_CALCUKIDUR | MR Egger | 3 | 4.007 | 0.995 | 1.032 | 0.000 | 2656.021 |
| ebi-a-GCST90017023 | Gut microbiota abundance (genus Lachnospiraceae ND3007 group id.11317) | finn-b-N14_CALCUKIDUR | Weighted median | 3 | 0.286 | 0.538 | 0.839 | 0.479 | 1.468 |
| ebi-a-GCST90017023 | Gut microbiota abundance (genus Lachnospiraceae ND3007 group id.11317) | finn-b-N14_CALCUKIDUR | Maximum likelihood | 3 | 0.239 | 0.430 | 0.828 | 0.518 | 1.323 |
| ebi-a-GCST90017024 | Gut microbiota abundance (genus Lachnospiraceae NK4A136 group id.11319) | finn-b-N14_CALCUKIDUR | Inverse variance weighted | 15 | 0.090 | 0.613 | 1.047 | 0.877 | 1.249 |
| ebi-a-GCST90017024 | Gut microbiota abundance (genus Lachnospiraceae NK4A136 group id.11319) | finn-b-N14_CALCUKIDUR | MR Egger | 15 | 0.180 | 0.963 | 1.009 | 0.708 | 1.436 |
| ebi-a-GCST90017024 | Gut microbiota abundance (genus Lachnospiraceae NK4A136 group id.11319) | finn-b-N14_CALCUKIDUR | Weighted median | 15 | 0.136 | 0.719 | 1.050 | 0.804 | 1.372 |
| ebi-a-GCST90017024 | Gut microbiota abundance (genus Lachnospiraceae NK4A136 group id.11319) | finn-b-N14_CALCUKIDUR | Maximum likelihood | 15 | 0.092 | 0.603 | 1.049 | 0.876 | 1.255 |
| ebi-a-GCST90017025 | Gut microbiota abundance (genus Lachnospiraceae UCG001 id.11321) | finn-b-N14_CALCUKIDUR | Inverse variance weighted | 13 | 0.103 | 0.473 | 0.929 | 0.759 | 1.137 |
| ebi-a-GCST90017025 | Gut microbiota abundance (genus Lachnospiraceae UCG001 id.11321) | finn-b-N14_CALCUKIDUR | MR Egger | 13 | 0.466 | 0.954 | 0.973 | 0.390 | 2.425 |
| ebi-a-GCST90017025 | Gut microbiota abundance (genus Lachnospiraceae UCG001 id.11321) | finn-b-N14_CALCUKIDUR | Weighted median | 13 | 0.123 | 0.749 | 0.961 | 0.756 | 1.223 |
| ebi-a-GCST90017025 | Gut microbiota abundance (genus Lachnospiraceae UCG001 id.11321) | finn-b-N14_CALCUKIDUR | Maximum likelihood | 13 | 0.087 | 0.385 | 0.927 | 0.782 | 1.100 |
| ebi-a-GCST90017026 | Gut microbiota abundance (genus Lachnospiraceae UCG004 id.11324) | finn-b-N14_CALCUKIDUR | Inverse variance weighted | 12 | 0.114 | 0.469 | 0.921 | 0.737 | 1.151 |
| ebi-a-GCST90017026 | Gut microbiota abundance (genus Lachnospiraceae UCG004 id.11324) | finn-b-N14_CALCUKIDUR | MR Egger | 12 | 0.472 | 0.492 | 1.401 | 0.555 | 3.533 |
| ebi-a-GCST90017026 | Gut microbiota abundance (genus Lachnospiraceae UCG004 id.11324) | finn-b-N14_CALCUKIDUR | Weighted median | 12 | 0.156 | 0.975 | 1.005 | 0.741 | 1.363 |
| ebi-a-GCST90017026 | Gut microbiota abundance (genus Lachnospiraceae UCG004 id.11324) | finn-b-N14_CALCUKIDUR | Maximum likelihood | 12 | 0.116 | 0.467 | 0.919 | 0.733 | 1.153 |
| ebi-a-GCST90017027 | Gut microbiota abundance (genus Lachnospiraceae UCG008 id.11328) | finn-b-N14_CALCUKIDUR | Inverse variance weighted | 11 | 0.077 | 0.107 | 0.883 | 0.759 | 1.027 |
| ebi-a-GCST90017027 | Gut microbiota abundance (genus Lachnospiraceae UCG008 id.11328) | finn-b-N14_CALCUKIDUR | MR Egger | 11 | 0.401 | 0.669 | 1.194 | 0.544 | 2.620 |
| ebi-a-GCST90017027 | Gut microbiota abundance (genus Lachnospiraceae UCG008 id.11328) | finn-b-N14_CALCUKIDUR | Weighted median | 11 | 0.110 | 0.105 | 0.836 | 0.674 | 1.038 |
| ebi-a-GCST90017027 | Gut microbiota abundance (genus Lachnospiraceae UCG008 id.11328) | finn-b-N14_CALCUKIDUR | Maximum likelihood | 11 | 0.079 | 0.119 | 0.884 | 0.757 | 1.032 |
| ebi-a-GCST90017028 | Gut microbiota abundance (genus Lachnospiraceae UCG010 id.11330) | finn-b-N14_CALCUKIDUR | Inverse variance weighted | 10 | 0.119 | 0.376 | 0.900 | 0.713 | 1.136 |
| ebi-a-GCST90017028 | Gut microbiota abundance (genus Lachnospiraceae UCG010 id.11330) | finn-b-N14_CALCUKIDUR | MR Egger | 10 | 0.362 | 0.319 | 0.681 | 0.335 | 1.384 |
| ebi-a-GCST90017028 | Gut microbiota abundance (genus Lachnospiraceae UCG010 id.11330) | finn-b-N14_CALCUKIDUR | Weighted median | 10 | 0.153 | 0.938 | 1.012 | 0.749 | 1.367 |
| ebi-a-GCST90017028 | Gut microbiota abundance (genus Lachnospiraceae UCG010 id.11330) | finn-b-N14_CALCUKIDUR | Maximum likelihood | 10 | 0.121 | 0.384 | 0.900 | 0.710 | 1.141 |
| ebi-a-GCST90017029 | Gut microbiota abundance (genus Lachnospira id.2004) | finn-b-N14_CALCUKIDUR | Wald ratio | 1 | 0.425 | 0.200 | 1.723 | 0.750 | 3.960 |
| ebi-a-GCST90017030 | Gut microbiota abundance (genus Lactobacillus id.1837) | finn-b-N14_CALCUKIDUR | Inverse variance weighted | 9 | 0.078 | 0.775 | 1.023 | 0.877 | 1.192 |
| ebi-a-GCST90017030 | Gut microbiota abundance (genus Lactobacillus id.1837) | finn-b-N14_CALCUKIDUR | MR Egger | 9 | 0.207 | 0.919 | 0.978 | 0.653 | 1.467 |
| ebi-a-GCST90017030 | Gut microbiota abundance (genus Lactobacillus id.1837) | finn-b-N14_CALCUKIDUR | Weighted median | 9 | 0.104 | 0.653 | 1.048 | 0.855 | 1.285 |
| ebi-a-GCST90017030 | Gut microbiota abundance (genus Lactobacillus id.1837) | finn-b-N14_CALCUKIDUR | Maximum likelihood | 9 | 0.079 | 0.773 | 1.023 | 0.876 | 1.195 |
| ebi-a-GCST90017031 | Gut microbiota abundance (genus Lactococcus id.1851) | finn-b-N14_CALCUKIDUR | Inverse variance weighted | 9 | 0.084 | 0.254 | 0.909 | 0.771 | 1.071 |
| ebi-a-GCST90017031 | Gut microbiota abundance (genus Lactococcus id.1851) | finn-b-N14_CALCUKIDUR | MR Egger | 9 | 0.395 | 0.483 | 0.747 | 0.344 | 1.618 |
| ebi-a-GCST90017031 | Gut microbiota abundance (genus Lactococcus id.1851) | finn-b-N14_CALCUKIDUR | Weighted median | 9 | 0.097 | 0.063 | 0.835 | 0.690 | 1.010 |
| ebi-a-GCST90017031 | Gut microbiota abundance (genus Lactococcus id.1851) | finn-b-N14_CALCUKIDUR | Maximum likelihood | 9 | 0.072 | 0.160 | 0.904 | 0.785 | 1.041 |
| ebi-a-GCST90017032 | Gut microbiota abundance (genus Marvinbryantia id.2005) | finn-b-N14_CALCUKIDUR | Inverse variance weighted | 10 | 0.114 | 0.604 | 1.061 | 0.849 | 1.325 |
| ebi-a-GCST90017032 | Gut microbiota abundance (genus Marvinbryantia id.2005) | finn-b-N14_CALCUKIDUR | MR Egger | 10 | 0.445 | 0.565 | 1.306 | 0.546 | 3.124 |
| ebi-a-GCST90017032 | Gut microbiota abundance (genus Marvinbryantia id.2005) | finn-b-N14_CALCUKIDUR | Weighted median | 10 | 0.148 | 0.858 | 1.027 | 0.768 | 1.373 |
| ebi-a-GCST90017032 | Gut microbiota abundance (genus Marvinbryantia id.2005) | finn-b-N14_CALCUKIDUR | Maximum likelihood | 10 | 0.115 | 0.599 | 1.062 | 0.848 | 1.331 |
| ebi-a-GCST90017033 | Gut microbiota abundance (genus Methanobrevibacter id.123) | finn-b-N14_CALCUKIDUR | Inverse variance weighted | 6 | 0.084 | 0.479 | 0.942 | 0.799 | 1.111 |
| ebi-a-GCST90017033 | Gut microbiota abundance (genus Methanobrevibacter id.123) | finn-b-N14_CALCUKIDUR | MR Egger | 6 | 0.315 | 0.252 | 1.524 | 0.822 | 2.827 |
| ebi-a-GCST90017033 | Gut microbiota abundance (genus Methanobrevibacter id.123) | finn-b-N14_CALCUKIDUR | Weighted median | 6 | 0.114 | 0.845 | 1.023 | 0.817 | 1.279 |
| ebi-a-GCST90017033 | Gut microbiota abundance (genus Methanobrevibacter id.123) | finn-b-N14_CALCUKIDUR | Maximum likelihood | 6 | 0.086 | 0.473 | 0.940 | 0.795 | 1.113 |
| ebi-a-GCST90017034 | Gut microbiota abundance (genus Odoribacter id.952) | finn-b-N14_CALCUKIDUR | Inverse variance weighted | 7 | 0.174 | 0.897 | 1.023 | 0.727 | 1.439 |
| ebi-a-GCST90017034 | Gut microbiota abundance (genus Odoribacter id.952) | finn-b-N14_CALCUKIDUR | MR Egger | 7 | 0.593 | 0.806 | 0.858 | 0.268 | 2.742 |
| ebi-a-GCST90017034 | Gut microbiota abundance (genus Odoribacter id.952) | finn-b-N14_CALCUKIDUR | Weighted median | 7 | 0.201 | 0.704 | 0.927 | 0.625 | 1.373 |
| ebi-a-GCST90017034 | Gut microbiota abundance (genus Odoribacter id.952) | finn-b-N14_CALCUKIDUR | Maximum likelihood | 7 | 0.146 | 0.864 | 1.025 | 0.770 | 1.365 |
| ebi-a-GCST90017035 | Gut microbiota abundance (genus Olsenella id.822) | finn-b-N14_CALCUKIDUR | Inverse variance weighted | 10 | 0.084 | 0.503 | 0.945 | 0.802 | 1.114 |
| ebi-a-GCST90017035 | Gut microbiota abundance (genus Olsenella id.822) | finn-b-N14_CALCUKIDUR | MR Egger | 10 | 0.204 | 0.047 | 1.615 | 1.082 | 2.411 |
| ebi-a-GCST90017035 | Gut microbiota abundance (genus Olsenella id.822) | finn-b-N14_CALCUKIDUR | Weighted median | 10 | 0.085 | 0.487 | 0.943 | 0.798 | 1.114 |
| ebi-a-GCST90017035 | Gut microbiota abundance (genus Olsenella id.822) | finn-b-N14_CALCUKIDUR | Maximum likelihood | 10 | 0.064 | 0.325 | 0.939 | 0.827 | 1.065 |
| ebi-a-GCST90017036 | Gut microbiota abundance (genus Oscillibacter id.2063) | finn-b-N14_CALCUKIDUR | Inverse variance weighted | 13 | 0.081 | 0.855 | 0.985 | 0.840 | 1.156 |
| ebi-a-GCST90017036 | Gut microbiota abundance (genus Oscillibacter id.2063) | finn-b-N14_CALCUKIDUR | MR Egger | 13 | 0.308 | 0.362 | 1.341 | 0.733 | 2.453 |
| ebi-a-GCST90017036 | Gut microbiota abundance (genus Oscillibacter id.2063) | finn-b-N14_CALCUKIDUR | Weighted median | 13 | 0.108 | 0.339 | 1.109 | 0.897 | 1.372 |
| ebi-a-GCST90017036 | Gut microbiota abundance (genus Oscillibacter id.2063) | finn-b-N14_CALCUKIDUR | Maximum likelihood | 13 | 0.083 | 0.854 | 0.985 | 0.838 | 1.158 |
| ebi-a-GCST90017037 | Gut microbiota abundance (genus Oscillospira id.2064) | finn-b-N14_CALCUKIDUR | Inverse variance weighted | 8 | 0.139 | 0.046 | 0.758 | 0.577 | 0.996 |
| ebi-a-GCST90017037 | Gut microbiota abundance (genus Oscillospira id.2064) | finn-b-N14_CALCUKIDUR | MR Egger | 8 | 0.628 | 0.466 | 0.613 | 0.179 | 2.100 |
| ebi-a-GCST90017037 | Gut microbiota abundance (genus Oscillospira id.2064) | finn-b-N14_CALCUKIDUR | Weighted median | 8 | 0.150 | 0.046 | 0.742 | 0.553 | 0.995 |
| ebi-a-GCST90017037 | Gut microbiota abundance (genus Oscillospira id.2064) | finn-b-N14_CALCUKIDUR | Maximum likelihood | 8 | 0.117 | 0.012 | 0.745 | 0.593 | 0.936 |
| ebi-a-GCST90017038 | Gut microbiota abundance (genus Oxalobacter id.2978) | finn-b-N14_CALCUKIDUR | Inverse variance weighted | 11 | 0.062 | 0.384 | 0.947 | 0.838 | 1.070 |
| ebi-a-GCST90017038 | Gut microbiota abundance (genus Oxalobacter id.2978) | finn-b-N14_CALCUKIDUR | MR Egger | 11 | 0.293 | 0.257 | 0.702 | 0.396 | 1.246 |
| ebi-a-GCST90017038 | Gut microbiota abundance (genus Oxalobacter id.2978) | finn-b-N14_CALCUKIDUR | Weighted median | 11 | 0.082 | 0.338 | 0.924 | 0.787 | 1.086 |
| ebi-a-GCST90017038 | Gut microbiota abundance (genus Oxalobacter id.2978) | finn-b-N14_CALCUKIDUR | Maximum likelihood | 11 | 0.063 | 0.384 | 0.947 | 0.837 | 1.071 |
| ebi-a-GCST90017039 | Gut microbiota abundance (genus Parabacteroides id.954) | finn-b-N14_CALCUKIDUR | Inverse variance weighted | 5 | 0.189 | 0.978 | 0.995 | 0.687 | 1.440 |
| ebi-a-GCST90017039 | Gut microbiota abundance (genus Parabacteroides id.954) | finn-b-N14_CALCUKIDUR | MR Egger | 5 | 1.093 | 0.149 | 0.121 | 0.014 | 1.029 |
| ebi-a-GCST90017039 | Gut microbiota abundance (genus Parabacteroides id.954) | finn-b-N14_CALCUKIDUR | Weighted median | 5 | 0.227 | 0.756 | 0.932 | 0.597 | 1.454 |
| ebi-a-GCST90017039 | Gut microbiota abundance (genus Parabacteroides id.954) | finn-b-N14_CALCUKIDUR | Maximum likelihood | 5 | 0.179 | 0.976 | 0.995 | 0.700 | 1.413 |
| ebi-a-GCST90017040 | Gut microbiota abundance (genus Paraprevotella id.962) | finn-b-N14_CALCUKIDUR | Inverse variance weighted | 13 | 0.069 | 0.479 | 0.952 | 0.831 | 1.091 |
| ebi-a-GCST90017040 | Gut microbiota abundance (genus Paraprevotella id.962) | finn-b-N14_CALCUKIDUR | MR Egger | 13 | 0.264 | 0.525 | 0.841 | 0.502 | 1.410 |
| ebi-a-GCST90017040 | Gut microbiota abundance (genus Paraprevotella id.962) | finn-b-N14_CALCUKIDUR | Weighted median | 13 | 0.091 | 0.230 | 0.897 | 0.750 | 1.072 |
| ebi-a-GCST90017040 | Gut microbiota abundance (genus Paraprevotella id.962) | finn-b-N14_CALCUKIDUR | Maximum likelihood | 13 | 0.070 | 0.475 | 0.951 | 0.829 | 1.091 |
| ebi-a-GCST90017041 | Gut microbiota abundance (genus Parasutterella id.2892) | finn-b-N14_CALCUKIDUR | Inverse variance weighted | 14 | 0.083 | 0.445 | 1.065 | 0.906 | 1.253 |
| ebi-a-GCST90017041 | Gut microbiota abundance (genus Parasutterella id.2892) | finn-b-N14_CALCUKIDUR | MR Egger | 14 | 0.230 | 0.706 | 0.915 | 0.583 | 1.435 |
| ebi-a-GCST90017041 | Gut microbiota abundance (genus Parasutterella id.2892) | finn-b-N14_CALCUKIDUR | Weighted median | 14 | 0.112 | 0.724 | 0.961 | 0.772 | 1.197 |
| ebi-a-GCST90017041 | Gut microbiota abundance (genus Parasutterella id.2892) | finn-b-N14_CALCUKIDUR | Maximum likelihood | 14 | 0.084 | 0.434 | 1.068 | 0.905 | 1.260 |
| ebi-a-GCST90017042 | Gut microbiota abundance (genus Peptococcus id.2037) | finn-b-N14_CALCUKIDUR | Inverse variance weighted | 12 | 0.066 | 0.906 | 1.008 | 0.885 | 1.147 |
| ebi-a-GCST90017042 | Gut microbiota abundance (genus Peptococcus id.2037) | finn-b-N14_CALCUKIDUR | MR Egger | 12 | 0.254 | 0.406 | 1.247 | 0.758 | 2.050 |
| ebi-a-GCST90017042 | Gut microbiota abundance (genus Peptococcus id.2037) | finn-b-N14_CALCUKIDUR | Weighted median | 12 | 0.086 | 0.584 | 1.048 | 0.886 | 1.241 |
| ebi-a-GCST90017042 | Gut microbiota abundance (genus Peptococcus id.2037) | finn-b-N14_CALCUKIDUR | Maximum likelihood | 12 | 0.067 | 0.905 | 1.008 | 0.884 | 1.149 |
| ebi-a-GCST90017043 | Gut microbiota abundance (genus Phascolarctobacterium id.2168) | finn-b-N14_CALCUKIDUR | Inverse variance weighted | 8 | 0.127 | 0.132 | 0.826 | 0.644 | 1.059 |
| ebi-a-GCST90017043 | Gut microbiota abundance (genus Phascolarctobacterium id.2168) | finn-b-N14_CALCUKIDUR | MR Egger | 8 | 0.603 | 0.196 | 0.416 | 0.127 | 1.355 |
| ebi-a-GCST90017043 | Gut microbiota abundance (genus Phascolarctobacterium id.2168) | finn-b-N14_CALCUKIDUR | Weighted median | 8 | 0.161 | 0.477 | 0.892 | 0.650 | 1.223 |
| ebi-a-GCST90017043 | Gut microbiota abundance (genus Phascolarctobacterium id.2168) | finn-b-N14_CALCUKIDUR | Maximum likelihood | 8 | 0.120 | 0.108 | 0.824 | 0.651 | 1.043 |
| ebi-a-GCST90017044 | Gut microbiota abundance (genus Prevotella7 id.11182) | finn-b-N14_CALCUKIDUR | Inverse variance weighted | 11 | 0.063 | 0.857 | 1.011 | 0.895 | 1.143 |
| ebi-a-GCST90017044 | Gut microbiota abundance (genus Prevotella7 id.11182) | finn-b-N14_CALCUKIDUR | MR Egger | 11 | 0.383 | 0.913 | 1.044 | 0.493 | 2.210 |
| ebi-a-GCST90017044 | Gut microbiota abundance (genus Prevotella7 id.11182) | finn-b-N14_CALCUKIDUR | Weighted median | 11 | 0.083 | 0.501 | 1.057 | 0.899 | 1.243 |
| ebi-a-GCST90017044 | Gut microbiota abundance (genus Prevotella7 id.11182) | finn-b-N14_CALCUKIDUR | Maximum likelihood | 11 | 0.060 | 0.845 | 1.012 | 0.899 | 1.139 |
| ebi-a-GCST90017045 | Gut microbiota abundance (genus Prevotella9 id.11183) | finn-b-N14_CALCUKIDUR | Inverse variance weighted | 15 | 0.080 | 0.679 | 0.968 | 0.828 | 1.131 |
| ebi-a-GCST90017045 | Gut microbiota abundance (genus Prevotella9 id.11183) | finn-b-N14_CALCUKIDUR | MR Egger | 15 | 0.242 | 0.884 | 0.965 | 0.601 | 1.549 |
| ebi-a-GCST90017045 | Gut microbiota abundance (genus Prevotella9 id.11183) | finn-b-N14_CALCUKIDUR | Weighted median | 15 | 0.106 | 0.527 | 0.935 | 0.759 | 1.151 |
| ebi-a-GCST90017045 | Gut microbiota abundance (genus Prevotella9 id.11183) | finn-b-N14_CALCUKIDUR | Maximum likelihood | 15 | 0.078 | 0.662 | 0.967 | 0.830 | 1.126 |
| ebi-a-GCST90017046 | Gut microbiota abundance (genus Rikenellaceae RC9 gut group id.11191) | finn-b-N14_CALCUKIDUR | Inverse variance weighted | 11 | 0.063 | 0.607 | 0.968 | 0.856 | 1.095 |
| ebi-a-GCST90017046 | Gut microbiota abundance (genus Rikenellaceae RC9 gut group id.11191) | finn-b-N14_CALCUKIDUR | MR Egger | 11 | 0.375 | 0.181 | 0.581 | 0.278 | 1.211 |
| ebi-a-GCST90017046 | Gut microbiota abundance (genus Rikenellaceae RC9 gut group id.11191) | finn-b-N14_CALCUKIDUR | Weighted median | 11 | 0.080 | 0.635 | 0.963 | 0.822 | 1.127 |
| ebi-a-GCST90017046 | Gut microbiota abundance (genus Rikenellaceae RC9 gut group id.11191) | finn-b-N14_CALCUKIDUR | Maximum likelihood | 11 | 0.058 | 0.560 | 0.967 | 0.862 | 1.084 |
| ebi-a-GCST90017047 | Gut microbiota abundance (genus Romboutsia id.11347) | finn-b-N14_CALCUKIDUR | Inverse variance weighted | 13 | 0.147 | 0.980 | 1.004 | 0.753 | 1.339 |
| ebi-a-GCST90017047 | Gut microbiota abundance (genus Romboutsia id.11347) | finn-b-N14_CALCUKIDUR | MR Egger | 13 | 0.441 | 0.715 | 0.848 | 0.357 | 2.013 |
| ebi-a-GCST90017047 | Gut microbiota abundance (genus Romboutsia id.11347) | finn-b-N14_CALCUKIDUR | Weighted median | 13 | 0.155 | 0.815 | 1.037 | 0.765 | 1.405 |
| ebi-a-GCST90017047 | Gut microbiota abundance (genus Romboutsia id.11347) | finn-b-N14_CALCUKIDUR | Maximum likelihood | 13 | 0.107 | 0.969 | 1.004 | 0.814 | 1.239 |
| ebi-a-GCST90017048 | Gut microbiota abundance (genus Roseburia id.2012) | finn-b-N14_CALCUKIDUR | Inverse variance weighted | 13 | 0.116 | 0.712 | 0.958 | 0.763 | 1.203 |
| ebi-a-GCST90017048 | Gut microbiota abundance (genus Roseburia id.2012) | finn-b-N14_CALCUKIDUR | MR Egger | 13 | 0.349 | 0.685 | 1.156 | 0.583 | 2.294 |
| ebi-a-GCST90017048 | Gut microbiota abundance (genus Roseburia id.2012) | finn-b-N14_CALCUKIDUR | Weighted median | 13 | 0.149 | 0.783 | 0.960 | 0.716 | 1.286 |
| ebi-a-GCST90017048 | Gut microbiota abundance (genus Roseburia id.2012) | finn-b-N14_CALCUKIDUR | Maximum likelihood | 13 | 0.118 | 0.711 | 0.957 | 0.760 | 1.205 |
| ebi-a-GCST90017049 | Gut microbiota abundance (genus Ruminiclostridium5 id.11355) | finn-b-N14_CALCUKIDUR | Inverse variance weighted | 11 | 0.184 | 0.589 | 1.105 | 0.770 | 1.585 |
| ebi-a-GCST90017049 | Gut microbiota abundance (genus Ruminiclostridium5 id.11355) | finn-b-N14_CALCUKIDUR | MR Egger | 11 | 0.794 | 0.636 | 0.678 | 0.143 | 3.212 |
| ebi-a-GCST90017049 | Gut microbiota abundance (genus Ruminiclostridium5 id.11355) | finn-b-N14_CALCUKIDUR | Weighted median | 11 | 0.189 | 0.660 | 1.087 | 0.750 | 1.573 |
| ebi-a-GCST90017049 | Gut microbiota abundance (genus Ruminiclostridium5 id.11355) | finn-b-N14_CALCUKIDUR | Maximum likelihood | 11 | 0.133 | 0.406 | 1.117 | 0.860 | 1.451 |
| ebi-a-GCST90017050 | Gut microbiota abundance (genus Ruminiclostridium6 id.11356) | finn-b-N14_CALCUKIDUR | Inverse variance weighted | 15 | 0.097 | 0.914 | 1.011 | 0.836 | 1.222 |
| ebi-a-GCST90017050 | Gut microbiota abundance (genus Ruminiclostridium6 id.11356) | finn-b-N14_CALCUKIDUR | MR Egger | 15 | 0.241 | 0.452 | 0.830 | 0.517 | 1.331 |
| ebi-a-GCST90017050 | Gut microbiota abundance (genus Ruminiclostridium6 id.11356) | finn-b-N14_CALCUKIDUR | Weighted median | 15 | 0.142 | 0.331 | 1.148 | 0.869 | 1.516 |
| ebi-a-GCST90017050 | Gut microbiota abundance (genus Ruminiclostridium6 id.11356) | finn-b-N14_CALCUKIDUR | Maximum likelihood | 15 | 0.099 | 0.913 | 1.011 | 0.833 | 1.227 |
| ebi-a-GCST90017051 | Gut microbiota abundance (genus Ruminiclostridium9 id.11357) | finn-b-N14_CALCUKIDUR | Inverse variance weighted | 8 | 0.149 | 0.852 | 1.028 | 0.767 | 1.378 |
| ebi-a-GCST90017051 | Gut microbiota abundance (genus Ruminiclostridium9 id.11357) | finn-b-N14_CALCUKIDUR | MR Egger | 8 | 0.716 | 0.206 | 0.362 | 0.089 | 1.474 |
| ebi-a-GCST90017051 | Gut microbiota abundance (genus Ruminiclostridium9 id.11357) | finn-b-N14_CALCUKIDUR | Weighted median | 8 | 0.187 | 0.750 | 1.061 | 0.735 | 1.532 |
| ebi-a-GCST90017051 | Gut microbiota abundance (genus Ruminiclostridium9 id.11357) | finn-b-N14_CALCUKIDUR | Maximum likelihood | 8 | 0.151 | 0.850 | 1.029 | 0.765 | 1.384 |
| ebi-a-GCST90017052 | Gut microbiota abundance (genus Ruminococcaceae NK4A214 group id.11358) | finn-b-N14_CALCUKIDUR | Inverse variance weighted | 13 | 0.106 | 0.800 | 1.027 | 0.834 | 1.265 |
| ebi-a-GCST90017052 | Gut microbiota abundance (genus Ruminococcaceae NK4A214 group id.11358) | finn-b-N14_CALCUKIDUR | MR Egger | 13 | 0.364 | 0.770 | 0.897 | 0.439 | 1.830 |
| ebi-a-GCST90017052 | Gut microbiota abundance (genus Ruminococcaceae NK4A214 group id.11358) | finn-b-N14_CALCUKIDUR | Weighted median | 13 | 0.152 | 0.666 | 1.068 | 0.793 | 1.437 |
| ebi-a-GCST90017052 | Gut microbiota abundance (genus Ruminococcaceae NK4A214 group id.11358) | finn-b-N14_CALCUKIDUR | Maximum likelihood | 13 | 0.109 | 0.792 | 1.029 | 0.832 | 1.273 |
| ebi-a-GCST90017053 | Gut microbiota abundance (genus Ruminococcaceae UCG002 id.11360) | finn-b-N14_CALCUKIDUR | Inverse variance weighted | 22 | 0.094 | 0.794 | 0.976 | 0.811 | 1.174 |
| ebi-a-GCST90017053 | Gut microbiota abundance (genus Ruminococcaceae UCG002 id.11360) | finn-b-N14_CALCUKIDUR | MR Egger | 22 | 0.256 | 0.840 | 0.949 | 0.575 | 1.566 |
| ebi-a-GCST90017053 | Gut microbiota abundance (genus Ruminococcaceae UCG002 id.11360) | finn-b-N14_CALCUKIDUR | Weighted median | 22 | 0.127 | 0.644 | 1.060 | 0.827 | 1.360 |
| ebi-a-GCST90017053 | Gut microbiota abundance (genus Ruminococcaceae UCG002 id.11360) | finn-b-N14_CALCUKIDUR | Maximum likelihood | 22 | 0.084 | 0.751 | 0.974 | 0.827 | 1.147 |
| ebi-a-GCST90017054 | Gut microbiota abundance (genus Ruminococcaceae UCG003 id.11361) | finn-b-N14_CALCUKIDUR | Inverse variance weighted | 12 | 0.103 | 0.684 | 1.043 | 0.852 | 1.276 |
| ebi-a-GCST90017054 | Gut microbiota abundance (genus Ruminococcaceae UCG003 id.11361) | finn-b-N14_CALCUKIDUR | MR Egger | 12 | 0.336 | 0.598 | 0.833 | 0.431 | 1.608 |
| ebi-a-GCST90017054 | Gut microbiota abundance (genus Ruminococcaceae UCG003 id.11361) | finn-b-N14_CALCUKIDUR | Weighted median | 12 | 0.139 | 0.405 | 1.123 | 0.855 | 1.474 |
| ebi-a-GCST90017054 | Gut microbiota abundance (genus Ruminococcaceae UCG003 id.11361) | finn-b-N14_CALCUKIDUR | Maximum likelihood | 12 | 0.105 | 0.679 | 1.044 | 0.851 | 1.282 |
| ebi-a-GCST90017055 | Gut microbiota abundance (genus Ruminococcaceae UCG004 id.11362) | finn-b-N14_CALCUKIDUR | Inverse variance weighted | 11 | 0.117 | 0.893 | 1.016 | 0.808 | 1.278 |
| ebi-a-GCST90017055 | Gut microbiota abundance (genus Ruminococcaceae UCG004 id.11362) | finn-b-N14_CALCUKIDUR | MR Egger | 11 | 0.662 | 0.439 | 1.709 | 0.467 | 6.260 |
| ebi-a-GCST90017055 | Gut microbiota abundance (genus Ruminococcaceae UCG004 id.11362) | finn-b-N14_CALCUKIDUR | Weighted median | 11 | 0.141 | 0.956 | 1.008 | 0.764 | 1.329 |
| ebi-a-GCST90017055 | Gut microbiota abundance (genus Ruminococcaceae UCG004 id.11362) | finn-b-N14_CALCUKIDUR | Maximum likelihood | 11 | 0.096 | 0.862 | 1.017 | 0.842 | 1.228 |
| ebi-a-GCST90017056 | Gut microbiota abundance (genus Ruminococcaceae UCG005 id.11363) | finn-b-N14_CALCUKIDUR | Inverse variance weighted | 14 | 0.096 | 0.248 | 1.117 | 0.926 | 1.349 |
| ebi-a-GCST90017056 | Gut microbiota abundance (genus Ruminococcaceae UCG005 id.11363) | finn-b-N14_CALCUKIDUR | MR Egger | 14 | 0.261 | 0.349 | 1.290 | 0.773 | 2.154 |
| ebi-a-GCST90017056 | Gut microbiota abundance (genus Ruminococcaceae UCG005 id.11363) | finn-b-N14_CALCUKIDUR | Weighted median | 14 | 0.125 | 0.171 | 1.187 | 0.929 | 1.516 |
| ebi-a-GCST90017056 | Gut microbiota abundance (genus Ruminococcaceae UCG005 id.11363) | finn-b-N14_CALCUKIDUR | Maximum likelihood | 14 | 0.096 | 0.242 | 1.119 | 0.927 | 1.353 |
| ebi-a-GCST90017057 | Gut microbiota abundance (genus Ruminococcaceae UCG009 id.11366) | finn-b-N14_CALCUKIDUR | Inverse variance weighted | 12 | 0.078 | 0.064 | 0.866 | 0.743 | 1.009 |
| ebi-a-GCST90017057 | Gut microbiota abundance (genus Ruminococcaceae UCG009 id.11366) | finn-b-N14_CALCUKIDUR | MR Egger | 12 | 0.317 | 0.959 | 0.983 | 0.528 | 1.832 |
| ebi-a-GCST90017057 | Gut microbiota abundance (genus Ruminococcaceae UCG009 id.11366) | finn-b-N14_CALCUKIDUR | Weighted median | 12 | 0.113 | 0.679 | 0.954 | 0.764 | 1.191 |
| ebi-a-GCST90017057 | Gut microbiota abundance (genus Ruminococcaceae UCG009 id.11366) | finn-b-N14_CALCUKIDUR | Maximum likelihood | 12 | 0.080 | 0.079 | 0.869 | 0.742 | 1.017 |
| ebi-a-GCST90017058 | Gut microbiota abundance (genus Ruminococcaceae UCG010 id.11367) | finn-b-N14_CALCUKIDUR | Inverse variance weighted | 6 | 0.215 | 0.148 | 1.365 | 0.895 | 2.080 |
| ebi-a-GCST90017058 | Gut microbiota abundance (genus Ruminococcaceae UCG010 id.11367) | finn-b-N14_CALCUKIDUR | MR Egger | 6 | 0.640 | 0.392 | 1.847 | 0.527 | 6.479 |
| ebi-a-GCST90017058 | Gut microbiota abundance (genus Ruminococcaceae UCG010 id.11367) | finn-b-N14_CALCUKIDUR | Weighted median | 6 | 0.199 | 0.068 | 1.437 | 0.974 | 2.122 |
| ebi-a-GCST90017058 | Gut microbiota abundance (genus Ruminococcaceae UCG010 id.11367) | finn-b-N14_CALCUKIDUR | Maximum likelihood | 6 | 0.142 | 0.019 | 1.398 | 1.058 | 1.847 |
| ebi-a-GCST90017059 | Gut microbiota abundance (genus Ruminococcaceae UCG011 id.11368) | finn-b-N14_CALCUKIDUR | Inverse variance weighted | 8 | 0.066 | 0.073 | 0.889 | 0.782 | 1.011 |
| ebi-a-GCST90017059 | Gut microbiota abundance (genus Ruminococcaceae UCG011 id.11368) | finn-b-N14_CALCUKIDUR | MR Egger | 8 | 0.327 | 0.248 | 1.520 | 0.801 | 2.885 |
| ebi-a-GCST90017059 | Gut microbiota abundance (genus Ruminococcaceae UCG011 id.11368) | finn-b-N14_CALCUKIDUR | Weighted median | 8 | 0.084 | 0.295 | 0.916 | 0.776 | 1.080 |
| ebi-a-GCST90017059 | Gut microbiota abundance (genus Ruminococcaceae UCG011 id.11368) | finn-b-N14_CALCUKIDUR | Maximum likelihood | 8 | 0.068 | 0.069 | 0.884 | 0.775 | 1.009 |
| ebi-a-GCST90017060 | Gut microbiota abundance (genus Ruminococcaceae UCG013 id.11370) | finn-b-N14_CALCUKIDUR | Inverse variance weighted | 12 | 0.119 | 0.326 | 0.889 | 0.704 | 1.124 |
| ebi-a-GCST90017060 | Gut microbiota abundance (genus Ruminococcaceae UCG013 id.11370) | finn-b-N14_CALCUKIDUR | MR Egger | 12 | 0.355 | 0.793 | 0.909 | 0.453 | 1.823 |
| ebi-a-GCST90017060 | Gut microbiota abundance (genus Ruminococcaceae UCG013 id.11370) | finn-b-N14_CALCUKIDUR | Weighted median | 12 | 0.161 | 0.408 | 0.875 | 0.638 | 1.200 |
| ebi-a-GCST90017060 | Gut microbiota abundance (genus Ruminococcaceae UCG013 id.11370) | finn-b-N14_CALCUKIDUR | Maximum likelihood | 12 | 0.115 | 0.286 | 0.884 | 0.705 | 1.109 |
| ebi-a-GCST90017061 | Gut microbiota abundance (genus Ruminococcaceae UCG014 id.11371) | finn-b-N14_CALCUKIDUR | Inverse variance weighted | 11 | 0.103 | 0.803 | 0.975 | 0.797 | 1.192 |
| ebi-a-GCST90017061 | Gut microbiota abundance (genus Ruminococcaceae UCG014 id.11371) | finn-b-N14_CALCUKIDUR | MR Egger | 11 | 0.243 | 0.272 | 0.753 | 0.468 | 1.212 |
| ebi-a-GCST90017061 | Gut microbiota abundance (genus Ruminococcaceae UCG014 id.11371) | finn-b-N14_CALCUKIDUR | Weighted median | 11 | 0.139 | 0.492 | 0.909 | 0.692 | 1.194 |
| ebi-a-GCST90017061 | Gut microbiota abundance (genus Ruminococcaceae UCG014 id.11371) | finn-b-N14_CALCUKIDUR | Maximum likelihood | 11 | 0.104 | 0.805 | 0.975 | 0.795 | 1.195 |
| ebi-a-GCST90017062 | Gut microbiota abundance (genus Ruminococcus1 id.11373) | finn-b-N14_CALCUKIDUR | Inverse variance weighted | 10 | 0.173 | 0.252 | 0.820 | 0.584 | 1.152 |
| ebi-a-GCST90017062 | Gut microbiota abundance (genus Ruminococcus1 id.11373) | finn-b-N14_CALCUKIDUR | MR Egger | 10 | 0.400 | 0.046 | 0.390 | 0.178 | 0.854 |
| ebi-a-GCST90017062 | Gut microbiota abundance (genus Ruminococcus1 id.11373) | finn-b-N14_CALCUKIDUR | Weighted median | 10 | 0.173 | 0.350 | 0.851 | 0.606 | 1.194 |
| ebi-a-GCST90017062 | Gut microbiota abundance (genus Ruminococcus1 id.11373) | finn-b-N14_CALCUKIDUR | Maximum likelihood | 10 | 0.122 | 0.075 | 0.804 | 0.633 | 1.022 |
| ebi-a-GCST90017063 | Gut microbiota abundance (genus Ruminococcus2 id.11374) | finn-b-N14_CALCUKIDUR | Inverse variance weighted | 15 | 0.111 | 0.972 | 1.004 | 0.807 | 1.249 |
| ebi-a-GCST90017063 | Gut microbiota abundance (genus Ruminococcus2 id.11374) | finn-b-N14_CALCUKIDUR | MR Egger | 15 | 0.277 | 0.733 | 0.908 | 0.527 | 1.564 |
| ebi-a-GCST90017063 | Gut microbiota abundance (genus Ruminococcus2 id.11374) | finn-b-N14_CALCUKIDUR | Weighted median | 15 | 0.129 | 0.836 | 0.974 | 0.756 | 1.254 |
| ebi-a-GCST90017063 | Gut microbiota abundance (genus Ruminococcus2 id.11374) | finn-b-N14_CALCUKIDUR | Maximum likelihood | 15 | 0.092 | 0.965 | 1.004 | 0.838 | 1.202 |
| ebi-a-GCST90017064 | Gut microbiota abundance (genus Ruminococcus gauvreauii group id.11342) | finn-b-N14_CALCUKIDUR | Inverse variance weighted | 12 | 0.112 | 0.662 | 0.952 | 0.764 | 1.187 |
| ebi-a-GCST90017064 | Gut microbiota abundance (genus Ruminococcus gauvreauii group id.11342) | finn-b-N14_CALCUKIDUR | MR Egger | 12 | 0.453 | 0.209 | 0.544 | 0.224 | 1.322 |
| ebi-a-GCST90017064 | Gut microbiota abundance (genus Ruminococcus gauvreauii group id.11342) | finn-b-N14_CALCUKIDUR | Weighted median | 12 | 0.137 | 0.682 | 1.058 | 0.808 | 1.385 |
| ebi-a-GCST90017064 | Gut microbiota abundance (genus Ruminococcus gauvreauii group id.11342) | finn-b-N14_CALCUKIDUR | Maximum likelihood | 12 | 0.106 | 0.649 | 0.953 | 0.774 | 1.173 |
| ebi-a-GCST90017065 | Gut microbiota abundance (genus Ruminococcus gnavus group id.14376) | finn-b-N14_CALCUKIDUR | Inverse variance weighted | 12 | 0.091 | 0.353 | 0.919 | 0.769 | 1.098 |
| ebi-a-GCST90017065 | Gut microbiota abundance (genus Ruminococcus gnavus group id.14376) | finn-b-N14_CALCUKIDUR | MR Egger | 12 | 0.445 | 0.544 | 0.756 | 0.316 | 1.809 |
| ebi-a-GCST90017065 | Gut microbiota abundance (genus Ruminococcus gnavus group id.14376) | finn-b-N14_CALCUKIDUR | Weighted median | 12 | 0.104 | 0.383 | 0.913 | 0.744 | 1.120 |
| ebi-a-GCST90017065 | Gut microbiota abundance (genus Ruminococcus gnavus group id.14376) | finn-b-N14_CALCUKIDUR | Maximum likelihood | 12 | 0.076 | 0.229 | 0.913 | 0.787 | 1.059 |
| ebi-a-GCST90017066 | Gut microbiota abundance (genus Ruminococcus torques group id.14377) | finn-b-N14_CALCUKIDUR | Inverse variance weighted | 9 | 0.148 | 0.487 | 1.109 | 0.829 | 1.483 |
| ebi-a-GCST90017066 | Gut microbiota abundance (genus Ruminococcus torques group id.14377) | finn-b-N14_CALCUKIDUR | MR Egger | 9 | 0.476 | 0.315 | 1.673 | 0.658 | 4.250 |
| ebi-a-GCST90017066 | Gut microbiota abundance (genus Ruminococcus torques group id.14377) | finn-b-N14_CALCUKIDUR | Weighted median | 9 | 0.185 | 0.350 | 1.189 | 0.827 | 1.710 |
| ebi-a-GCST90017066 | Gut microbiota abundance (genus Ruminococcus torques group id.14377) | finn-b-N14_CALCUKIDUR | Maximum likelihood | 9 | 0.150 | 0.482 | 1.111 | 0.828 | 1.492 |
| ebi-a-GCST90017067 | Gut microbiota abundance (genus Sellimonas id.14369) | finn-b-N14_CALCUKIDUR | Inverse variance weighted | 9 | 0.106 | 0.857 | 1.019 | 0.828 | 1.255 |
| ebi-a-GCST90017067 | Gut microbiota abundance (genus Sellimonas id.14369) | finn-b-N14_CALCUKIDUR | MR Egger | 9 | 0.654 | 0.739 | 0.797 | 0.221 | 2.870 |
| ebi-a-GCST90017067 | Gut microbiota abundance (genus Sellimonas id.14369) | finn-b-N14_CALCUKIDUR | Weighted median | 9 | 0.086 | 0.345 | 1.085 | 0.916 | 1.284 |
| ebi-a-GCST90017067 | Gut microbiota abundance (genus Sellimonas id.14369) | finn-b-N14_CALCUKIDUR | Maximum likelihood | 9 | 0.062 | 0.718 | 1.023 | 0.906 | 1.155 |
| ebi-a-GCST90017068 | Gut microbiota abundance (genus Senegalimassilia id.11160) | finn-b-N14_CALCUKIDUR | Inverse variance weighted | 5 | 0.128 | 0.184 | 1.186 | 0.922 | 1.525 |
| ebi-a-GCST90017068 | Gut microbiota abundance (genus Senegalimassilia id.11160) | finn-b-N14_CALCUKIDUR | MR Egger | 5 | 0.511 | 0.596 | 1.352 | 0.496 | 3.684 |
| ebi-a-GCST90017068 | Gut microbiota abundance (genus Senegalimassilia id.11160) | finn-b-N14_CALCUKIDUR | Weighted median | 5 | 0.163 | 0.188 | 1.240 | 0.900 | 1.708 |
| ebi-a-GCST90017068 | Gut microbiota abundance (genus Senegalimassilia id.11160) | finn-b-N14_CALCUKIDUR | Maximum likelihood | 5 | 0.131 | 0.182 | 1.190 | 0.922 | 1.537 |
| ebi-a-GCST90017069 | Gut microbiota abundance (genus Slackia id.825) | finn-b-N14_CALCUKIDUR | Inverse variance weighted | 6 | 0.141 | 0.996 | 0.999 | 0.759 | 1.316 |
| ebi-a-GCST90017069 | Gut microbiota abundance (genus Slackia id.825) | finn-b-N14_CALCUKIDUR | MR Egger | 6 | 1.017 | 0.943 | 0.925 | 0.126 | 6.786 |
| ebi-a-GCST90017069 | Gut microbiota abundance (genus Slackia id.825) | finn-b-N14_CALCUKIDUR | Weighted median | 6 | 0.143 | 0.506 | 1.100 | 0.831 | 1.456 |
| ebi-a-GCST90017069 | Gut microbiota abundance (genus Slackia id.825) | finn-b-N14_CALCUKIDUR | Maximum likelihood | 6 | 0.110 | 0.995 | 0.999 | 0.805 | 1.240 |
| ebi-a-GCST90017070 | Gut microbiota abundance (genus Streptococcus id.1853) | finn-b-N14_CALCUKIDUR | Inverse variance weighted | 14 | 0.109 | 0.276 | 1.127 | 0.909 | 1.396 |
| ebi-a-GCST90017070 | Gut microbiota abundance (genus Streptococcus id.1853) | finn-b-N14_CALCUKIDUR | MR Egger | 14 | 0.396 | 0.685 | 1.179 | 0.543 | 2.562 |
| ebi-a-GCST90017070 | Gut microbiota abundance (genus Streptococcus id.1853) | finn-b-N14_CALCUKIDUR | Weighted median | 14 | 0.159 | 0.848 | 1.031 | 0.755 | 1.408 |
| ebi-a-GCST90017070 | Gut microbiota abundance (genus Streptococcus id.1853) | finn-b-N14_CALCUKIDUR | Maximum likelihood | 14 | 0.112 | 0.269 | 1.132 | 0.909 | 1.410 |
| ebi-a-GCST90017071 | Gut microbiota abundance (genus Subdoligranulum id.2070) | finn-b-N14_CALCUKIDUR | Inverse variance weighted | 11 | 0.129 | 0.070 | 1.264 | 0.981 | 1.629 |
| ebi-a-GCST90017071 | Gut microbiota abundance (genus Subdoligranulum id.2070) | finn-b-N14_CALCUKIDUR | MR Egger | 11 | 0.337 | 0.857 | 0.939 | 0.485 | 1.819 |
| ebi-a-GCST90017071 | Gut microbiota abundance (genus Subdoligranulum id.2070) | finn-b-N14_CALCUKIDUR | Weighted median | 11 | 0.158 | 0.384 | 1.148 | 0.842 | 1.565 |
| ebi-a-GCST90017071 | Gut microbiota abundance (genus Subdoligranulum id.2070) | finn-b-N14_CALCUKIDUR | Maximum likelihood | 11 | 0.120 | 0.041 | 1.277 | 1.009 | 1.615 |
| ebi-a-GCST90017072 | Gut microbiota abundance (genus Sutterella id.2896) | finn-b-N14_CALCUKIDUR | Inverse variance weighted | 12 | 0.105 | 0.059 | 1.219 | 0.992 | 1.497 |
| ebi-a-GCST90017072 | Gut microbiota abundance (genus Sutterella id.2896) | finn-b-N14_CALCUKIDUR | MR Egger | 12 | 0.456 | 0.087 | 2.374 | 0.972 | 5.799 |
| ebi-a-GCST90017072 | Gut microbiota abundance (genus Sutterella id.2896) | finn-b-N14_CALCUKIDUR | Weighted median | 12 | 0.144 | 0.325 | 1.152 | 0.869 | 1.527 |
| ebi-a-GCST90017072 | Gut microbiota abundance (genus Sutterella id.2896) | finn-b-N14_CALCUKIDUR | Maximum likelihood | 12 | 0.108 | 0.054 | 1.231 | 0.996 | 1.521 |
| ebi-a-GCST90017073 | Gut microbiota abundance (genus Terrisporobacter id.11348) | finn-b-N14_CALCUKIDUR | Inverse variance weighted | 5 | 0.151 | 0.358 | 1.149 | 0.854 | 1.545 |
| ebi-a-GCST90017073 | Gut microbiota abundance (genus Terrisporobacter id.11348) | finn-b-N14_CALCUKIDUR | MR Egger | 5 | 0.514 | 0.587 | 1.366 | 0.499 | 3.744 |
| ebi-a-GCST90017073 | Gut microbiota abundance (genus Terrisporobacter id.11348) | finn-b-N14_CALCUKIDUR | Weighted median | 5 | 0.161 | 0.712 | 1.061 | 0.774 | 1.454 |
| ebi-a-GCST90017073 | Gut microbiota abundance (genus Terrisporobacter id.11348) | finn-b-N14_CALCUKIDUR | Maximum likelihood | 5 | 0.126 | 0.244 | 1.158 | 0.905 | 1.483 |
| ebi-a-GCST90017074 | Gut microbiota abundance (genus Turicibacter id.2162) | finn-b-N14_CALCUKIDUR | Inverse variance weighted | 10 | 0.092 | 0.793 | 0.976 | 0.814 | 1.170 |
| ebi-a-GCST90017074 | Gut microbiota abundance (genus Turicibacter id.2162) | finn-b-N14_CALCUKIDUR | MR Egger | 10 | 0.397 | 0.394 | 1.430 | 0.656 | 3.115 |
| ebi-a-GCST90017074 | Gut microbiota abundance (genus Turicibacter id.2162) | finn-b-N14_CALCUKIDUR | Weighted median | 10 | 0.121 | 0.861 | 1.021 | 0.805 | 1.296 |
| ebi-a-GCST90017074 | Gut microbiota abundance (genus Turicibacter id.2162) | finn-b-N14_CALCUKIDUR | Maximum likelihood | 10 | 0.094 | 0.807 | 0.977 | 0.813 | 1.175 |
| ebi-a-GCST90017075 | Gut microbiota abundance (genus Tyzzerella3 id.11335) | finn-b-N14_CALCUKIDUR | Inverse variance weighted | 13 | 0.065 | 0.604 | 1.034 | 0.910 | 1.176 |
| ebi-a-GCST90017075 | Gut microbiota abundance (genus Tyzzerella3 id.11335) | finn-b-N14_CALCUKIDUR | MR Egger | 13 | 0.375 | 0.304 | 0.668 | 0.320 | 1.392 |
| ebi-a-GCST90017075 | Gut microbiota abundance (genus Tyzzerella3 id.11335) | finn-b-N14_CALCUKIDUR | Weighted median | 13 | 0.086 | 0.750 | 1.028 | 0.868 | 1.217 |
| ebi-a-GCST90017075 | Gut microbiota abundance (genus Tyzzerella3 id.11335) | finn-b-N14_CALCUKIDUR | Maximum likelihood | 13 | 0.066 | 0.593 | 1.036 | 0.910 | 1.180 |
| ebi-a-GCST90017088 | Gut microbiota abundance (genus Veillonella id.2198) | finn-b-N14_CALCUKIDUR | Inverse variance weighted | 6 | 0.127 | 0.816 | 0.971 | 0.757 | 1.245 |
| ebi-a-GCST90017088 | Gut microbiota abundance (genus Veillonella id.2198) | finn-b-N14_CALCUKIDUR | MR Egger | 6 | 1.009 | 0.758 | 1.396 | 0.193 | 10.095 |
| ebi-a-GCST90017088 | Gut microbiota abundance (genus Veillonella id.2198) | finn-b-N14_CALCUKIDUR | Weighted median | 6 | 0.160 | 0.977 | 0.995 | 0.728 | 1.361 |
| ebi-a-GCST90017088 | Gut microbiota abundance (genus Veillonella id.2198) | finn-b-N14_CALCUKIDUR | Maximum likelihood | 6 | 0.129 | 0.815 | 0.970 | 0.754 | 1.249 |
| ebi-a-GCST90017089 | Gut microbiota abundance (genus Victivallis id.2256) | finn-b-N14_CALCUKIDUR | Inverse variance weighted | 10 | 0.068 | 0.288 | 1.075 | 0.941 | 1.229 |
| ebi-a-GCST90017089 | Gut microbiota abundance (genus Victivallis id.2256) | finn-b-N14_CALCUKIDUR | MR Egger | 10 | 0.549 | 0.674 | 1.271 | 0.434 | 3.726 |
| ebi-a-GCST90017089 | Gut microbiota abundance (genus Victivallis id.2256) | finn-b-N14_CALCUKIDUR | Weighted median | 10 | 0.085 | 0.413 | 0.933 | 0.790 | 1.101 |
| ebi-a-GCST90017089 | Gut microbiota abundance (genus Victivallis id.2256) | finn-b-N14_CALCUKIDUR | Maximum likelihood | 10 | 0.063 | 0.220 | 1.080 | 0.955 | 1.221 |
| ebi-a-GCST90016908 | Gut microbiota abundance (class Actinobacteria id.419) | finn-b-N14_CALCUKIDUR | Inverse variance weighted | 15 | 0.108 | 0.236 | 1.137 | 0.920 | 1.404 |
| ebi-a-GCST90016908 | Gut microbiota abundance (class Actinobacteria id.419) | finn-b-N14_CALCUKIDUR | Maximum likelihood | 15 | 0.095 | 0.161 | 1.143 | 0.948 | 1.378 |
| ebi-a-GCST90016908 | Gut microbiota abundance (class Actinobacteria id.419) | finn-b-N14_CALCUKIDUR | MR Egger | 15 | 0.306 | 0.135 | 1.627 | 0.894 | 2.962 |
| ebi-a-GCST90016908 | Gut microbiota abundance (class Actinobacteria id.419) | finn-b-N14_CALCUKIDUR | Weighted median | 15 | 0.133 | 0.024 | 1.349 | 1.040 | 1.750 |
| ebi-a-GCST90016909 | Gut microbiota abundance (class Alphaproteobacteria id.2379) | finn-b-N14_CALCUKIDUR | Inverse variance weighted | 7 | 0.113 | 0.595 | 0.942 | 0.755 | 1.175 |
| ebi-a-GCST90016909 | Gut microbiota abundance (class Alphaproteobacteria id.2379) | finn-b-N14_CALCUKIDUR | Maximum likelihood | 7 | 0.115 | 0.590 | 0.940 | 0.751 | 1.177 |
| ebi-a-GCST90016909 | Gut microbiota abundance (class Alphaproteobacteria id.2379) | finn-b-N14_CALCUKIDUR | MR Egger | 7 | 0.422 | 0.311 | 1.609 | 0.703 | 3.683 |
| ebi-a-GCST90016909 | Gut microbiota abundance (class Alphaproteobacteria id.2379) | finn-b-N14_CALCUKIDUR | Weighted median | 7 | 0.144 | 0.842 | 0.972 | 0.733 | 1.288 |
| ebi-a-GCST90016910 | Gut microbiota abundance (class Bacilli id.1673) | finn-b-N14_CALCUKIDUR | Inverse variance weighted | 18 | 0.094 | 0.551 | 1.058 | 0.879 | 1.273 |
| ebi-a-GCST90016910 | Gut microbiota abundance (class Bacilli id.1673) | finn-b-N14_CALCUKIDUR | Maximum likelihood | 18 | 0.097 | 0.548 | 1.060 | 0.877 | 1.282 |
| ebi-a-GCST90016910 | Gut microbiota abundance (class Bacilli id.1673) | finn-b-N14_CALCUKIDUR | MR Egger | 18 | 0.259 | 0.119 | 0.653 | 0.393 | 1.084 |
| ebi-a-GCST90016910 | Gut microbiota abundance (class Bacilli id.1673) | finn-b-N14_CALCUKIDUR | Weighted median | 18 | 0.132 | 0.299 | 1.147 | 0.885 | 1.487 |
| ebi-a-GCST90016911 | Gut microbiota abundance (class Bacteroidia id.912) | finn-b-N14_CALCUKIDUR | Inverse variance weighted | 14 | 0.107 | 0.735 | 1.037 | 0.841 | 1.278 |
| ebi-a-GCST90016911 | Gut microbiota abundance (class Bacteroidia id.912) | finn-b-N14_CALCUKIDUR | Maximum likelihood | 14 | 0.108 | 0.731 | 1.038 | 0.839 | 1.284 |
| ebi-a-GCST90016911 | Gut microbiota abundance (class Bacteroidia id.912) | finn-b-N14_CALCUKIDUR | MR Egger | 14 | 0.218 | 0.642 | 1.110 | 0.724 | 1.702 |
| ebi-a-GCST90016911 | Gut microbiota abundance (class Bacteroidia id.912) | finn-b-N14_CALCUKIDUR | Weighted median | 14 | 0.137 | 0.394 | 1.124 | 0.859 | 1.470 |
| ebi-a-GCST90016912 | Gut microbiota abundance (class Betaproteobacteria id.2867) | finn-b-N14_CALCUKIDUR | Inverse variance weighted | 11 | 0.122 | 0.159 | 1.187 | 0.935 | 1.507 |
| ebi-a-GCST90016912 | Gut microbiota abundance (class Betaproteobacteria id.2867) | finn-b-N14_CALCUKIDUR | Maximum likelihood | 11 | 0.125 | 0.150 | 1.197 | 0.937 | 1.528 |
| ebi-a-GCST90016912 | Gut microbiota abundance (class Betaproteobacteria id.2867) | finn-b-N14_CALCUKIDUR | MR Egger | 11 | 0.407 | 0.227 | 1.696 | 0.764 | 3.766 |
| ebi-a-GCST90016912 | Gut microbiota abundance (class Betaproteobacteria id.2867) | finn-b-N14_CALCUKIDUR | Weighted median | 11 | 0.178 | 0.185 | 1.265 | 0.893 | 1.792 |
| ebi-a-GCST90016913 | Gut microbiota abundance (class Clostridia id.1859) | finn-b-N14_CALCUKIDUR | Inverse variance weighted | 12 | 0.113 | 0.113 | 0.836 | 0.670 | 1.043 |
| ebi-a-GCST90016913 | Gut microbiota abundance (class Clostridia id.1859) | finn-b-N14_CALCUKIDUR | Maximum likelihood | 12 | 0.117 | 0.114 | 0.831 | 0.661 | 1.045 |
| ebi-a-GCST90016913 | Gut microbiota abundance (class Clostridia id.1859) | finn-b-N14_CALCUKIDUR | MR Egger | 12 | 0.307 | 0.342 | 1.358 | 0.744 | 2.477 |
| ebi-a-GCST90016913 | Gut microbiota abundance (class Clostridia id.1859) | finn-b-N14_CALCUKIDUR | Weighted median | 12 | 0.161 | 0.658 | 0.931 | 0.680 | 1.276 |
| ebi-a-GCST90016914 | Gut microbiota abundance (class Coriobacteriia id.809) | finn-b-N14_CALCUKIDUR | Inverse variance weighted | 13 | 0.164 | 0.906 | 1.020 | 0.739 | 1.407 |
| ebi-a-GCST90016914 | Gut microbiota abundance (class Coriobacteriia id.809) | finn-b-N14_CALCUKIDUR | Maximum likelihood | 13 | 0.121 | 0.860 | 1.022 | 0.805 | 1.296 |
| ebi-a-GCST90016914 | Gut microbiota abundance (class Coriobacteriia id.809) | finn-b-N14_CALCUKIDUR | MR Egger | 13 | 0.726 | 0.849 | 1.152 | 0.277 | 4.781 |
| ebi-a-GCST90016914 | Gut microbiota abundance (class Coriobacteriia id.809) | finn-b-N14_CALCUKIDUR | Weighted median | 13 | 0.174 | 0.558 | 1.107 | 0.788 | 1.556 |
| ebi-a-GCST90016915 | Gut microbiota abundance (class Deltaproteobacteria id.3087) | finn-b-N14_CALCUKIDUR | Inverse variance weighted | 13 | 0.103 | 0.045 | 0.814 | 0.666 | 0.995 |
| ebi-a-GCST90016915 | Gut microbiota abundance (class Deltaproteobacteria id.3087) | finn-b-N14_CALCUKIDUR | Maximum likelihood | 13 | 0.105 | 0.050 | 0.814 | 0.663 | 1.000 |
| ebi-a-GCST90016915 | Gut microbiota abundance (class Deltaproteobacteria id.3087) | finn-b-N14_CALCUKIDUR | MR Egger | 13 | 0.275 | 0.252 | 0.717 | 0.418 | 1.229 |
| ebi-a-GCST90016915 | Gut microbiota abundance (class Deltaproteobacteria id.3087) | finn-b-N14_CALCUKIDUR | Weighted median | 13 | 0.147 | 0.060 | 0.759 | 0.570 | 1.012 |
| ebi-a-GCST90016916 | Gut microbiota abundance (class Erysipelotrichia id.2147) | finn-b-N14_CALCUKIDUR | Inverse variance weighted | 13 | 0.122 | 0.380 | 0.899 | 0.708 | 1.141 |
| ebi-a-GCST90016916 | Gut microbiota abundance (class Erysipelotrichia id.2147) | finn-b-N14_CALCUKIDUR | Maximum likelihood | 13 | 0.123 | 0.398 | 0.901 | 0.708 | 1.147 |
| ebi-a-GCST90016916 | Gut microbiota abundance (class Erysipelotrichia id.2147) | finn-b-N14_CALCUKIDUR | MR Egger | 13 | 0.527 | 0.476 | 1.475 | 0.525 | 4.142 |
| ebi-a-GCST90016916 | Gut microbiota abundance (class Erysipelotrichia id.2147) | finn-b-N14_CALCUKIDUR | Weighted median | 13 | 0.163 | 0.671 | 0.933 | 0.678 | 1.284 |
| ebi-a-GCST90016917 | Gut microbiota abundance (class Gammaproteobacteria id.3303) | finn-b-N14_CALCUKIDUR | Inverse variance weighted | 6 | 0.274 | 0.690 | 1.116 | 0.652 | 1.911 |
| ebi-a-GCST90016917 | Gut microbiota abundance (class Gammaproteobacteria id.3303) | finn-b-N14_CALCUKIDUR | Maximum likelihood | 6 | 0.178 | 0.490 | 1.130 | 0.798 | 1.601 |
| ebi-a-GCST90016917 | Gut microbiota abundance (class Gammaproteobacteria id.3303) | finn-b-N14_CALCUKIDUR | MR Egger | 6 | 0.915 | 0.860 | 0.842 | 0.140 | 5.055 |
| ebi-a-GCST90016917 | Gut microbiota abundance (class Gammaproteobacteria id.3303) | finn-b-N14_CALCUKIDUR | Weighted median | 6 | 0.256 | 0.761 | 1.081 | 0.654 | 1.786 |
| ebi-a-GCST90016918 | Gut microbiota abundance (class Lentisphaeria id.2250) | finn-b-N14_CALCUKIDUR | Inverse variance weighted | 8 | 0.091 | 0.416 | 0.929 | 0.777 | 1.110 |
| ebi-a-GCST90016918 | Gut microbiota abundance (class Lentisphaeria id.2250) | finn-b-N14_CALCUKIDUR | Maximum likelihood | 8 | 0.079 | 0.349 | 0.928 | 0.795 | 1.084 |
| ebi-a-GCST90016918 | Gut microbiota abundance (class Lentisphaeria id.2250) | finn-b-N14_CALCUKIDUR | MR Egger | 8 | 0.349 | 0.777 | 0.902 | 0.455 | 1.787 |
| ebi-a-GCST90016918 | Gut microbiota abundance (class Lentisphaeria id.2250) | finn-b-N14_CALCUKIDUR | Weighted median | 8 | 0.101 | 0.433 | 0.924 | 0.758 | 1.126 |
| ebi-a-GCST90016919 | Gut microbiota abundance (class Melainabacteria id.1589) | finn-b-N14_CALCUKIDUR | Inverse variance weighted | 10 | 0.078 | 0.305 | 0.923 | 0.792 | 1.076 |
| ebi-a-GCST90016919 | Gut microbiota abundance (class Melainabacteria id.1589) | finn-b-N14_CALCUKIDUR | Maximum likelihood | 10 | 0.079 | 0.307 | 0.922 | 0.790 | 1.077 |
| ebi-a-GCST90016919 | Gut microbiota abundance (class Melainabacteria id.1589) | finn-b-N14_CALCUKIDUR | MR Egger | 10 | 0.236 | 0.322 | 1.283 | 0.808 | 2.038 |
| ebi-a-GCST90016919 | Gut microbiota abundance (class Melainabacteria id.1589) | finn-b-N14_CALCUKIDUR | Weighted median | 10 | 0.102 | 0.490 | 0.932 | 0.764 | 1.138 |
| ebi-a-GCST90016920 | Gut microbiota abundance (class Methanobacteria id.119) | finn-b-N14_CALCUKIDUR | Inverse variance weighted | 10 | 0.064 | 0.496 | 0.958 | 0.846 | 1.085 |
| ebi-a-GCST90016920 | Gut microbiota abundance (class Methanobacteria id.119) | finn-b-N14_CALCUKIDUR | Maximum likelihood | 10 | 0.065 | 0.488 | 0.956 | 0.843 | 1.085 |
| ebi-a-GCST90016920 | Gut microbiota abundance (class Methanobacteria id.119) | finn-b-N14_CALCUKIDUR | MR Egger | 10 | 0.261 | 0.194 | 1.448 | 0.869 | 2.414 |
| ebi-a-GCST90016920 | Gut microbiota abundance (class Methanobacteria id.119) | finn-b-N14_CALCUKIDUR | Weighted median | 10 | 0.083 | 0.760 | 0.975 | 0.828 | 1.148 |
| ebi-a-GCST90016921 | Gut microbiota abundance (class Mollicutes id.3920) | finn-b-N14_CALCUKIDUR | Inverse variance weighted | 12 | 0.096 | 0.319 | 0.909 | 0.752 | 1.097 |
| ebi-a-GCST90016921 | Gut microbiota abundance (class Mollicutes id.3920) | finn-b-N14_CALCUKIDUR | Maximum likelihood | 12 | 0.097 | 0.342 | 0.912 | 0.753 | 1.103 |
| ebi-a-GCST90016921 | Gut microbiota abundance (class Mollicutes id.3920) | finn-b-N14_CALCUKIDUR | MR Egger | 12 | 0.327 | 0.772 | 0.907 | 0.478 | 1.723 |
| ebi-a-GCST90016921 | Gut microbiota abundance (class Mollicutes id.3920) | finn-b-N14_CALCUKIDUR | Weighted median | 12 | 0.113 | 0.761 | 0.966 | 0.774 | 1.207 |
| ebi-a-GCST90016922 | Gut microbiota abundance (class Negativicutes id.2164) | finn-b-N14_CALCUKIDUR | Inverse variance weighted | 12 | 0.121 | 0.822 | 0.973 | 0.767 | 1.235 |
| ebi-a-GCST90016922 | Gut microbiota abundance (class Negativicutes id.2164) | finn-b-N14_CALCUKIDUR | Maximum likelihood | 12 | 0.124 | 0.824 | 0.973 | 0.763 | 1.240 |
| ebi-a-GCST90016922 | Gut microbiota abundance (class Negativicutes id.2164) | finn-b-N14_CALCUKIDUR | MR Egger | 12 | 0.418 | 0.314 | 0.642 | 0.283 | 1.456 |
| ebi-a-GCST90016922 | Gut microbiota abundance (class Negativicutes id.2164) | finn-b-N14_CALCUKIDUR | Weighted median | 12 | 0.172 | 0.720 | 0.940 | 0.671 | 1.317 |
| ebi-a-GCST90016923 | Gut microbiota abundance (class Verrucomicrobiae id.4029) | finn-b-N14_CALCUKIDUR | Inverse variance weighted | 11 | 0.101 | 0.848 | 0.981 | 0.804 | 1.196 |
| ebi-a-GCST90016923 | Gut microbiota abundance (class Verrucomicrobiae id.4029) | finn-b-N14_CALCUKIDUR | Maximum likelihood | 11 | 0.103 | 0.852 | 0.981 | 0.802 | 1.199 |
| ebi-a-GCST90016923 | Gut microbiota abundance (class Verrucomicrobiae id.4029) | finn-b-N14_CALCUKIDUR | MR Egger | 11 | 0.338 | 0.531 | 1.246 | 0.643 | 2.417 |
| ebi-a-GCST90016923 | Gut microbiota abundance (class Verrucomicrobiae id.4029) | finn-b-N14_CALCUKIDUR | Weighted median | 11 | 0.131 | 0.403 | 1.116 | 0.863 | 1.443 |
| ebi-a-GCST90016924 | Gut microbiota abundance (family Acidaminococcaceae id.2166) | finn-b-N14_CALCUKIDUR | Inverse variance weighted | 7 | 0.155 | 0.707 | 0.943 | 0.696 | 1.279 |
| ebi-a-GCST90016924 | Gut microbiota abundance (family Acidaminococcaceae id.2166) | finn-b-N14_CALCUKIDUR | Maximum likelihood | 7 | 0.121 | 0.611 | 0.940 | 0.742 | 1.191 |
| ebi-a-GCST90016924 | Gut microbiota abundance (family Acidaminococcaceae id.2166) | finn-b-N14_CALCUKIDUR | MR Egger | 7 | 0.469 | 0.331 | 0.604 | 0.241 | 1.514 |
| ebi-a-GCST90016924 | Gut microbiota abundance (family Acidaminococcaceae id.2166) | finn-b-N14_CALCUKIDUR | Weighted median | 7 | 0.156 | 0.770 | 0.955 | 0.704 | 1.297 |
| ebi-a-GCST90016925 | Gut microbiota abundance (family Actinomycetaceae id.421) | finn-b-N14_CALCUKIDUR | Inverse variance weighted | 5 | 0.117 | 0.599 | 0.940 | 0.747 | 1.183 |
| ebi-a-GCST90016925 | Gut microbiota abundance (family Actinomycetaceae id.421) | finn-b-N14_CALCUKIDUR | Maximum likelihood | 5 | 0.118 | 0.595 | 0.939 | 0.745 | 1.184 |
| ebi-a-GCST90016925 | Gut microbiota abundance (family Actinomycetaceae id.421) | finn-b-N14_CALCUKIDUR | MR Egger | 5 | 0.268 | 0.332 | 0.734 | 0.434 | 1.241 |
| ebi-a-GCST90016925 | Gut microbiota abundance (family Actinomycetaceae id.421) | finn-b-N14_CALCUKIDUR | Weighted median | 5 | 0.152 | 0.549 | 0.913 | 0.677 | 1.231 |
| ebi-a-GCST90016926 | Gut microbiota abundance (family Alcaligenaceae id.2875) | finn-b-N14_CALCUKIDUR | Inverse variance weighted | 11 | 0.128 | 0.452 | 1.101 | 0.857 | 1.415 |
| ebi-a-GCST90016926 | Gut microbiota abundance (family Alcaligenaceae id.2875) | finn-b-N14_CALCUKIDUR | Maximum likelihood | 11 | 0.130 | 0.435 | 1.107 | 0.858 | 1.428 |
| ebi-a-GCST90016926 | Gut microbiota abundance (family Alcaligenaceae id.2875) | finn-b-N14_CALCUKIDUR | MR Egger | 11 | 0.583 | 0.207 | 2.212 | 0.705 | 6.941 |
| ebi-a-GCST90016926 | Gut microbiota abundance (family Alcaligenaceae id.2875) | finn-b-N14_CALCUKIDUR | Weighted median | 11 | 0.171 | 0.942 | 0.988 | 0.706 | 1.381 |
| ebi-a-GCST90016927 | Gut microbiota abundance (family Bacteroidaceae id.917) | finn-b-N14_CALCUKIDUR | Inverse variance weighted | 9 | 0.184 | 0.387 | 1.173 | 0.818 | 1.683 |
| ebi-a-GCST90016927 | Gut microbiota abundance (family Bacteroidaceae id.917) | finn-b-N14_CALCUKIDUR | Maximum likelihood | 9 | 0.141 | 0.217 | 1.191 | 0.903 | 1.570 |
| ebi-a-GCST90016927 | Gut microbiota abundance (family Bacteroidaceae id.917) | finn-b-N14_CALCUKIDUR | MR Egger | 9 | 1.006 | 0.774 | 0.741 | 0.103 | 5.319 |
| ebi-a-GCST90016927 | Gut microbiota abundance (family Bacteroidaceae id.917) | finn-b-N14_CALCUKIDUR | Weighted median | 9 | 0.200 | 0.810 | 1.049 | 0.709 | 1.552 |
| ebi-a-GCST90016928 | Gut microbiota abundance (family Bacteroidales S24 7group id.11173) | finn-b-N14_CALCUKIDUR | Inverse variance weighted | 8 | 0.095 | 0.942 | 1.007 | 0.836 | 1.212 |
| ebi-a-GCST90016928 | Gut microbiota abundance (family Bacteroidales S24 7group id.11173) | finn-b-N14_CALCUKIDUR | Maximum likelihood | 8 | 0.096 | 0.941 | 1.007 | 0.834 | 1.216 |
| ebi-a-GCST90016928 | Gut microbiota abundance (family Bacteroidales S24 7group id.11173) | finn-b-N14_CALCUKIDUR | MR Egger | 8 | 0.381 | 0.216 | 1.695 | 0.803 | 3.580 |
| ebi-a-GCST90016928 | Gut microbiota abundance (family Bacteroidales S24 7group id.11173) | finn-b-N14_CALCUKIDUR | Weighted median | 8 | 0.124 | 0.884 | 1.018 | 0.799 | 1.298 |
| ebi-a-GCST90016929 | Gut microbiota abundance (family Bifidobacteriaceae id.433) | finn-b-N14_CALCUKIDUR | Inverse variance weighted | 12 | 0.113 | 0.165 | 1.169 | 0.938 | 1.458 |
| ebi-a-GCST90016929 | Gut microbiota abundance (family Bifidobacteriaceae id.433) | finn-b-N14_CALCUKIDUR | Maximum likelihood | 12 | 0.101 | 0.117 | 1.171 | 0.961 | 1.426 |
| ebi-a-GCST90016929 | Gut microbiota abundance (family Bifidobacteriaceae id.433) | finn-b-N14_CALCUKIDUR | MR Egger | 12 | 0.333 | 0.046 | 2.136 | 1.113 | 4.101 |
| ebi-a-GCST90016929 | Gut microbiota abundance (family Bifidobacteriaceae id.433) | finn-b-N14_CALCUKIDUR | Weighted median | 12 | 0.144 | 0.114 | 1.257 | 0.947 | 1.668 |
| ebi-a-GCST90016931 | Gut microbiota abundance (family Clostridiaceae1 id.1869) | finn-b-N14_CALCUKIDUR | Inverse variance weighted | 10 | 0.116 | 0.007 | 0.729 | 0.581 | 0.916 |
| ebi-a-GCST90016931 | Gut microbiota abundance (family Clostridiaceae1 id.1869) | finn-b-N14_CALCUKIDUR | Maximum likelihood | 10 | 0.123 | 0.008 | 0.721 | 0.566 | 0.918 |
| ebi-a-GCST90016931 | Gut microbiota abundance (family Clostridiaceae1 id.1869) | finn-b-N14_CALCUKIDUR | MR Egger | 10 | 0.344 | 0.319 | 0.694 | 0.354 | 1.362 |
| ebi-a-GCST90016931 | Gut microbiota abundance (family Clostridiaceae1 id.1869) | finn-b-N14_CALCUKIDUR | Weighted median | 10 | 0.151 | 0.156 | 0.807 | 0.600 | 1.086 |
| ebi-a-GCST90016932 | Gut microbiota abundance (family Clostridiales vadin BB60 group id.11286) | finn-b-N14_CALCUKIDUR | Inverse variance weighted | 15 | 0.081 | 0.712 | 0.970 | 0.828 | 1.138 |
| ebi-a-GCST90016932 | Gut microbiota abundance (family Clostridiales vadin BB60 group id.11286) | finn-b-N14_CALCUKIDUR | Maximum likelihood | 15 | 0.082 | 0.709 | 0.970 | 0.826 | 1.139 |
| ebi-a-GCST90016932 | Gut microbiota abundance (family Clostridiales vadin BB60 group id.11286) | finn-b-N14_CALCUKIDUR | MR Egger | 15 | 0.222 | 0.438 | 1.195 | 0.773 | 1.848 |
| ebi-a-GCST90016932 | Gut microbiota abundance (family Clostridiales vadin BB60 group id.11286) | finn-b-N14_CALCUKIDUR | Weighted median | 15 | 0.107 | 0.928 | 1.010 | 0.818 | 1.246 |
| ebi-a-GCST90016933 | Gut microbiota abundance (family Coriobacteriaceae id.811) | finn-b-N14_CALCUKIDUR | Inverse variance weighted | 13 | 0.164 | 0.906 | 1.020 | 0.739 | 1.407 |
| ebi-a-GCST90016933 | Gut microbiota abundance (family Coriobacteriaceae id.811) | finn-b-N14_CALCUKIDUR | Maximum likelihood | 13 | 0.121 | 0.860 | 1.022 | 0.805 | 1.296 |
| ebi-a-GCST90016933 | Gut microbiota abundance (family Coriobacteriaceae id.811) | finn-b-N14_CALCUKIDUR | MR Egger | 13 | 0.726 | 0.849 | 1.152 | 0.277 | 4.781 |
| ebi-a-GCST90016933 | Gut microbiota abundance (family Coriobacteriaceae id.811) | finn-b-N14_CALCUKIDUR | Weighted median | 13 | 0.170 | 0.549 | 1.107 | 0.794 | 1.545 |
| ebi-a-GCST90016934 | Gut microbiota abundance (family Defluviitaleaceae id.1924) | finn-b-N14_CALCUKIDUR | Inverse variance weighted | 11 | 0.090 | 0.371 | 1.084 | 0.909 | 1.293 |
| ebi-a-GCST90016934 | Gut microbiota abundance (family Defluviitaleaceae id.1924) | finn-b-N14_CALCUKIDUR | Maximum likelihood | 11 | 0.092 | 0.365 | 1.087 | 0.908 | 1.300 |
| ebi-a-GCST90016934 | Gut microbiota abundance (family Defluviitaleaceae id.1924) | finn-b-N14_CALCUKIDUR | MR Egger | 11 | 0.307 | 0.700 | 0.885 | 0.484 | 1.616 |
| ebi-a-GCST90016934 | Gut microbiota abundance (family Defluviitaleaceae id.1924) | finn-b-N14_CALCUKIDUR | Weighted median | 11 | 0.123 | 0.696 | 1.049 | 0.825 | 1.334 |
| ebi-a-GCST90016935 | Gut microbiota abundance (family Desulfovibrionaceae id.3169) | finn-b-N14_CALCUKIDUR | Inverse variance weighted | 10 | 0.117 | 0.077 | 0.813 | 0.646 | 1.023 |
| ebi-a-GCST90016935 | Gut microbiota abundance (family Desulfovibrionaceae id.3169) | finn-b-N14_CALCUKIDUR | Maximum likelihood | 10 | 0.115 | 0.060 | 0.806 | 0.644 | 1.009 |
| ebi-a-GCST90016935 | Gut microbiota abundance (family Desulfovibrionaceae id.3169) | finn-b-N14_CALCUKIDUR | MR Egger | 10 | 0.297 | 0.328 | 0.733 | 0.409 | 1.314 |
| ebi-a-GCST90016935 | Gut microbiota abundance (family Desulfovibrionaceae id.3169) | finn-b-N14_CALCUKIDUR | Weighted median | 10 | 0.162 | 0.253 | 0.831 | 0.604 | 1.142 |
| ebi-a-GCST90016936 | Gut microbiota abundance (family Enterobacteriaceae id.3469) | finn-b-N14_CALCUKIDUR | Inverse variance weighted | 7 | 0.244 | 0.905 | 1.030 | 0.638 | 1.661 |
| ebi-a-GCST90016936 | Gut microbiota abundance (family Enterobacteriaceae id.3469) | finn-b-N14_CALCUKIDUR | Maximum likelihood | 7 | 0.154 | 0.832 | 1.033 | 0.764 | 1.398 |
| ebi-a-GCST90016936 | Gut microbiota abundance (family Enterobacteriaceae id.3469) | finn-b-N14_CALCUKIDUR | MR Egger | 7 | 1.398 | 0.285 | 0.188 | 0.012 | 2.906 |
| ebi-a-GCST90016936 | Gut microbiota abundance (family Enterobacteriaceae id.3469) | finn-b-N14_CALCUKIDUR | Weighted median | 7 | 0.223 | 0.051 | 1.543 | 0.997 | 2.387 |
| ebi-a-GCST90016937 | Gut microbiota abundance (family Erysipelotrichaceae id.2149) | finn-b-N14_CALCUKIDUR | Inverse variance weighted | 13 | 0.122 | 0.380 | 0.899 | 0.708 | 1.141 |
| ebi-a-GCST90016937 | Gut microbiota abundance (family Erysipelotrichaceae id.2149) | finn-b-N14_CALCUKIDUR | Maximum likelihood | 13 | 0.123 | 0.398 | 0.901 | 0.708 | 1.147 |
| ebi-a-GCST90016937 | Gut microbiota abundance (family Erysipelotrichaceae id.2149) | finn-b-N14_CALCUKIDUR | MR Egger | 13 | 0.527 | 0.476 | 1.475 | 0.525 | 4.142 |
| ebi-a-GCST90016937 | Gut microbiota abundance (family Erysipelotrichaceae id.2149) | finn-b-N14_CALCUKIDUR | Weighted median | 13 | 0.163 | 0.671 | 0.933 | 0.678 | 1.284 |
| ebi-a-GCST90016938 | Gut microbiota abundance (family Family XI id.1936) | finn-b-N14_CALCUKIDUR | Inverse variance weighted | 8 | 0.062 | 0.544 | 0.963 | 0.852 | 1.088 |
| ebi-a-GCST90016938 | Gut microbiota abundance (family Family XI id.1936) | finn-b-N14_CALCUKIDUR | Maximum likelihood | 8 | 0.063 | 0.543 | 0.962 | 0.850 | 1.089 |
| ebi-a-GCST90016938 | Gut microbiota abundance (family Family XI id.1936) | finn-b-N14_CALCUKIDUR | MR Egger | 8 | 0.397 | 0.818 | 1.100 | 0.506 | 2.394 |
| ebi-a-GCST90016938 | Gut microbiota abundance (family Family XI id.1936) | finn-b-N14_CALCUKIDUR | Weighted median | 8 | 0.081 | 0.372 | 0.931 | 0.795 | 1.090 |
| ebi-a-GCST90016939 | Gut microbiota abundance (family Family XIII id.1957) | finn-b-N14_CALCUKIDUR | Inverse variance weighted | 9 | 0.162 | 0.350 | 0.859 | 0.625 | 1.181 |
| ebi-a-GCST90016939 | Gut microbiota abundance (family Family XIII id.1957) | finn-b-N14_CALCUKIDUR | Maximum likelihood | 9 | 0.145 | 0.269 | 0.852 | 0.642 | 1.131 |
| ebi-a-GCST90016939 | Gut microbiota abundance (family Family XIII id.1957) | finn-b-N14_CALCUKIDUR | MR Egger | 9 | 0.605 | 0.434 | 1.652 | 0.504 | 5.412 |
| ebi-a-GCST90016939 | Gut microbiota abundance (family Family XIII id.1957) | finn-b-N14_CALCUKIDUR | Weighted median | 9 | 0.186 | 0.886 | 1.027 | 0.713 | 1.480 |
| ebi-a-GCST90016940 | Gut microbiota abundance (family Lachnospiraceae id.1987) | finn-b-N14_CALCUKIDUR | Inverse variance weighted | 16 | 0.111 | 0.509 | 1.076 | 0.865 | 1.339 |
| ebi-a-GCST90016940 | Gut microbiota abundance (family Lachnospiraceae id.1987) | finn-b-N14_CALCUKIDUR | Maximum likelihood | 16 | 0.113 | 0.496 | 1.080 | 0.865 | 1.349 |
| ebi-a-GCST90016940 | Gut microbiota abundance (family Lachnospiraceae id.1987) | finn-b-N14_CALCUKIDUR | MR Egger | 16 | 0.399 | 0.628 | 1.218 | 0.558 | 2.661 |
| ebi-a-GCST90016940 | Gut microbiota abundance (family Lachnospiraceae id.1987) | finn-b-N14_CALCUKIDUR | Weighted median | 16 | 0.157 | 0.932 | 0.987 | 0.726 | 1.342 |
| ebi-a-GCST90016941 | Gut microbiota abundance (family Lactobacillaceae id.1836) | finn-b-N14_CALCUKIDUR | Inverse variance weighted | 8 | 0.084 | 0.842 | 1.017 | 0.862 | 1.200 |
| ebi-a-GCST90016941 | Gut microbiota abundance (family Lactobacillaceae id.1836) | finn-b-N14_CALCUKIDUR | Maximum likelihood | 8 | 0.086 | 0.839 | 1.018 | 0.860 | 1.204 |
| ebi-a-GCST90016941 | Gut microbiota abundance (family Lactobacillaceae id.1836) | finn-b-N14_CALCUKIDUR | MR Egger | 8 | 0.211 | 0.823 | 0.952 | 0.629 | 1.440 |
| ebi-a-GCST90016941 | Gut microbiota abundance (family Lactobacillaceae id.1836) | finn-b-N14_CALCUKIDUR | Weighted median | 8 | 0.110 | 0.738 | 1.038 | 0.836 | 1.288 |
| ebi-a-GCST90016942 | Gut microbiota abundance (family Methanobacteriaceae id.121) | finn-b-N14_CALCUKIDUR | Inverse variance weighted | 10 | 0.064 | 0.496 | 0.958 | 0.846 | 1.085 |
| ebi-a-GCST90016942 | Gut microbiota abundance (family Methanobacteriaceae id.121) | finn-b-N14_CALCUKIDUR | Maximum likelihood | 10 | 0.065 | 0.488 | 0.956 | 0.843 | 1.085 |
| ebi-a-GCST90016942 | Gut microbiota abundance (family Methanobacteriaceae id.121) | finn-b-N14_CALCUKIDUR | MR Egger | 10 | 0.261 | 0.194 | 1.448 | 0.869 | 2.414 |
| ebi-a-GCST90016942 | Gut microbiota abundance (family Methanobacteriaceae id.121) | finn-b-N14_CALCUKIDUR | Weighted median | 10 | 0.083 | 0.761 | 0.975 | 0.828 | 1.148 |
| ebi-a-GCST90016943 | Gut microbiota abundance (family Oxalobacteraceae id.2966) | finn-b-N14_CALCUKIDUR | Inverse variance weighted | 14 | 0.059 | 0.922 | 1.006 | 0.896 | 1.129 |
| ebi-a-GCST90016943 | Gut microbiota abundance (family Oxalobacteraceae id.2966) | finn-b-N14_CALCUKIDUR | Maximum likelihood | 14 | 0.060 | 0.920 | 1.006 | 0.894 | 1.132 |
| ebi-a-GCST90016943 | Gut microbiota abundance (family Oxalobacteraceae id.2966) | finn-b-N14_CALCUKIDUR | MR Egger | 14 | 0.227 | 0.253 | 0.761 | 0.487 | 1.188 |
| ebi-a-GCST90016943 | Gut microbiota abundance (family Oxalobacteraceae id.2966) | finn-b-N14_CALCUKIDUR | Weighted median | 14 | 0.082 | 0.941 | 1.006 | 0.857 | 1.181 |
| ebi-a-GCST90016944 | Gut microbiota abundance (family Pasteurellaceae id.3689) | finn-b-N14_CALCUKIDUR | Inverse variance weighted | 14 | 0.090 | 0.763 | 1.028 | 0.861 | 1.226 |
| ebi-a-GCST90016944 | Gut microbiota abundance (family Pasteurellaceae id.3689) | finn-b-N14_CALCUKIDUR | Maximum likelihood | 14 | 0.075 | 0.697 | 1.030 | 0.889 | 1.193 |
| ebi-a-GCST90016944 | Gut microbiota abundance (family Pasteurellaceae id.3689) | finn-b-N14_CALCUKIDUR | MR Egger | 14 | 0.195 | 0.447 | 0.858 | 0.585 | 1.258 |
| ebi-a-GCST90016944 | Gut microbiota abundance (family Pasteurellaceae id.3689) | finn-b-N14_CALCUKIDUR | Weighted median | 14 | 0.107 | 0.996 | 0.999 | 0.811 | 1.231 |
| ebi-a-GCST90016945 | Gut microbiota abundance (family Peptococcaceae id.2024) | finn-b-N14_CALCUKIDUR | Inverse variance weighted | 9 | 0.101 | 0.248 | 0.890 | 0.730 | 1.085 |
| ebi-a-GCST90016945 | Gut microbiota abundance (family Peptococcaceae id.2024) | finn-b-N14_CALCUKIDUR | Maximum likelihood | 9 | 0.104 | 0.251 | 0.888 | 0.725 | 1.088 |
| ebi-a-GCST90016945 | Gut microbiota abundance (family Peptococcaceae id.2024) | finn-b-N14_CALCUKIDUR | MR Egger | 9 | 0.280 | 0.779 | 0.922 | 0.533 | 1.594 |
| ebi-a-GCST90016945 | Gut microbiota abundance (family Peptococcaceae id.2024) | finn-b-N14_CALCUKIDUR | Weighted median | 9 | 0.136 | 0.340 | 0.878 | 0.673 | 1.147 |
| ebi-a-GCST90016946 | Gut microbiota abundance (family Peptostreptococcaceae id.2042) | finn-b-N14_CALCUKIDUR | Inverse variance weighted | 13 | 0.095 | 0.265 | 1.112 | 0.923 | 1.341 |
| ebi-a-GCST90016946 | Gut microbiota abundance (family Peptostreptococcaceae id.2042) | finn-b-N14_CALCUKIDUR | Maximum likelihood | 13 | 0.097 | 0.253 | 1.117 | 0.924 | 1.351 |
| ebi-a-GCST90016946 | Gut microbiota abundance (family Peptostreptococcaceae id.2042) | finn-b-N14_CALCUKIDUR | MR Egger | 13 | 0.217 | 0.775 | 0.938 | 0.613 | 1.435 |
| ebi-a-GCST90016946 | Gut microbiota abundance (family Peptostreptococcaceae id.2042) | finn-b-N14_CALCUKIDUR | Weighted median | 13 | 0.130 | 0.537 | 1.084 | 0.839 | 1.399 |
| ebi-a-GCST90016947 | Gut microbiota abundance (family Porphyromonadaceae id.943) | finn-b-N14_CALCUKIDUR | Inverse variance weighted | 9 | 0.151 | 0.834 | 1.032 | 0.768 | 1.388 |
| ebi-a-GCST90016947 | Gut microbiota abundance (family Porphyromonadaceae id.943) | finn-b-N14_CALCUKIDUR | Maximum likelihood | 9 | 0.154 | 0.830 | 1.034 | 0.765 | 1.397 |
| ebi-a-GCST90016947 | Gut microbiota abundance (family Porphyromonadaceae id.943) | finn-b-N14_CALCUKIDUR | MR Egger | 9 | 0.670 | 0.518 | 1.578 | 0.424 | 5.869 |
| ebi-a-GCST90016947 | Gut microbiota abundance (family Porphyromonadaceae id.943) | finn-b-N14_CALCUKIDUR | Weighted median | 9 | 0.196 | 0.205 | 1.282 | 0.873 | 1.883 |
| ebi-a-GCST90016948 | Gut microbiota abundance (family Prevotellaceae id.960) | finn-b-N14_CALCUKIDUR | Inverse variance weighted | 16 | 0.142 | 0.722 | 1.052 | 0.797 | 1.388 |
| ebi-a-GCST90016948 | Gut microbiota abundance (family Prevotellaceae id.960) | finn-b-N14_CALCUKIDUR | Maximum likelihood | 16 | 0.099 | 0.561 | 1.060 | 0.872 | 1.287 |
| ebi-a-GCST90016948 | Gut microbiota abundance (family Prevotellaceae id.960) | finn-b-N14_CALCUKIDUR | MR Egger | 16 | 0.533 | 0.795 | 1.151 | 0.405 | 3.269 |
| ebi-a-GCST90016948 | Gut microbiota abundance (family Prevotellaceae id.960) | finn-b-N14_CALCUKIDUR | Weighted median | 16 | 0.146 | 0.293 | 0.857 | 0.644 | 1.142 |
| ebi-a-GCST90016949 | Gut microbiota abundance (family Rhodospirillaceae id.2717) | finn-b-N14_CALCUKIDUR | Inverse variance weighted | 15 | 0.083 | 0.967 | 1.003 | 0.853 | 1.181 |
| ebi-a-GCST90016949 | Gut microbiota abundance (family Rhodospirillaceae id.2717) | finn-b-N14_CALCUKIDUR | Maximum likelihood | 15 | 0.076 | 0.960 | 1.004 | 0.866 | 1.164 |
| ebi-a-GCST90016949 | Gut microbiota abundance (family Rhodospirillaceae id.2717) | finn-b-N14_CALCUKIDUR | MR Egger | 15 | 0.361 | 0.533 | 0.794 | 0.391 | 1.610 |
| ebi-a-GCST90016949 | Gut microbiota abundance (family Rhodospirillaceae id.2717) | finn-b-N14_CALCUKIDUR | Weighted median | 15 | 0.102 | 0.825 | 1.023 | 0.838 | 1.249 |
| ebi-a-GCST90016950 | Gut microbiota abundance (family Rikenellaceae id.967) | finn-b-N14_CALCUKIDUR | Inverse variance weighted | 17 | 0.099 | 0.216 | 1.130 | 0.931 | 1.373 |
| ebi-a-GCST90016950 | Gut microbiota abundance (family Rikenellaceae id.967) | finn-b-N14_CALCUKIDUR | Maximum likelihood | 17 | 0.101 | 0.207 | 1.136 | 0.932 | 1.385 |
| ebi-a-GCST90016950 | Gut microbiota abundance (family Rikenellaceae id.967) | finn-b-N14_CALCUKIDUR | MR Egger | 17 | 0.308 | 0.744 | 1.108 | 0.606 | 2.024 |
| ebi-a-GCST90016950 | Gut microbiota abundance (family Rikenellaceae id.967) | finn-b-N14_CALCUKIDUR | Weighted median | 17 | 0.136 | 0.677 | 1.058 | 0.810 | 1.383 |
| ebi-a-GCST90016951 | Gut microbiota abundance (family Ruminococcaceae id.2050) | finn-b-N14_CALCUKIDUR | Inverse variance weighted | 9 | 0.122 | 0.157 | 1.188 | 0.936 | 1.509 |
| ebi-a-GCST90016951 | Gut microbiota abundance (family Ruminococcaceae id.2050) | finn-b-N14_CALCUKIDUR | Maximum likelihood | 9 | 0.124 | 0.158 | 1.192 | 0.934 | 1.520 |
| ebi-a-GCST90016951 | Gut microbiota abundance (family Ruminococcaceae id.2050) | finn-b-N14_CALCUKIDUR | MR Egger | 9 | 0.275 | 0.891 | 0.962 | 0.561 | 1.650 |
| ebi-a-GCST90016951 | Gut microbiota abundance (family Ruminococcaceae id.2050) | finn-b-N14_CALCUKIDUR | Weighted median | 9 | 0.154 | 0.205 | 1.216 | 0.899 | 1.645 |
| ebi-a-GCST90016952 | Gut microbiota abundance (family Streptococcaceae id.1850) | finn-b-N14_CALCUKIDUR | Inverse variance weighted | 13 | 0.113 | 0.122 | 1.190 | 0.954 | 1.485 |
| ebi-a-GCST90016952 | Gut microbiota abundance (family Streptococcaceae id.1850) | finn-b-N14_CALCUKIDUR | Maximum likelihood | 13 | 0.115 | 0.113 | 1.200 | 0.958 | 1.503 |
| ebi-a-GCST90016952 | Gut microbiota abundance (family Streptococcaceae id.1850) | finn-b-N14_CALCUKIDUR | MR Egger | 13 | 0.452 | 0.835 | 1.101 | 0.454 | 2.671 |
| ebi-a-GCST90016952 | Gut microbiota abundance (family Streptococcaceae id.1850) | finn-b-N14_CALCUKIDUR | Weighted median | 13 | 0.156 | 0.412 | 1.136 | 0.837 | 1.543 |
| ebi-a-GCST90016956 | Gut microbiota abundance (family Veillonellaceae id.2172) | finn-b-N14_CALCUKIDUR | Inverse variance weighted | 19 | 0.085 | 0.528 | 0.948 | 0.803 | 1.119 |
| ebi-a-GCST90016956 | Gut microbiota abundance (family Veillonellaceae id.2172) | finn-b-N14_CALCUKIDUR | Maximum likelihood | 19 | 0.084 | 0.521 | 0.947 | 0.804 | 1.117 |
| ebi-a-GCST90016956 | Gut microbiota abundance (family Veillonellaceae id.2172) | finn-b-N14_CALCUKIDUR | MR Egger | 19 | 0.166 | 0.340 | 1.177 | 0.850 | 1.628 |
| ebi-a-GCST90016956 | Gut microbiota abundance (family Veillonellaceae id.2172) | finn-b-N14_CALCUKIDUR | Weighted median | 19 | 0.120 | 0.917 | 0.988 | 0.781 | 1.249 |
| ebi-a-GCST90016957 | Gut microbiota abundance (family Verrucomicrobiaceae id.4036) | finn-b-N14_CALCUKIDUR | Inverse variance weighted | 11 | 0.101 | 0.848 | 0.981 | 0.804 | 1.196 |
| ebi-a-GCST90016957 | Gut microbiota abundance (family Verrucomicrobiaceae id.4036) | finn-b-N14_CALCUKIDUR | Maximum likelihood | 11 | 0.103 | 0.851 | 0.981 | 0.802 | 1.199 |
| ebi-a-GCST90016957 | Gut microbiota abundance (family Verrucomicrobiaceae id.4036) | finn-b-N14_CALCUKIDUR | MR Egger | 11 | 0.338 | 0.531 | 1.246 | 0.643 | 2.417 |
| ebi-a-GCST90016957 | Gut microbiota abundance (family Verrucomicrobiaceae id.4036) | finn-b-N14_CALCUKIDUR | Weighted median | 11 | 0.133 | 0.408 | 1.116 | 0.861 | 1.447 |
| ebi-a-GCST90016958 | Gut microbiota abundance (family Victivallaceae id.2255) | finn-b-N14_CALCUKIDUR | Inverse variance weighted | 12 | 0.055 | 0.881 | 0.992 | 0.890 | 1.105 |
| ebi-a-GCST90016958 | Gut microbiota abundance (family Victivallaceae id.2255) | finn-b-N14_CALCUKIDUR | Maximum likelihood | 12 | 0.056 | 0.883 | 0.992 | 0.889 | 1.107 |
| ebi-a-GCST90016958 | Gut microbiota abundance (family Victivallaceae id.2255) | finn-b-N14_CALCUKIDUR | MR Egger | 12 | 0.263 | 0.373 | 0.783 | 0.468 | 1.310 |
| ebi-a-GCST90016958 | Gut microbiota abundance (family Victivallaceae id.2255) | finn-b-N14_CALCUKIDUR | Weighted median | 12 | 0.075 | 0.535 | 0.955 | 0.824 | 1.105 |
| ebi-a-GCST90017090 | Gut microbiota abundance (order Actinomycetales id.420) | finn-b-N14_CALCUKIDUR | Inverse variance weighted | 5 | 0.117 | 0.599 | 0.940 | 0.747 | 1.184 |
| ebi-a-GCST90017090 | Gut microbiota abundance (order Actinomycetales id.420) | finn-b-N14_CALCUKIDUR | Maximum likelihood | 5 | 0.118 | 0.596 | 0.939 | 0.745 | 1.184 |
| ebi-a-GCST90017090 | Gut microbiota abundance (order Actinomycetales id.420) | finn-b-N14_CALCUKIDUR | MR Egger | 5 | 0.268 | 0.332 | 0.734 | 0.434 | 1.241 |
| ebi-a-GCST90017090 | Gut microbiota abundance (order Actinomycetales id.420) | finn-b-N14_CALCUKIDUR | Weighted median | 5 | 0.147 | 0.535 | 0.913 | 0.684 | 1.218 |
| ebi-a-GCST90017091 | Gut microbiota abundance (order Bacillales id.1674) | finn-b-N14_CALCUKIDUR | Inverse variance weighted | 9 | 0.066 | 0.737 | 1.023 | 0.898 | 1.164 |
| ebi-a-GCST90017091 | Gut microbiota abundance (order Bacillales id.1674) | finn-b-N14_CALCUKIDUR | Maximum likelihood | 9 | 0.061 | 0.702 | 1.024 | 0.908 | 1.154 |
| ebi-a-GCST90017091 | Gut microbiota abundance (order Bacillales id.1674) | finn-b-N14_CALCUKIDUR | MR Egger | 9 | 0.305 | 0.928 | 1.029 | 0.566 | 1.872 |
| ebi-a-GCST90017091 | Gut microbiota abundance (order Bacillales id.1674) | finn-b-N14_CALCUKIDUR | Weighted median | 9 | 0.086 | 0.585 | 1.048 | 0.886 | 1.239 |
| ebi-a-GCST90017092 | Gut microbiota abundance (order Bacteroidales id.913) | finn-b-N14_CALCUKIDUR | Inverse variance weighted | 14 | 0.107 | 0.735 | 1.037 | 0.841 | 1.278 |
| ebi-a-GCST90017092 | Gut microbiota abundance (order Bacteroidales id.913) | finn-b-N14_CALCUKIDUR | Maximum likelihood | 14 | 0.108 | 0.731 | 1.038 | 0.839 | 1.284 |
| ebi-a-GCST90017092 | Gut microbiota abundance (order Bacteroidales id.913) | finn-b-N14_CALCUKIDUR | MR Egger | 14 | 0.218 | 0.642 | 1.110 | 0.724 | 1.702 |
| ebi-a-GCST90017092 | Gut microbiota abundance (order Bacteroidales id.913) | finn-b-N14_CALCUKIDUR | Weighted median | 14 | 0.142 | 0.411 | 1.124 | 0.851 | 1.485 |
| ebi-a-GCST90017093 | Gut microbiota abundance (order Bifidobacteriales id.432) | finn-b-N14_CALCUKIDUR | Inverse variance weighted | 12 | 0.113 | 0.165 | 1.169 | 0.938 | 1.458 |
| ebi-a-GCST90017093 | Gut microbiota abundance (order Bifidobacteriales id.432) | finn-b-N14_CALCUKIDUR | Maximum likelihood | 12 | 0.101 | 0.117 | 1.171 | 0.961 | 1.426 |
| ebi-a-GCST90017093 | Gut microbiota abundance (order Bifidobacteriales id.432) | finn-b-N14_CALCUKIDUR | MR Egger | 12 | 0.333 | 0.046 | 2.136 | 1.113 | 4.101 |
| ebi-a-GCST90017093 | Gut microbiota abundance (order Bifidobacteriales id.432) | finn-b-N14_CALCUKIDUR | Weighted median | 12 | 0.140 | 0.102 | 1.257 | 0.955 | 1.653 |
| ebi-a-GCST90017094 | Gut microbiota abundance (order Burkholderiales id.2874) | finn-b-N14_CALCUKIDUR | Inverse variance weighted | 10 | 0.136 | 0.326 | 1.142 | 0.876 | 1.490 |
| ebi-a-GCST90017094 | Gut microbiota abundance (order Burkholderiales id.2874) | finn-b-N14_CALCUKIDUR | Maximum likelihood | 10 | 0.130 | 0.278 | 1.152 | 0.892 | 1.486 |
| ebi-a-GCST90017094 | Gut microbiota abundance (order Burkholderiales id.2874) | finn-b-N14_CALCUKIDUR | MR Egger | 10 | 0.436 | 0.308 | 1.608 | 0.684 | 3.779 |
| ebi-a-GCST90017094 | Gut microbiota abundance (order Burkholderiales id.2874) | finn-b-N14_CALCUKIDUR | Weighted median | 10 | 0.181 | 0.562 | 1.111 | 0.779 | 1.585 |
| ebi-a-GCST90017095 | Gut microbiota abundance (order Clostridiales id.1863) | finn-b-N14_CALCUKIDUR | Inverse variance weighted | 13 | 0.111 | 0.079 | 0.823 | 0.662 | 1.023 |
| ebi-a-GCST90017095 | Gut microbiota abundance (order Clostridiales id.1863) | finn-b-N14_CALCUKIDUR | Maximum likelihood | 13 | 0.115 | 0.093 | 0.824 | 0.658 | 1.032 |
| ebi-a-GCST90017095 | Gut microbiota abundance (order Clostridiales id.1863) | finn-b-N14_CALCUKIDUR | MR Egger | 13 | 0.302 | 0.298 | 1.390 | 0.770 | 2.511 |
| ebi-a-GCST90017095 | Gut microbiota abundance (order Clostridiales id.1863) | finn-b-N14_CALCUKIDUR | Weighted median | 13 | 0.165 | 0.635 | 0.924 | 0.669 | 1.278 |
| ebi-a-GCST90017096 | Gut microbiota abundance (order Coriobacteriales id.810) | finn-b-N14_CALCUKIDUR | Inverse variance weighted | 13 | 0.164 | 0.906 | 1.020 | 0.739 | 1.407 |
| ebi-a-GCST90017096 | Gut microbiota abundance (order Coriobacteriales id.810) | finn-b-N14_CALCUKIDUR | Maximum likelihood | 13 | 0.121 | 0.860 | 1.022 | 0.805 | 1.296 |
| ebi-a-GCST90017096 | Gut microbiota abundance (order Coriobacteriales id.810) | finn-b-N14_CALCUKIDUR | MR Egger | 13 | 0.726 | 0.849 | 1.152 | 0.277 | 4.781 |
| ebi-a-GCST90017096 | Gut microbiota abundance (order Coriobacteriales id.810) | finn-b-N14_CALCUKIDUR | Weighted median | 13 | 0.175 | 0.561 | 1.107 | 0.785 | 1.561 |
| ebi-a-GCST90017097 | Gut microbiota abundance (order Desulfovibrionales id.3156) | finn-b-N14_CALCUKIDUR | Inverse variance weighted | 12 | 0.105 | 0.084 | 0.834 | 0.679 | 1.025 |
| ebi-a-GCST90017097 | Gut microbiota abundance (order Desulfovibrionales id.3156) | finn-b-N14_CALCUKIDUR | Maximum likelihood | 12 | 0.107 | 0.093 | 0.835 | 0.677 | 1.031 |
| ebi-a-GCST90017097 | Gut microbiota abundance (order Desulfovibrionales id.3156) | finn-b-N14_CALCUKIDUR | MR Egger | 12 | 0.273 | 0.260 | 0.722 | 0.423 | 1.233 |
| ebi-a-GCST90017097 | Gut microbiota abundance (order Desulfovibrionales id.3156) | finn-b-N14_CALCUKIDUR | Weighted median | 12 | 0.149 | 0.142 | 0.804 | 0.600 | 1.076 |
| ebi-a-GCST90017098 | Gut microbiota abundance (order Enterobacteriales id.3468) | finn-b-N14_CALCUKIDUR | Inverse variance weighted | 7 | 0.244 | 0.905 | 1.030 | 0.638 | 1.661 |
| ebi-a-GCST90017098 | Gut microbiota abundance (order Enterobacteriales id.3468) | finn-b-N14_CALCUKIDUR | Maximum likelihood | 7 | 0.154 | 0.832 | 1.033 | 0.764 | 1.398 |
| ebi-a-GCST90017098 | Gut microbiota abundance (order Enterobacteriales id.3468) | finn-b-N14_CALCUKIDUR | MR Egger | 7 | 1.398 | 0.285 | 0.188 | 0.012 | 2.906 |
| ebi-a-GCST90017098 | Gut microbiota abundance (order Enterobacteriales id.3468) | finn-b-N14_CALCUKIDUR | Weighted median | 7 | 0.229 | 0.058 | 1.543 | 0.985 | 2.416 |
| ebi-a-GCST90017099 | Gut microbiota abundance (order Erysipelotrichales id.2148) | finn-b-N14_CALCUKIDUR | Inverse variance weighted | 13 | 0.122 | 0.380 | 0.899 | 0.708 | 1.141 |
| ebi-a-GCST90017099 | Gut microbiota abundance (order Erysipelotrichales id.2148) | finn-b-N14_CALCUKIDUR | Maximum likelihood | 13 | 0.123 | 0.398 | 0.901 | 0.708 | 1.147 |
| ebi-a-GCST90017099 | Gut microbiota abundance (order Erysipelotrichales id.2148) | finn-b-N14_CALCUKIDUR | MR Egger | 13 | 0.527 | 0.476 | 1.475 | 0.525 | 4.142 |
| ebi-a-GCST90017099 | Gut microbiota abundance (order Erysipelotrichales id.2148) | finn-b-N14_CALCUKIDUR | Weighted median | 13 | 0.153 | 0.651 | 0.933 | 0.691 | 1.259 |
| ebi-a-GCST90017100 | Gut microbiota abundance (order Gastranaerophilales id.1591) | finn-b-N14_CALCUKIDUR | Inverse variance weighted | 9 | 0.082 | 0.410 | 0.935 | 0.796 | 1.097 |
| ebi-a-GCST90017100 | Gut microbiota abundance (order Gastranaerophilales id.1591) | finn-b-N14_CALCUKIDUR | Maximum likelihood | 9 | 0.083 | 0.406 | 0.933 | 0.793 | 1.098 |
| ebi-a-GCST90017100 | Gut microbiota abundance (order Gastranaerophilales id.1591) | finn-b-N14_CALCUKIDUR | MR Egger | 9 | 0.244 | 0.336 | 1.287 | 0.798 | 2.077 |
| ebi-a-GCST90017100 | Gut microbiota abundance (order Gastranaerophilales id.1591) | finn-b-N14_CALCUKIDUR | Weighted median | 9 | 0.107 | 0.624 | 0.949 | 0.769 | 1.171 |
| ebi-a-GCST90017101 | Gut microbiota abundance (order Lactobacillales id.1800) | finn-b-N14_CALCUKIDUR | Inverse variance weighted | 15 | 0.105 | 0.831 | 1.023 | 0.833 | 1.255 |
| ebi-a-GCST90017101 | Gut microbiota abundance (order Lactobacillales id.1800) | finn-b-N14_CALCUKIDUR | Maximum likelihood | 15 | 0.107 | 0.824 | 1.024 | 0.831 | 1.262 |
| ebi-a-GCST90017101 | Gut microbiota abundance (order Lactobacillales id.1800) | finn-b-N14_CALCUKIDUR | MR Egger | 15 | 0.270 | 0.233 | 0.714 | 0.421 | 1.211 |
| ebi-a-GCST90017101 | Gut microbiota abundance (order Lactobacillales id.1800) | finn-b-N14_CALCUKIDUR | Weighted median | 15 | 0.134 | 0.326 | 1.140 | 0.877 | 1.483 |
| ebi-a-GCST90017102 | Gut microbiota abundance (order Methanobacteriales id.120) | finn-b-N14_CALCUKIDUR | Inverse variance weighted | 10 | 0.064 | 0.496 | 0.958 | 0.846 | 1.085 |
| ebi-a-GCST90017102 | Gut microbiota abundance (order Methanobacteriales id.120) | finn-b-N14_CALCUKIDUR | Maximum likelihood | 10 | 0.065 | 0.488 | 0.956 | 0.843 | 1.085 |
| ebi-a-GCST90017102 | Gut microbiota abundance (order Methanobacteriales id.120) | finn-b-N14_CALCUKIDUR | MR Egger | 10 | 0.261 | 0.194 | 1.448 | 0.869 | 2.414 |
| ebi-a-GCST90017102 | Gut microbiota abundance (order Methanobacteriales id.120) | finn-b-N14_CALCUKIDUR | Weighted median | 10 | 0.083 | 0.760 | 0.975 | 0.829 | 1.147 |
| ebi-a-GCST90017103 | Gut microbiota abundance (order Mollicutes RF9 id.11579) | finn-b-N14_CALCUKIDUR | Inverse variance weighted | 13 | 0.088 | 0.987 | 1.001 | 0.842 | 1.191 |
| ebi-a-GCST90017103 | Gut microbiota abundance (order Mollicutes RF9 id.11579) | finn-b-N14_CALCUKIDUR | Maximum likelihood | 13 | 0.090 | 0.987 | 1.001 | 0.840 | 1.194 |
| ebi-a-GCST90017103 | Gut microbiota abundance (order Mollicutes RF9 id.11579) | finn-b-N14_CALCUKIDUR | MR Egger | 13 | 0.278 | 0.649 | 1.139 | 0.660 | 1.965 |
| ebi-a-GCST90017103 | Gut microbiota abundance (order Mollicutes RF9 id.11579) | finn-b-N14_CALCUKIDUR | Weighted median | 13 | 0.117 | 0.798 | 1.030 | 0.819 | 1.297 |
| ebi-a-GCST90017104 | Gut microbiota abundance (order NB1n id.3953) | finn-b-N14_CALCUKIDUR | Inverse variance weighted | 13 | 0.062 | 0.003 | 0.833 | 0.737 | 0.940 |
| ebi-a-GCST90017104 | Gut microbiota abundance (order NB1n id.3953) | finn-b-N14_CALCUKIDUR | Maximum likelihood | 13 | 0.064 | 0.003 | 0.827 | 0.730 | 0.938 |
| ebi-a-GCST90017104 | Gut microbiota abundance (order NB1n id.3953) | finn-b-N14_CALCUKIDUR | MR Egger | 13 | 0.273 | 0.865 | 0.954 | 0.558 | 1.629 |
| ebi-a-GCST90017104 | Gut microbiota abundance (order NB1n id.3953) | finn-b-N14_CALCUKIDUR | Weighted median | 13 | 0.089 | 0.110 | 0.868 | 0.729 | 1.033 |
| ebi-a-GCST90017105 | Gut microbiota abundance (order Pasteurellales id.3688) | finn-b-N14_CALCUKIDUR | Inverse variance weighted | 14 | 0.090 | 0.763 | 1.028 | 0.861 | 1.226 |
| ebi-a-GCST90017105 | Gut microbiota abundance (order Pasteurellales id.3688) | finn-b-N14_CALCUKIDUR | Maximum likelihood | 14 | 0.075 | 0.697 | 1.030 | 0.889 | 1.193 |
| ebi-a-GCST90017105 | Gut microbiota abundance (order Pasteurellales id.3688) | finn-b-N14_CALCUKIDUR | MR Egger | 14 | 0.195 | 0.447 | 0.858 | 0.585 | 1.258 |
| ebi-a-GCST90017105 | Gut microbiota abundance (order Pasteurellales id.3688) | finn-b-N14_CALCUKIDUR | Weighted median | 14 | 0.100 | 0.995 | 0.999 | 0.821 | 1.216 |
| ebi-a-GCST90017106 | Gut microbiota abundance (order Rhodospirillales id.2667) | finn-b-N14_CALCUKIDUR | Inverse variance weighted | 14 | 0.075 | 0.252 | 1.090 | 0.940 | 1.264 |
| ebi-a-GCST90017106 | Gut microbiota abundance (order Rhodospirillales id.2667) | finn-b-N14_CALCUKIDUR | Maximum likelihood | 14 | 0.078 | 0.244 | 1.095 | 0.940 | 1.275 |
| ebi-a-GCST90017106 | Gut microbiota abundance (order Rhodospirillales id.2667) | finn-b-N14_CALCUKIDUR | MR Egger | 14 | 0.306 | 0.449 | 0.787 | 0.432 | 1.434 |
| ebi-a-GCST90017106 | Gut microbiota abundance (order Rhodospirillales id.2667) | finn-b-N14_CALCUKIDUR | Weighted median | 14 | 0.105 | 0.735 | 1.036 | 0.843 | 1.273 |
| ebi-a-GCST90017107 | Gut microbiota abundance (order Selenomonadales id.2165) | finn-b-N14_CALCUKIDUR | Inverse variance weighted | 12 | 0.121 | 0.822 | 0.973 | 0.767 | 1.235 |
| ebi-a-GCST90017107 | Gut microbiota abundance (order Selenomonadales id.2165) | finn-b-N14_CALCUKIDUR | Maximum likelihood | 12 | 0.124 | 0.824 | 0.973 | 0.763 | 1.240 |
| ebi-a-GCST90017107 | Gut microbiota abundance (order Selenomonadales id.2165) | finn-b-N14_CALCUKIDUR | MR Egger | 12 | 0.418 | 0.314 | 0.642 | 0.283 | 1.456 |
| ebi-a-GCST90017107 | Gut microbiota abundance (order Selenomonadales id.2165) | finn-b-N14_CALCUKIDUR | Weighted median | 12 | 0.177 | 0.727 | 0.940 | 0.665 | 1.330 |
| ebi-a-GCST90017108 | Gut microbiota abundance (order Verrucomicrobiales id.4030) | finn-b-N14_CALCUKIDUR | Inverse variance weighted | 11 | 0.101 | 0.848 | 0.981 | 0.804 | 1.196 |
| ebi-a-GCST90017108 | Gut microbiota abundance (order Verrucomicrobiales id.4030) | finn-b-N14_CALCUKIDUR | Maximum likelihood | 11 | 0.103 | 0.852 | 0.981 | 0.802 | 1.199 |
| ebi-a-GCST90017108 | Gut microbiota abundance (order Verrucomicrobiales id.4030) | finn-b-N14_CALCUKIDUR | MR Egger | 11 | 0.338 | 0.531 | 1.246 | 0.643 | 2.417 |
| ebi-a-GCST90017108 | Gut microbiota abundance (order Verrucomicrobiales id.4030) | finn-b-N14_CALCUKIDUR | Weighted median | 11 | 0.138 | 0.427 | 1.116 | 0.851 | 1.463 |
| ebi-a-GCST90017109 | Gut microbiota abundance (order Victivallales id.2254) | finn-b-N14_CALCUKIDUR | Inverse variance weighted | 8 | 0.091 | 0.416 | 0.929 | 0.777 | 1.110 |
| ebi-a-GCST90017109 | Gut microbiota abundance (order Victivallales id.2254) | finn-b-N14_CALCUKIDUR | Maximum likelihood | 8 | 0.079 | 0.349 | 0.928 | 0.795 | 1.084 |
| ebi-a-GCST90017109 | Gut microbiota abundance (order Victivallales id.2254) | finn-b-N14_CALCUKIDUR | MR Egger | 8 | 0.349 | 0.777 | 0.902 | 0.455 | 1.787 |
| ebi-a-GCST90017109 | Gut microbiota abundance (order Victivallales id.2254) | finn-b-N14_CALCUKIDUR | Weighted median | 8 | 0.104 | 0.446 | 0.924 | 0.754 | 1.133 |
| ebi-a-GCST90017110 | Gut microbiota abundance (phylum Actinobacteria id.400) | finn-b-N14_CALCUKIDUR | Inverse variance weighted | 15 | 0.106 | 0.579 | 0.943 | 0.765 | 1.161 |
| ebi-a-GCST90017110 | Gut microbiota abundance (phylum Actinobacteria id.400) | finn-b-N14_CALCUKIDUR | Maximum likelihood | 15 | 0.108 | 0.569 | 0.940 | 0.760 | 1.163 |
| ebi-a-GCST90017110 | Gut microbiota abundance (phylum Actinobacteria id.400) | finn-b-N14_CALCUKIDUR | MR Egger | 15 | 0.451 | 0.058 | 2.547 | 1.053 | 6.159 |
| ebi-a-GCST90017110 | Gut microbiota abundance (phylum Actinobacteria id.400) | finn-b-N14_CALCUKIDUR | Weighted median | 15 | 0.152 | 0.990 | 0.998 | 0.741 | 1.345 |
| ebi-a-GCST90017111 | Gut microbiota abundance (phylum Bacteroidetes id.905) | finn-b-N14_CALCUKIDUR | Inverse variance weighted | 11 | 0.125 | 0.489 | 1.091 | 0.853 | 1.394 |
| ebi-a-GCST90017111 | Gut microbiota abundance (phylum Bacteroidetes id.905) | finn-b-N14_CALCUKIDUR | Maximum likelihood | 11 | 0.126 | 0.478 | 1.094 | 0.854 | 1.401 |
| ebi-a-GCST90017111 | Gut microbiota abundance (phylum Bacteroidetes id.905) | finn-b-N14_CALCUKIDUR | MR Egger | 11 | 0.285 | 0.144 | 1.578 | 0.902 | 2.761 |
| ebi-a-GCST90017111 | Gut microbiota abundance (phylum Bacteroidetes id.905) | finn-b-N14_CALCUKIDUR | Weighted median | 11 | 0.158 | 0.206 | 1.221 | 0.896 | 1.665 |
| ebi-a-GCST90017112 | Gut microbiota abundance (phylum Cyanobacteria id.1500) | finn-b-N14_CALCUKIDUR | Inverse variance weighted | 8 | 0.093 | 0.658 | 0.959 | 0.799 | 1.152 |
| ebi-a-GCST90017112 | Gut microbiota abundance (phylum Cyanobacteria id.1500) | finn-b-N14_CALCUKIDUR | Maximum likelihood | 8 | 0.095 | 0.663 | 0.960 | 0.797 | 1.155 |
| ebi-a-GCST90017112 | Gut microbiota abundance (phylum Cyanobacteria id.1500) | finn-b-N14_CALCUKIDUR | MR Egger | 8 | 0.333 | 0.886 | 0.952 | 0.496 | 1.826 |
| ebi-a-GCST90017112 | Gut microbiota abundance (phylum Cyanobacteria id.1500) | finn-b-N14_CALCUKIDUR | Weighted median | 8 | 0.122 | 0.729 | 0.959 | 0.754 | 1.218 |
| ebi-a-GCST90017113 | Gut microbiota abundance (phylum Euryarchaeota id.55) | finn-b-N14_CALCUKIDUR | Inverse variance weighted | 12 | 0.057 | 0.556 | 1.034 | 0.925 | 1.155 |
| ebi-a-GCST90017113 | Gut microbiota abundance (phylum Euryarchaeota id.55) | finn-b-N14_CALCUKIDUR | Maximum likelihood | 12 | 0.058 | 0.549 | 1.035 | 0.925 | 1.159 |
| ebi-a-GCST90017113 | Gut microbiota abundance (phylum Euryarchaeota id.55) | finn-b-N14_CALCUKIDUR | MR Egger | 12 | 0.250 | 0.246 | 1.361 | 0.833 | 2.222 |
| ebi-a-GCST90017113 | Gut microbiota abundance (phylum Euryarchaeota id.55) | finn-b-N14_CALCUKIDUR | Weighted median | 12 | 0.076 | 0.143 | 1.117 | 0.963 | 1.296 |
| ebi-a-GCST90017114 | Gut microbiota abundance (phylum Firmicutes id.1672) | finn-b-N14_CALCUKIDUR | Inverse variance weighted | 15 | 0.099 | 0.652 | 0.956 | 0.787 | 1.162 |
| ebi-a-GCST90017114 | Gut microbiota abundance (phylum Firmicutes id.1672) | finn-b-N14_CALCUKIDUR | Maximum likelihood | 15 | 0.101 | 0.648 | 0.955 | 0.783 | 1.164 |
| ebi-a-GCST90017114 | Gut microbiota abundance (phylum Firmicutes id.1672) | finn-b-N14_CALCUKIDUR | MR Egger | 15 | 0.233 | 0.831 | 1.052 | 0.667 | 1.659 |
| ebi-a-GCST90017114 | Gut microbiota abundance (phylum Firmicutes id.1672) | finn-b-N14_CALCUKIDUR | Weighted median | 15 | 0.136 | 0.725 | 0.953 | 0.731 | 1.244 |
| ebi-a-GCST90017115 | Gut microbiota abundance (phylum Lentisphaerae id.2238) | finn-b-N14_CALCUKIDUR | Inverse variance weighted | 9 | 0.080 | 0.314 | 0.923 | 0.789 | 1.079 |
| ebi-a-GCST90017115 | Gut microbiota abundance (phylum Lentisphaerae id.2238) | finn-b-N14_CALCUKIDUR | Maximum likelihood | 9 | 0.074 | 0.260 | 0.920 | 0.796 | 1.064 |
| ebi-a-GCST90017115 | Gut microbiota abundance (phylum Lentisphaerae id.2238) | finn-b-N14_CALCUKIDUR | MR Egger | 9 | 0.323 | 0.682 | 0.871 | 0.463 | 1.640 |
| ebi-a-GCST90017115 | Gut microbiota abundance (phylum Lentisphaerae id.2238) | finn-b-N14_CALCUKIDUR | Weighted median | 9 | 0.097 | 0.356 | 0.914 | 0.756 | 1.106 |
| ebi-a-GCST90017116 | Gut microbiota abundance (phylum Proteobacteria id.2375) | finn-b-N14_CALCUKIDUR | Inverse variance weighted | 12 | 0.118 | 0.536 | 1.076 | 0.853 | 1.356 |
| ebi-a-GCST90017116 | Gut microbiota abundance (phylum Proteobacteria id.2375) | finn-b-N14_CALCUKIDUR | Maximum likelihood | 12 | 0.119 | 0.515 | 1.081 | 0.856 | 1.365 |
| ebi-a-GCST90017116 | Gut microbiota abundance (phylum Proteobacteria id.2375) | finn-b-N14_CALCUKIDUR | MR Egger | 12 | 0.337 | 0.654 | 0.856 | 0.442 | 1.656 |
| ebi-a-GCST90017116 | Gut microbiota abundance (phylum Proteobacteria id.2375) | finn-b-N14_CALCUKIDUR | Weighted median | 12 | 0.162 | 0.731 | 1.057 | 0.770 | 1.451 |
| ebi-a-GCST90017117 | Gut microbiota abundance (phylum Tenericutes id.3919) | finn-b-N14_CALCUKIDUR | Inverse variance weighted | 12 | 0.096 | 0.319 | 0.909 | 0.752 | 1.097 |
| ebi-a-GCST90017117 | Gut microbiota abundance (phylum Tenericutes id.3919) | finn-b-N14_CALCUKIDUR | Maximum likelihood | 12 | 0.097 | 0.342 | 0.912 | 0.753 | 1.103 |
| ebi-a-GCST90017117 | Gut microbiota abundance (phylum Tenericutes id.3919) | finn-b-N14_CALCUKIDUR | MR Egger | 12 | 0.327 | 0.772 | 0.907 | 0.478 | 1.723 |
| ebi-a-GCST90017117 | Gut microbiota abundance (phylum Tenericutes id.3919) | finn-b-N14_CALCUKIDUR | Weighted median | 12 | 0.128 | 0.787 | 0.966 | 0.752 | 1.241 |
| ebi-a-GCST90017118 | Gut microbiota abundance (phylum Verrucomicrobia id.3982) | finn-b-N14_CALCUKIDUR | Inverse variance weighted | 12 | 0.096 | 0.378 | 1.088 | 0.902 | 1.313 |
| ebi-a-GCST90017118 | Gut microbiota abundance (phylum Verrucomicrobia id.3982) | finn-b-N14_CALCUKIDUR | Maximum likelihood | 12 | 0.097 | 0.365 | 1.092 | 0.903 | 1.321 |
| ebi-a-GCST90017118 | Gut microbiota abundance (phylum Verrucomicrobia id.3982) | finn-b-N14_CALCUKIDUR | MR Egger | 12 | 0.251 | 0.670 | 1.116 | 0.682 | 1.826 |
| ebi-a-GCST90017118 | Gut microbiota abundance (phylum Verrucomicrobia id.3982) | finn-b-N14_CALCUKIDUR | Weighted median | 12 | 0.132 | 0.335 | 1.135 | 0.877 | 1.469 |

| Supplementary Table 5: Correlation disease or trait of SNPs in significant IVW results retrievaled by PhenoScanner. | | | |
| --- | --- | --- | --- |
| snp | a1 | a2 | trait |
| rs2300774 | G | A | High light scatter percentage of red cells |
| rs2300774 | G | A | Mean corpuscular hemoglobin |
| rs2300774 | G | A | Mean corpuscular hemoglobin concentration |
| rs2300774 | G | A | Mean corpuscular volume |
| rs2300774 | G | A | Platelet count |
| rs2300774 | G | A | Plateletcrit |
| rs2300774 | G | A | Red blood cell count |
| rs2300774 | G | A | Red cell distribution width |
| rs2300774 | G | A | Reticulocyte fraction of red cells |
| rs2300774 | G | A | Mean corpuscular hemoglobin concentration |
| rs2300774 | G | A | Mean corpuscular volume |
| rs2300774 | G | A | Mean corpuscular volume |
| rs8003149 | C | T | Body mass index |
| rs8003149 | C | T | Diastolic blood pressure |
| rs8003149 | C | T | Height |
| rs8003149 | C | T | Impedance of arm left |
| rs8003149 | C | T | Impedance of arm right |
| rs8003149 | C | T | Impedance of whole body |
| rs2889192 | G | T | mDC:%32+; mDC subset (CD32+) |
| rs2838334 | A | G | Mean corpuscular hemoglobin |
| rs2838334 | A | G | Mean corpuscular volume |
| rs2838334 | A | G | Red blood cell count |
| rs2838334 | A | G | Coronary artery disease |
| rs6058181 | C | T | Height |
| rs6058181 | C | T | Height |
| rs6058181 | C | T | Plasma protein C levels |
| rs6058181 | C | T | Prothrombin time by INR international normalized ratio |
| rs6058181 | C | T | Arm fat-free mass left |
| rs6058181 | C | T | Arm fat-free mass right |
| rs6058181 | C | T | Arm predicted mass left |
| rs6058181 | C | T | Arm predicted mass right |
| rs6058181 | C | T | Basal metabolic rate |
| rs6058181 | C | T | Cause of death: subarachnoid haemorrhage from intracranial artery, unspecified |
| rs6058181 | C | T | Comparative height size at age 10 |
| rs6058181 | C | T | Creatinine in urine |
| rs6058181 | C | T | Height |
| rs6058181 | C | T | Leg fat-free mass left |
| rs6058181 | C | T | Leg fat-free mass right |
| rs6058181 | C | T | Leg predicted mass left |
| rs6058181 | C | T | Leg predicted mass right |
| rs6058181 | C | T | Self-reported deep venous thrombosis |
| rs6058181 | C | T | Sitting height |
| rs6058181 | C | T | Trunk fat-free mass |
| rs6058181 | C | T | Trunk predicted mass |
| rs6058181 | C | T | Whole body fat-free mass |
| rs6058181 | C | T | Whole body water mass |
| rs6910935 | A | G | Treatment with cetraben emollient cream |
| rs9536330 | C | T | Forced vital capacity, best measure |
| rs10497836 | C | T | Arm fat-free mass left |
| rs10497836 | C | T | Arm fat-free mass right |
| rs10497836 | C | T | Arm predicted mass left |
| rs10497836 | C | T | Arm predicted mass right |
| rs10497836 | C | T | Impedance of arm left |
| rs10497836 | C | T | Impedance of arm right |
| rs2171249 | C | T | Asthma 18 years old |
| rs3761728 | G | T | Arm fat mass left |
| rs3761728 | G | T | Arm fat mass right |
| rs3761728 | G | T | Arm fat-free mass left |
| rs3761728 | G | T | Arm fat-free mass right |
| rs3761728 | G | T | Arm predicted mass left |
| rs3761728 | G | T | Arm predicted mass right |
| rs3761728 | G | T | Basal metabolic rate |
| rs3761728 | G | T | Body fat percentage |
| rs3761728 | G | T | Comparative height size at age 10 |
| rs3761728 | G | T | Height |
| rs3761728 | G | T | Hip circumference |
| rs3761728 | G | T | Leg fat mass left |
| rs3761728 | G | T | Leg fat mass right |
| rs3761728 | G | T | Leg fat-free mass left |
| rs3761728 | G | T | Leg fat-free mass right |
| rs3761728 | G | T | Leg predicted mass left |
| rs3761728 | G | T | Leg predicted mass right |
| rs3761728 | G | T | Systolic blood pressure |
| rs3761728 | G | T | Trunk fat mass |
| rs3761728 | G | T | Trunk fat percentage |
| rs3761728 | G | T | Trunk fat-free mass |
| rs3761728 | G | T | Trunk predicted mass |
| rs3761728 | G | T | Waist circumference |
| rs3761728 | G | T | Weight |
| rs3761728 | G | T | Whole body fat mass |
| rs3761728 | G | T | Whole body fat-free mass |
| rs3761728 | G | T | Whole body water mass |
| rs4347804 | A | G | Self-reported thalassaemia |
| rs11054680 | C | T | Heel bone mineral density left |
| rs3734633 | G | A | Arm fat-free mass left |
| rs3734633 | G | A | Arm fat-free mass right |
| rs3734633 | G | A | Arm predicted mass left |
| rs3734633 | G | A | Arm predicted mass right |
| rs3734633 | G | A | Basal metabolic rate |
| rs3734633 | G | A | Leg fat-free mass left |
| rs3734633 | G | A | Leg fat-free mass right |
| rs3734633 | G | A | Leg predicted mass left |
| rs3734633 | G | A | Leg predicted mass right |
| rs3734633 | G | A | Weight |
| rs3734633 | G | A | Whole body fat-free mass |
| rs3734633 | G | A | Whole body water mass |
| rs55756211 | C | T | Pulse rate |
| rs55756211 | C | T | Relative age of first facial hair |

| Supplementary Table 6: IVs in reverse MR analysis. | | | | | | | | |
| --- | --- | --- | --- | --- | --- | --- | --- | --- |
| SNP | effect_allele.exposure | other_allele.exposure | eaf.exposure | se.exposure | beta.exposure | pval.exposure | R2 | F |
| rs6667242 | G | A | 0.126 | 0.032 | 0.200 | 5.38E-10 | 0.009 | 1935.484 |
| rs2924808 | C | G | 0.366 | 0.022 | 0.138 | 3.92E-10 | 0.009 | 1935.054 |
| rs71606723 | T | A | 0.339 | 0.023 | 0.133 | 3.31E-09 | 0.008 | 1745.290 |
| rs10051765 | C | T | 0.401 | 0.022 | 0.143 | 3.53E-11 | 0.010 | 2160.595 |
| rs1010269 | G | A | 0.820 | 0.028 | 0.156 | 1.95E-08 | 0.007 | 1579.072 |
| rs2585442 | G | C | 0.319 | 0.023 | 0.133 | 5.37E-09 | 0.008 | 1685.912 |
| rs2776288 | G | A | 0.350 | 0.022 | -0.127 | 1.19E-08 | 0.007 | 1612.790 |
| rs9608071 | T | G | 0.200 | 0.027 | 0.153 | 6.84E-09 | 0.008 | 1658.394 |
| rs74780677 | G | A | 0.073 | 0.042 | -0.246 | 4.18E-09 | 0.008 | 1795.876 |

| Supplementary Table 7: All results of reverse MR analysIs | | | | | | | |
| --- | --- | --- | --- | --- | --- | --- | --- |
| id.exposure | id.outcome | outcome | method | pval | or | or_lci95 | or_uci95 |
| finn-b-N14_CALCUKIDUR | ebi-a-GCST90016908 | class Actinobacteria id.419 | Inverse variance weighted | 0.902 | 1.004 | 0.944 | 1.068 |
| finn-b-N14_CALCUKIDUR | ebi-a-GCST90016908 | class Actinobacteria id.419 | Maximum likelihood | 0.900 | 1.004 | 0.943 | 1.069 |
| finn-b-N14_CALCUKIDUR | ebi-a-GCST90016908 | class Actinobacteria id.419 | MR Egger | 0.524 | 1.196 | 0.712 | 2.007 |
| finn-b-N14_CALCUKIDUR | ebi-a-GCST90016908 | class Actinobacteria id.419 | Weighted median | 0.746 | 0.987 | 0.909 | 1.071 |
| finn-b-N14_CALCUKIDUR | ebi-a-GCST90016909 | class Alphaproteobacteria id.2379 | Inverse variance weighted | 0.962 | 1.002 | 0.925 | 1.086 |
| finn-b-N14_CALCUKIDUR | ebi-a-GCST90016909 | class Alphaproteobacteria id.2379 | Maximum likelihood | 0.962 | 1.002 | 0.924 | 1.086 |
| finn-b-N14_CALCUKIDUR | ebi-a-GCST90016909 | class Alphaproteobacteria id.2379 | MR Egger | 0.478 | 0.776 | 0.402 | 1.496 |
| finn-b-N14_CALCUKIDUR | ebi-a-GCST90016909 | class Alphaproteobacteria id.2379 | Weighted median | 0.981 | 0.999 | 0.904 | 1.103 |
| finn-b-N14_CALCUKIDUR | ebi-a-GCST90016910 | class Bacilli id.1673 | Inverse variance weighted | 0.282 | 1.033 | 0.973 | 1.097 |
| finn-b-N14_CALCUKIDUR | ebi-a-GCST90016910 | class Bacilli id.1673 | Maximum likelihood | 0.284 | 1.034 | 0.973 | 1.098 |
| finn-b-N14_CALCUKIDUR | ebi-a-GCST90016910 | class Bacilli id.1673 | MR Egger | 0.320 | 1.306 | 0.806 | 2.116 |
| finn-b-N14_CALCUKIDUR | ebi-a-GCST90016910 | class Bacilli id.1673 | Weighted median | 0.425 | 1.032 | 0.955 | 1.117 |
| finn-b-N14_CALCUKIDUR | ebi-a-GCST90016911 | class Bacteroidia id.912 | Inverse variance weighted | 0.110 | 0.954 | 0.900 | 1.011 |
| finn-b-N14_CALCUKIDUR | ebi-a-GCST90016911 | class Bacteroidia id.912 | Maximum likelihood | 0.108 | 0.953 | 0.898 | 1.011 |
| finn-b-N14_CALCUKIDUR | ebi-a-GCST90016911 | class Bacteroidia id.912 | MR Egger | 0.613 | 0.880 | 0.551 | 1.406 |
| finn-b-N14_CALCUKIDUR | ebi-a-GCST90016911 | class Bacteroidia id.912 | Weighted median | 0.134 | 0.942 | 0.871 | 1.019 |
| finn-b-N14_CALCUKIDUR | ebi-a-GCST90016912 | class Betaproteobacteria id.2867 | Inverse variance weighted | 0.640 | 1.014 | 0.955 | 1.077 |
| finn-b-N14_CALCUKIDUR | ebi-a-GCST90016912 | class Betaproteobacteria id.2867 | Maximum likelihood | 0.637 | 1.015 | 0.955 | 1.078 |
| finn-b-N14_CALCUKIDUR | ebi-a-GCST90016912 | class Betaproteobacteria id.2867 | MR Egger | 0.332 | 1.297 | 0.800 | 2.104 |
| finn-b-N14_CALCUKIDUR | ebi-a-GCST90016912 | class Betaproteobacteria id.2867 | Weighted median | 0.580 | 0.979 | 0.907 | 1.056 |
| finn-b-N14_CALCUKIDUR | ebi-a-GCST90016913 | class Clostridia id.1859 | Inverse variance weighted | 0.652 | 1.017 | 0.945 | 1.095 |
| finn-b-N14_CALCUKIDUR | ebi-a-GCST90016913 | class Clostridia id.1859 | Maximum likelihood | 0.559 | 1.018 | 0.959 | 1.080 |
| finn-b-N14_CALCUKIDUR | ebi-a-GCST90016913 | class Clostridia id.1859 | MR Egger | 0.514 | 0.805 | 0.435 | 1.488 |
| finn-b-N14_CALCUKIDUR | ebi-a-GCST90016913 | class Clostridia id.1859 | Weighted median | 0.230 | 1.048 | 0.971 | 1.131 |
| finn-b-N14_CALCUKIDUR | ebi-a-GCST90016914 | class Coriobacteriia id.809 | Inverse variance weighted | 0.699 | 0.988 | 0.931 | 1.049 |
| finn-b-N14_CALCUKIDUR | ebi-a-GCST90016914 | class Coriobacteriia id.809 | Maximum likelihood | 0.695 | 0.988 | 0.931 | 1.049 |
| finn-b-N14_CALCUKIDUR | ebi-a-GCST90016914 | class Coriobacteriia id.809 | MR Egger | 0.278 | 1.337 | 0.830 | 2.156 |
| finn-b-N14_CALCUKIDUR | ebi-a-GCST90016914 | class Coriobacteriia id.809 | Weighted median | 0.891 | 1.005 | 0.930 | 1.087 |
| finn-b-N14_CALCUKIDUR | ebi-a-GCST90016915 | class Deltaproteobacteria id.3087 | Inverse variance weighted | 0.575 | 1.019 | 0.955 | 1.086 |
| finn-b-N14_CALCUKIDUR | ebi-a-GCST90016915 | class Deltaproteobacteria id.3087 | Maximum likelihood | 0.574 | 1.019 | 0.955 | 1.086 |
| finn-b-N14_CALCUKIDUR | ebi-a-GCST90016915 | class Deltaproteobacteria id.3087 | MR Egger | 0.641 | 0.879 | 0.525 | 1.472 |
| finn-b-N14_CALCUKIDUR | ebi-a-GCST90016915 | class Deltaproteobacteria id.3087 | Weighted median | 0.565 | 1.023 | 0.947 | 1.104 |
| finn-b-N14_CALCUKIDUR | ebi-a-GCST90016916 | class Erysipelotrichia id.2147 | Inverse variance weighted | 0.956 | 0.997 | 0.911 | 1.092 |
| finn-b-N14_CALCUKIDUR | ebi-a-GCST90016916 | class Erysipelotrichia id.2147 | Maximum likelihood | 0.929 | 0.997 | 0.939 | 1.059 |
| finn-b-N14_CALCUKIDUR | ebi-a-GCST90016916 | class Erysipelotrichia id.2147 | MR Egger | 0.495 | 0.757 | 0.356 | 1.607 |
| finn-b-N14_CALCUKIDUR | ebi-a-GCST90016916 | class Erysipelotrichia id.2147 | Weighted median | 0.994 | 1.000 | 0.924 | 1.082 |
| finn-b-N14_CALCUKIDUR | ebi-a-GCST90016917 | class Gammaproteobacteria id.3303 | Inverse variance weighted | 0.571 | 1.018 | 0.957 | 1.084 |
| finn-b-N14_CALCUKIDUR | ebi-a-GCST90016917 | class Gammaproteobacteria id.3303 | Maximum likelihood | 0.568 | 1.018 | 0.956 | 1.085 |
| finn-b-N14_CALCUKIDUR | ebi-a-GCST90016917 | class Gammaproteobacteria id.3303 | MR Egger | 0.421 | 0.802 | 0.487 | 1.323 |
| finn-b-N14_CALCUKIDUR | ebi-a-GCST90016917 | class Gammaproteobacteria id.3303 | Weighted median | 0.829 | 1.009 | 0.931 | 1.093 |
| finn-b-N14_CALCUKIDUR | ebi-a-GCST90016918 | class Lentisphaeria id.2250 | Inverse variance weighted | 0.202 | 1.089 | 0.955 | 1.242 |
| finn-b-N14_CALCUKIDUR | ebi-a-GCST90016918 | class Lentisphaeria id.2250 | Maximum likelihood | 0.139 | 1.092 | 0.972 | 1.228 |
| finn-b-N14_CALCUKIDUR | ebi-a-GCST90016918 | class Lentisphaeria id.2250 | MR Egger | 0.114 | 2.434 | 0.948 | 6.253 |
| finn-b-N14_CALCUKIDUR | ebi-a-GCST90016918 | class Lentisphaeria id.2250 | Weighted median | 0.960 | 1.004 | 0.859 | 1.174 |
| finn-b-N14_CALCUKIDUR | ebi-a-GCST90016919 | class Melainabacteria id.1589 | Inverse variance weighted | 0.149 | 1.079 | 0.973 | 1.195 |
| finn-b-N14_CALCUKIDUR | ebi-a-GCST90016919 | class Melainabacteria id.1589 | Maximum likelihood | 0.146 | 1.080 | 0.974 | 1.198 |
| finn-b-N14_CALCUKIDUR | ebi-a-GCST90016919 | class Melainabacteria id.1589 | MR Egger | 0.796 | 0.891 | 0.386 | 2.057 |
| finn-b-N14_CALCUKIDUR | ebi-a-GCST90016919 | class Melainabacteria id.1589 | Weighted median | 0.168 | 1.095 | 0.963 | 1.245 |
[truncated: 103,623 more chars]
